# Mapping the global mRNA transcriptome during development of the murine first molar

**DOI:** 10.3389/fgene.2015.00047

**Published:** 2015-02-26

**Authors:** Maria A. Landin, Ståle Nygård, Maziar G. Shabestari, Eshrat Babaie, Janne E. Reseland, Harald Osmundsen

**Affiliations:** ^1^Department of Oral Biology, Faculty of Dentistry, University of OsloOslo, Norway; ^2^Bioinformatics Core Facility, Institute for Medical Informatics, Oslo University Hospital and University of OsloOslo, Norway; ^3^Department of Biomaterials, Institute for Clinical Dentistry, University of OsloOslo, Norway; ^4^The Biotechnology Centre of Oslo, University of OsloOslo, Norway

**Keywords:** gene expression, microarrays, metallothionein 1, ITPR3, qPCR, tooth development

## Abstract

The main objective of this study was to map global gene expression in order to provide information about the populations of mRNA species participating in murine tooth development at 24 h intervals, starting at the 11th embryonic day (E11.5) up to the 7th post-natal day (P7). The levels of RNA species expressed during murine tooth development were mesured using a total of 58 deoxyoligonucleotide microarrays. Microarray data was validated using real-time RT-PCR. Differentially expressed genes (*p* < 0.05) were subjected to bioinformatic analysis to identify cellular activities significantly associated with these genes. Using ANOVA the microarray data yielded 4362 genes as being differentially expressed from the 11th embryonic day (E11.5) up to 7 days post-natal (P7), 1921 of these being genes without known functions. The remaining 2441 genes were subjected to further statistical analysis using a supervised procedure. Bioinformatic analysis results for each time-point studied suggests that the main molecular functions associated with genes expressed at the early pre-natal stages (E12.5–E18.5) were cell cycle progression, cell morphology, lipid metabolism, cellular growth, proliferation, senescence and apoptosis, whereas most genes expressed at post-natal and secretory stages (P0–P7) were significantly associated with regulation of cell migration, biosynthesis, differentiation, oxidative stress, polarization and cell death. Differentially expressed genes (DE) not described earlier during murine tooth development; *Inositol 1, 4, 5-triphosphate receptor* 3 (*Itpr3*), metallothionein 1(*Mt1*), cyclin-dependent kinase 4 (*Cdk4*), cathepsin D (Ctsd)*, keratin complex 2, basic, gene 6a* (Krt2-6a), cofilin 1, non-muscle (*Cfl1*), cyclin 2 (*Ccnd2*), were verified by real-time RT-PCR.

## Introduction

During murine tooth development substantial changes occur within a time-span of 24 h. From E11.5 up to P7, i.e., in the course of 16 days the tooth germ develops from one layered oral epithelium into a phenotypic molar tooth. The developing murine tooth germ is therefore an excellent model for studying the time-course of gene expression in a rapidly, developing organ.

Differentiation requires participation of a large number of genes, the expression of which is regulated in time and space. The numbers and types of genes involved, however, vary depending on the developmental stage. In addition, miRNAs and lncRNAs are also known to be involved during tooth development (Jevnaker and Osmundsen, 2008; Michon et al., 2010; Michon, 2011) providing an additional layer of complexity.

Microarray studies generate vast amounts of gene expression data. Genes that exhibit significantly different levels of expression at different developmental stages during tooth development are termed differentially expressed (DE) genes; these must be isolated using appropriate statistical methods (Reiner et al., 2003; Smyth, 2004). Secondly it is of interest to determine biological processes, biochemical functions, and sub-cellular locations significantly associated with DE genes, linking DE genes to alterations in cellular physiology by mapping DE genes to gene ontology (GO) terms (Ashburner et al., 2000; Rhee et al., 2008).

More than 4300 mRNAs and some of their translated proteins have, so far, been detected during tooth germ development by microarray, *in situ* hybridization and immunocytochemistry (Jevnaker and Osmundsen, 2008; Landin et al., 2012). In a previous study (Landin et al., 2012) we used three pre-selected genes (Ambn, Amelx, and Enam) as starting point for profile search and identified 84 differentially expressed genes with a similar expression pattern as these enamel genes. However, the results of this study only show a tinny frame of the big picture of genetic events that occur during murine tooth development. Mapping global gene expression to capture the majority of genes involved during murine tooth development may provide an overview of the occurring genetic changes.

The global mapping of gene expression for each time-point studied may also reveal participation of novel genes or transcription factors as well as genes with unknown functions (Etokebe et al., 2009) during murine tooth development. Understanding the molecular cell biology during murine tooth development opens for development in novel bio-therapeutic strategies in dentistry.

In the current study we attempted to map the global gene expression in the molar murine tooth germ at each of 16 time-points by uploading the 2441 genes differentially (*P* ≤ 0.05) expressed at every time point studied (E11.5-P7). To interpret the resulting gene-expression data, we used Ingenuity Pathway analysis (IPA) (Kramer et al., 2014).

## Methods

### Experimental design

The global gene expression in tooth germs from wild type mice was monitored from embryonic day 11 (E11.5) up to 7 days after birth (P7) using a reference design; the 11th embryonic day was considered time point zero (T_0_) and all time-points studied were compared to E11.5. The sample size for each time point was *n* = 3–5 embryos/pups from three different mothers.

### Experimental animals

Pregnant Balb-c mice CD-1strain were used in this study. The day of vaginal plug was set to E0.5 Adult mice were sacrificed by cervical dislocation, the embryos or pups by decapitation. Embryos were staged according to the Theiler criteria as described in Landin et al. (2012). Animal housing (Scantainer ventilated cabinet Q-110) had 12 h light/dark cycle. The cabinet temperature was maintained at 21°C with a relative humidity of 55% (ScanClime plus). Fodder and water were supplied *ad lib*. The animals were kept according to the regulations of the Norwegian Gene Technology Act of 1994.

### Dissection of tooth germs and RNA extraction

Dissection, homogenization of whole tooth germs and total RNA extraction was carried out as previously described in Osmundsen et al. (2007) and Landin et al. (2012). At the pre-natal stages total RNA was isolated from 3 to 5 tooth germs. At post-natal stages batches of at least three tooth germs were used at each time point. RNA concentration was measured at 260 nm in a Nanodrop ND 1000 spectrophotometer. RNA fractions with the ratio of absorbance 260 and 280 nm around 2.0 and with RIN-values higher than 8.5 as measured using an Agilent Bioanalyzer (Agilent, Palo Alto, CA, USA) were used for analysis of gene expression using deoxyoligonucleotide microarrays and real-time RT-PCR.

### Complementary DNA synthesis and labeling

Complementary DNA (cDNA) was synthetized and indirect labeled from 1 μg total RNA using Genisphere Array 900™. Indirect labeling was used to avoid bias associated with differences in molecular size of the indicator molecules as previously described (Osmundsen et al., 2007; Landin et al., 2012).

### Microarray analysis of mRNAs isolated from tooth germs

Murine deoxyoligonucleotide (30 k)-microarrays were purchased from the NTNU Microarray Core Facility, Trondheim, Norway. The slides had been printed using the Operon murine v.3 oligo set (Qiagen GmbH, Hilden Germany). The arrays included probes for 10 different mRNAs from *Arabidopsis thaliana*. A spike mixture of 10 different mRNAs from *A. thaliana* (purchased from Stratagene, La Jolla, CA, USA) mixed in pairs at 10 different ratios, ranging from 0.1 to 5 was used to monitor the quality of the hybridisation. Each time point 3–6 biological replicates were subjected to independent microarray analysis. Each microarray was scanned in a Packard Bioscience Scanarray Lite microarray scanner (Perkin-Elmer). The Cy3 (543 nm) and Cy5 (645 nm) fluorescence signals were quantified by using the ScanArrayExpress v.3.0 software (Perkin-Elmer).

### Accession number

Raw and normalized microarray data have been deposited in the ArrayExpress database with reference E-MEX-3581.

### Microarray data preparation and normalization

For each microarray, measured net fluorescence intensities (median values, with background subtracted) were Lowess normalized. Spots with net intensity less than 200 across the entire time-course were filtered away. The filtered data were log_2_ transformed and subjected to median subtraction and z-score normalization (Quackenbush, 2002; Cheadle et al., 2003).

### Statistical analysis of microarray data

To find non-constant (non-zero) genes expressed during murine tooth development (E11.5-P7), statistical analysis of microarray data was carried out, using Spotfire v. 9 Microarray Analysis Software (TIBCO Software Inc, Palo alto, CAL, USA). Microarray data was derived from sets of three to six arrays at each time point. Data from a total of 58 arrays were combined into a single data file and treated as single color data to facilitate statistical analysis of time-courses. False discovery rate (FDR; 0.05) of Benjamini and Hochberg (Benjamini et al., 2001), was used to correct selection of genes for false positives. The ANOVA facility of the Spitfire program was used to select genes which exhibited statistically significant differences in levels of expression (*P* < 0.05) between the various developmental stages.

### Validation of microarray results using real-time RT-PCR

Expression of selected DE genes: *Inositol 1, 4, 5-triphosphate receptor* 3 (*Itpr3*), metallothionein 1(*Mt1*), cyclin-dependent kinase 4 (*Cdk4*), cathepsin D (*Ctsd*)*, keratin complex 2, basic, gene 6a* (*Krt2-6a*), cofilin 1, non-muscle (*Cfl1*), cyclin 2 (*Ccnd2*), were verified by real-time quantitative RT-PCR. These genes were selected because they are not described in literatur during tooth murine tooth development. The assays were carried out as described previously (Osmundsen et al., 2007; Landin et al., 2012) using Stratagene MX 3005P PCR instrument (Stratagene, La Jolla, CA, USA) using both biological and technical triplicates. The spesific primers are listed in Table 1. Statistical evaluation of the significance of differences between measured *Ct*-values was carried out using the REST 2009 program (Pfaffl et al., 2002a,b).

**Table 1:** **Sequences of deoxyoligonucleotide primers used for real-time RT-PCR assays**.

**Gene**	**Sequence of left primer**	**Sequence of right primer**
*Itp3*	5′-ATG CTG CAG GCC TAT GAG GAG-3′	5′-TAC AGA CTG CTT GCG GCT CAG-3′
*Mt1*	5′-CAG GGC TGT GTC TGC AAA G-3′	5′-GCT GGG TTG GTC CGA TAC TA-3′
*Cdk4*	5′-GTT TCT AGG CGG CCT GGA TT-3′	5′-CAG CTT GAC GGT CCC ATT AC-3′
*Ctsd*	5′-CCA CTG TCA GGG AAC TGG AT-3′	5′CTC CTT CAG ACA GGC AGA GG-3′
*Krt2-6a*	5′-AGG CTG CTG AAG GAG TAC CA-3′	5′-TCA ACC TGC ACT CCT CTC CT-3′
*Ccnd2*	5′-GGA GGT AAG GGA AGC ACT CC-3′	5′-CTC CTC GAT GGT CAA CAG GT-3′
*Cfl1*	5′-TCT ATG CCA GCT CCA AGG AT-3′	5′-TCT GGG GCT GTT AAG ATG CT-3′
*Lmna1*	5′-AGG ACC TCG AGG CTC TTC TC-3′	5′-CTC CTT CAG CGT CTG TAG CC-3′
*RpL27*	5′-GGG AAA GTG GTG GTG GTG CT-3′	5′-CAC CAG GGC ATG GCT GTA AG-3′

*The table lists genes, and their corresponding deoxyoligonucleotide primer pairs, used to measure mRNA levels in tooth germs at various time-points using real-time RT-PCR assays. Primers were designed to have melting point 60°C and product size of about 100 bp*.

### Bioinformatics analysis of differentially expressed genes

The 2441 differentially expressed genes with known Entrez Gene ID at all time- points, were uploaded onto IPA (Ingenuity Systems Inc., Redwood City, CAL, USA) (29). Bioinformatics core analysis was used to identify significant associations (*P* ≤ 0.05) with canonical pathways, signaling pathways and with molecular and cellular functions with clusters of differentially expressed genes as judged by Fisher's exact test. The core analysis was performed by uploading the ratios (Time_n_/E11.5) (*n* = E12.5-P7) of the global expression data directly from Spotfire. The identifier was the ENTREZ ID. The parameters chosen were: The reference set was the Ingenuity Knowledge Base (genes only), species [mouse, human and primary mouse cell culture (epithelial cells, odontoblasts, ameloblasts, and adipocytes)].

IPA Transcription Factor Analysis (Kramer et al., 2014) was used to identify the transcription factors associated with significant changes in gene expression during murine tooth development.

Network analysis (Thomas and Bonchev, 2010) was used to create graphical representations of molecules interacting at each time-point. The network size was set to 35 nodes/molecules. Network analysis also predicted the upstream and downstream effects of activation or inhibition on other molecules by applying expression values from the dataset (Supplementary data).

## Results

### Genes with expression level changing over time

Microarray results showed that a total 4362 of non-constant (non-zero) genes are expressed during murine tooth development from E11.5 up to P7; 1921 genes with unknown function and very highly expressed at all time-points with net fluorescence intensities above 10000 (results not shown) and 2441 differentially expressed genes at all time- points with known Entrez Gene ID. These 2441 genes were subjected to further bioinformatics analysis.

### Time courses of expression for selected genes as assayed using real-time RT-PCR

**Figure 1:**
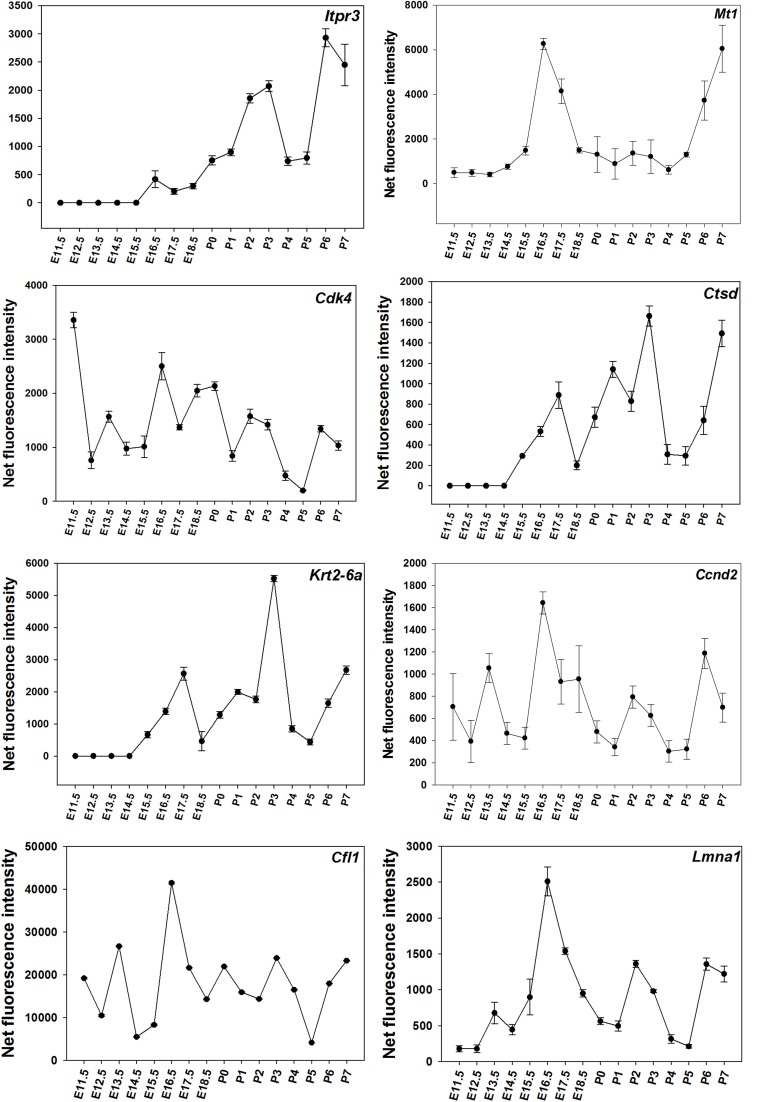
**Microarray results for for DE genes throughout the time-course**. The figure presents results showing the net normalized fluorescence intensities for DE genes monitored using microarrays with SD.

**Figure 2:**
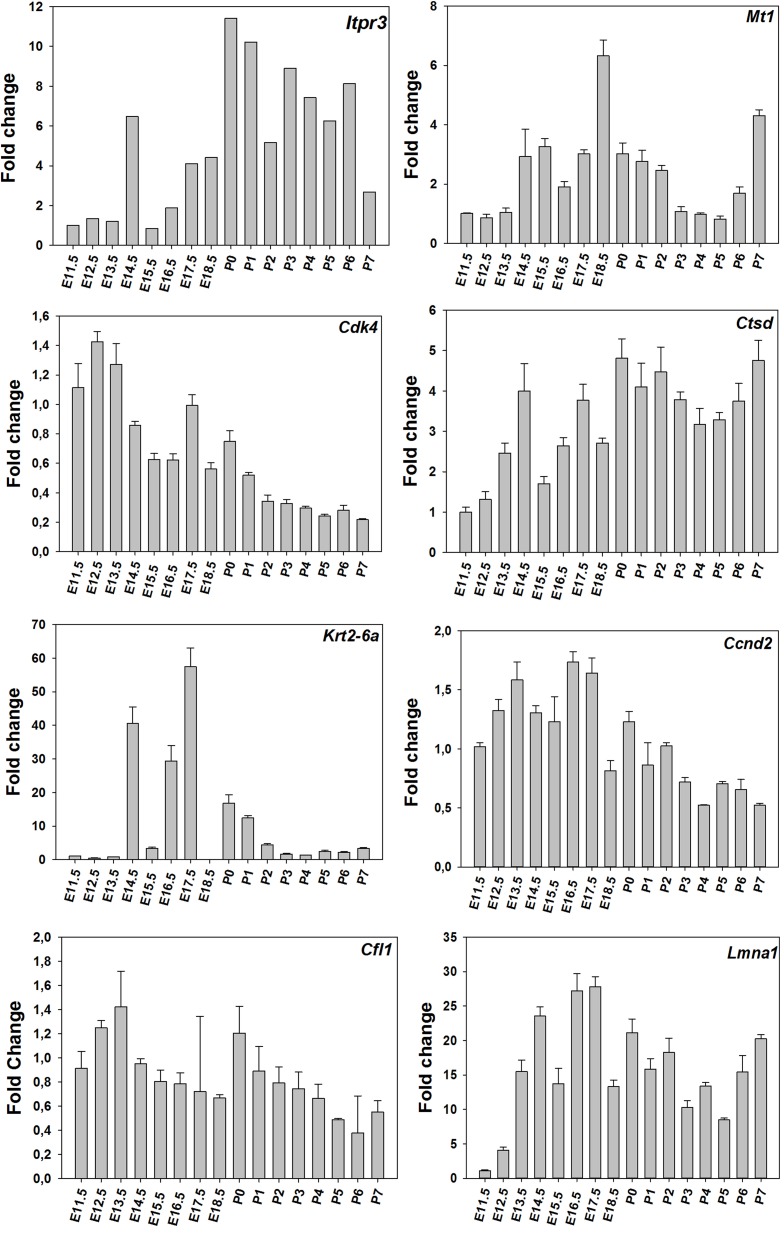
**RT-PCR results for the time-course**. Levels of selected mRNAs in total RNA isolated from the molar tooth germ at the various times of development using real-time RT-PCR.

Time-course of expression of selected genes (Figure 1) was also monitored using real-time RT-PCR. The results suggest that time-courses assayed by real-time RT-PCR show a similar trend to expression data obtained using microarrays (Figure 2).

### Bioinformatics analysis of the time-course ingenuity pathway analysis (IPA)

#### Transcription factor analysis

**Figure 3:**
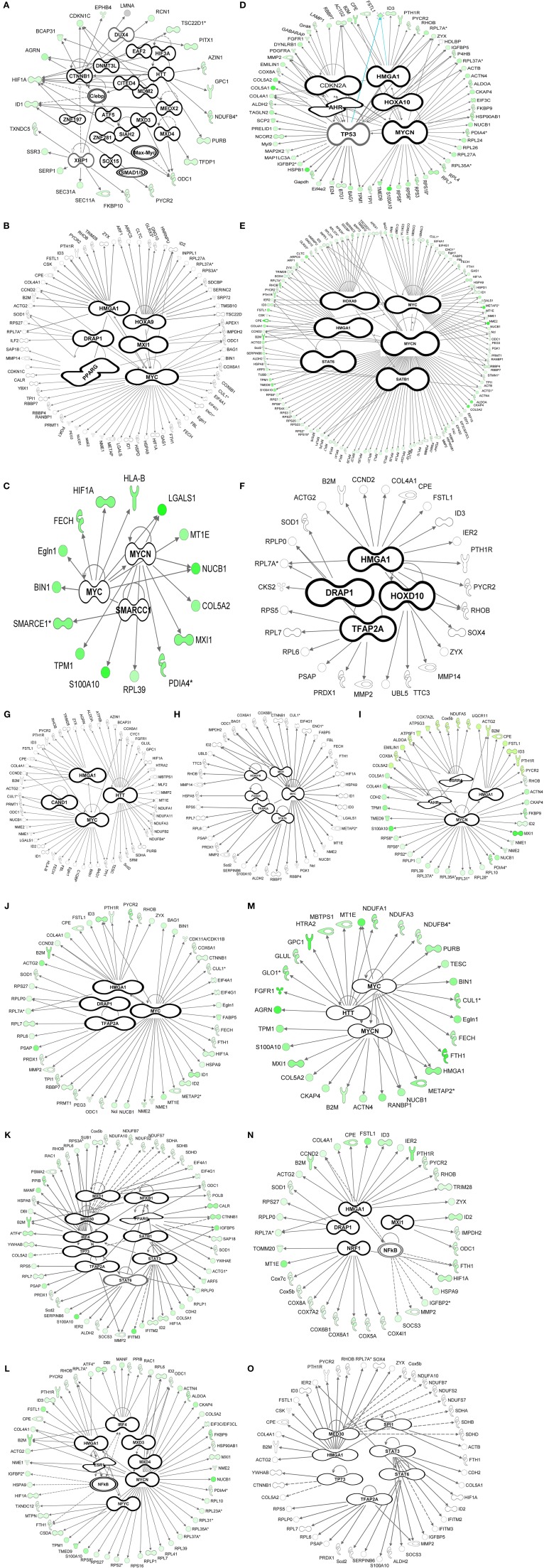
**Transcription factor analysis (IPA)**. Bioinformatics transcription factor analysis performed using Ingenuity Pathway Analysis (IPA) for the 4362 genes throughout the time-course **(A–O)** exhibiting a *p*-value of overlap <0.01 associating significantly ~1000 genes with 63 transcription factors. Pre-natal stages: Bud stages [**A** (E12.5), **B** (E13.5)]. Enamel knot stage [**C** (E14.5)]. Bell stages [**D** (E15.5), **E** (E16.5), **F** (E17.5), and **G** (E18.5)]. Day of birth (P0). Post-natal stages: Differentiation of odontoblasts **(I–K)**, start of mineralization: dentin deposition **(N–L)**, enamel deposition **(O)**.

Transcription factor analysis suggested that 19 transcription factors are involved in the transcription of 23 genes during the invagination of the epithelium into the mesenchyme at E12.5 (Table 2). The transcription factor (TF) Huntingtin (*Htt*) in the tooth germ regulated other transcription factors e.g., *Hif1a, Purb*, and *C/ebp* (Figure 3A).

**Table 2:** **Bioinformatic results for transcription factor analysis using IPA**.

**Time-point**	**TF**	**P- of overlap**	**TF role in cell**
BIOINFORMATIC RESULTS FOR TRANSCRIPTION FACTOR ANALYSIS (IPA) AT PRE-NATAL DAYS (E12.5–E18.5)			
E12.5	Atf5	1,41E-03	Expression, apoptosis, G2/M phase transition, growth, differentiation, survival, cell survival
	C/Ebp	1,41E-03	Differentiation, proliferation, expression, apoptosis, adipogenesis, transcription
	Cited4	8,28E-03	Unknown
	Ctnnb1	1,41E-03	Expression, proliferation, apoptosis, differentiation, transcription, transformation, growth, adhesion, migration
	Dnmt3l	8,28E-03	Unknown
	Dux4	1,65E-03	Unknown
	Eaf2	8,28E-03	Apoptosis, growth, stabilization, expression
	Hif3a	1,41E-03	Transactivation, quantity, activation
	Htt	8,67E-05	Cell death, apoptosis, expression in, degeneration, quantity, survival, activation
	Max-Myc	8,28E-03	Unknown
	Meox2	6,77E-06	Migration, size, transactivation in, G0/G1 phase transition, binding, activation, angiogenesis, apoptosis
	Mdm2	1,41E-03	Apoptosis, cell cycle progression, degradation, proliferation, ubiquitination, expression, growth, G1 phase, differentiation
	Mxd3	1,41E-03	Transformation, cell death, expression, differentiation, S phase, proliferation, survival
	Mxd4	1,41E-03	Unknown
	Siah2	8,28E-03	Apoptosis, degradation, association, activation, differentiation, transmigration, quantity
	Sox15	1,41E-03	Unknown
	Smad1/5	8,18E-03	Expression, migration
	Znf281	8,28E-03	Differentiation
	Znf197	1,41E-03	Unknown
	Xbp1	8,28E-03	Expression, cell death, production, survival, differentiation, transcription, degradation, endoplasmic reticulum stress response
E13.5	Drap1	2,37E-08	Assembly
	Hoxa9	2,37E-08	Expression in, quantity, transformation, proliferation, colony formation, number, differentiation, morphology, apoptosis
	Hmga1		Unknown
	Myc	3,81E-05	Apoptosis, proliferation, transformation, growth, cell cycle progression, expression, differentiation, S phase, death
	Mxl1	1,41E-03	Proliferation, function, transcription, cell cycle progression, expression, morphology, G2/M phase, S phase
	Pparg	6,77E-06	Differentiation, expression, adipogenesis, proliferation, apoptosis, growth, transcription, cell cycle progression, cell quantity, cell morphology
	Mxi1	6,77E-06	Transformation, proliferation, function, transcription, cell cycle progression, expression, morphology, G2/M phase, S phase
E14.5	Myc	8,28E-03	See E13.5
	Mycn	2,48E-05	Proliferation, apoptosis, transformation, expression, growth, differentiation, transactivation, transcription, survival
	Mxl1	1,78E-03	See E13.5
	Smarcc	1,60E-02	Apoptosis, remodeling, disassembly, development, expression, reorganization, binding, stabilization, ubiquitination
E15.5	Ahr	5,16E-04	Apoptosis, cell cycle progression, expression, differentiation, proliferation, quantity, homeostasis, transcription, function
	Cdkn2a	4,11E-09	Proliferation, apoptosis, cell cycle progression, growth, senescence, G1 phase, transformation, S phase, binding
	Hmga1	2,49E-04	Unknown
	Hoxa10	1,41E-03	Differentiation, proliferation, repression, morphology, development, expansion, G1 phase, mineralization
	Mycn	1,14E-14	See E14.5
	Tp53	2,48E-05	Apoptosis, cell cycle progression, proliferation, cell death, expression, growth, G1 phase, senescence, survival
E16.5	Drap1	4,11E-09	See E13.5
	Dysf	2,23E -3	Repair, morphology, fusion, size, healing, resealing, adhesion, expression
	Elavl1	2,94E-3	Translation, expression, number, phosphorylation, sensitivity, proliferation, growth, apoptosis
	Epo	1,71E-4	Proliferation, differentiation, apoptosis, colony formation, growth, production, survival, quantity, maturation
	Hmga1	9,84E-15	Unknown
	Hoxa9	8,28E-34	See E13.5
	Hif-1a	2,37E-08	Expression, apoptosis, proliferation, activation, transcription, differentiation, growth, cell death, migration
	Kras	7,6E-5	Apoptosis, transformation, growth, proliferation, expression, morphology, cell death, survival, colony formation
	Myc	5,6E-32	See E13.5
	Mycn	2,13E-32	See E14.5
	Stat6	3,33E-17	Differentiation, proliferation, expression, development, function, quantity, number, polarization, recruitment
	Psen1	9,6E-5	Apoptosis, quantity, expression, differentiation, formation, cell death, activation, migration
E17.5	Drap1	6,77E-06	See E13.5
	Hmga1	2,02E-07	Unknown
	Hoxd10	7,12E-24	Expression, migration, angiogenesis, polarization, invasion
	Tfap2a	2,58E-12	migration, growth, expression, apoptosis, cell death, proliferation, development, invasion, differentiation, invasion
	Xbp1	2,58E-12	Expression, cell death, survival, differentiation, transcription, degradation, endoplasmic reticulum expansion
	Rela	2,58E-12	Apoptosis, expression, proliferation, survival, activation, cell death, transactivation, transcription, migration
	Arnt	2,58E-12	Proliferation, expression, differentiation, migration, growth, apoptosis, G1 phase, glycolysis
	E2f1	2,58E-12	Apoptosis, proliferation, cell cycle progression, S phase, expression, G1/S phase transition, G1 phase, growth, cell death, cell quantity
	Max	2,58E-12	Apoptosis, transformation, growth, proliferation, mitogenesis, cell cycle progression, differentiation, migration, S phase
	Myod1	2,02E-07	Differentiation, cell cycle progression, myogenesis, expression, activation, growth, binding, survival, fate determination
	Nfkb	2,02E-07	Apoptosis, cell survival, proliferation, cell death transcription, differentiation, activation, growth
	Stat3	2,02E-07	Proliferation, expression, apoptosis, growth, differentiation, transformation, migration, survival, cell death
E18.5	Cand1	2,02E-07	Differentiation
	Hmga1	6,77E-06	Unknown
	Hnf1a	8,19E-06	Expression, transcription, apoptosis, activation, differentiation, proliferation, transactivation, cell number
	Ctnnb1	8,19E-06	Proliferation, apoptosis, differentiation, transcription, transformation, growth, activation, adhesion, migration
	Htt	8,19E-06	See E12.5
	Myc	2,58E-12	See E13.5
**BIOINFORMATIC RESULTS FOR TRANSCRIPTION FACTOR ANALYSIS (IPA) AT POST NATAL DAYS (P0–P7)**			
P0	Myc	2,83E-24	See E13.5
	Stat6	8,05E-12	See E16.5
	Tfap2a	1,64E-07	Migration, growth, expression, apoptosis, cell death, proliferation, development, invasion, differentiation, invasion
	Hoxd10	4,00E-07	Expression, migration, angiogenesis, polarization, invasion
	Mxl1	2,67E-05	See E13.5
	Foxo1	2,64E-07	Expression, transcription, apoptosis, transactivation, proliferation, activation, differentiation, binding, downregulation, ubiquitination
P1	Ahr	6,05E-10	Apoptosis, cell cycle progression, expression, differentiation, proliferation, quantity, homeostasis, morphology, transcription, function
	Esrra	1,00E-07	Differentiation, expression, number, proliferation, growth, migration, ossification, uptake, glycolysis
	Hmga1	6,16E-15	Unknown
	Mycn	2,60E-07	See E16.5
**Time-point**	**TF in the data set**	**P- of overlap**	**TF role in cell**
P2	Drap1	2,91E-04	Assembly
	Hmga1	1,61E-05	Unknown
	Myc	1,13E-27	See E13.5
	Tfap2a	2,70E-05	Migration, growth, expression, apoptosis, cell death, proliferation, development, invasion, differentiation, invasion
P3	Irf4	5,90E-12	Differentiation, number, proliferation, expression, development, lack, abnormal morphology, function, quantity, apoptosis
	Nfkb1	4,66E-06	Apoptosis, cell number, proliferation, function, differentiation, quantity, development, expression, cell death, activation
	Med30	3,64E-05	Unknown
	Mxd1	5,48E-05	Cell transformation, proliferation, apoptosis, S phase, G1 phase, expression in, growth, cell quantity, transcription
	Pparg	7,56E-07	Differentiation, expression, adipogenesis, proliferation, apoptosis, growth, transcription, cell cycle progression, quantity
	Stab1	5,80E-07	Proliferation, expression, differentiation, activation, quantity, organization, recruitment, number, apoptosis, development
	Stat3	5,31E-07	proliferation, expression, apoptosis, growth, differentiation, transformation, migration, survival, invasion
	Stat6	1,34E-11	See E16.5
	Tp73	6,43E-27	Apoptosis, expression, cell death, growth, cell cycle progression, proliferation, DNA damage response, colony formation, differentiation
	Tfap2a	5,56E-09	Migration, growth, expression, apoptosis, cell death, proliferation, development, differentiation, invasion
P4	Esr1		Expression, growth, proliferation, transcription, transactivation, phosphorylation, apoptosis, invasion, migration, binding
	Hmga1		Unknown
	Irf4	1,90E-14	Differentiation, number, proliferation, expression, development, lack, abnormal morphology, function, quantity, apoptosis
	Mycn	2,38E-11	See E14.5
	Mxd3	1,02E-13	See E12.5
	Mxd4		Unknown
	Nfkb	8,09E-06	Apoptosis, expression, survival, proliferation, transcription, cell death, activation, differentiation, growth
	Nfyc	3,25E-04	Expression, binding, transcription
P5	Myc	1,92E-17	See E13.5
	Htt	5,86E-03	See E12.5
	Mycn	5,20E-07	See E14.5
P6	Hmga1	1,30E-02	unknown
	Drap1	5,86E-03	See E13.5
	Nfkb	1,30E-12	See P4
	Nrf1	9,27E-06	Apoptosis, organization, oxidative stress response, cell death, proliferation, quantity, cell viability, expression, replication
	Mxl1	2,85E-03	See E13.5
P7	Hmga1	9,27E-06	Unknown
	Med30	1,13E-05	Unknown
	Stat3	2,07E-14	See P3
	Stat6	1,04E-11	See P3
	SPI1	1,89E-11	Differentiation, number, morphology, proliferation, apoptosis, expression, transcription
	Tp73	1,74E-25	See P3
	Tfap2a	2,32E-11	See P0

*The 2441 differentially expressed genes (DE) (p ≤ 0.05) were used to determine significant associations (p ≤ 0.01) with transcription factors (TF) prior to birth. TFs with p-value of overlap <0.01 where considered to be significantly associated with the DE genes from the dataset*.

*Abreviations: TF, Transcription factors; embryonic day (E12.5–E18.5)*.

At the early bud stage (E13.5) the number of transcription factors involved decreased to 6 compared to E12.5 (Figure 3B). In this early budding stage, transcription factor analysis show, that V-mycavian mielocytomatosis viral oncogene homolog (*myc*) regulated other transcription factors (*Hif1A, Eno1, Eif4g1, Eif4A1, Id1, Id2*, and *Hmga1*) (Figure 3B). *Myc* seems also to be regulated by *Drap1, Hox9*, and *Hmga1*. In addition *Hmga1* regulated the transcription factors *Id3* and *Trim28* (Figure 3B).

During the formation of the enamel knot (E14.5) the number of transcription factors decreased to 3 compared to E12.5–E13.5 (Figure 3C). At this embryonic stage of tooth development, myc seems to regulate the transcription factors *Hif1a*, *Smarcc1*, and *Mycn*. *Mycn* regulates *Mxl1* (Figure 3C).

At the early bell stage (E15.5) both the number transcription factors and genes regulated by TF increased (Figure 3D). Transcription factor analysis suggests that *Tp53, Ahr* and *Hoxa10*, may play an important role at this stage of tooth development. *Tp53* is regulated by *Ahr* and *Hoxa10* and in turn regulates *Bag1, Ncor2, Actg2*, and *Hmga1* (Figure 3D). During bell stages (E16.5–E17.5) the number of transcription factors decreased (Figures 3E,F) compared to E15.5 (Figures 3D,E). *Hmga1* regulated *Id3, Trim28*, and *Sox4*, while *Drap1* regulates *Ilf2* (Figure 3E).

At the late bell stage (E18.5) (Figure 3G) and post-natal stages (P0–P7) (Figures 3H–O) the number of transcription factors remains almost constant.

#### Network analysis

Genes expressed during placode formation (E12.5) were associated with network functions such as post-translational modification, cellular growth and proliferation, cell cycle and cell-to-cell signaling (Table 4 and Supplemental data pages 3–6).

During bud formation (E13.5) the genes expressed at this stage were associated with the following network functions: Post-transcriptional modification, protein synthesis and folding, cellular compromise, cell death (Table 4 and Supplemental data pages 7–23).

The genes expressed at enamel knot (E14.5) Table 4 and Supplemental data pages 24–32) and late cap stages (E15.5–E16.5), were associated with networks associated with functions listed in Table 4 and Supplemental data pages 33–52).

Genes expressed at bell stages E17.5–E18.5 were significantly associated with networks connected to lipid metabolism, nucleic acid and carbohydrate metabolism, small molecule biochemistry, senescence and energy production (Table 4 and Supplemental data pages 53–82).

Genes expressed at the post-natal stages (P0–P7) were associated with networks involved in DNA/RNA replication, cell-to-cell signaling, protein synthesis, cell death and free radical scavenging (Table 4 and Supplemental data pages 84–197).

#### Biological functions and canonical pathways

**Figure 4:**
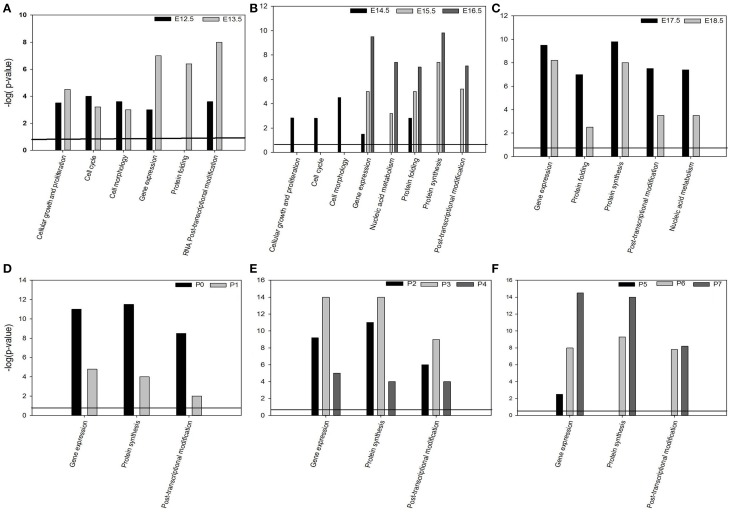
**Bioinformatics analysis of the global mRNA transcriptome during murine tooth development using Molecular & Cellular functions (A–F) significantly (*p* < 0.005) associated with DE genes expressed during murine tooth development**.

Bioinformatics analysis of the global gene expression significantly (*p* = 0.05) associated the 2441 differentially genes(*p* ≤ 0.05) of the dataset with the molecular and cellular functions: “Gene expression” (all time-points studied) (Figures 4A–F), “cellular growth and proliferation” (E12.5–E13.5) (Figure 4A), “RNA post-transcriptional modification” (E-12.5–E13.5 and P0–P7) (Figures 4A,D–F), “cell cycle,” “cell morphology” (E12.5-E14.5) (Figures 4A,B), “protein folding” (E12.5–E18.5) (Figures 4A–C), “nucleic acid metabolism” (E15.5-P7) (Figures 4B–F).

The canonical pathways associated with the 2441 DE genes are listed in Table 3.

**Table 3:** **Canonical pathways**.

**Time-point**	**Canonical pathway**	**Molecules in the dataset**	***p*-value**
E12.5	Polyamine Regulation	*Azin1,Odc1*	7,56E-03
	Oxidative Phosphorylation	*Ndufs6,Cox7a2l,Atp6v1b2*	8,73E-03
	Urea Cycle and Metabolism of Amino Groups	*Pycr2,Odc1 Glud1,Aldh18a1,Pycr2*	1,38E-02
	mTOR Signaling	*Eif3g,Rhoq,Eif3j,Hif1a*	2,54E-02
	N-Glycan Biosynthesis	*Tusc3,Stt3a,Rpn2,Ddost*	2,73E-02
E13.5	Oxidative Phosphorylation	*Atp5d,Uqcr11,Cox8a,Atp5l,Uqcrb,Ndufb8,Atp5g2,Ndufa1,Ndufa5,Atp5g1,Ndufs6,Uqcrfs1,Atp5j,Atp5c1,Atp5b,Ndufa6,Ndufb7,Uqcrc2,Ndufa12,Ndufb2,Ndufa4,Atp6v0b,Uqcrh,Cox6a1,Cox7a2l,Ndufa2,Ndufs2,Atp6v1g1,Cox4i1,Atp5i,Sdha,Ndufc1,Cox6b1,Ndufv3,Ndufa13,Ndufv2,Ndufs8,Ndufa11,Cox5a,Sdhd,Ndufa3,Atp6v1b2*	4,19E-34
	EIF2 Signaling	*Rpl24,Rpl11,Rpl27a,Rps18,Rpl22l1,Rps8,Rpl14,Eif4a2,Rps21,Eif4g1,Eif3c/Eif3cl,Rps23,Rpl7,Rps7,Rps27,Rpl6,Rps3a,Map2k2,Rps3,Rpl18,Rpl4,Rpl3,Rpl34,Rps2,Rps19,Rpl23a,Rps27l,Rps29,Eif3g,Fau,Rpl39,Eif4a1,Rpl32,Eif3i,Rplp1*	1,51E-22
	Ubiquinone Biosynthesis	*Ndufa4,Ndufc1,Ndufv3,Ndufb8,Ndufa1,Ndufa2,Ndufa13,Ndufa5,Ndufv2,Ndufs8,Ndufa11,Ndufa6,Bckdha,Ndufb7,Ndufs6,Ndufa12,Ndufs2,Ndufa3,Ndufb2*	8,01E-16
	mTOR Signaling	*Rps18,Rps8,Eif4a2,Rps21,Hif1a,Eif4g1,Eif3c/Eif3cl,Rps23,Rps7,Rps27,Rps3a,Rhob,Rps3,Rps2,Rps19,Rac1,Rps27l,Rps29,Eif3g,Fau,Rhoq,Prkci,Eif4a1,Eif3i*	5,21E-09
E14.5	Synaptic Long Term Potentiation	*Calm1,Prkci,Ppp1r10,Map2k2,Gna11,Ppp1r11*	1,42E-03
	Urea Cycle and Metabolism of Amino Groups	*Glud1,Aldh18a1,Pycr2, Srm,Glud1,Aldh18a1*	5,14E-03
	Pyruvate Metabolism	*Bckdha,Acat1,Dld,Glo1*	8,83E-03
	Melatonin Signaling	*Calm1,MAP2K2*	1,02E-02
	Insulin Receptor Signaling	*Prkci,Rhoq,Ppp1r10,Map2k2,Ppp1r11*	
E15.5	Oxidative Phosphorylation	*Sdha,Atp5j,Uqcrh,Ndufv3,Cox8a,Cox7a2l,Ndufa1,Atp6v1e1,Atp5c1,Ndufs8,Ndufc2, Uqcrc1,Ndufb2*	1,39E-10
	EIF2 Signaling	*Rpl11,Rpl27a,Rps18,Rpl22l1,Rps8,Eif4a2,Rpl26,Rps21,Eif4g1,Eif3c/Eif3cl,Rpl7,Rps11,Rpl6,Map2k2,Eif1ax,Eif5,Rpl18,Rpl3,Eif3h,Rpl34,Rps2,Rps19,Rpl23a,Rplp0,Rps29,Eif3g,Fau,Rps4x,Rpl28,Rpl39,Eif4a1,Rpl32,Eif3i,Rps25,Rplp1,Rpl41,Rps14*	8,45E-08
	Regulation of Actin-based Motility by Rho	*Actr3,Pfn1,Myl6,Rhob,Myl12b,Arpc2,Actb,Rac1,Pfn2,Actg2*	1,58E-07
	Integrin Signaling	*Actb,Rac1,Ilk,Tln1,Actr3,Arf5,Map2k2,Rhob,Myl12b,Arpc2,Zyx,Actg2,Actn4,Cttn*	1,47E-06
E16.5	Oxidative Phosphorylation	*Atp5d,Uqcr11,Cox8a,Atp5l,Uqcrb,Ndufb8,Atp5g2,Ndufa1,Ndufb10,Ndufa5,Atp5g1,Ndufs6,Uqcrfs1,Atp5f1,Atp5g3,Atp5j,Atp5c1,Atp5b,Ndufa6,Ndufb7,Ndufa12,Ndufb2,Ndufa4,Cox7b,Atp6v0b,Uqcrh,Cox6a1,Cox7a2l,Ndufa2,Ndufb9,Ndufc2,Ndufs2,Atp6v1g1,Atp5i,Cox4i1,Sdha,Ndufc1,Cox7a2,Cox6b1,Ndufv3,Ndufa13,Atp6v1e1,Ndufs8,Ndufv2,Ndufa11,Cox5a,Ndufa3,Uqcrc1,Atp6v1b2*	4,55E-43
	EIF2 Signaling	*Rpl11,Rpl27a,Rps18,Rpl22l1,Rps8,Eif4a2,Rpl26,Rps21,Eif4g1,Eif3c/Eif3cl,Rpl7,Rps11,Rpl6,Map2k2,Eif1ax,Eif5,Rpl18,Rpl3,Eif3h,Rpl34,Rps2,Rps19,Rpl23a,Rplp0,Rps29,Eif3g,Fau,Rps4x,Rpl28,Rpl39,Eif4a1,Rpl32,Eif3i,Rps25,Rplp1,Rpl41,Rps14*	8,95E-25
	Ubiquinone Biosynthesis	*Ndufa4,Ndufc1,Ndufv3,Ndufb8,Ndufa1,Ndufa2,Ndufa13,Ndufb10,Ndufb9,Ndufa5,Ndufv2,Ndufs8,Ndufa11,Ndufc2,Ndufa6,Bckdha,Ndufb7,Ndufs6,Ndufa12,Ndufs2,Ndufa3,Ndufb2*	3,72E-17
	Regulation of eIF4 and p70S6K Signaling	*Eif3h,Rps2,Rps18,Rps19,Rps8,Eif4a2,Paip2,Rps21,Eif4g1,Eif3c/Eif3cl,Rps11,Eif3g,Rps29,Rps4x,Fau,Map2k2,Eif1ax,Eif4a1,Eif3i,Rps25,Rps14*	1,32E-09
E17.5	Oxidative Phosphorylation	*Same genes as E16.5*	4,55E-43
	EIF2 Signaling	*Eif3h,Rps2,Rps18,Rps19,Rps8,Eif4a2,Paip2,Rps21,Eif4g1,Eif3c/Eif3cl,Rps11,Eif3g,Rps29,Rps4x,Fau,Map2k2,Eif1ax,Eif4a1,Eif3i,Rps25,Rps14*	8,95E-25
	Ubiquinone Biosynthesis	*Ndufa4,Ndufc1,Ndufv3,Ndufb8,Ndufa1,Ndufa2,Ndufa13,Ndufb10,Ndufb9,Ndufa5,Ndufv2,Ndufs8,Ndufa11,Ndufc2,Ndufa6,Bckdha,Ndufb7,Ndufs6,Ndufa12,Ndufs2,Ndufa3,Ndufb2*	3,72E-17
	Regulation of eIF4 and p70S6K Signaling	*Eif3h,Null,Rps2,Rps18,Rps19,Rps8,Null,Eif4a2,Paip2,Rps21,Eif4g1,Eif3c/Eif3cl,Rps11,Eif3g,Rps29,Rps4x,Fau,Null,Map2k2,Eif1ax,Eif4a1,Null,Eif3i,Rps25,Rps14*	1,32E-09
E18.5	Oxidative Phosphorylation	*Cox7b,Atp6v0b,Cox6a1,Uqcrh,Cox8a,Atp5l,Cox7a2l,Ndufa1,Ndufa2,Ndufb10,Ndufb9,Ndufc2,Ndufs6,Uqcrfs1,Ndufs2,Atp6v1g1,Sdha,Ndufv3,Atp6v1e1,Ndufs8,Atp5b,Ndufa11,Ndufa6,Ndufa3,Ndufb2,Atp6v1b2*	1,85E-21
	EIF2 Signaling	*Rpl11,Rps18,Rpl22l1,Rps8,Rpl26,Rps23,Eif3c/Eif3cl,Rpl7,Rps3a,Map2k2,Eif5,Ppp1ca,Rpl18,Eif3h,Rpl34,Rps2,Rps19,Rpl23a,Rplp0,Rps29,Eif3g,Rps4x,Rpl28,Rpl39,Rpl32,Rplp1,Eif3l,Rpl41*	3,95E-19
	Ubiquinone Biosynthesis	*Ndufv3,Ndufa1,Ndufa2,Ndufb10,Ndufb9,Null,Ndufs8,Ndufc2,Ndufa11,Bckdha,Ndufa6,Ndufs6,Ndufs2,Ndufa3,Ndufb2*	4,18E-11
	Regulation of Actin-based Motility by Rho	*Pfn1,Cfl1,Myl6,Actb,Arpc5,Rac1,Pfn2,Actr3,Rhoq,Rhob,Arpc2,Arpc3,Actg2*	1,66E-08
P0	Oxidative Phosphorylation	*Atp5d,Uqcr11,Cox8a,Atp5l,Uqcrb,Ndufb8,Atp5g2,Ndufa1,Ndufb10,Ndufa5,Atp5g1,Ndufs6,Atp5f1,Atp5g3,Atp5j,Ndufs7,Atp5c1,Atp5b,Ndufa6,Uqcrc2,Ndufa12,Ndufb2,Ndufa4,Cox7b,Atp6v0b,Uqcrh,Cox6a1,Cox7a2l,Ndufa2,Ndufb9,Ndufc2,Ndufs2,Atp6v1g1,Atp5i,Cox4i1,Sdha,Ndufc1,Cox6b1,Cox7a2,Ndufv3,Ndufa13,Atp6v1e1,Ndufs8,Ndufv2,Ndufa11,Cox5a,Sdhd,Uqcrc1,Atp6v1b2*	1,52E-44
	EIF2 Signaling	*Rpl11,Rpl27a,Eif4a2,Rps23,Eif3c/Eif3cl,Rps7,Rps3a,Eif4g2,Map2k2,Eif1ax,Ppp1ca,Rpl3,Rpl23a,Rps29,Rps4x,Fau,Rpl39,Rps25,Rpl41,Rplp1,Rps18,Rpl22l1,Rps8,Rpl26,Rps21,Eif4g1,Rpl7,Rpl6,Rps27,Rps3,Rpl4,Eif3h,Rpl34,Rps2,Rps19,Eif3j,Eif3g,Rpl28,Eif3i,Rpl32,Rps14*	8,13E-30
	Ubiquinone Biosynthesis	*Ndufa4,Ndufc1,Ndufs7,Ndufv3,Ndufb8,Ndufa1,Ndufa2,Ndufa13,Ndufb10,Ndufa5,Ndufb9,Ndufv2,Ndufs8,Ndufa11,Ndufc2,Ndufa6,Bckdha,Ndufs6,Ndufa12,Ndufs2,Ndufb2*	3,1E-17
	Regulation of Actin-based Motility by Rho	*Rhoq,Actr3,Pfn1,Rhob,Myl6,Cfl1,Myl12b,Arpc2,Actb,Pfn2,Rac1,Arpc3,Actg2*	4,26E-26
	Regulation of eIF4 and p70S6K Signaling	*Rps18,Rps8,Eif4a2,Rps21,Eif4g1,Eif3c/Eif3cl,Rps23,Rps7,Rps27,Rps3a,Eif4g2,Map2k2,Eif1ax,Rps3,Eif3h,Rps2,Rps19,Eif3j,Eif3g,Rps29,Rps4x,Fau,Eif3i,Rps25,Rps14*	1,75E-13
P1	Oxidative Phosphorylation	*Atp6v0b,Uqcrh,Cox6a1,Uqcr11,Cox8a,Atp5l,Uqcrb,Cox7a2l,Atp5g2,Ndufa1,Ndufb10,Ndufa5,Ndufb9,Atp6v1g1,Atp5f1,Atp5i,Cox4i1,Atp5g3,Sdha,Ndufc1,Cox7a2,Ndufv3,Ndufa13,Atp6v1e1,Ndufv2,Ndufs8,Ndufa11,Ndufa6,Uqcrc2,Cox5a,Ndufa3,Ndufb2,Atp6v1b2*	8,39E-31
	Ubiquinone Biosynthesis	*Ndufc1,Ndufv3,Ndufa1,Ndufa13,Ndufb10,Ndufa5,Ndufb9,Ndufs8,Ndufv2,Ndufa11,Ndufa6,Ndufa3,Ndufb2*	1,39E-10
	EIF2 Signaling	*Rpl34,Rps2,Eif3j,Rps8,Rps21,Rps29,Map2k2,Rpl28,Rpl39,Eif5,Rpl32,Eif3i,Rplp1*	4,58E-07
	Integrin Signaling	*Rapgef1,Capn6,Ralb,Ilk,Arf1,Actr3,Arf5,Rhoq,Rhob,Map2k2,Myl12b,Arpc2,Arpc3,Actg2,Actn4,Cttn*	2,56E-05
P2	Oxidative Phosphorylation	*Atp5d,Uqcr11,Cox8a,Atp5l,Uqcrb,Ndufb8,Atp5g2,Ndufa1,Ndufb10,Ndufa5,Ndufs6,Uqcrfs1,Atp5f1,Atp5g3,Atp5j,Atp5c1,Atp5b,Ndufa6,Uqcrc2,Ndufa12,Ndufb2,Atp6v0b,Uqcrh,Cox6a1,Cox7a2l,Ndufa2,Ndufb9,Ndufc2,Ndufs2,Atp6v1g1,Cox4i1,Atp5i,Sdha,Ndufc1,Cox7a2,Ndufv3,Ndufa13,Atp6v1e1,Ndufv2,Ndufs8,Ndufa11,Cox5a,Uqcrc1,Atp6v1b2*	5,12E-40
	EIF2 Signaling	*Rpl11,Rpl27a,Eif4a2,Rps23,Eif3c/Eif3cl,Rps11,Rps7,Map2k2,Eif1ax,Ppp1ca,Pabpc1,Rpl3,Rpl23a,Rplp0,Rps29,Rps4x,Fau,Rpl41,Rplp1,Rpl24,Rps18,Rpl22l1rps8,Rpl26,Rps21,Eif4g1,Rpl7,Rpl6,Rps27,Rps3,Rpl18,Rpl4,Rpl34,Rps2,Rps19,Eif3g,Rpl28,Eif4a1,Rpl32,Eif3l,Rps14*	2,12E-30
	Ubiquinone Biosynthesis	*Ndufc1,Null,Ndufv3,Null,Ndufb8,Ndufa1,Ndufa2,Ndufa13,Ndufb10,Null,Ndufb9,Ndufa5,Ndufv2,Ndufs8,Ndufa11,Ndufc2,Ndufa6,Bckdha,Ndufs6,Ndufa12,Ndufs2,Null,Ndufb2*	8,16E-17
	Regulation of eIF4 and p70S6K Signaling	*Rhoq,Actr3,Pfn1,Rhob,Myl6,Cfl1,Myl12b,Arpc2,Actb,Null,Pfn2,Rac1,Arpc3,Actg2*	6,71E-12
P3	Oxidative Phosphorylation	*Atp5d,Uqcr11,Cox8a,Atp5l,Atp5g2,Uqcrb,Ndufb8,Ndufa1,Ndufb10,Ndufa5,Atp5g1,Ndufs6,Uqcrfs1,Atp5f1,Atp5g3,Atp5j,Ndufs7,Atp5c1,Atp5b,Ndufa6,Uqcrc2,Ndufb7,Ndufa12,Ndufb2,Ndufa4,Cox7b,Sdhb,Atp6v0b,Uqcrh,Cox6a1,Cox7a2l,Ndufa2,Ndufb9,Ndufc2,Ndufs2,Atp6v1g1,Atp5i,Cox4i1,Sdha,Ndufc1,Cox7a2,Cox6b1,Ndufv3,Ndufa13,Atp6v1e1,Ndufs8,Ndufv2,Ndufa11,Cox5a,Sdhd,Ndufa3,Uqcrc1,Atp6v1b2*	8,64E-46
	EIF2 Signaling	*Rpl11,Rpl27a,Eif4a2,Rps23,Eif3c/Eif3cl,Rps11,Rps7,Rps3a,Eif4g2,Map2k2,Eif1ax,Eif5,Ppp1ca,Pabpc1,Rpl3,Rpl23a,Rplp0,Rps29,Rps4x,Fau,Rpl39,Rps25,Rpl41,Rplp1,Rpl24,Rps18,Rpl22l1,Rps8,Rpl26,Rpl14,Rps21,Eif4g1,Rpl7,Rpl6,Rps27,Rps3,Rpl18,Rpl4,Eif3h,Rpl34,Rps2,Rps19,Eif3j,Rps27l,Eif3g,Rpl28,Eif4a1,Eif3i,Rpl32,Rps14*	1,06E-37
	Ubiquinone Biosynthesis	*Ndufa4,Ndufb8,Ndufa1,Ndufa2,Ndufb10,Ndufb9,Ndufa5,Ndufc2,Bckdha,Ndufs6,Ndufs2,Ndufc1,Ndufs7,Ndufv3,Ndufa13,Ndufv2,Ndufs8,Ndufa11,Ndufa6,Ndufb7,Ndufa12,Ndufa3,Ndufb2*	3,77E-19
	Regulation of eIF4 and p70S6K Signaling	*Rps18,Rps8,Eif4a2,Rps21,Paip2,Eif4g1,Rps23,Eif3c/Eif3cl,Rps11,Rps7,Rps27,Rps3a,Eif4g2,Map2k2,Eif1ax,Rps3,Pabpc1,Eif3h,Rps2,Rps19,Eif3j,Rps27l,Rps29,Eif3g,Rps4x,Fau,Eif4a1,Eif3i,Rps25,Rps14*	1,41E-16
P4	Oxidative Phosphorylation	*Ndufa4,Atp6v0b,Uqcrh,Cox6a1,Atp5d,Uqcr11,Cox8a,Atp5l,Ndufb8,Cox7a2l,Ndufa1,Ndufa2,Ndufa5,Atp5g1,Atp6v1g1,Atp5f1,Atp5g3,Sdha,Ndufc1,Cox6b1,Ndufv3,Ndufa13,Atp6v1e1,Atp5c1,Ndufv2,Atp5b,Ndufs8,Ndufa11,Uqcrc2,Ndufb7,Cox5a,Sdhd,Ndufa3,Uqcrc1,Ndufb2,Atp6v1b2*	4,28E-35
	Ubiquinone Biosynthesis	*Ndufa4,Ndufc1,Ndufv3,Ndufb8,Ndufa1,Ndufa2,Ndufa13,Ndufa5,Ndufv2,Ndufs8,Ndufa11,Bckdha,Ndufb7,Ndufa3,Ndufb2*	1,66E-12
	EIF2 Signaling	*Rps2,Rpl22l1,Eif3j,Rps8,Eif4a2,Rpl23a,Eif3c/Eif3cl,Null,Rpl7,Rps29,Eif3g,Rpl6,Rps27,Rpl39,Rpl32,Rplp1,Rpl41*	7,71E-09
	Regulation of Actin-based Motility by Rho	*Pfn1,Rhoq,Myl6,Rhob,Myl12b,Arpc2,Rac1,Pfn2,Arpc3,Actg2*	1,97E-05
P5	Oxidative Phosphorylation	*Atp6v1e1,Atp6v0b,Atp6v0c,Atp6v1f,Ndufb4,Atp5l,Ndufa3,Atp6v1g1,Ndufa1,Atp6v1b2,Ndufa2*	6,17E-07
	Ubiquinone Biosynthesis	*Bckdha,Ndufb4,Ndufa3,Ndufa1,Ndufa2*	1,04E-03
	Integrin Signaling	*Capn6,Rhoq,Ralb,Ilk,Arpc3,Tln1,Actn4*	9,3E-03
	Lipid Antigen Presentation by CD1	*B2m,Calr,Ap2a1,Psap,Canx*	2,32E-02
	Hepatic Stellate Cell Activation	*Vegfb,Myl6,Fgfr1,Pdgfra,Igfbp5*	2,53E-02
P6	Oxidative Phosphorylation	*Atp6v0c,Ndufa10,Atp5d,Uqcr11,Cox8a,Atp5l,Uqcrb,Ndufb8,Atp5g2,Ndufa1,Ndufa5,Atp5g1,Ndufs6,Uqcrfs1,Atp5f1,Atp5g3,Atp5j,Ndufs7,Atp5c1,Atp5b,Atp5e,Ndufa6,Uqcrc2,Ndufa12,Cyc1,Ndufb2,Ndufa4,Atp6v0b,Uqcrh,Cox6a1,Ndufb4,Cox7a2l,Ndufa2,Atp5h,Ndufb9,Atp6v1f,Ndufs2,Atp6v1g1,Cox4i1,Atp5i,Sdha,Ndufc1,Cox7a2,Cox6b1,Ndufv3,Ndufa13,Atp6v1e1,Uqcr10,Ndufs8,Ndufv2,Ndufa11,Cox5a,Sdhd,Ndufa3,Ndufa7,Atp6v1b2*	1,03E-40
	EIF2 Signaling	*Rpl27a,Rps18,Eif1,Rps8,Eif4a2,Rpl26,Eif4g1,Eif3c/Eif3cl,Rpl7,Rps7,Rps27,Eif4g2,Map2k2,Eif1ax,Eif5,Rps16,Rps3,Rpl4,Rpl34,Eif3f,Rps2,Rps19,Eif3j,Rpl23a,Rplp0,Rps29,Eif3g,Fau,Rpl10,Rpl39,Eif4a1,Rpl32,Rplp1*	3,41E-16
	Ubiquinone Biosynthesis	*Ndufa4,Ndufc1,Ndufa10,Ndufs7,Ndufv3,Ndufb4,Ndufb8,Ndufa1,Ndufa2,Ndufa13,Ndufb9,Ndufa5,Ndufv2,Ndufs8,Ndufa11,Ndufa6,Bckdha,Ndufs6,Ndufa12,Ndufs2,Ndufa3,Ndufa7,Ndufb2*	1,37E-15
	Regulation of eIF4 and p70S6K Signaling	*Eif3f,Rps2,Rps18,Eif1,Rps19,Eif3j,Rps8,Eif4a2,Paip2,Eif4g1,Eif3c/Eif3cl,Rps29,Rps7,Eif3g,Fau,Rps27,Eif4g2,Map2k2,Eif1ax,Rps16,Eif4a1,Rps3*	7,13E-08
P7	Oxidative Phosphorylation	*Atp6v0c,Ndufa10,Atp5d,Uqcr11,Cox8a,Atp5l,Atp5g2,Uqcrb,Ndufb8,Ndufa1,Ndufb10,Ndufa5,Atp5g1,Ndufs6,Uqcrfs1,Atp5f1,Atp5g3,Atp5j,Ndufs7,Atp5c1,Atp5b,Atp5e,Ndufa6,Uqcrc2,Ndufb7,Ndufa12,Cyc1,Ndufb2,Ndufa4,Cox7b,Sdhb,Atp6v0b,Uqcrh,Cox6a1,Ndufb4,Cox7a2l,Ndufa2,Atp5h,Atp6v1f,Ndufc2,Ndufs2,Atp6v1g1,Atp5i,Cox4i1,Sdha,Ndufc1,Cox7a2,Cox6b1,Ndufv3,Ndufa13,Atp6v1e1,Uqcr10,Ndufs8,Ndufv2,Ndufa11,Cox5a,Sdhd,Ndufa3,Uqcrc1,Atp6v1b2,Ndufa7*	4,72E-46
	EIF2 Signaling	*Rpl11,Rpl27a,Eif1,Eif4a2,Eif3c/Eif3cl,Rps11,Rps3a,Map2k2,Eif1ax,Eif5,Ppp1ca,Rpl3,Rpl23a,Rplp0,Rpl37,Rps29,Rps4x,Fau,Rpl10,Rpl39,Rps25,Rpl41,Rplp1,Rps18,Rpl22l1,Rps8,Rpl26,Rpl14,Rps21,Eif4g1,Rpl7,Rpl6,Rps27,Rps16,Rps3,Rpl18,Rpl4,Eif3h,Eif3f,Rpl34,Rps2,Rps19,Rps27l,Eif3g,Rps5,Rpl28,Eif4a1,Eif3i,Rpl32,Eif3l,Rps14*	9,55E-32
	Ubiquinone Biosynthesis	*Ndufa4,Ndufa10,Ndufb4,Ndufb8,Ndufa1,Ndufa2,Ndufb10,Ndufb9,Ndufa5,Ndufc2,Bckdha,Ndufs6,Ndufs2,Ndufc1,Ndufs7,Ndufv3,Ndufa13,Ndufv2,Ndufs8,Ndufa11,Ndufa6,Ndufb7,Ndufa12,Ndufa3,Ndufb2,Ndufa7*	4,23E-18
	Regulation of eIF4 and p70S6K Signaling	*Rps18,Eif1,Rps8,Eif4a2,Rps21,Paip2,Eif4g1,Eif3c/Eif3cl,Rps11,Rps27,Rps3a,Map2k2,Eif1ax,Rps16,Rps3,Eif3h,Eif3f,Rps2,Rps19,Rps27l,Eif3g,Rps29,Rps4x,Fau,Rps5,Eif4a1,Eif3i,Rps25,Eif3l,Rps14*	1,09E-12

*Signaling or metabolic pathways (IPA) significantly associated with DE genes during murine tooth development*.

**Table 4:** **Biological functions associated with network analysis**.

**Top biofunctions**	**E12.5**	**E13.5**	**E14.5**	**E15.5**	**E16.5**	**E17.5**	**E18.5**	**P0**	**P1**	**P2**	**P3**	**P4**	**P5**	**P6**	**P7**
Amino acid metabolism	–	–	–	–	–	–	x	–	–	–	X	–	–	–	–
Carbohydrate metabolism	X	–	–	X	–	–	X	X	X	X	–	X	X	X	–
Cell cycle	X	X	X	–	X	X	X	X	X	X	X	X	–	–	X
Cell death	–	X	–	–	X	X	X	X	X	X	–	X	X	X	X
Cell morphology	–	X	X	X	–	X	X	–	X	–	–	–	X	–	X
Cell-to- cell signaling	X	–	X	–	–	–	–	–	X	–	–	X	X	–	–
Cellular assembly and organization	–	–	X	X	X	X	X	X	X	X	X	X	X	X	X
Cellular compromise	–	X	–	X	–	–	X	X	X	–	X	X	X	X	X
Cellular development	X	X	X	X	X	X	X	X	X	X	X	X	–	X	X
Cellular function and maintenance	–	–	X	X	X	X	X	X	X	X	X	X	X	X	X
Cellular growth and proliferation	X	X	X	X	X	X	X	X	X	X	X	X	–	X	X
Cellular movment	X	–	X	X	X	–	X	–	X	–	–	X	X	–	X
Connective tissue development	X	–	–	X	–	X	X	X	X	–	X	–	–	X	X
DNA replication	–	X	–	X	X	X	X	–	X	X	X	–	–	X	X
Embryonic developmemt	–	X	–	–	–	X	–	X	X	X	–	–	–	X	–
Energi production	–	X	–	X	X	X	–	X	X	X	X	X	–	X	X
Free radical scavenging	–	–	–	–	–	–	–	X	–	X	–	–	–	–	–
Gene expresssion	–	X	–	–	X	X	X	X	X	X	X	X	–	X	X
Hematological system development	–	–	–	–	–	X	–	–	–	–	–	–	–	–	–
Lipid metabolism	–	–	–	–	X	X	–	X	X	X	X	X	–	–	X
Molecular transport	–	X	–	–	X	X	X	X	X	X	X	X	X	X	X
Nucleic acid metabolism	–	X	–	X	X	X	X	X	X	X	X	X	–	X	X
Organ morphology	–	–	–	–	–	–	–	–	X	X	–	–	–	X	X
Organismal development	–	–	X	–	–	–	X	–	–	–	–	–	–	–	–
Post-transcriptional modification	–	–	X	–	–	–	–	X	X	X	X	X	–	X	X
Post-translational modification	–	X	–	–	–	–	X	–	–	–	–	–	–	–	–
Proliferation	–	–	–	–	–	–	–	–	–	–	–	–	–	–	–
Protein degradation	–	–	–	–	–	–	–	X	–	–	–	–	–	–	–
Protein degradation	–	X	–	–	–	–	–	–	–	–	–	–	–	–	–
Protein folding	–	X	–	–	–	–	–	X	–	–	X	X	–	–	X
Protein synthesis	–	X	–	–	X	X	X	X	X	X	X	X	–	X	X
Protein trafficking	–	–	–	–	–	X	X	–	–	X	–	X	–	X	X
RNA post-transcriptional modification	X	X	–	X	X	X	X	X	X	X	X	–	–	X	–
RNA replication	–	–	–	–	–	–	–	–	–	–	–	–	–	X	–
Scenescence	–	–	X	–	X	X	X	–	–	–	–	–	–	–	–
Skeletal and muscular development	–	–	–	–	–	–	–	–	–	–	–	X	–	–	X
Small molecule biochemistry	–	X	–	–	X	X	X	X	X	X	X	X	X	X	X
Tissue development	–	X	–	–	X	X	–	X	X	X	X	–	–	–	–
Tissue morphology	–	–	X	–	–	X	X	X	–	–	–	–	–	X	–
Vitamin and mineral metabolism	–	–	–	–	–	–	–	–	X	–	–	–	–	–	–

*The table shows a summary of the biological functions significantly associated with the networks resulting from bioinformatics time-course network analysis presented as supplementary data*.

## Discussion

Transcription factor and network analysis of the 2441 differentially expressed genes suggests that during the embryonic stages of murine tooth development (E12.5–E13.5) cell proliferation and cell death are highly regulated e.g., expression of transcription regulators like Huntingtin (*Htt), Hif1a, Purb, Cbp1, C/ebp, Myc, and Id1*. The epithelial and mesenchymal cells during bud- and bell-stages, proliferate, migrate, adhere and communicate through tight junction signaling as shown by the expression and regulation of *Ccnd2* on the data set. *Ccnd2* also plays a role in Wnt/β-catenin signaling pathway (Liu and Millar, 2010). Wnt/β-catenin is known to play a central role for the morphogenesis of ectodermal appendages such as teeth, hair and exocrine glands (Haara et al., 2012).

During early murine tooth development, ectodermal placodes proliferate and invaginate into the mesenchyme to form an epithelial bud (E12.5–E13.5). The proliferating epithelial cells form a controlled invasion front into the mesenchyme and hold a correct temporal and spatial pattern in the growing bud; this process requires genes that control migration and adhesion between cells e.g., *Ctnnb1*. Migration of epithelial cells into the underlying mesenchyme requires changes in the cytoskeleton enabling the epithelial cells to migrate and invade the mesenchyme. The cytoskeleton provides both structural scaffolding for cells and a transportation network, where particles move along microtubule and actin highways powered by molecular motors that burn the cellular fuel, ATP (Ikuta et al., 2014).

The number of cells in the growing epithelium bud/bell is also controlled either by apoptosis *(Hif3a, Hif1a, C/Ebp, Ctnnb1, Hoxa9, Hoxa10, Myc, and Eaf2*) or arrest of cell cycle (*Meox2, Mdm2 and Mxd3 and Mxi1*) (E12.5–E18.5). Cyclin d2 (encoded by *Ccnd2*) is expressed in dental pulp and periodontal ligament cells (Liu et al., 2009). It is also known that *Ccnd2* activates C*dk4/6*, allowing the cells to progress through the G1-S checkpoint (Morsczeck et al., 2009). Epithelial cells also invade and displace the underlying mesenchyme. Mesenchymal cells probably use senescence as a trigger of tissue re-modeling in order to allow the epithelial cells to invade and replace mesenchymal tissue. Senescent cells arrest their own proliferation, recruit phagocytic immune cells and promote tissue renewal. Developmental senescence is well studied process during mammalian embryonic development (Munoz-Espin and Serrano, 2014). We can speculate that during early murine tooth development the epithelial cells at bud (E12.5–E13.5), cap (E14.5–E15.5) and bell stages (E16.5–E18.5) migrate into the mesenchyme where senescence, followed by clearance and then regeneration allows the growing epithelial derived bud/bell to grow into and displace the mesenchymal tissue. This is probably modulated by e.g., *Cdkn2a, Foxo, Smarcc*, and *TP53* (E12.5–E15.5). *MycN* is the cytoplasmic form of Myc where interacts with α- and β-tubulins and is expressed in differentiating cells (Conacci-Sorrell et al., 2010). Transcription factor analysis suggests that *MycN* regulates different genes at the enamel knot stage E14.5 early bell stages E15.5–E16.5 and late bell stage (P0). At post natal stages P4–P5 *MycN* may in addition to participate in cell proliferation and control of cell cycle progression be involved in the differentiation of mesenchyme cells into odontoblasts.

Microarray and qPCR results suggest that metallotionin 1 (*Mt1*) is highly expressed during the bell stage (E16.5) and post-natal stages (P5–P7). Very little is known about the expression of metallothionein 1 in the developing murine teeth prior to mineralization. Metallothioneins together with zinc transporters control zinc (Zn) homeostasis (Nartey et al., 1987). Zn is an essential trace element indispensable in cellular processes for embryonic and postnatal development in mammals (Kitabayashi et al., 2010). Zn deficiency causes growth retardation, reduced bone volume, dental decay. Zinc equilibrium is also required for odontoblast differentiation and dentin formation during dentinogenesis (Lin et al., 2011).

During differentiation stages (Wang et al., 2013) (P0–P5) and early mineralization stages (P6–P7) in addition to cell migration apoptosis and cell death, mesenchymal cells facing the epithelial cells at the basal side of the growing bell start to differentiate, elongate following by secretion of dentin around P4–P5, triggering secretion and mineralization initiation by the epithelial cells now differentiating into ameloblasts (P6–P7). Some transcription factors like *Myc, Mycn, Htt Stat6, Mxi1*, and *Drap1* seem to regulate different genes at the post-natal stages (P0, P2, P4, P5, and P6) compared to the embryonic stages (E12.5, E13.5, and E16.5). *Esr1* (P4) encode for an estrogen receptor. Estrogen and its receptors are essential for epithelial cell development, proliferation, suggesting that not only genes and their products but also hormones play an important during murine tooth development (Wang et al., 2013). Low estrogen production is associated with increased production of *Tgfa*, interleukins 1, 6, 8, and 10 leading to periodontal disease (Tezal et al., 2000; Dvorak et al., 2009; Zhang et al., 2011).

It is important to point out that levels of mRNA do not necessarily correlate with levels of translated protein (protein biosynthesis) from the same mRNA (Pascal et al., 2008). The quantitation of levels of mRNA and protein are complementary and also necessary for a complete understanding of how altered gene expression affects cellular physiology (Greenbaum et al., 2003), in addition to miRNAs, long non-coding RNAs (lncRNAs) (Okazaki et al., 2002; Batista and Chang, 2013) that do not code for functional proteins (Rinn and Chang, 2012) may play an important role in spatial positioning of the epithelial/mesenchymal cells during murine tooth development adding layers of complexity to the study of murine tooth development.

## Author contributions

ML performed microarray, RT-PCR and bioinformatics analysis. Prof. HO provided exceptional scientific resources, guidance and incredible scientific inspiration. MS helped with qPCR. SN contributed with bioinformatics expertise. EB performed qPCR oligoprimers synthesis and analysis. JR helped with fruitfully discussions editing the manuscript.

### Conflict of interest statement

The authors declare that the research was conducted in the absence of any commercial or financial relationships that could be construed as a potential conflict of interest.
