# Supplementary material for: Mapping the global mRNA transcriptome during development of the murine first molar
Source: Front Genet. 2015 Feb 26;6:47. doi: 10.3389/fgene.2015.00047 (PMC4362327; doi:10.3389/fgene.2015.00047)

Supplementary Material

Mapping the global mRNA transcriptome during development of the murine first molar

Maria A. Landin[1], Ståle Nygård[4], Maziar Shabestari[2], Eshrat Babaie[3], Janne E. Reseland[2] and Harald Osmundsen[1]

[1]Department of Oral Biology, Faculty of Dentistry, University of Oslo, Norway

[2]Department of Biomaterials, Institute for Clinical Dentistry, University of Oslo, Norway

[3]The Biotechnology Centre of Oslo, University of Oslo, Norway

[4]Bioinformatics core facility, Institute for medical informatics, Oslo University Hospital and University of Oslo

*** Correspondence:** Maria A. dos Santos Silva Landin, Department of Oral Biology, Faculty of Dentistry,

University of Oslo, Norway

mariaal@odont.uio.no
dosantla@online.no

1. Supplementary Data

Detailed network analysis using IPA for each othe the 16-time-points studied was used as basic tool to try to interpret the complex events occurring during murine tooth development. Due to the huge amount of networks generated for each time-point we present the resulting networks as supplementary data divided in three main categories (pre-natal): 1) prenatal stages: Placode and bud stage (E12.5-E13.5), 2) cap stage (E14.5) and 3) bell stages (E15.5-E18.5) (network analysis of pre-natal stages) and 4) post-natal stages (network analysis of post-natal stages).

2. Supplementary Figures

Legends to supplementary data

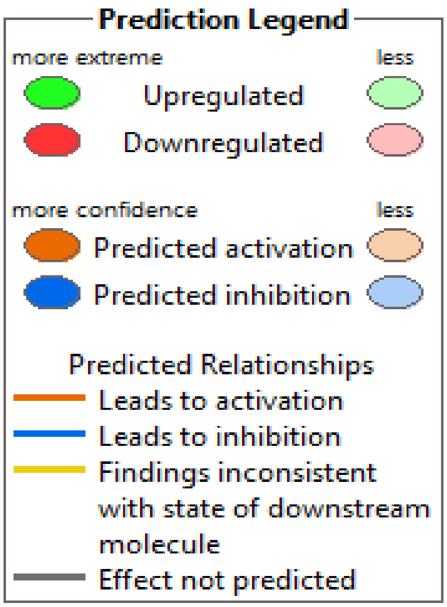

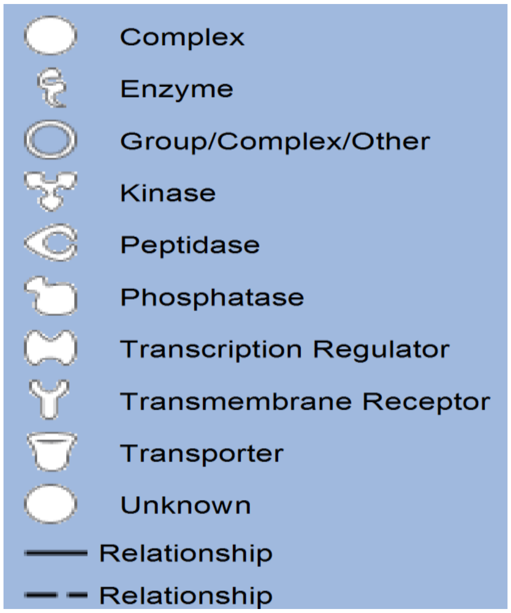

Supplemtary fig.1 Networks associated with genes expressed at placode (E12.5) and bud stage (E13.5)

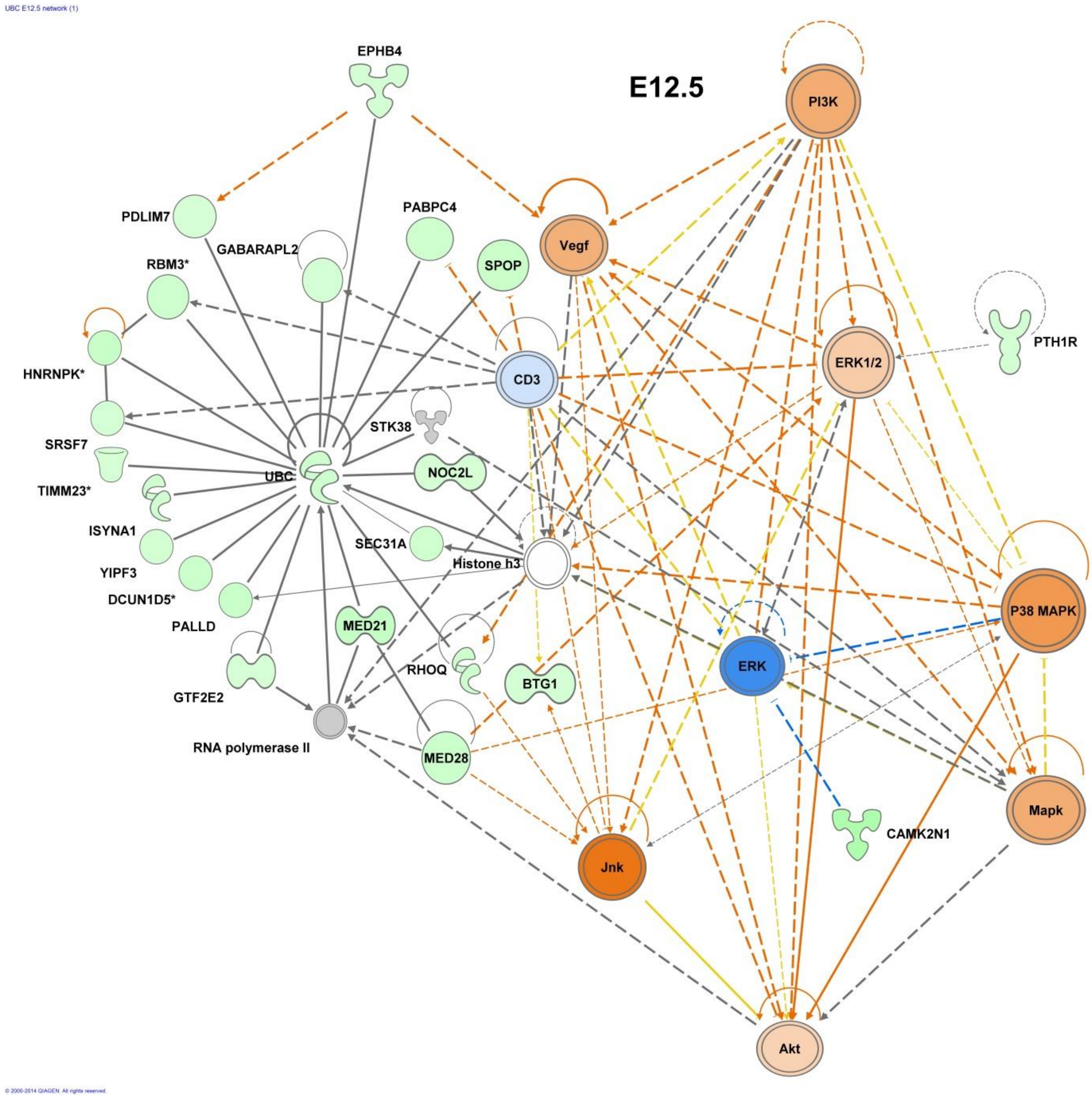

Network 1

RNA post-transcriptional modification, cell cycle, carbohydrate metabolism

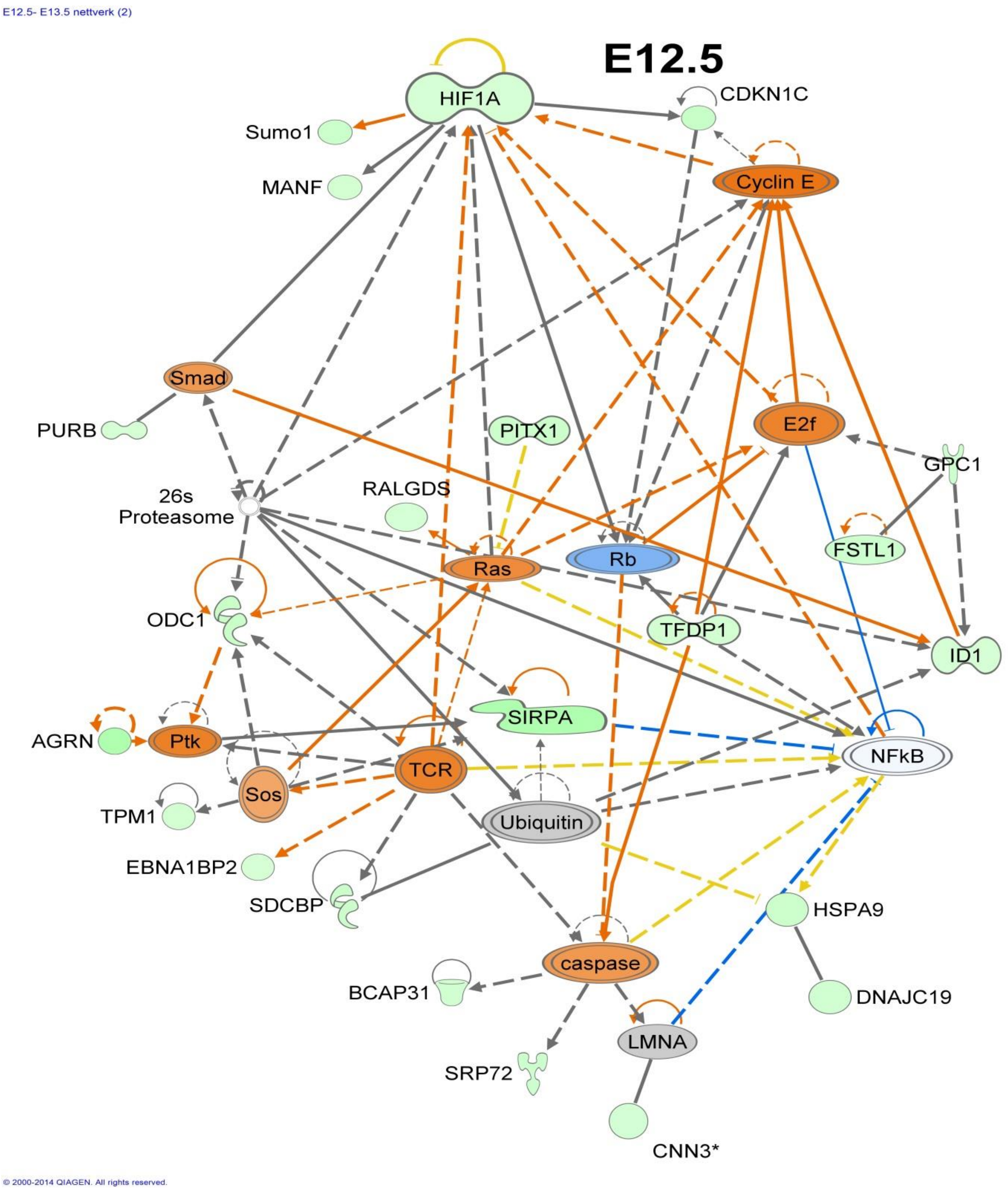

Network 2

Cellular development, cellular growth and proliferation, connective tissue development and function

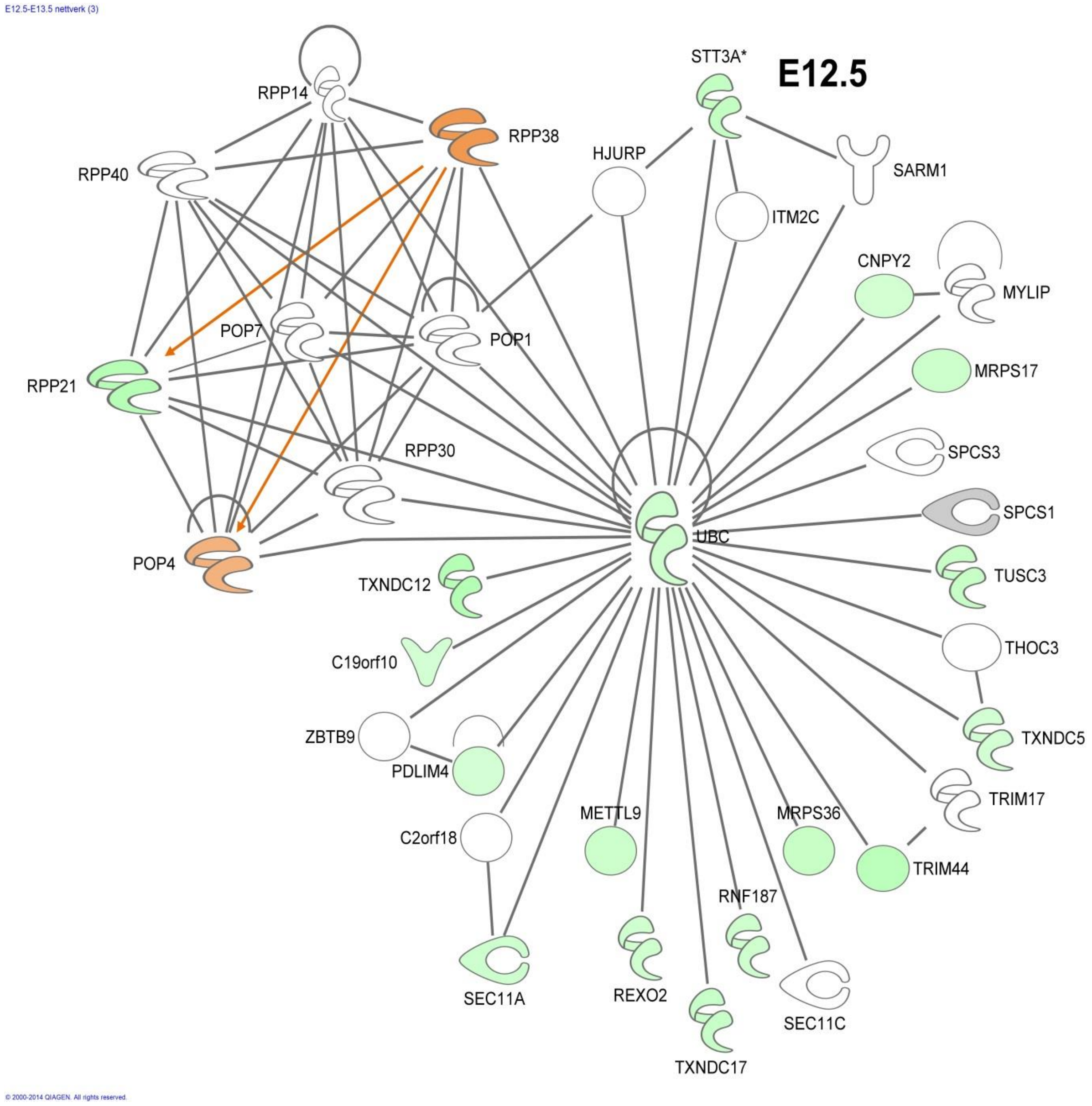

Network 3

Cellular growth and proliferation

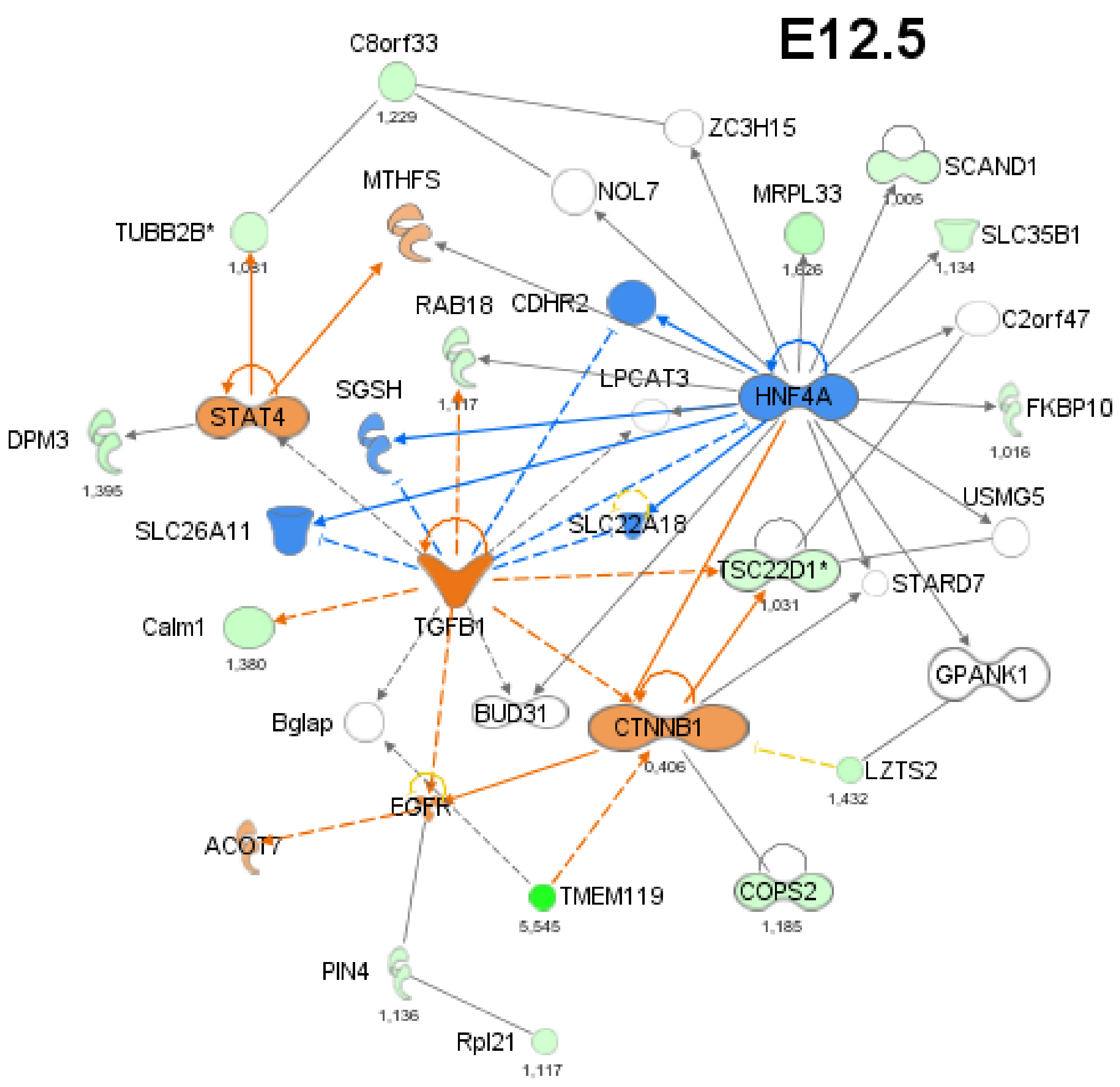

Network 4

Cellular movment, cell cycle, cell-to-cell signaling and interaction

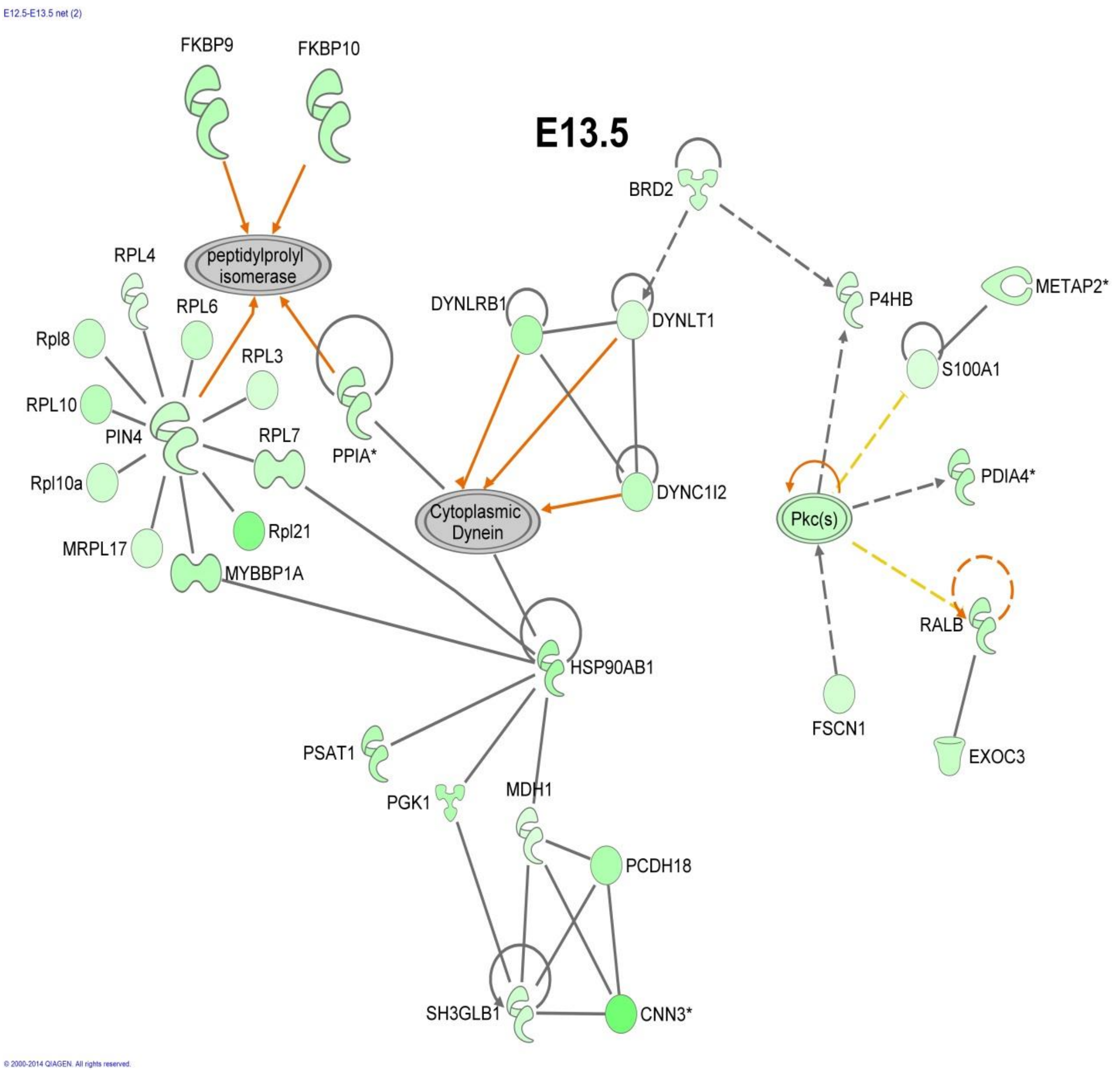

Network 1

Post-translational modification, protein folding

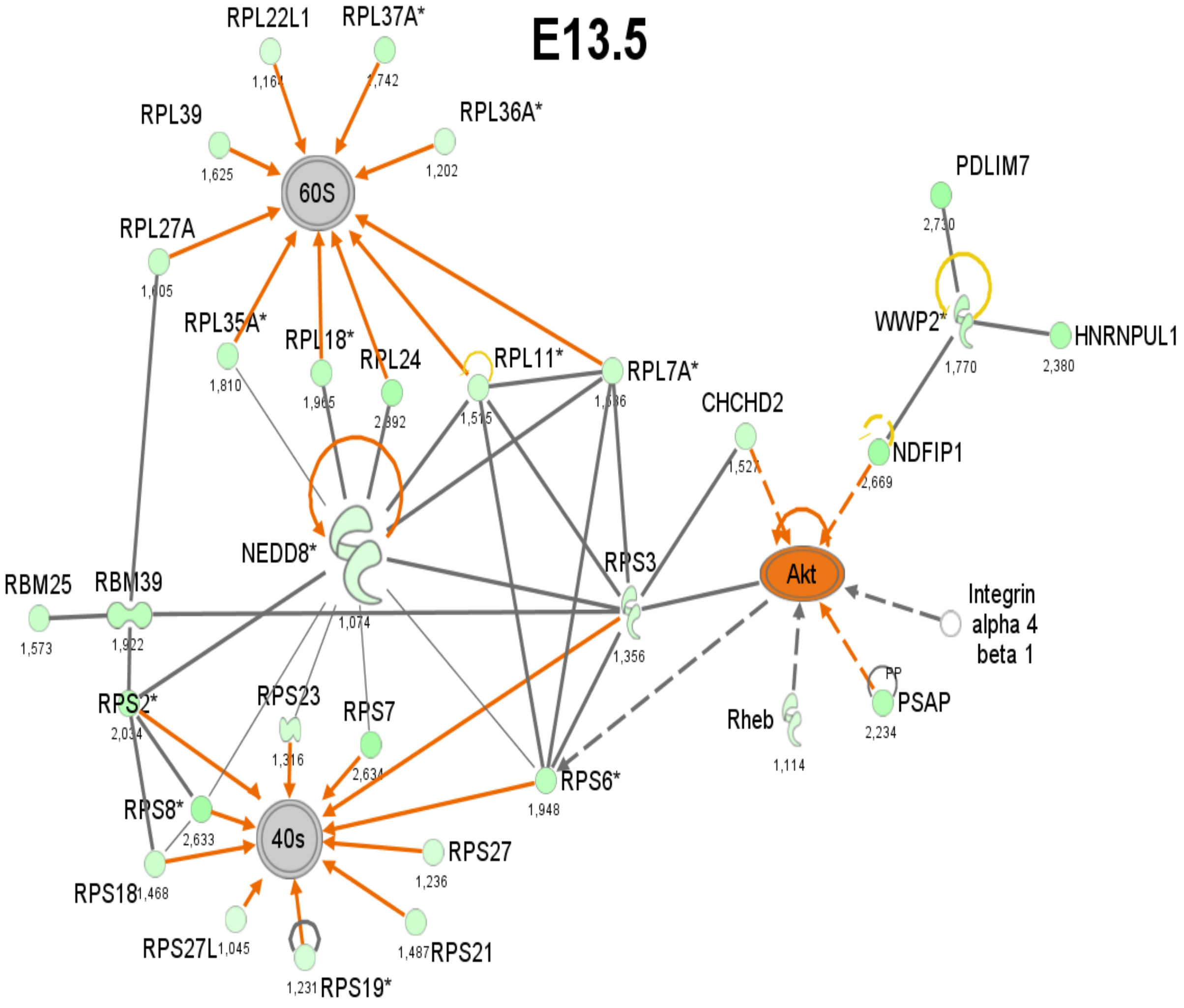

Network 2

RNA post-transcriptional modification, protein synthesis

E12.5-E13.5 net (4)

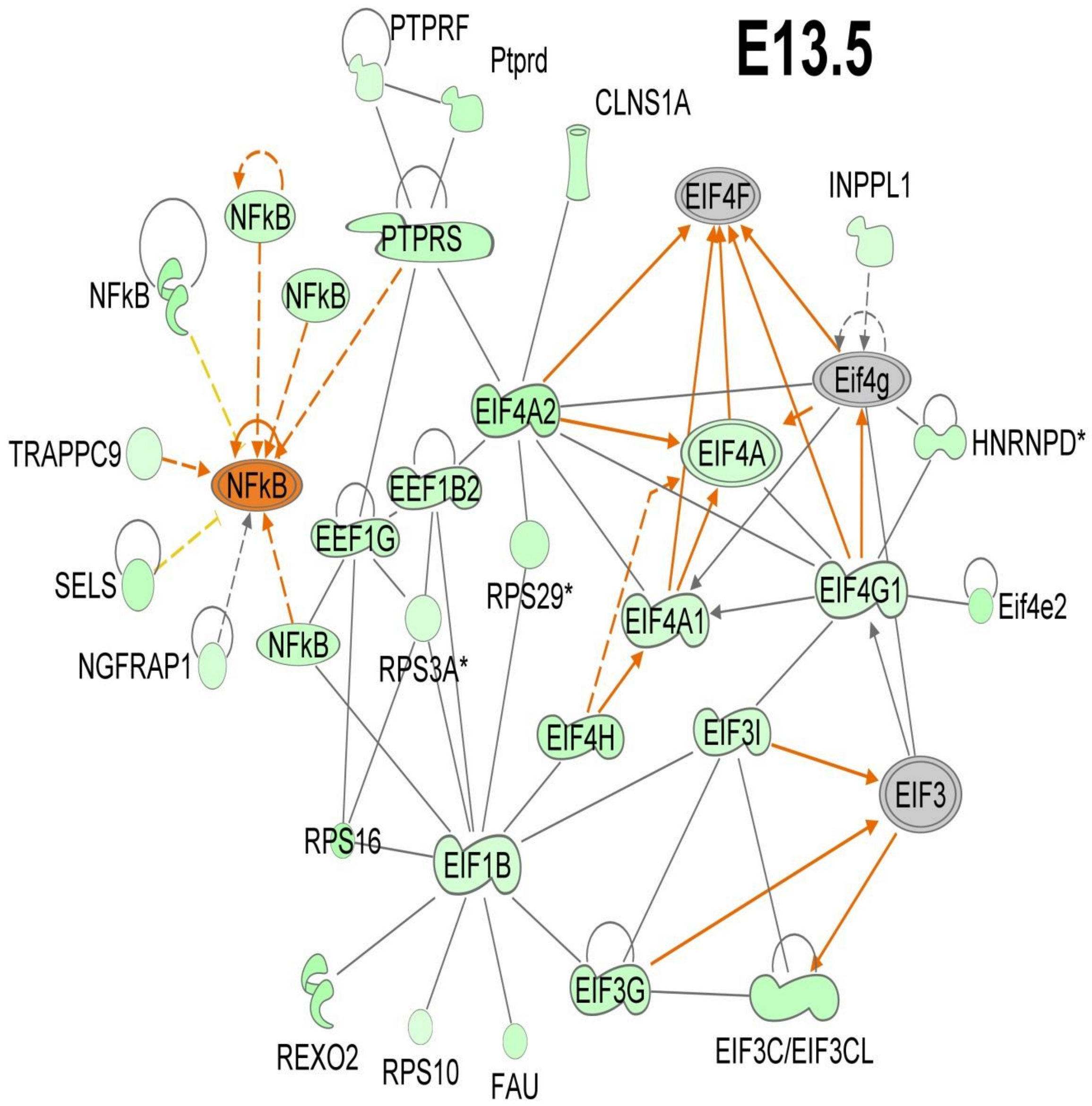

© 2000-2014 QIAGEN. All rights reserved.

Network 3

Gene expression, protein synthesis

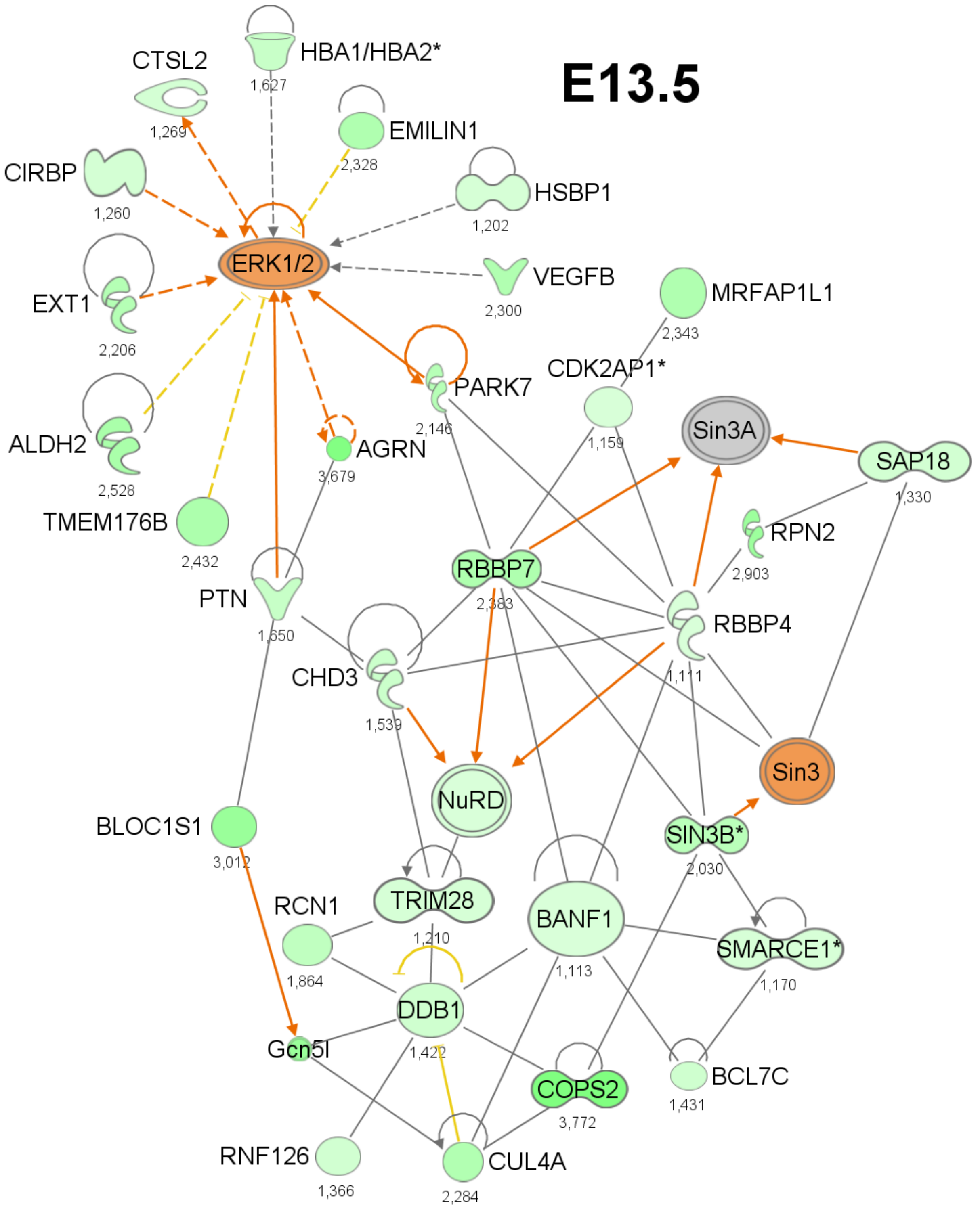

Network 4

Cell morphology, cell cycle, DNA replication

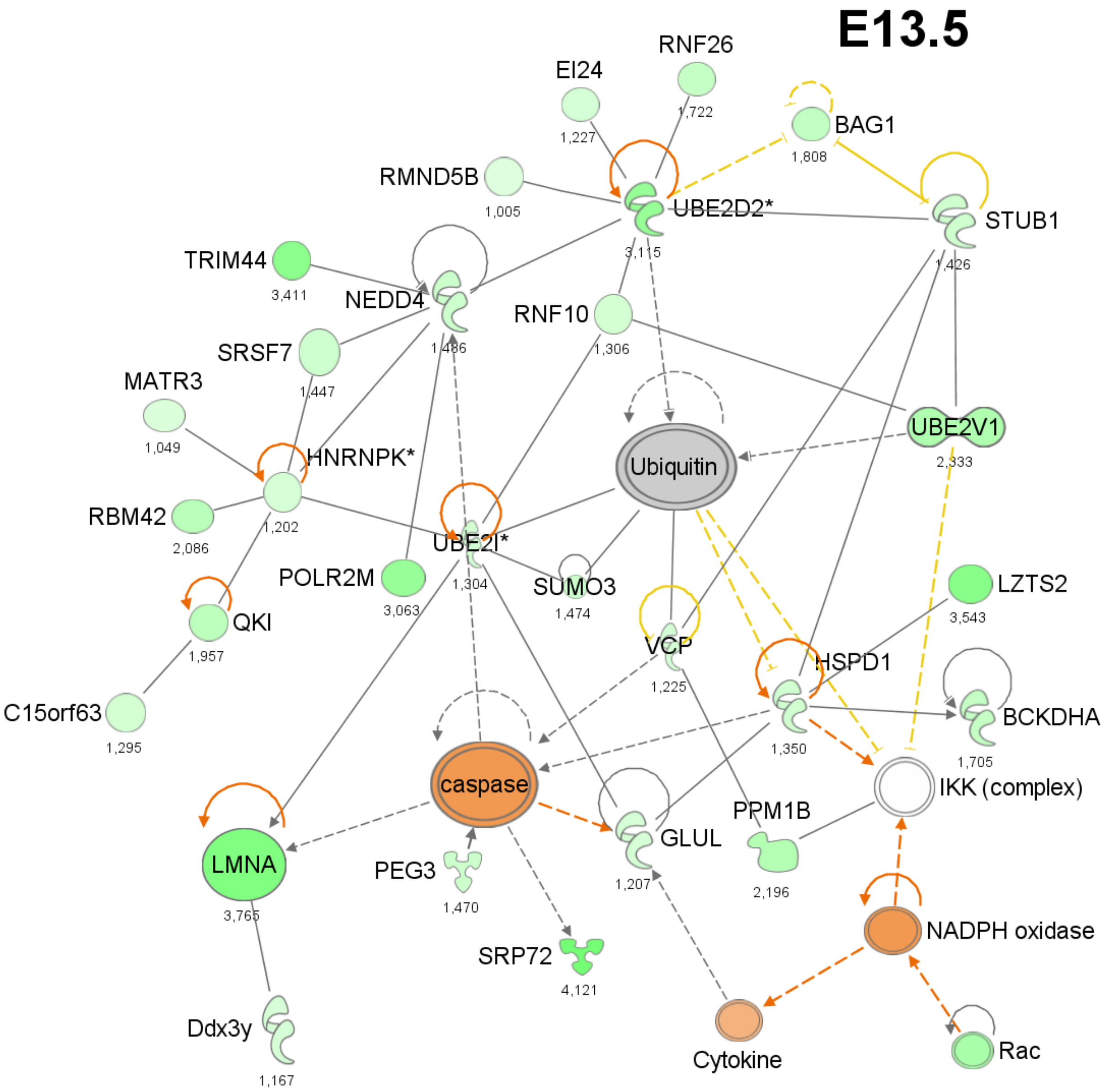

Network 5

Protein degradation, protein synthesis, post-translational modification

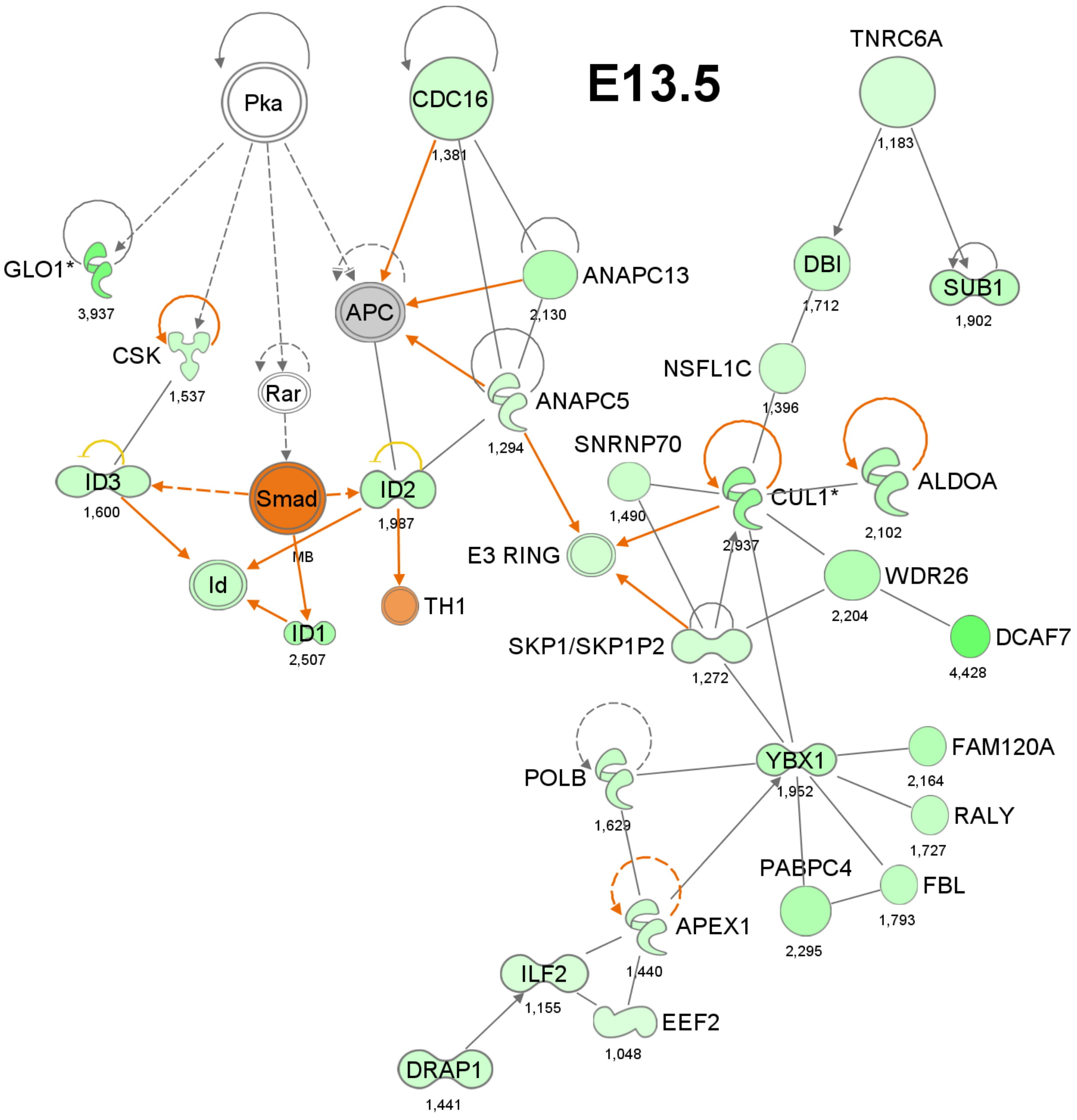

Network 6

Gene expression, cellular development, cellular growth and proliferation

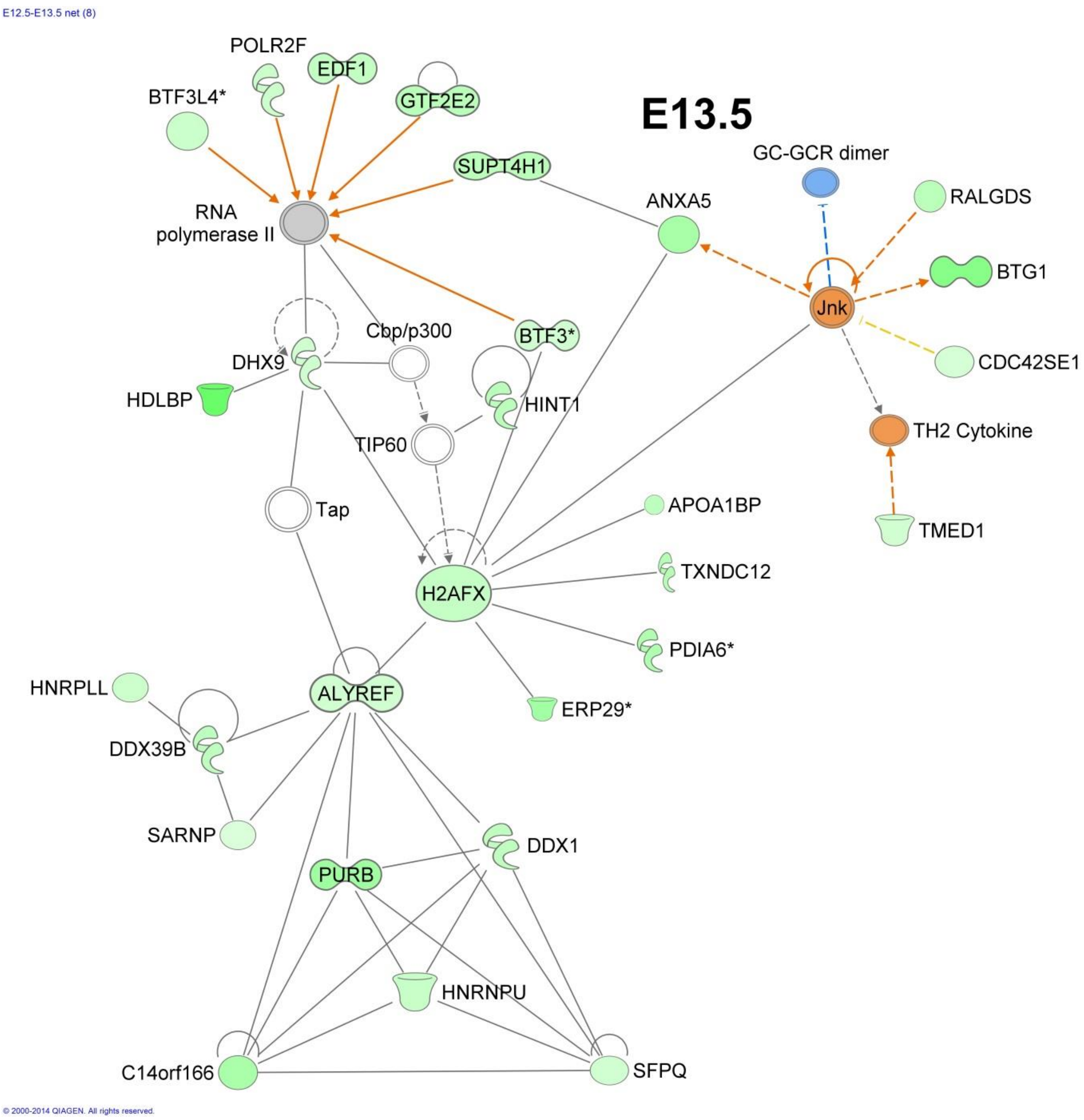

Network 7

Molecular transport, RNA trafficking, DNA replication

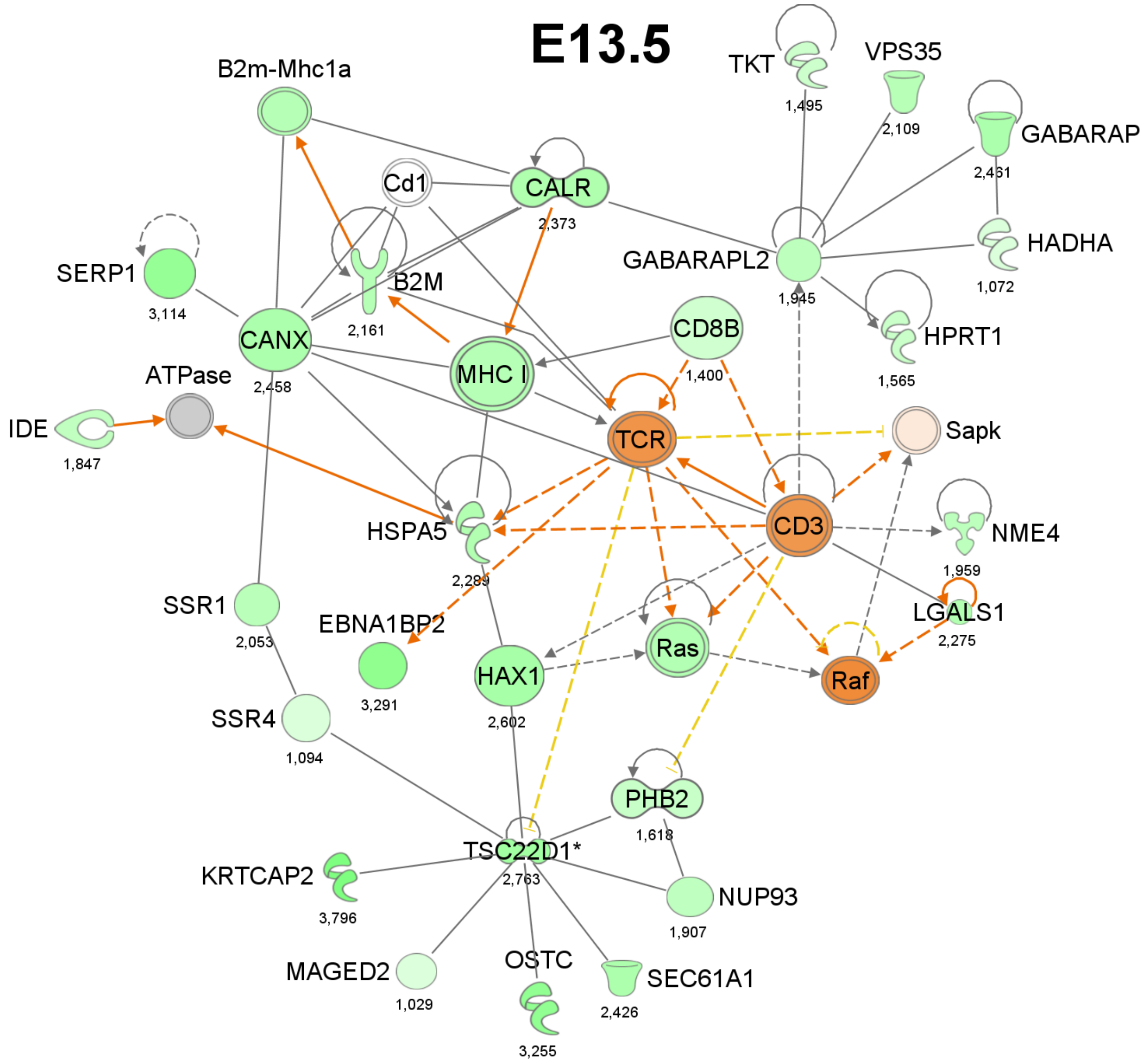

Network 8

Post-translational modification, protein folding, cellular compromise

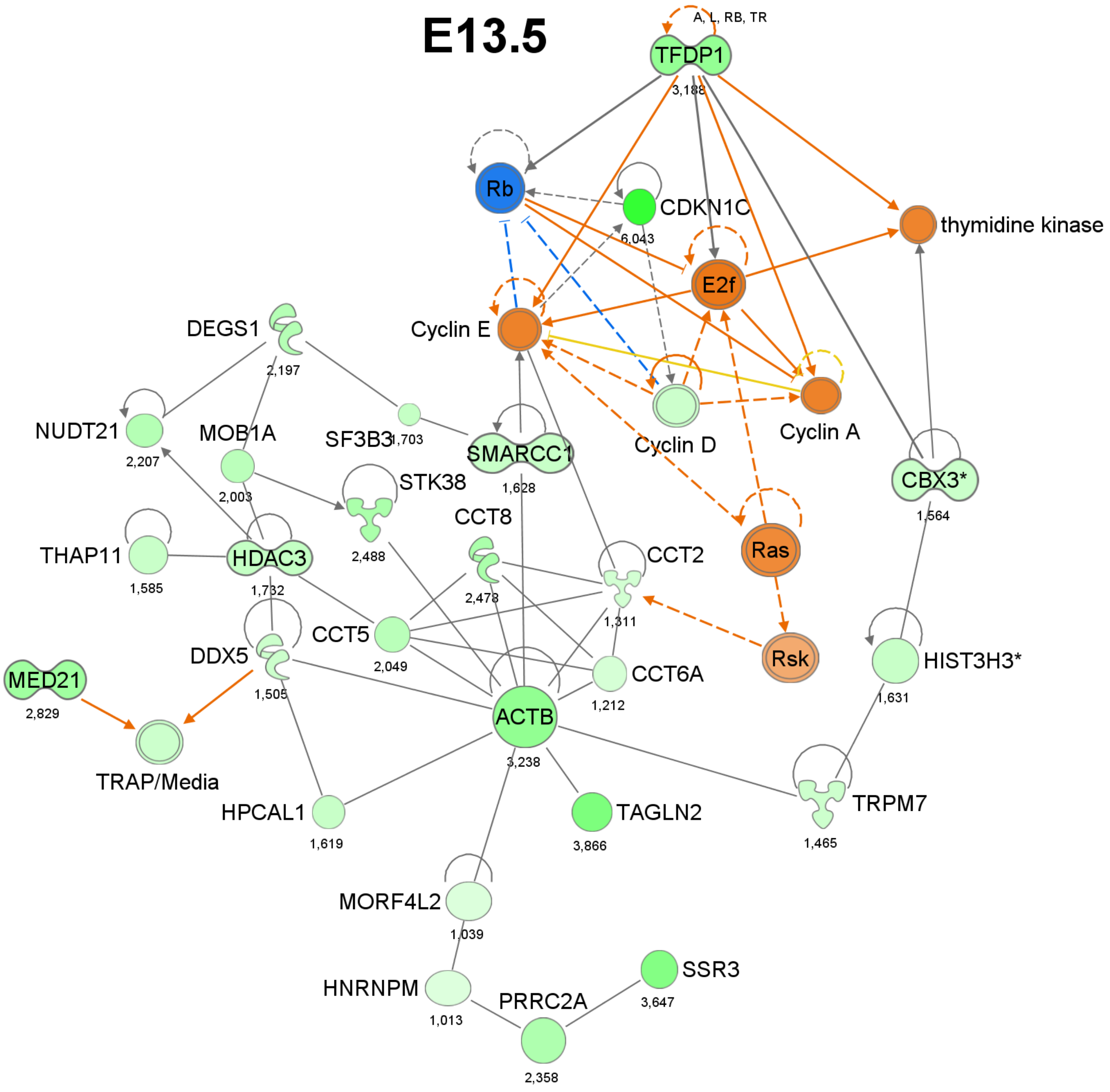

Network 9

Post-transcriptional modification, protein folding, cellular compromise

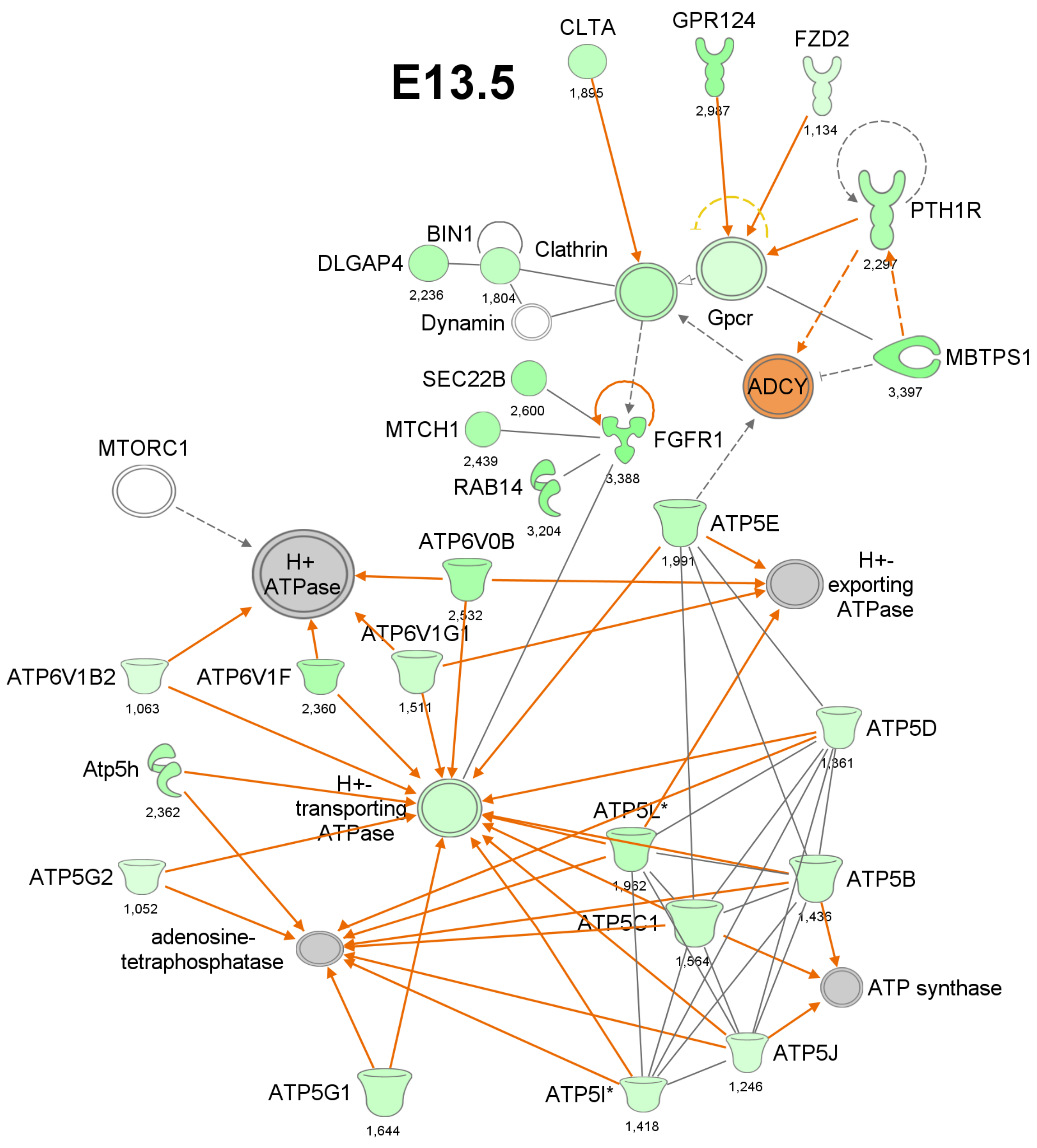

Network 10

Energy production, nucleic acid metabolism, small molecule biochemistry

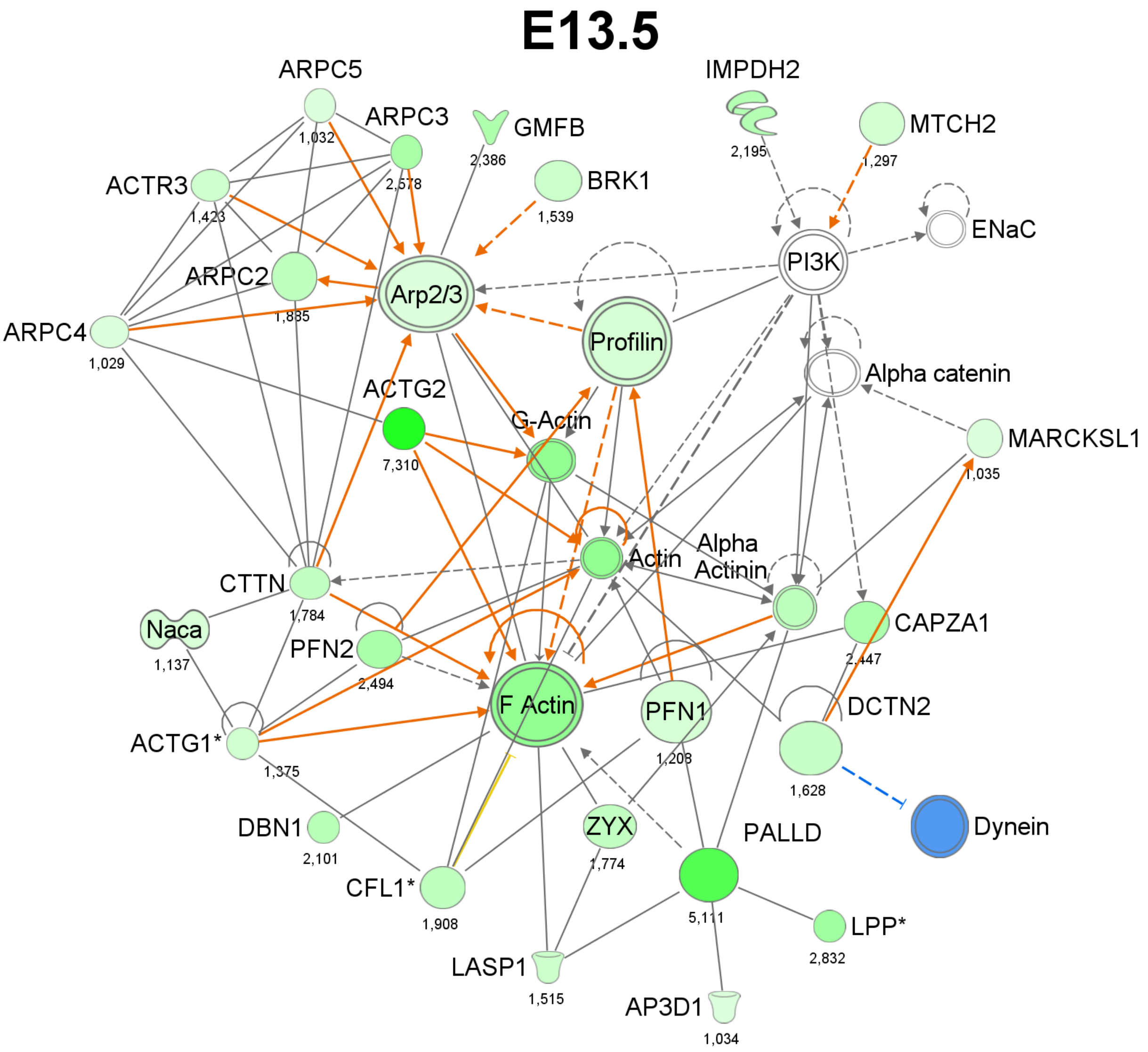

Network 11

Cellular assembly and organization, cellular function and maintenance, tissue development

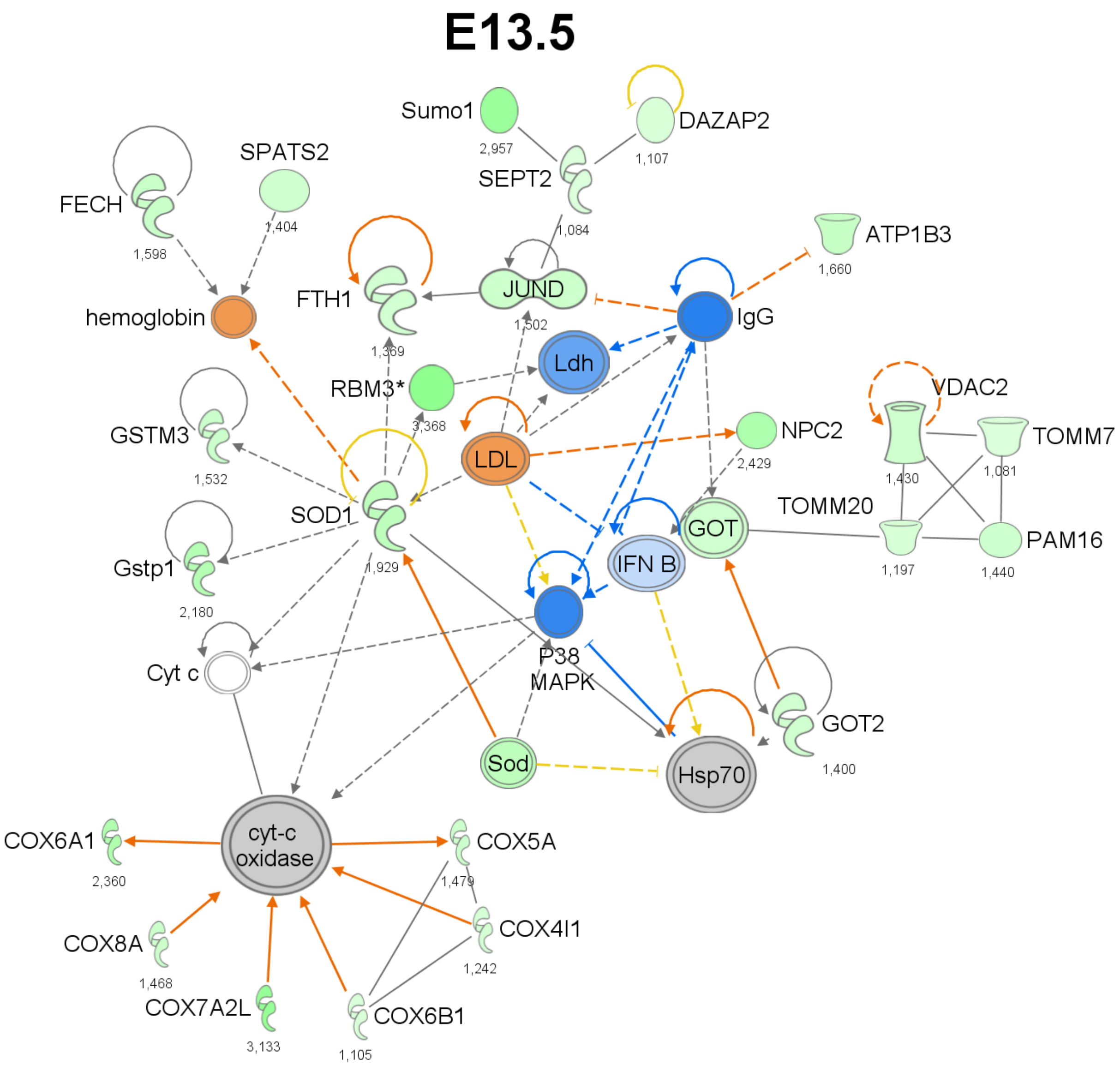

Network 12

Protein synthesis, molecular transport

E12.5-E13.5 net (13)

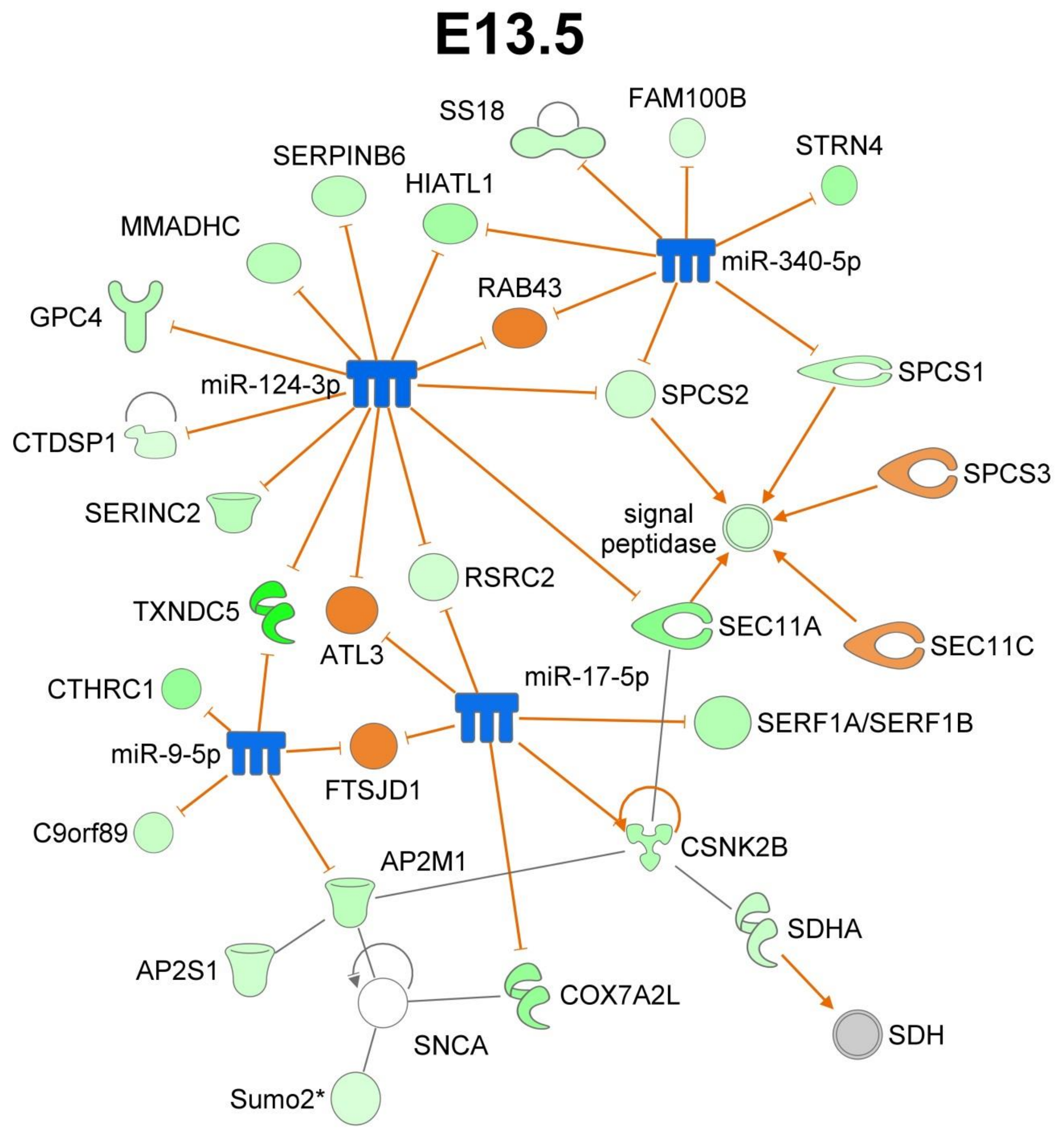

© 2000-2014 QIAGEN. All rights reserved.

Network 13

Cell death

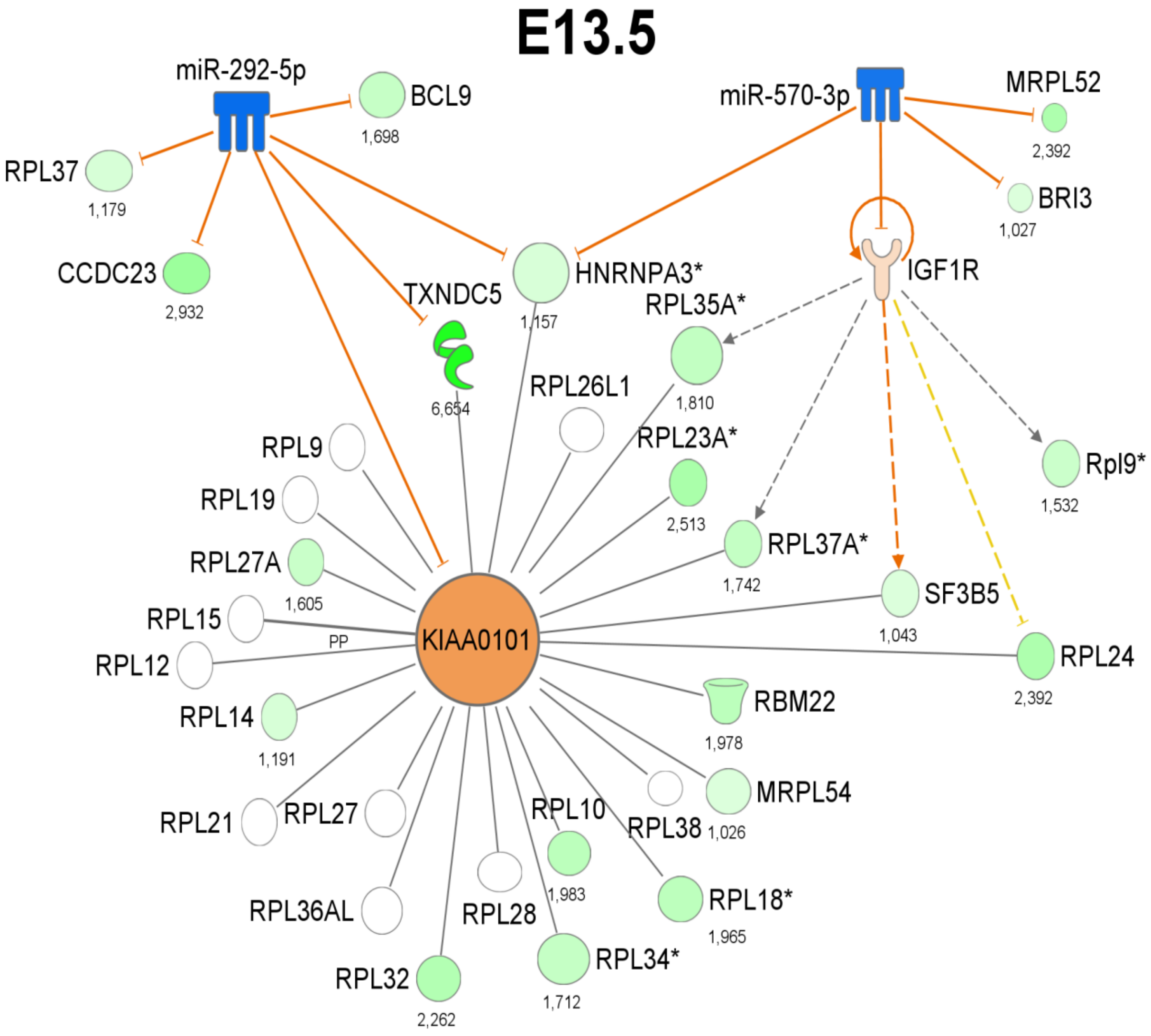

Network 14

Cell cycle, cellular development, embryonic development

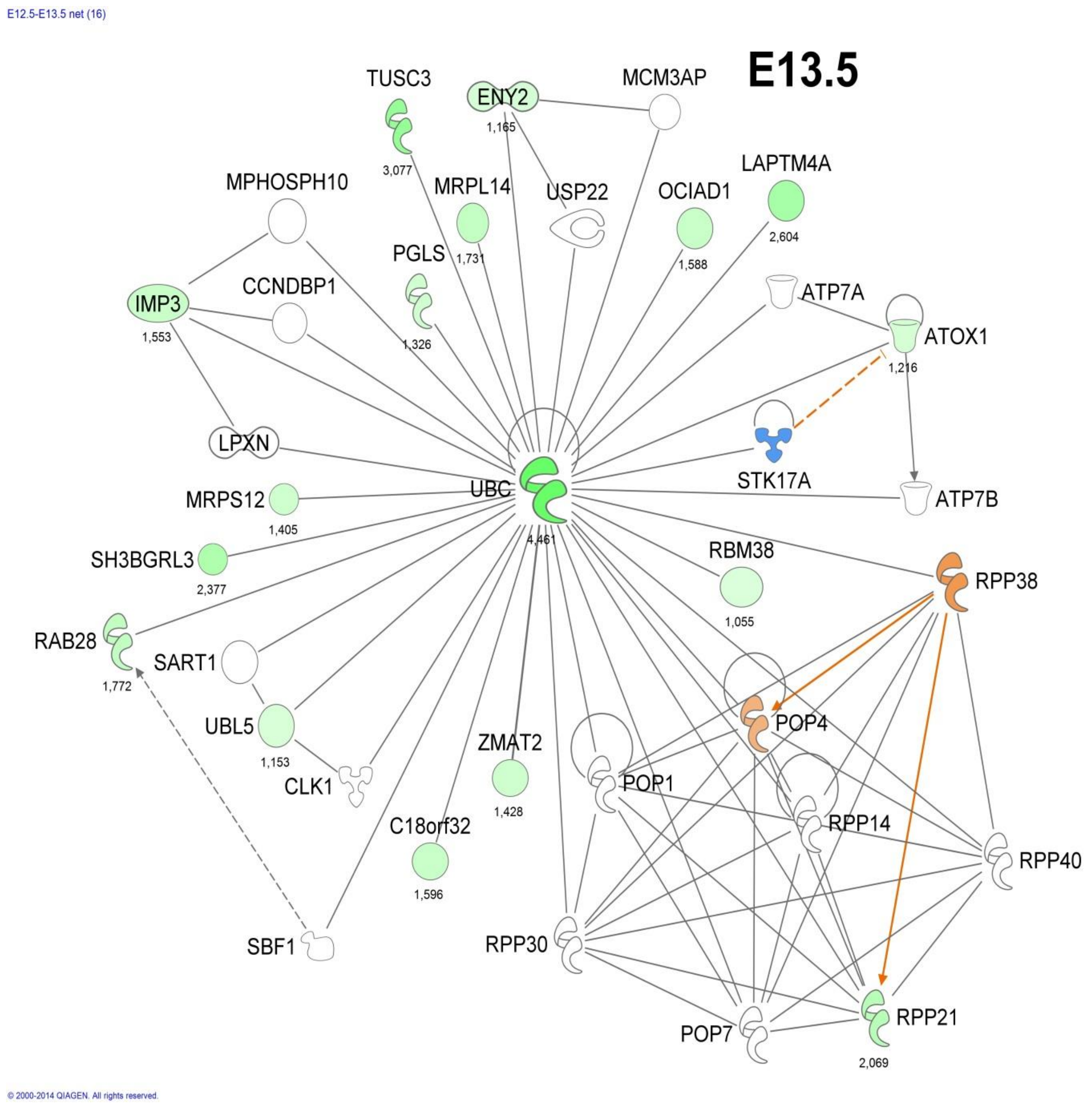

Network 15

Molecular transport, cellular function and maintenance, small molecule biochemistry

E12.5-E13.5 net (16)

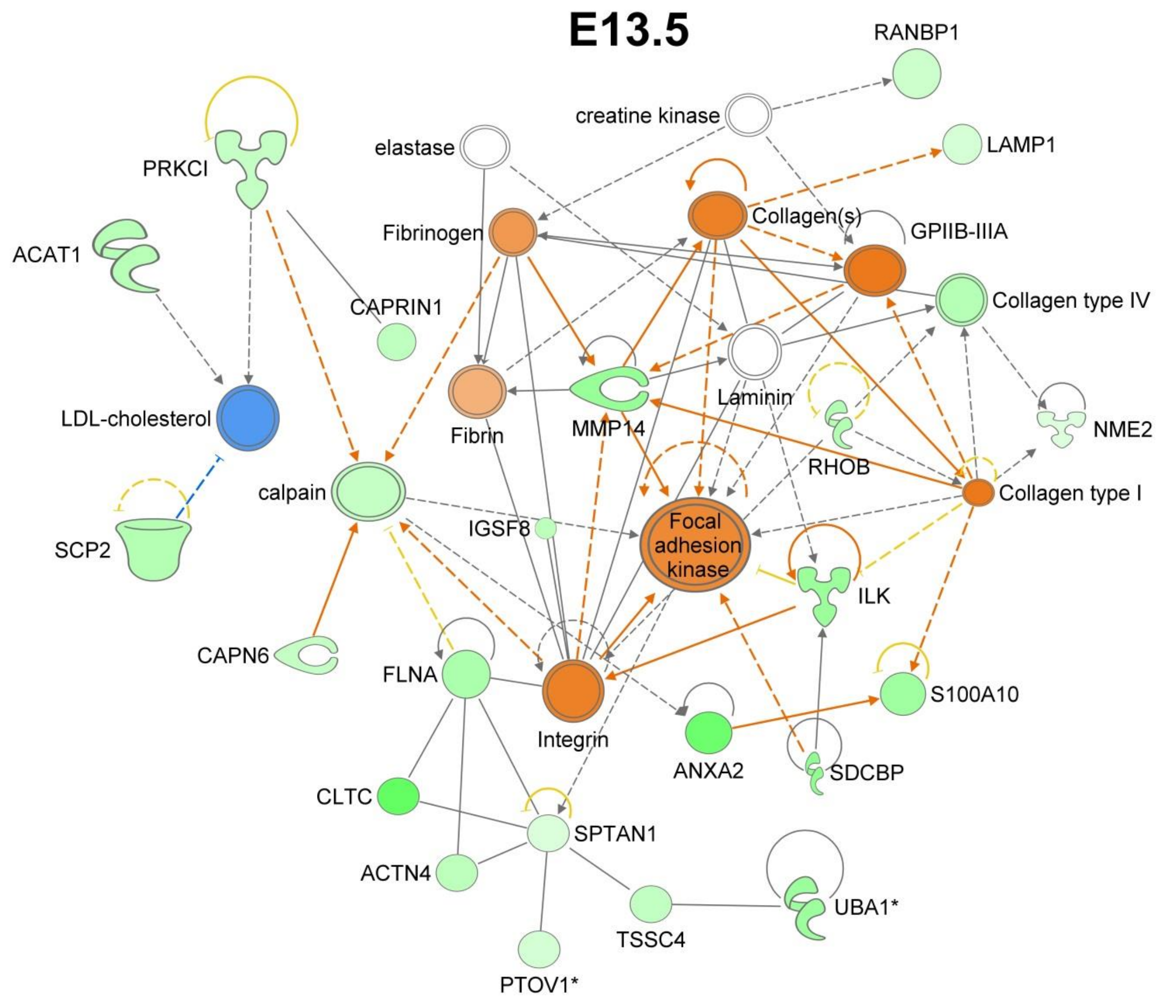

© 2000-2014 QIAGEN. All rights reserved.

Network 16

Cellular assembly and organization, cellular development, cellular growth and proliferation

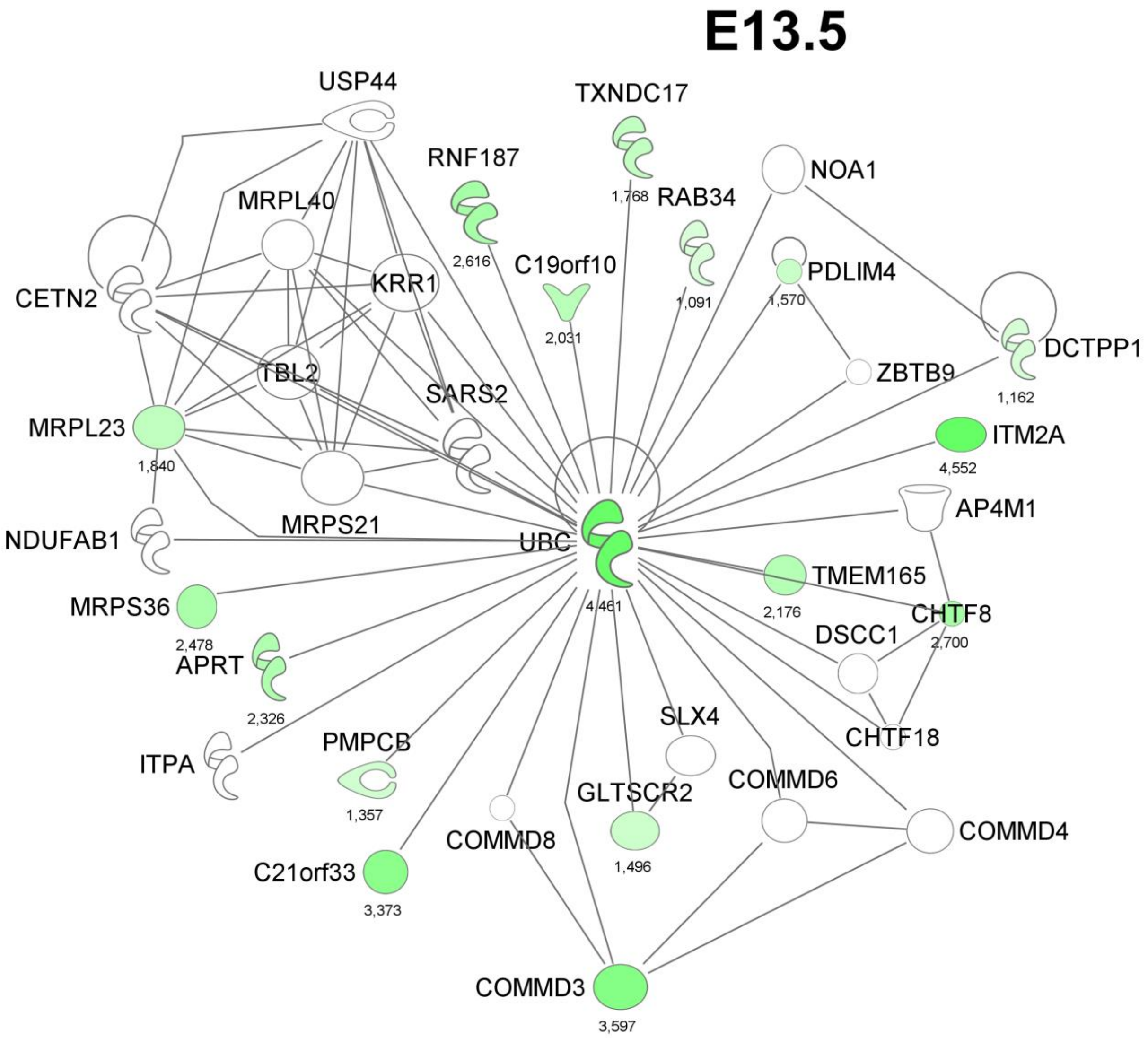

Network 17

DNA replication, nucleic acid metabolism, small molecule metabolism

Supplementary Figure 2. **Networks associated with genes expressed at enamel knot/cap stage (E14.5)**

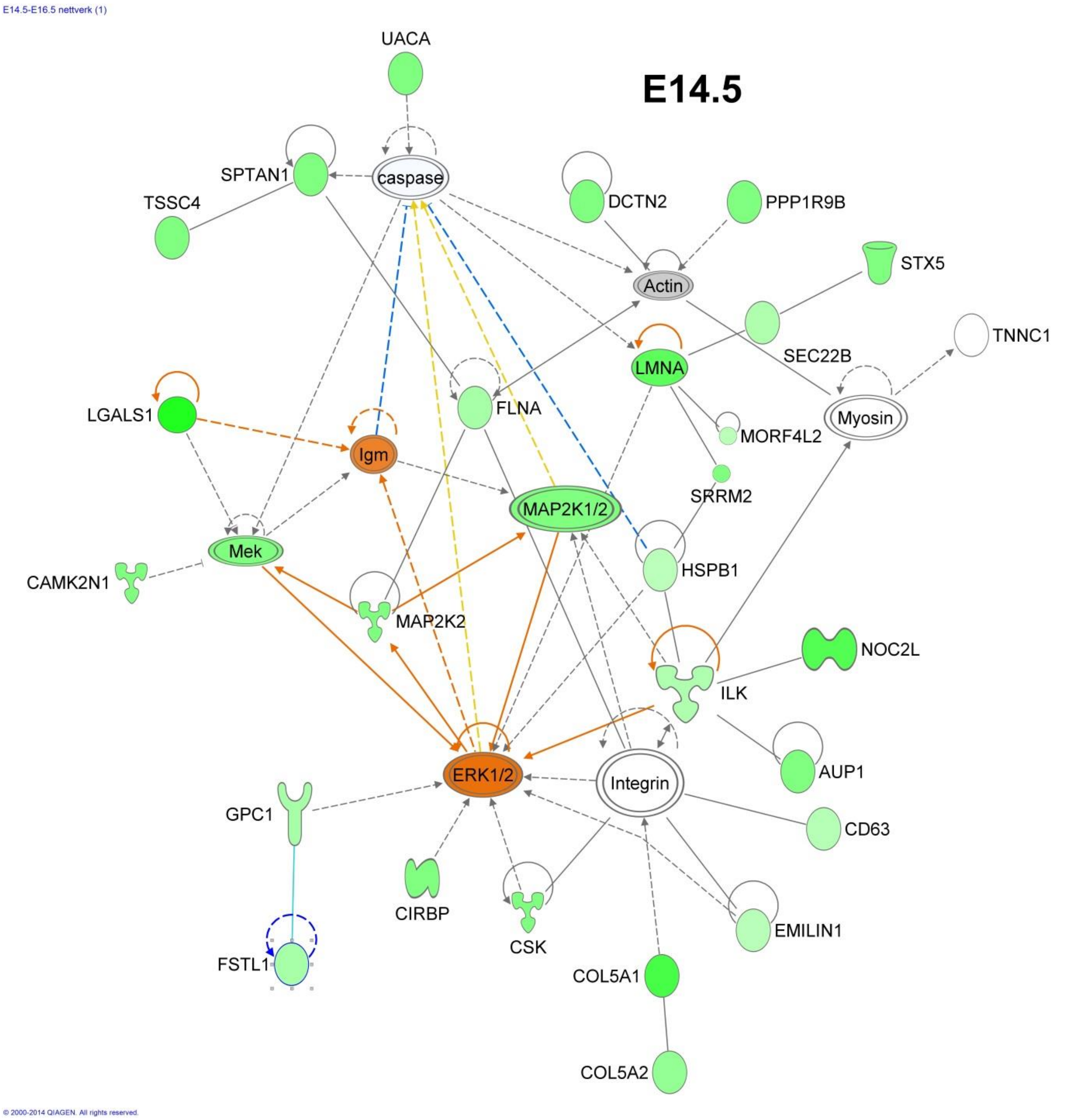

Network 1

Cellular assembly and organization, cellular function and maintenance

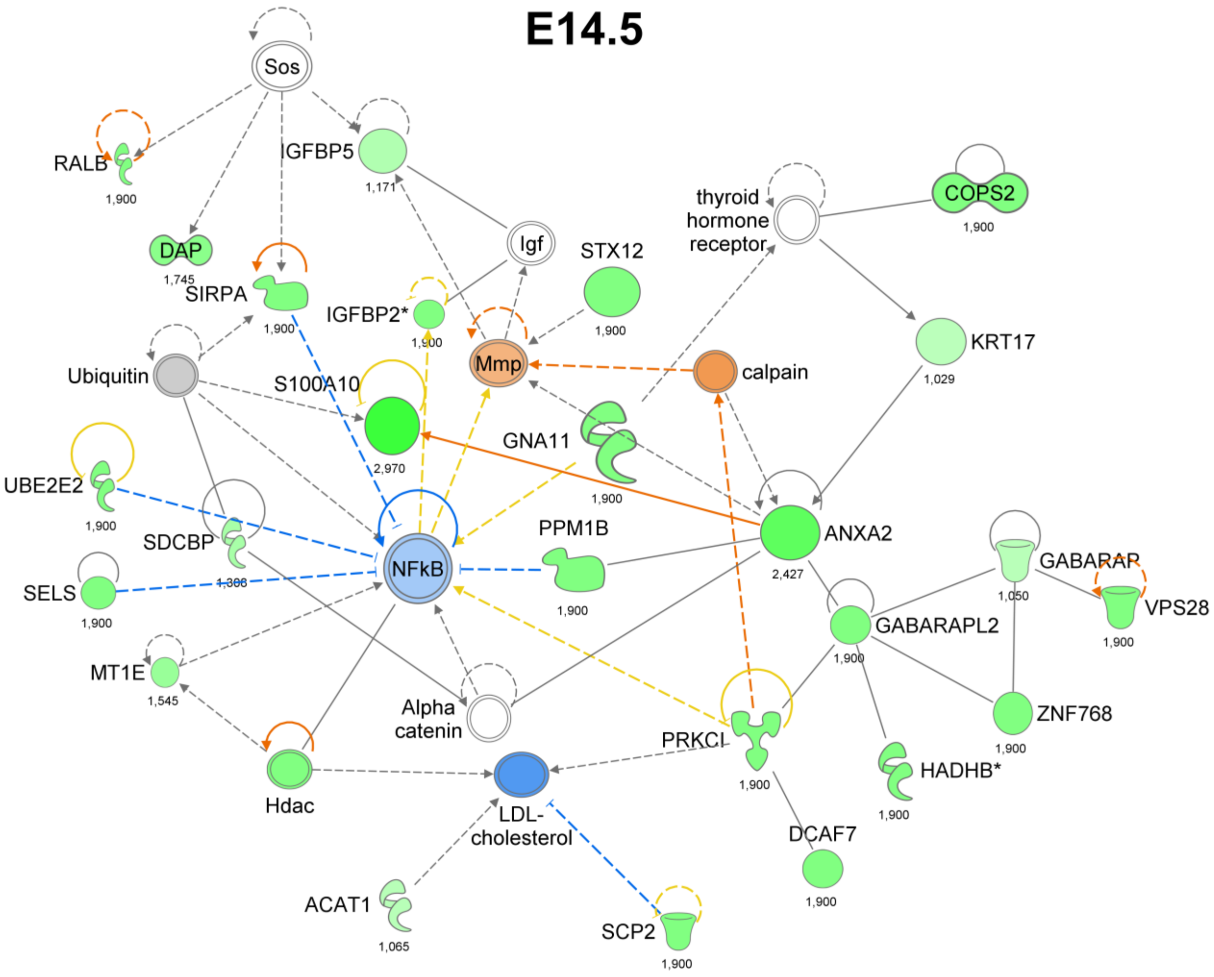

Network 2

Cellular movment, cell morphology, cellular assembly and organization

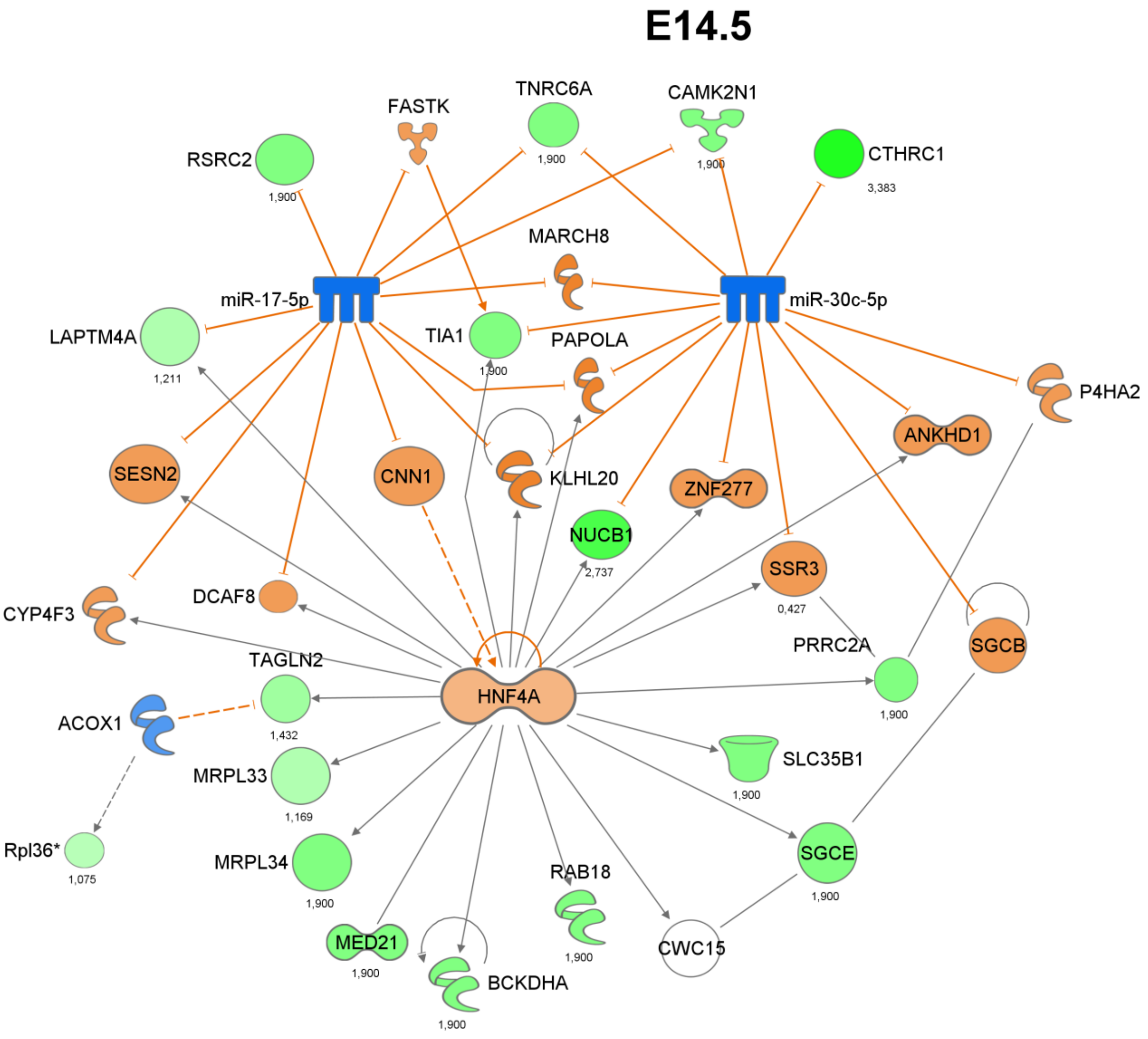

Network 3

Organismal development, cell cycle

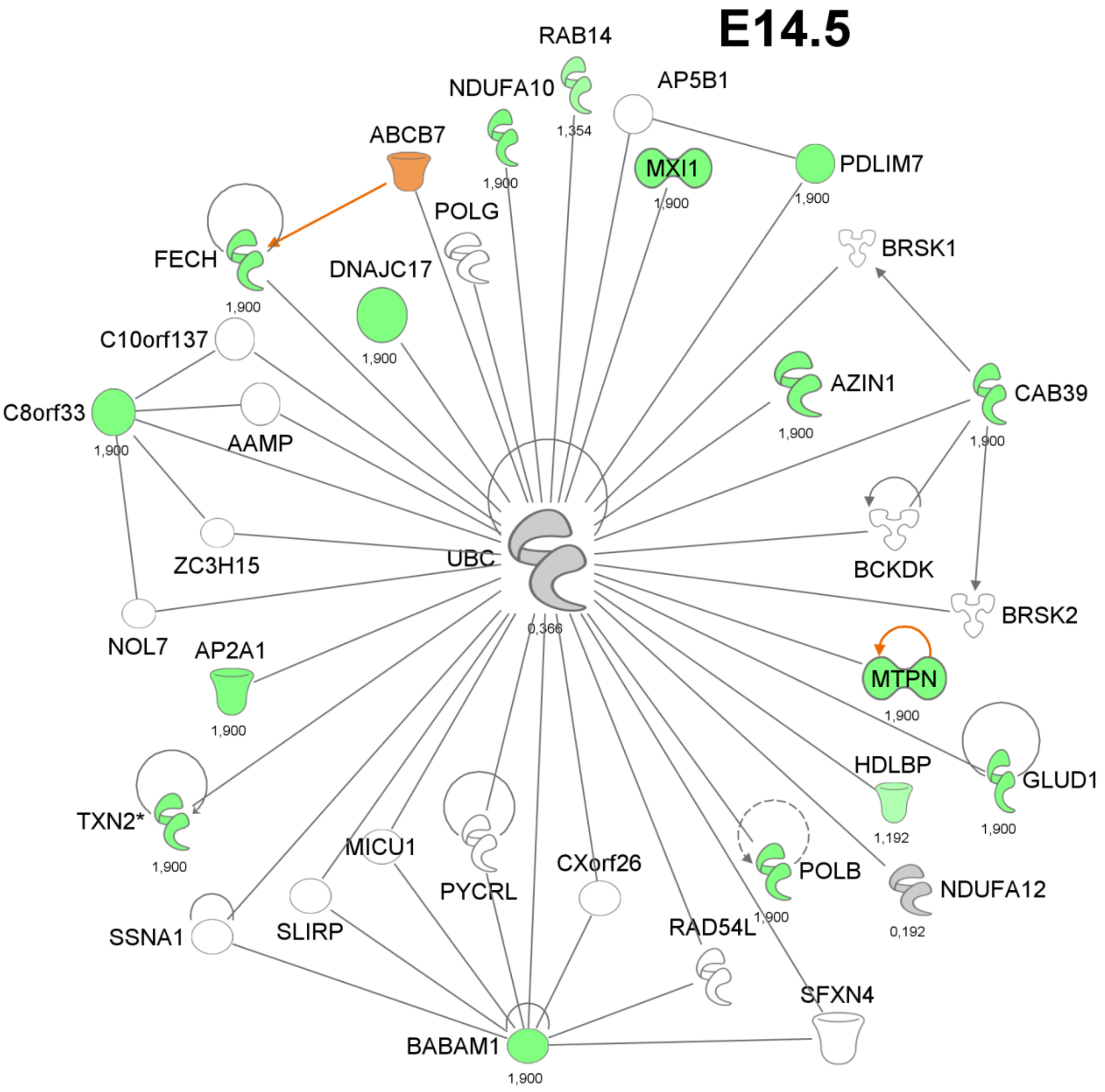

Network 4

Tissue morphology

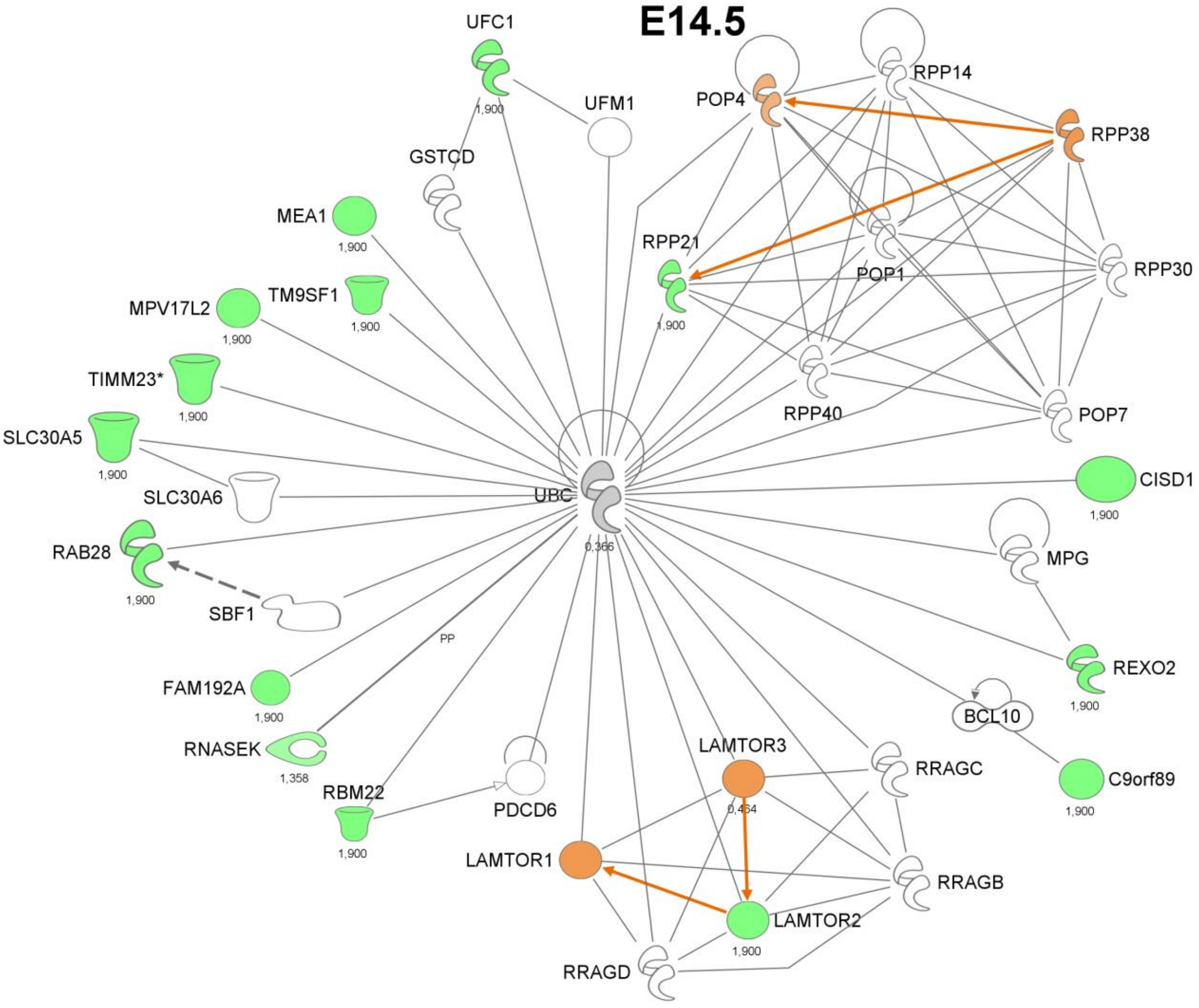

Network 5

Molecular transport

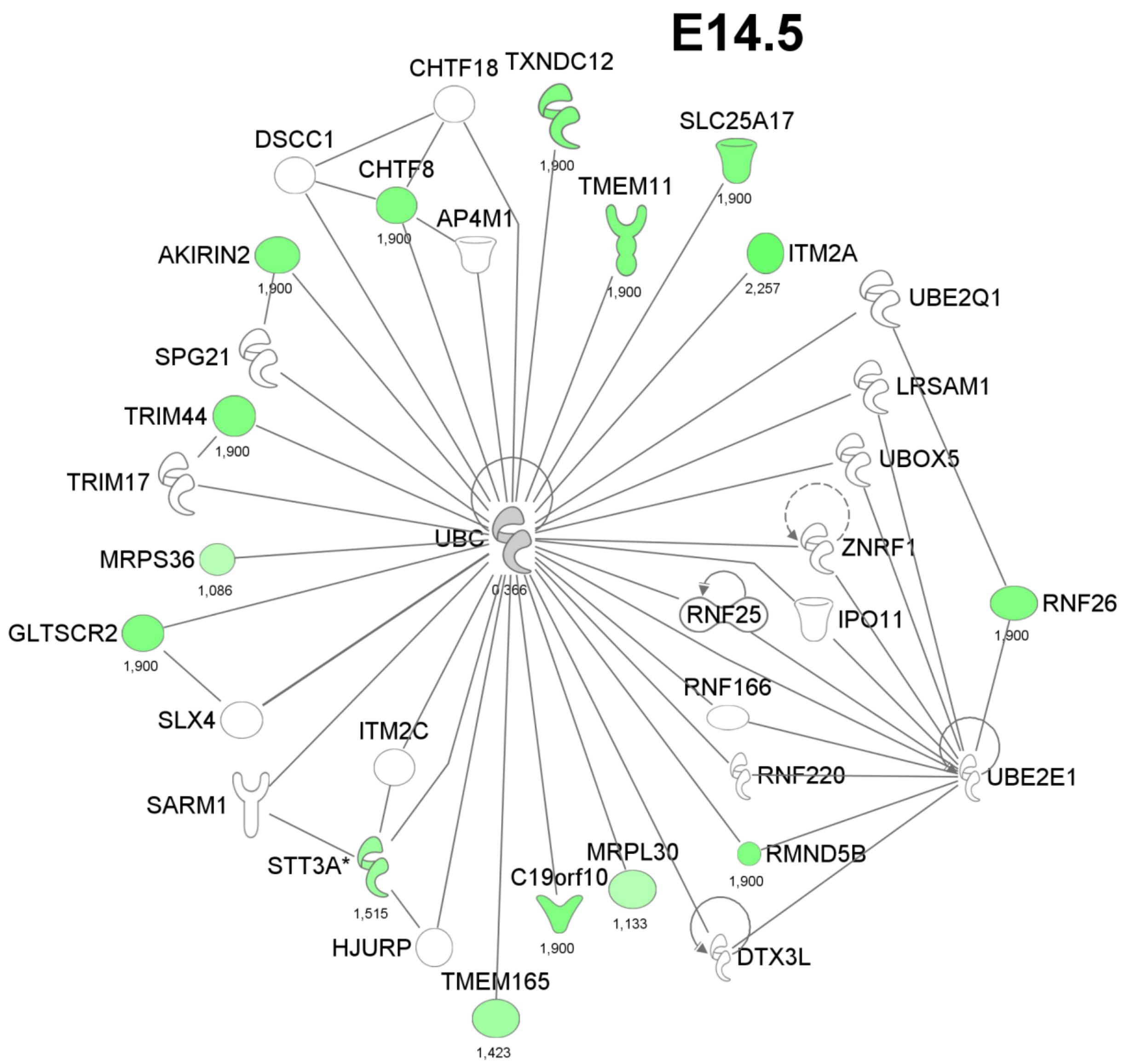

Network 6

Post-transcriptional modification, scenescence

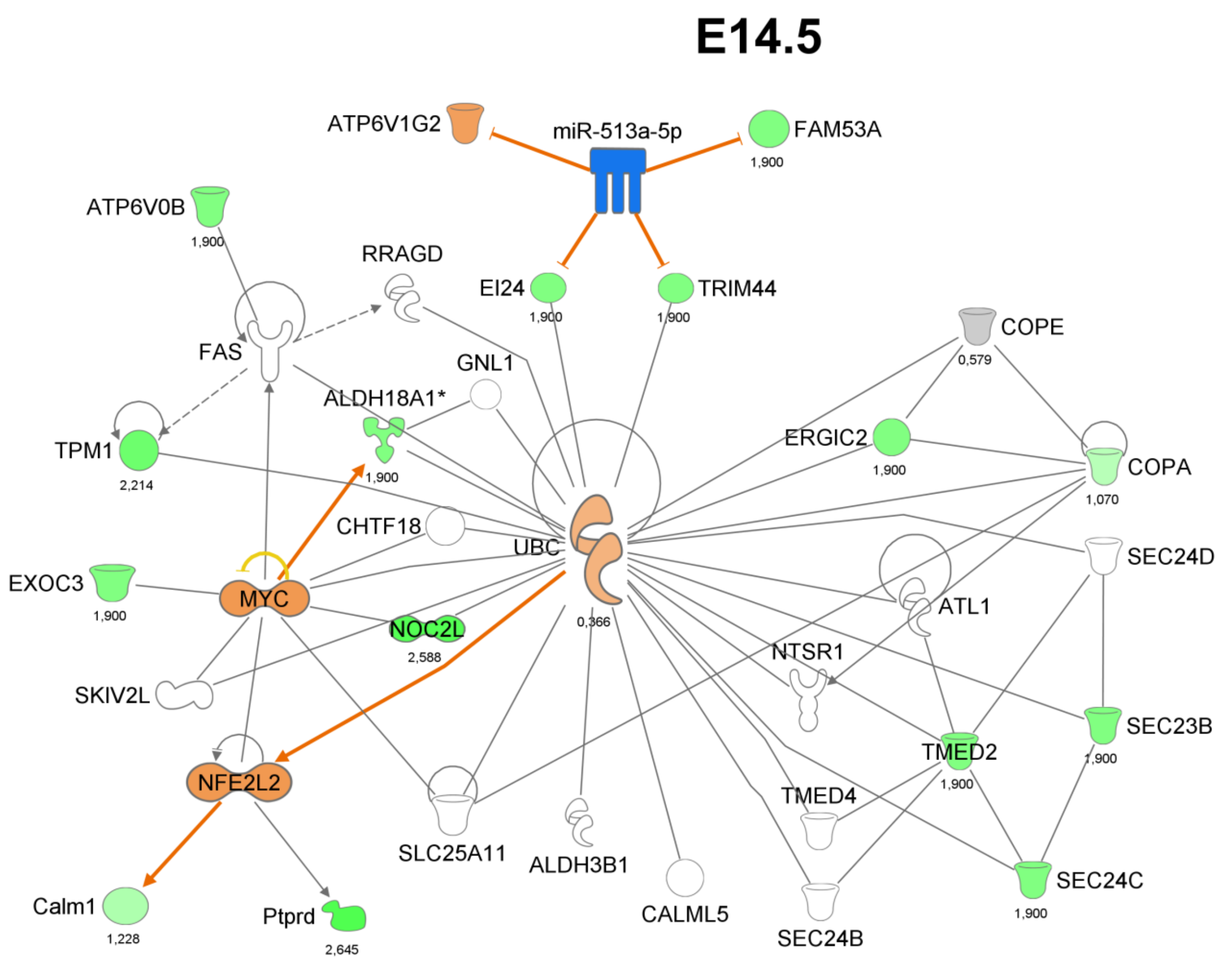

Network 7

Cell morphology, post-transcriptional modification

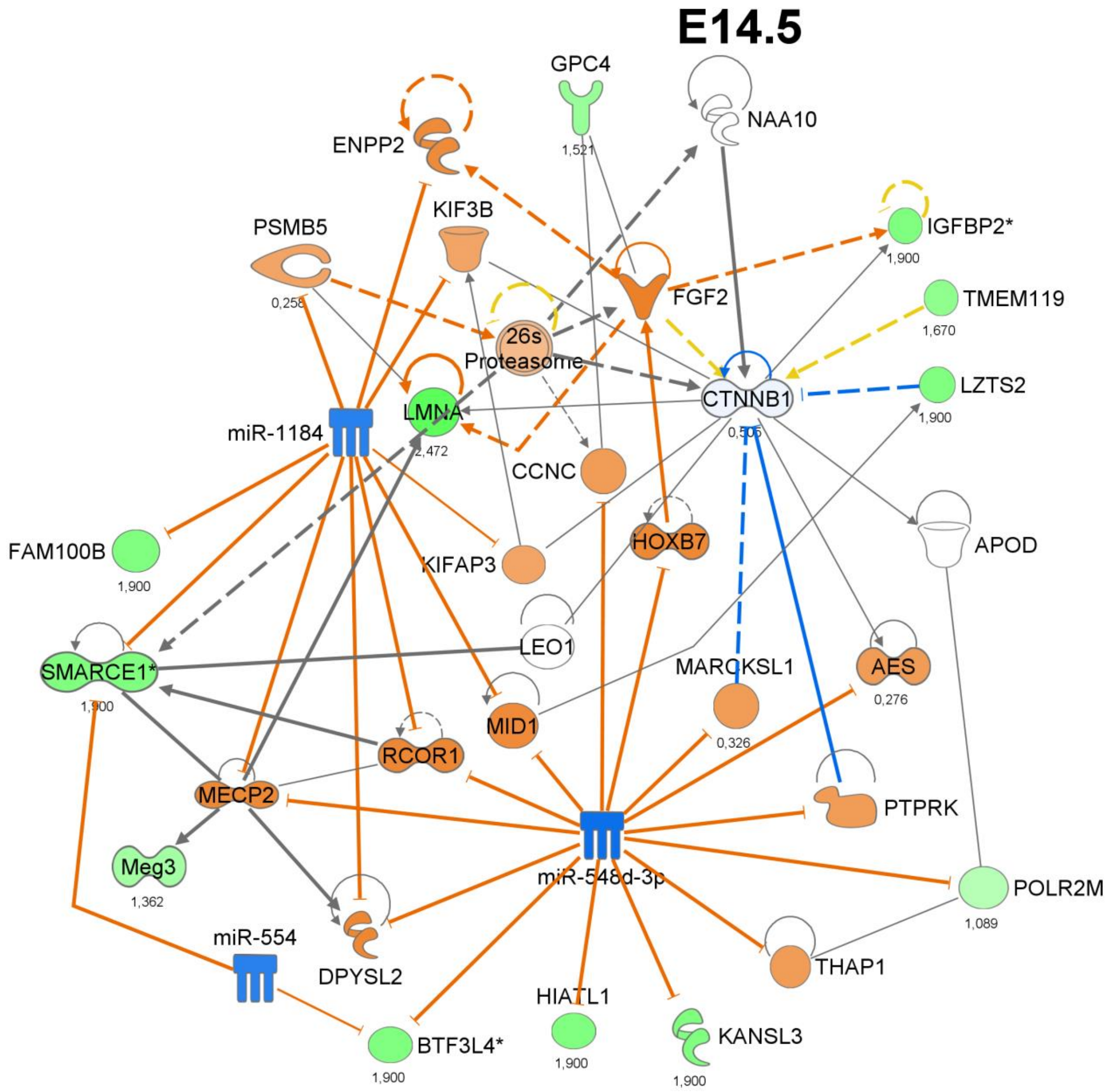

Network 8

Cellular development, cellular growth and proliferation

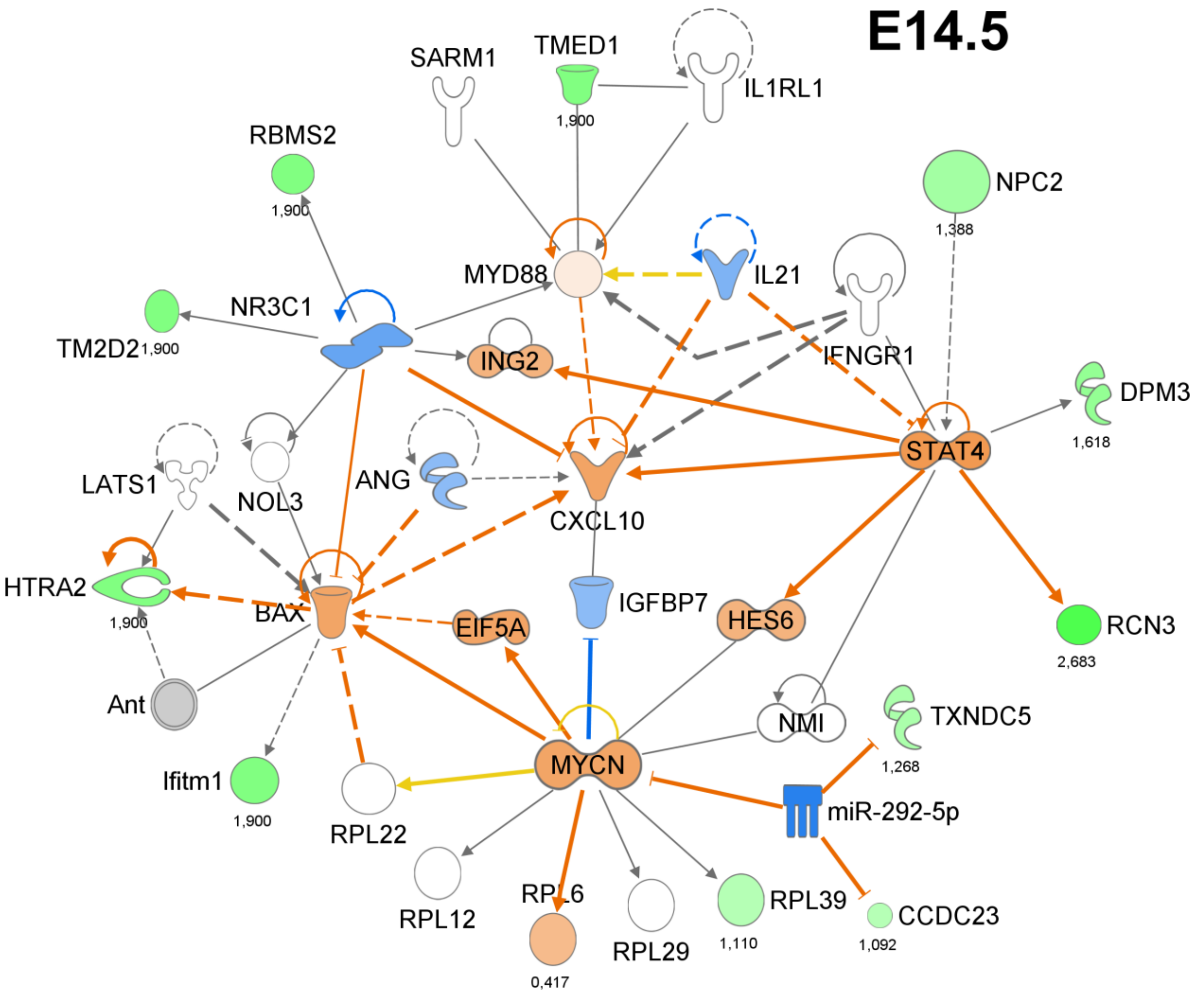

Network 9

Cell-to-cell signaling and interaction

Supplementary Figure 3. **Networks associated with genes expressed at bell stages (E15.5-E18.5)**

UBC E15.5

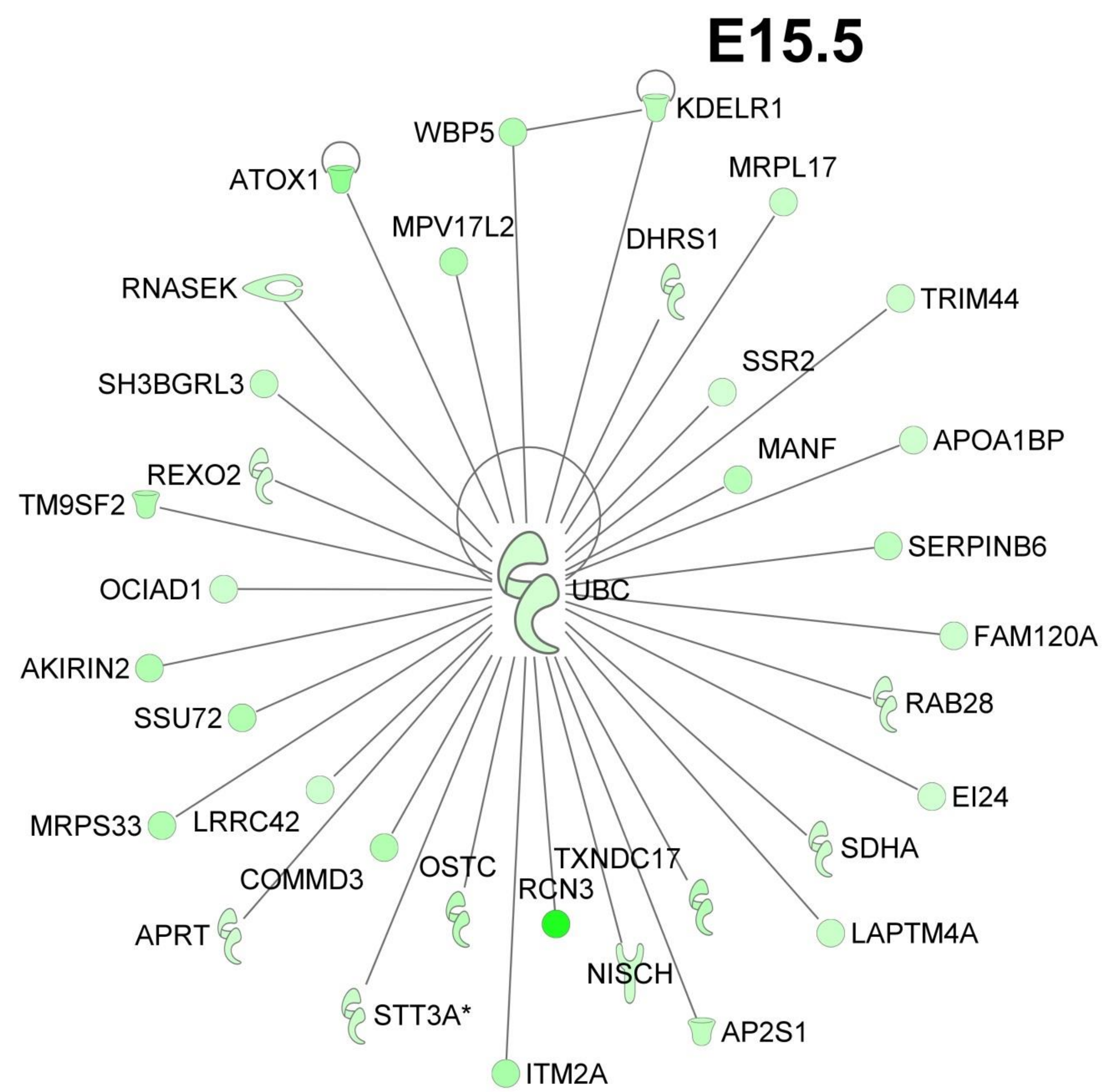

© 2000-2014 QIAGEN. All rights reserved.

Network 1

Cellular movment

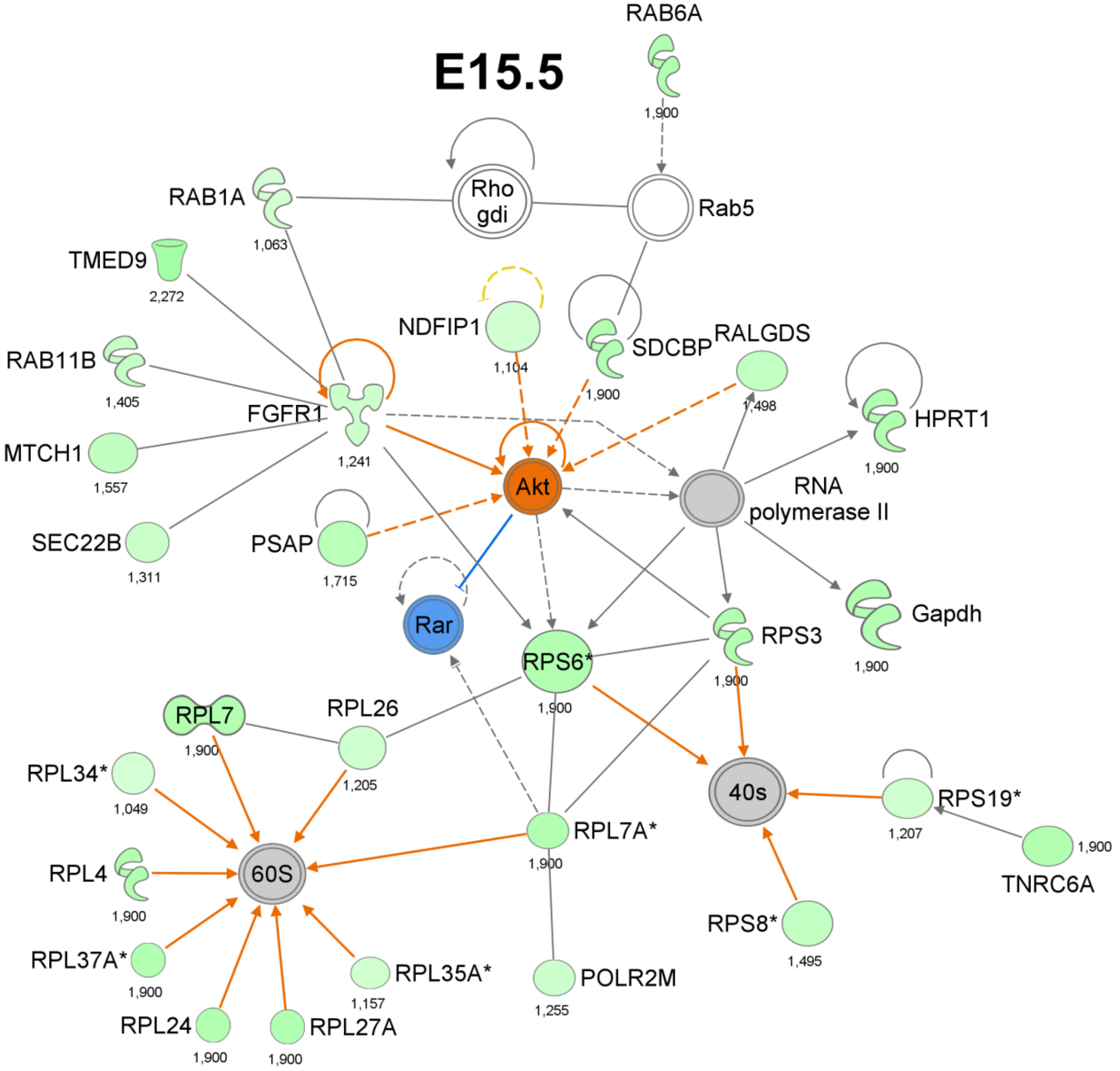

Network 2

RNA post-transcriptional modification

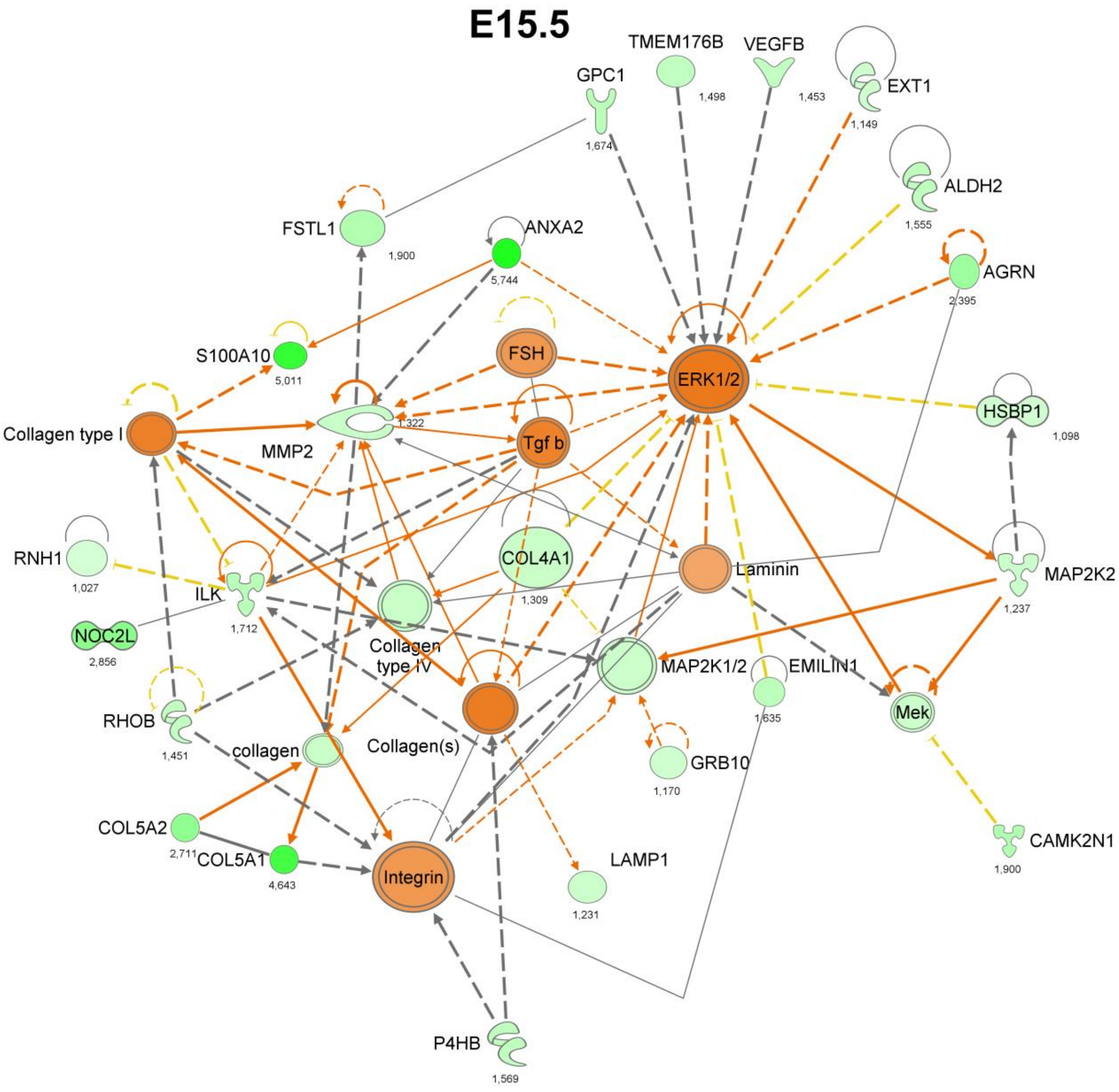

Network 3

Cellular assembly and organization, cellular function and maitenance

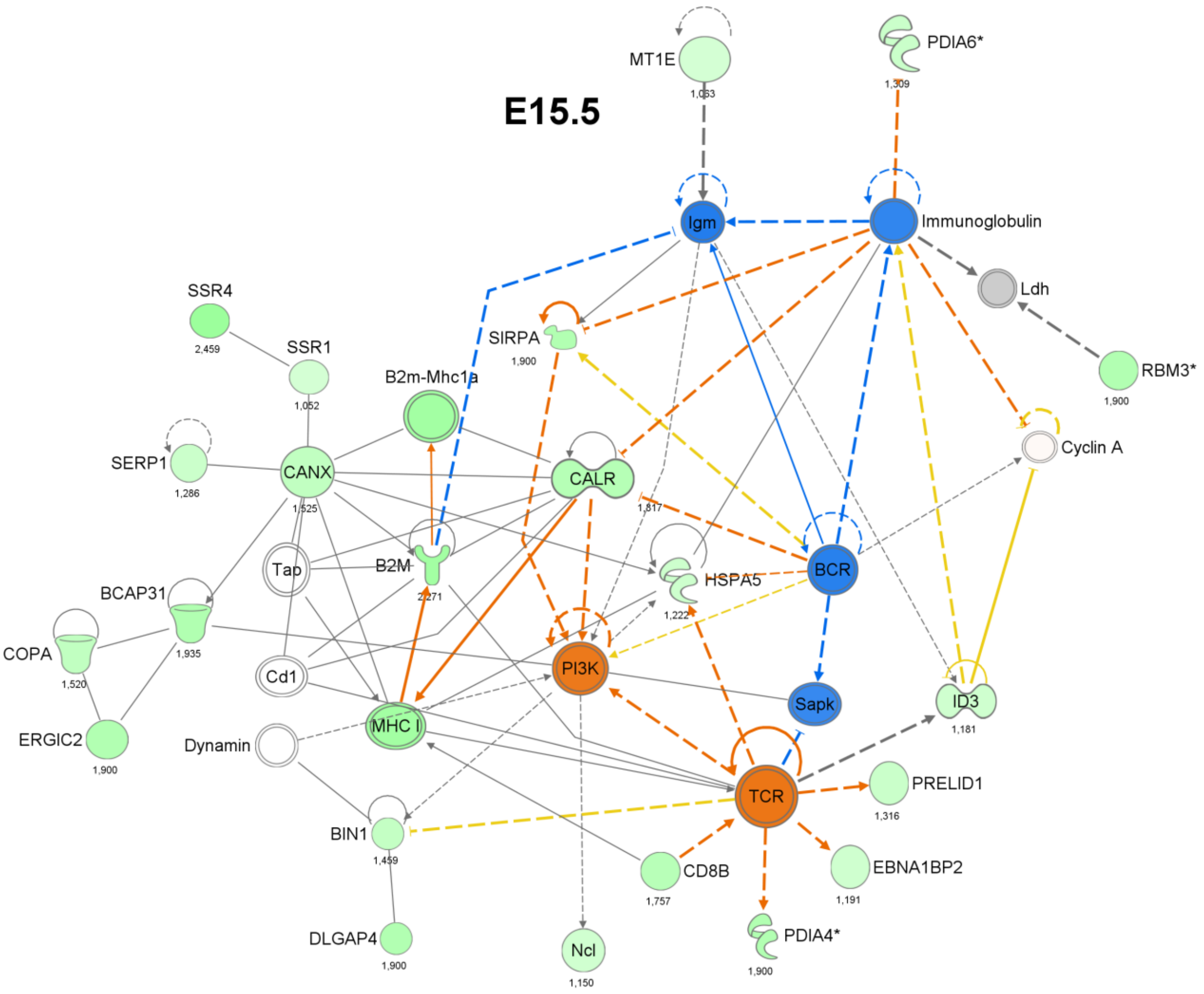

Network 4

Post- transcriptional modification, protein folding

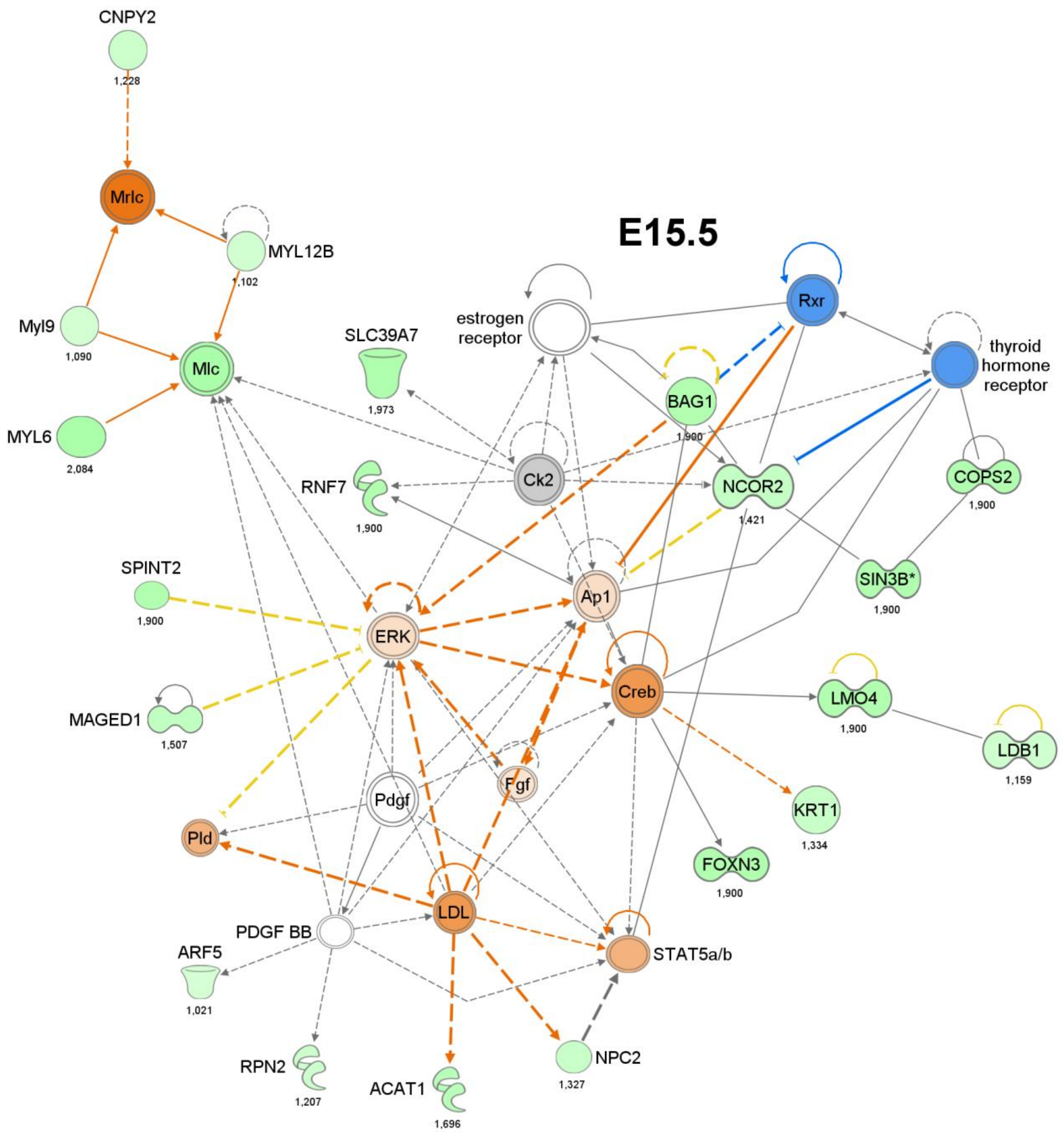

Network 5

Cellular development

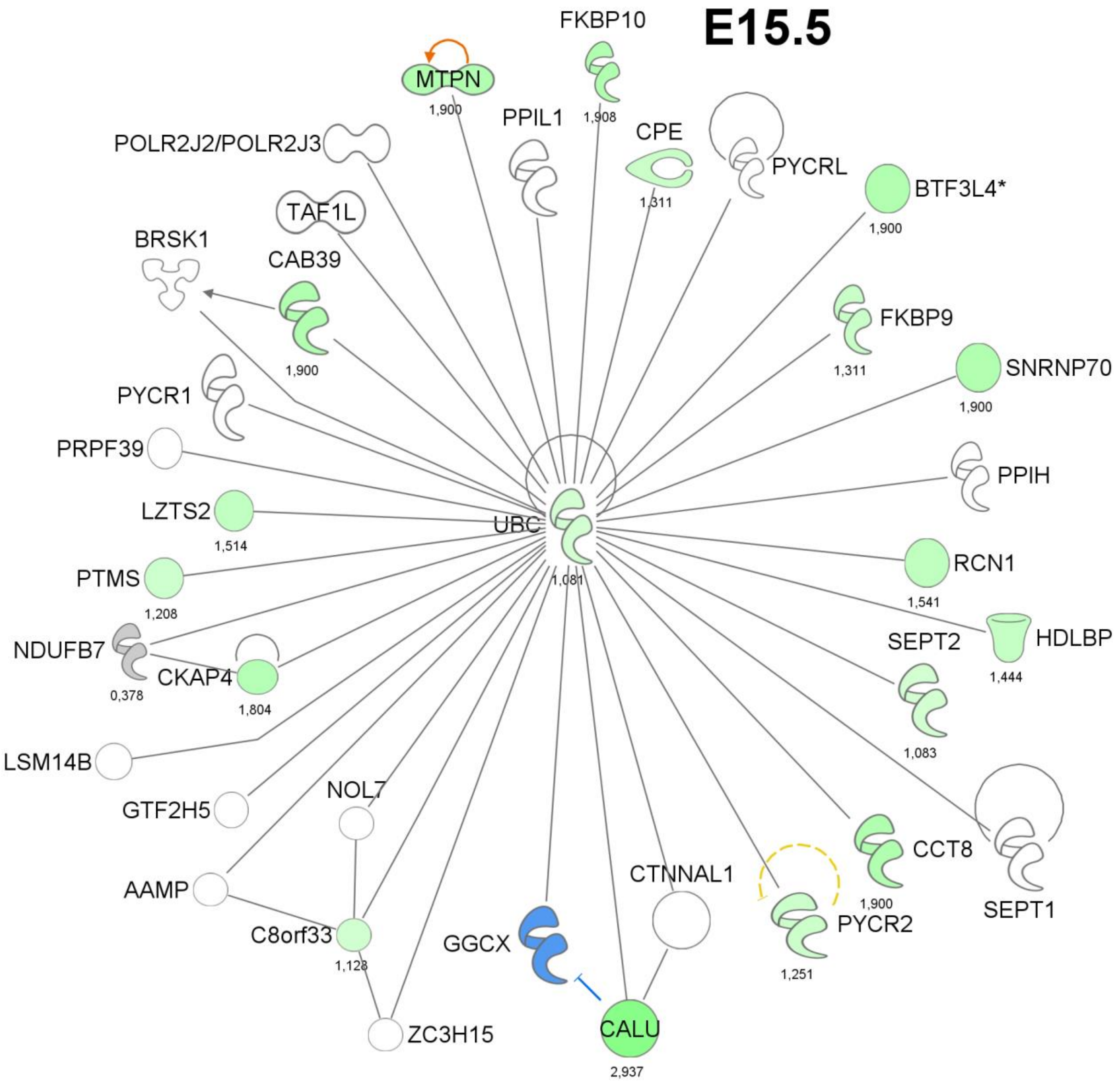

Network 6

Cell morphology, cellular assembly and organization, cellular compromise

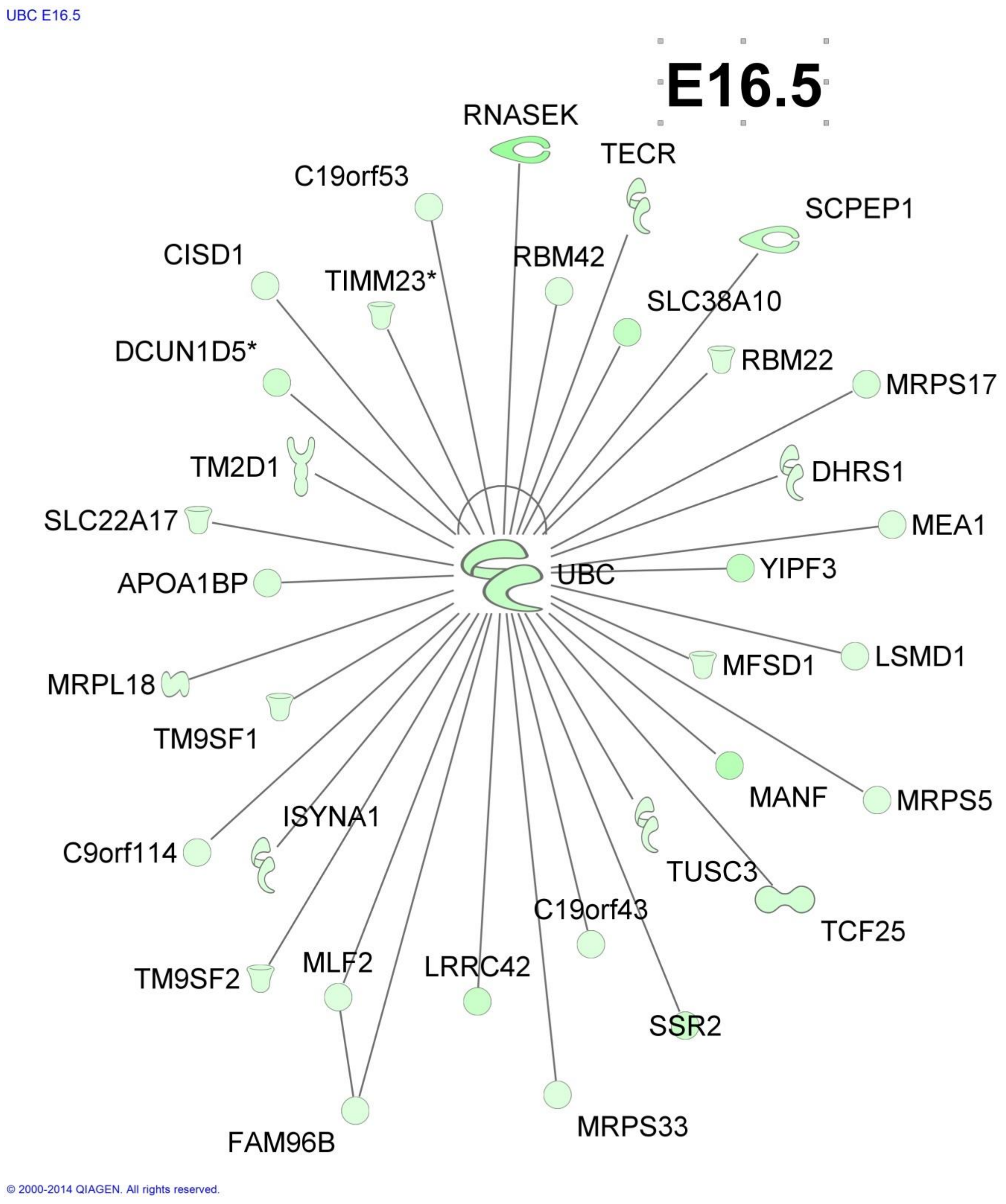

Network 1

Lipid metabolism, nucleic acid metabolism, small molecule biochemistry, scenescence

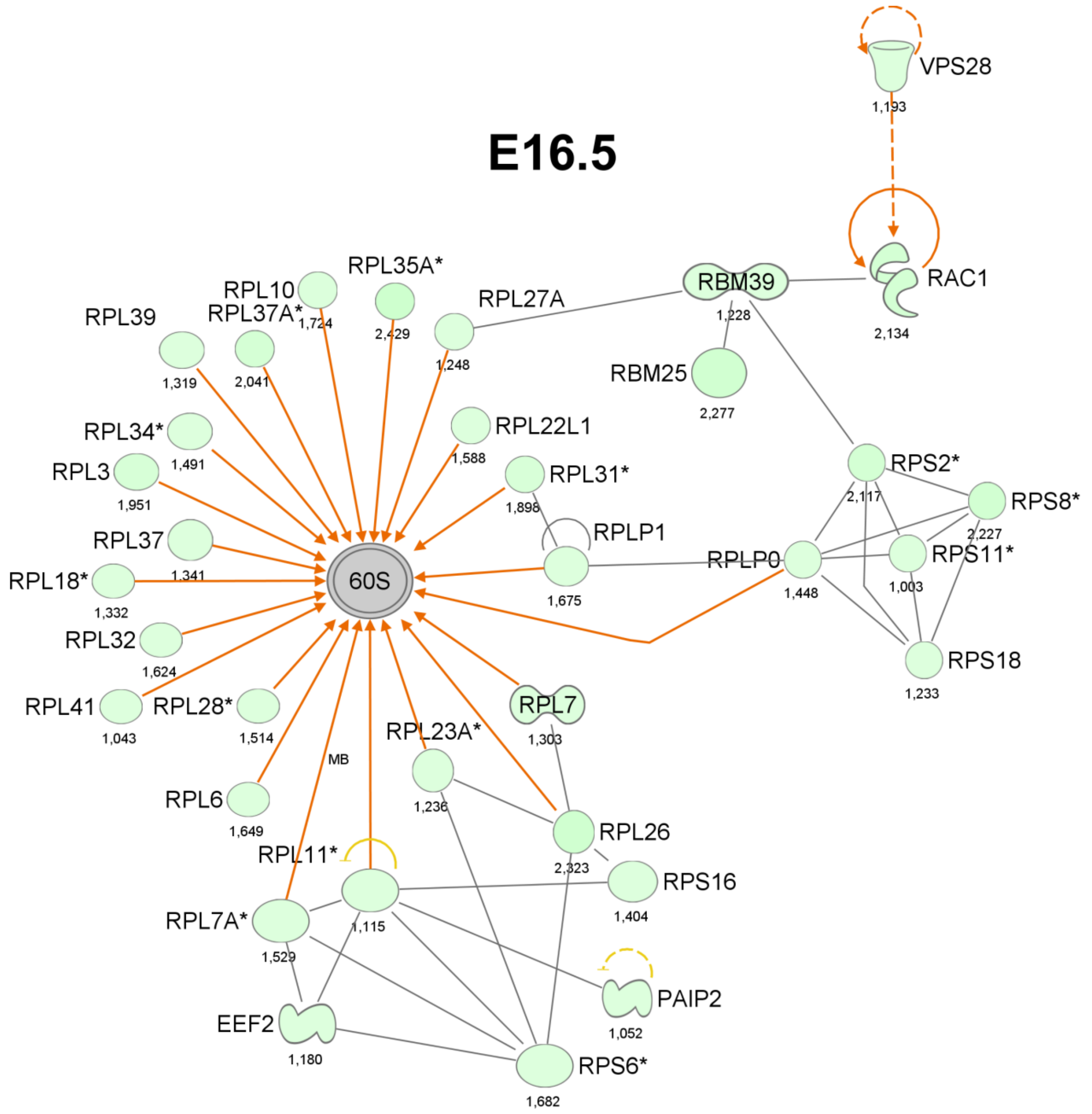

Network 2

RNA post-transcriptional modification

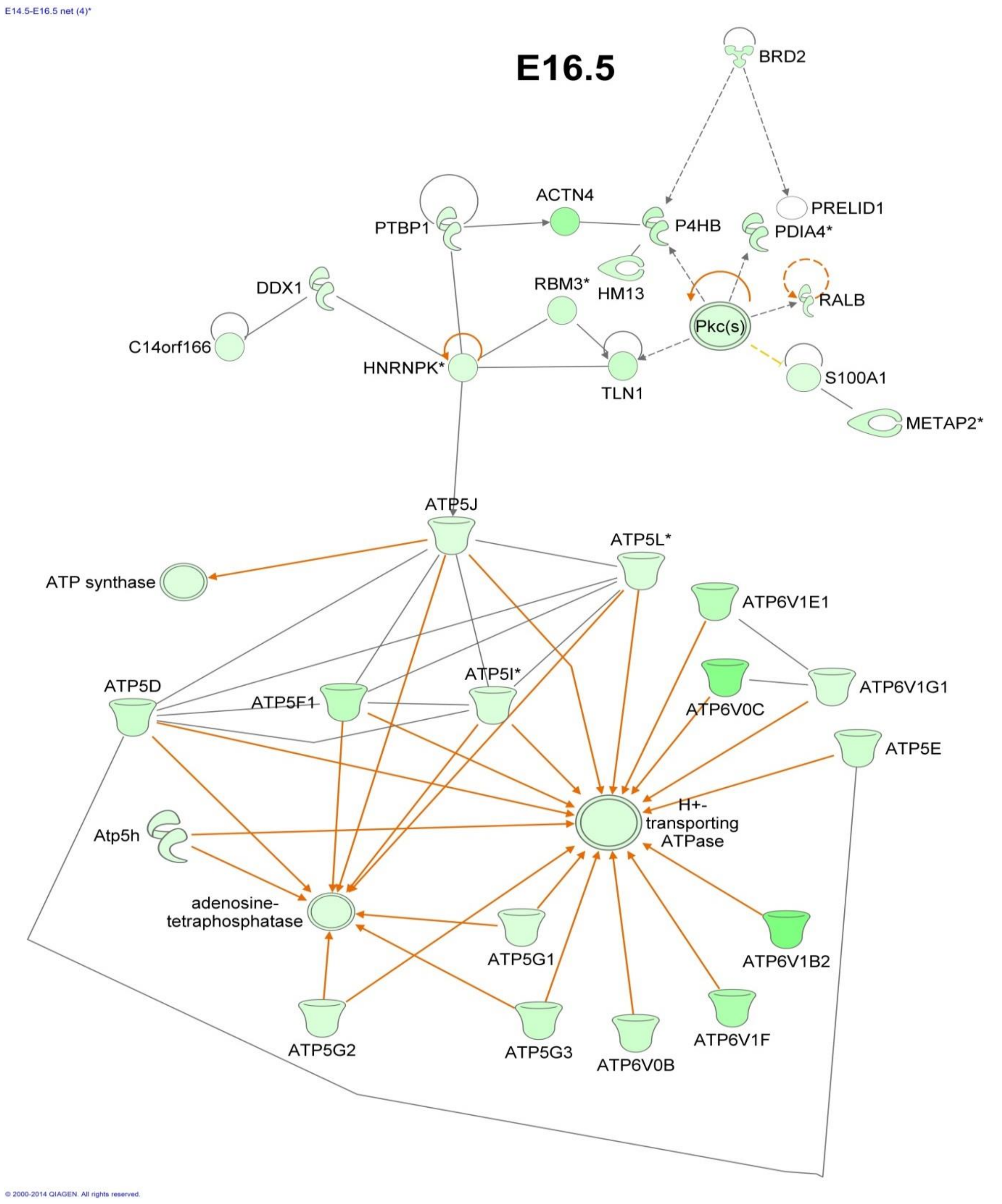

Network 4

Molecular transport, energy production, nucleic acid metabolism

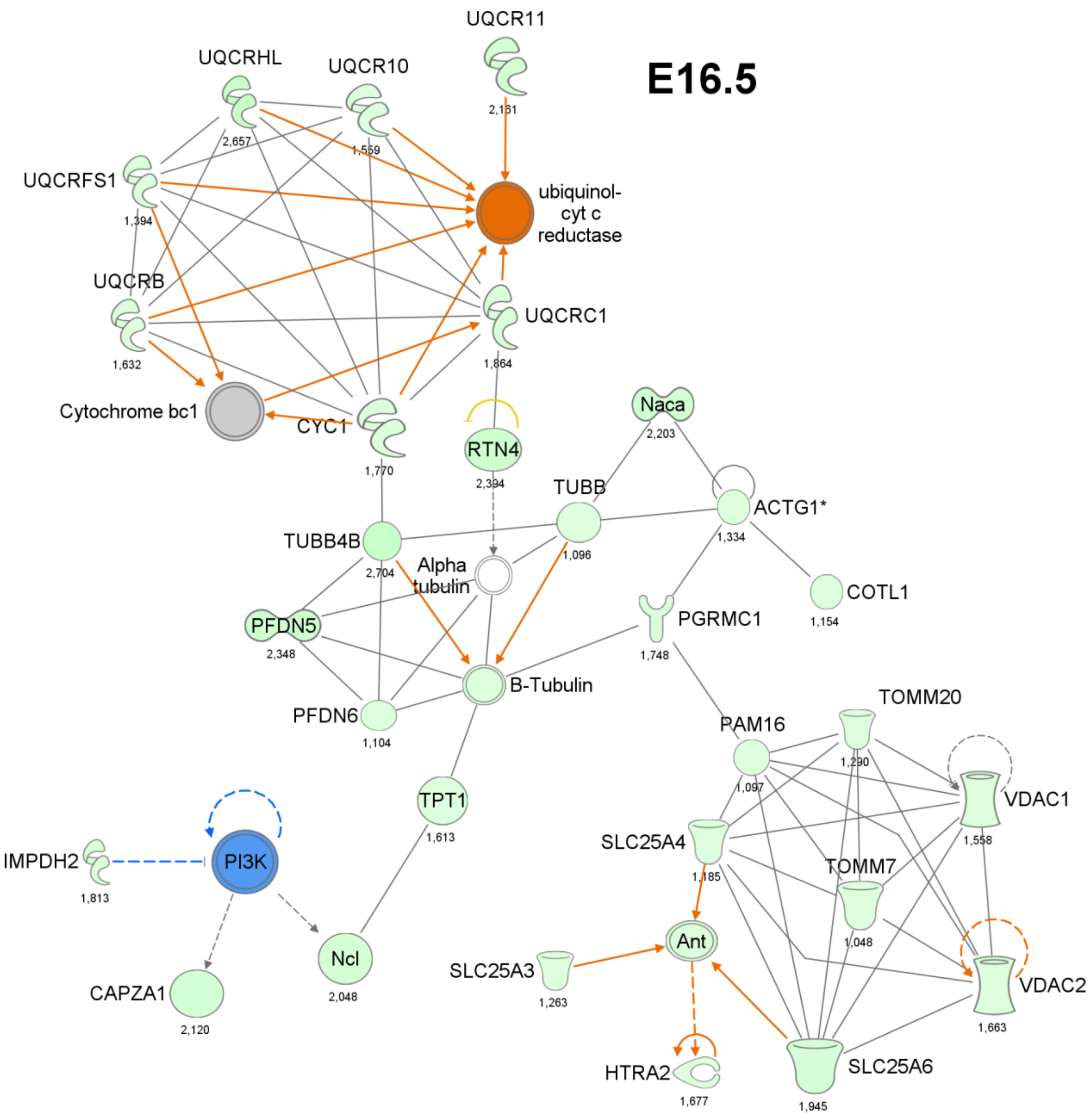

Network 5

Cellular assembly and organization

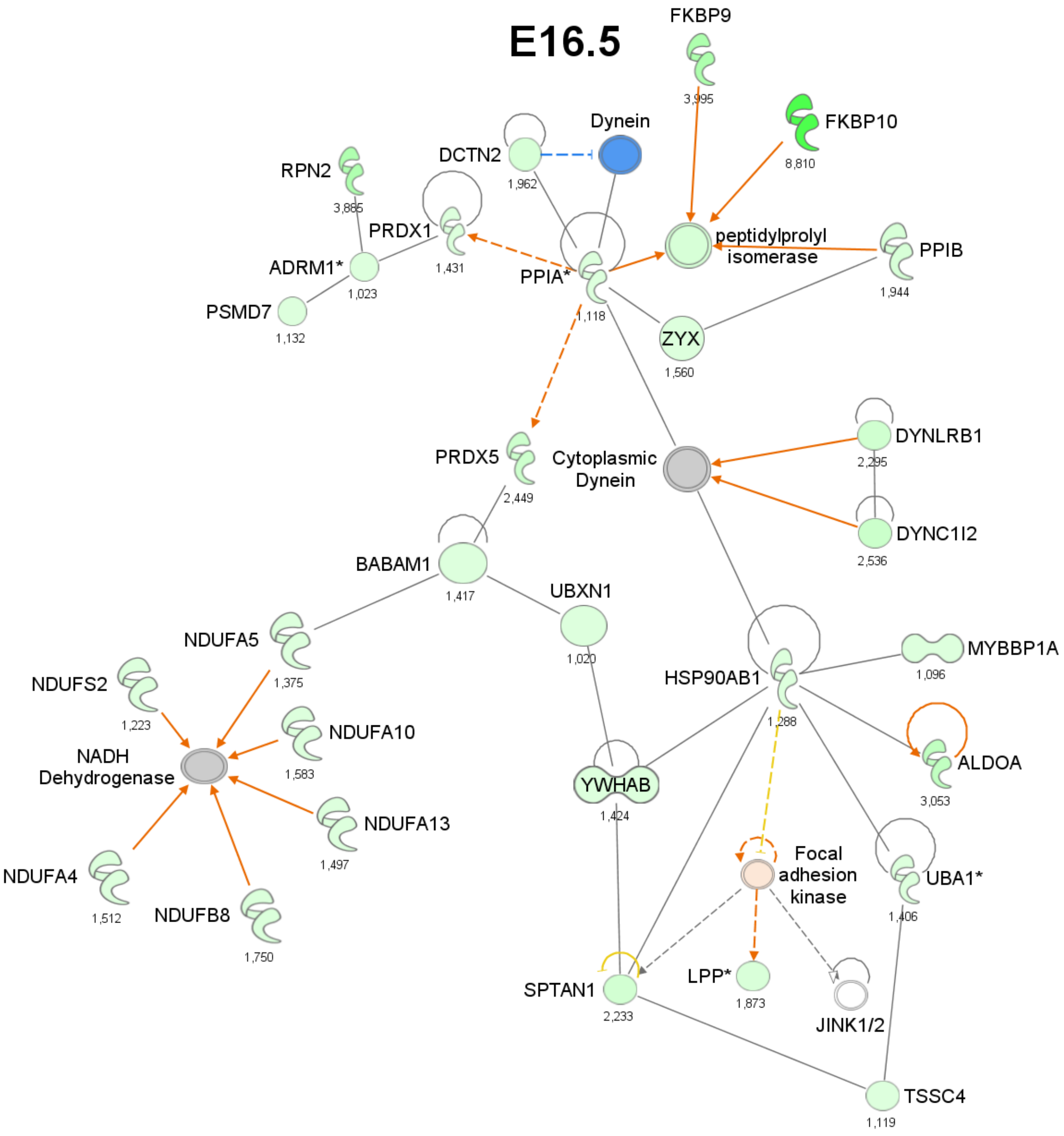

Network 6

Cell death, small molecule biochemistry

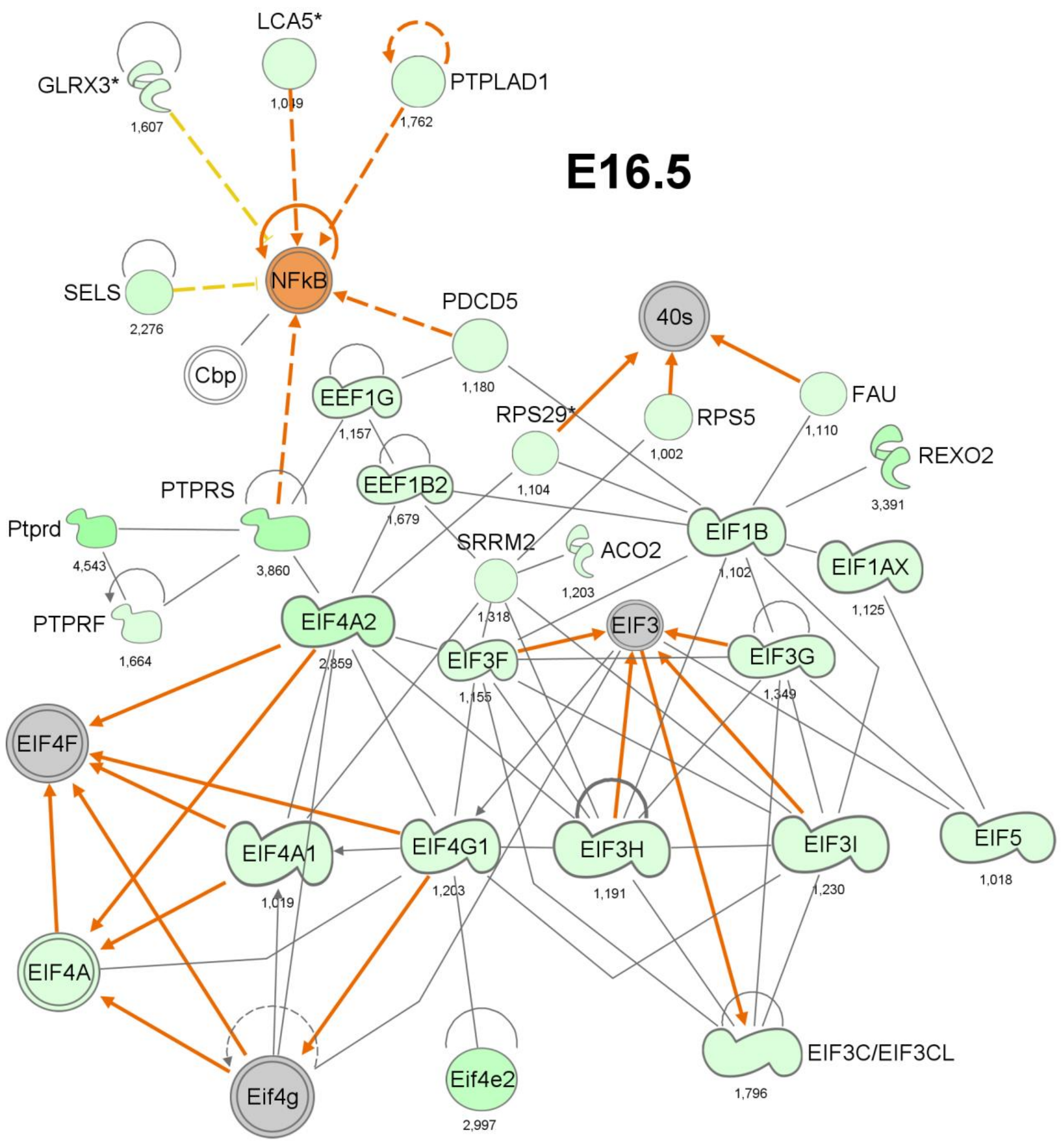

Network 7

Gene expression, protein synthesis

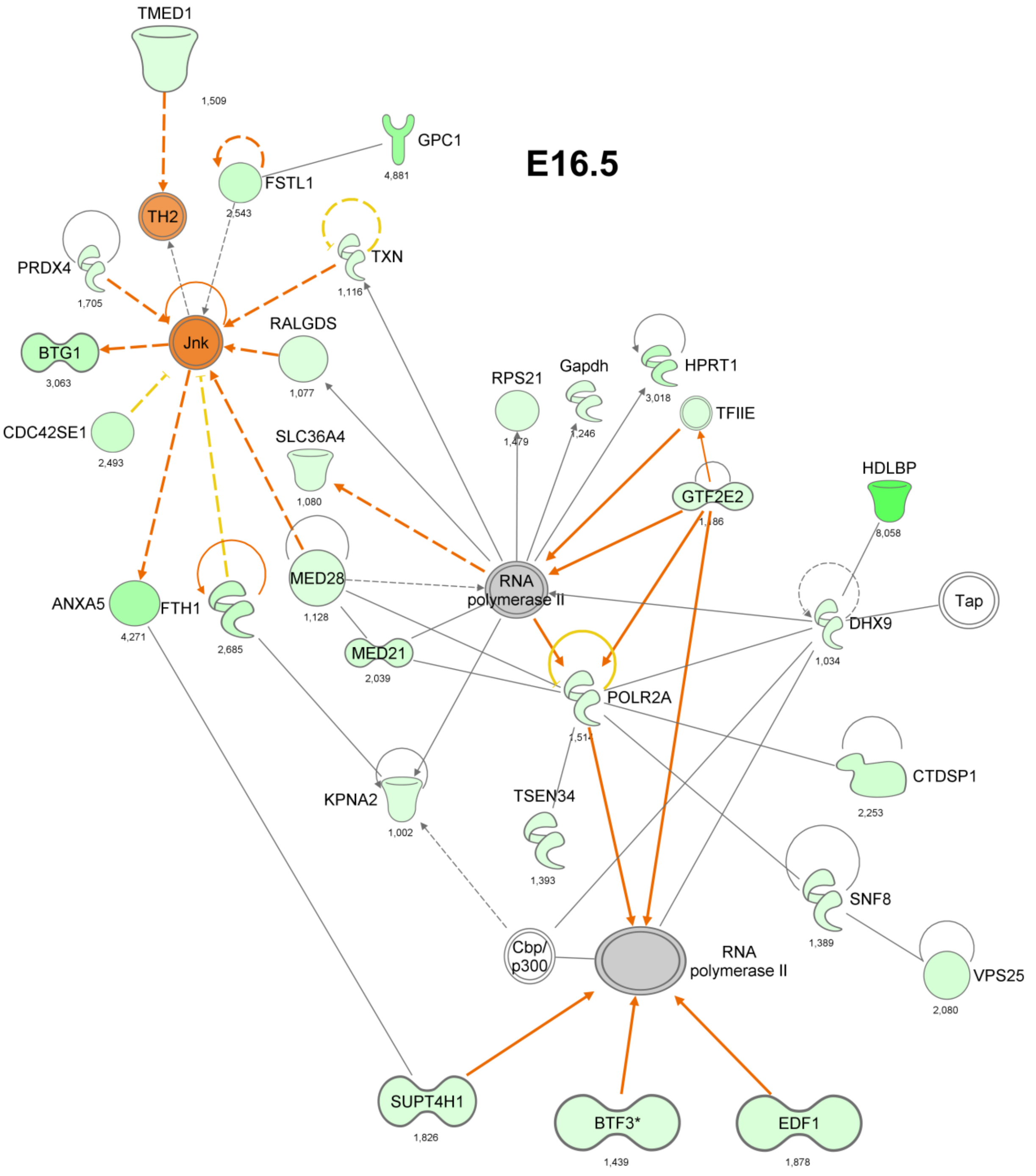

Network 8

Gene expression, cell cycle

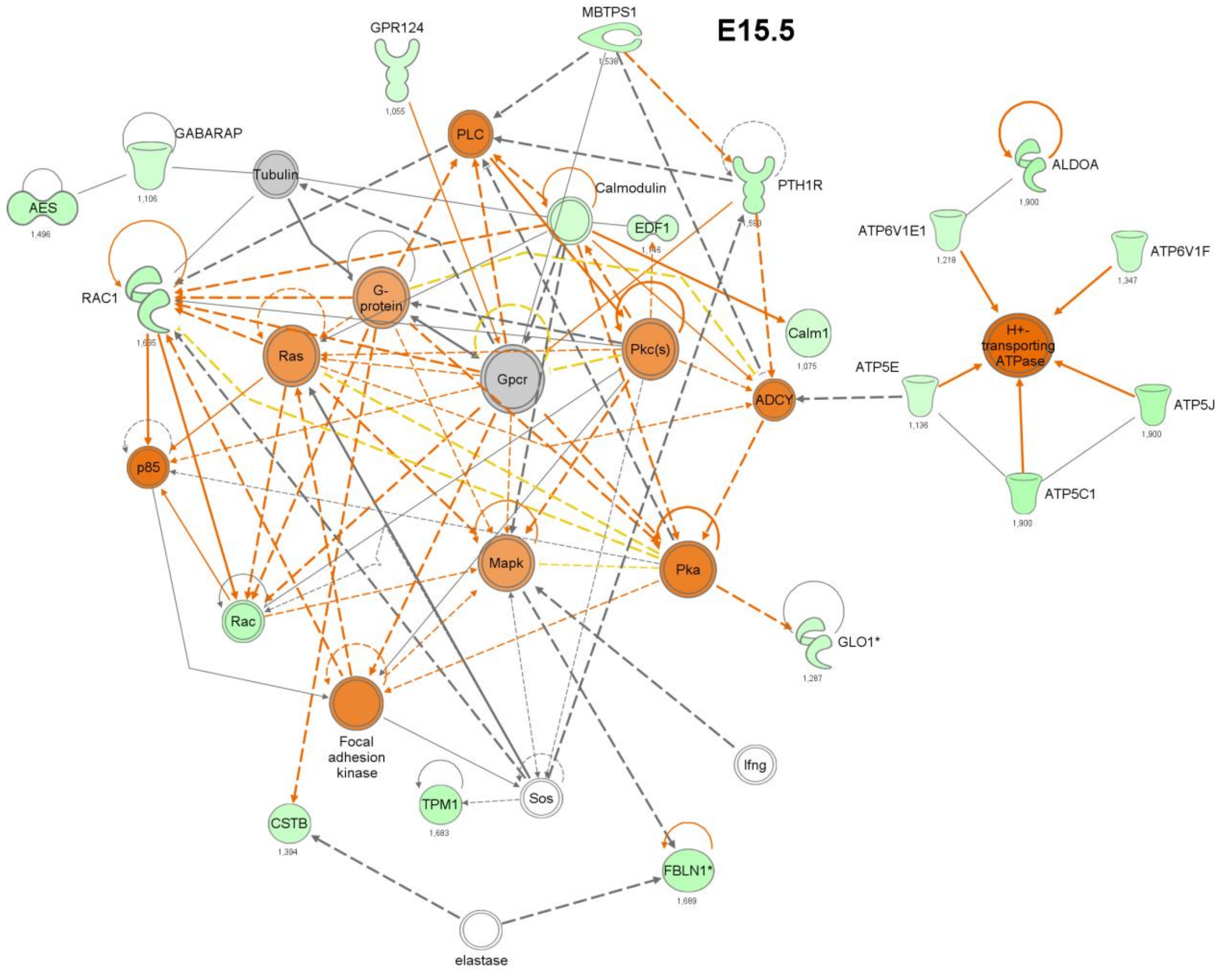

Network 9

DNA replication, energy production, nucleic acid metabolism

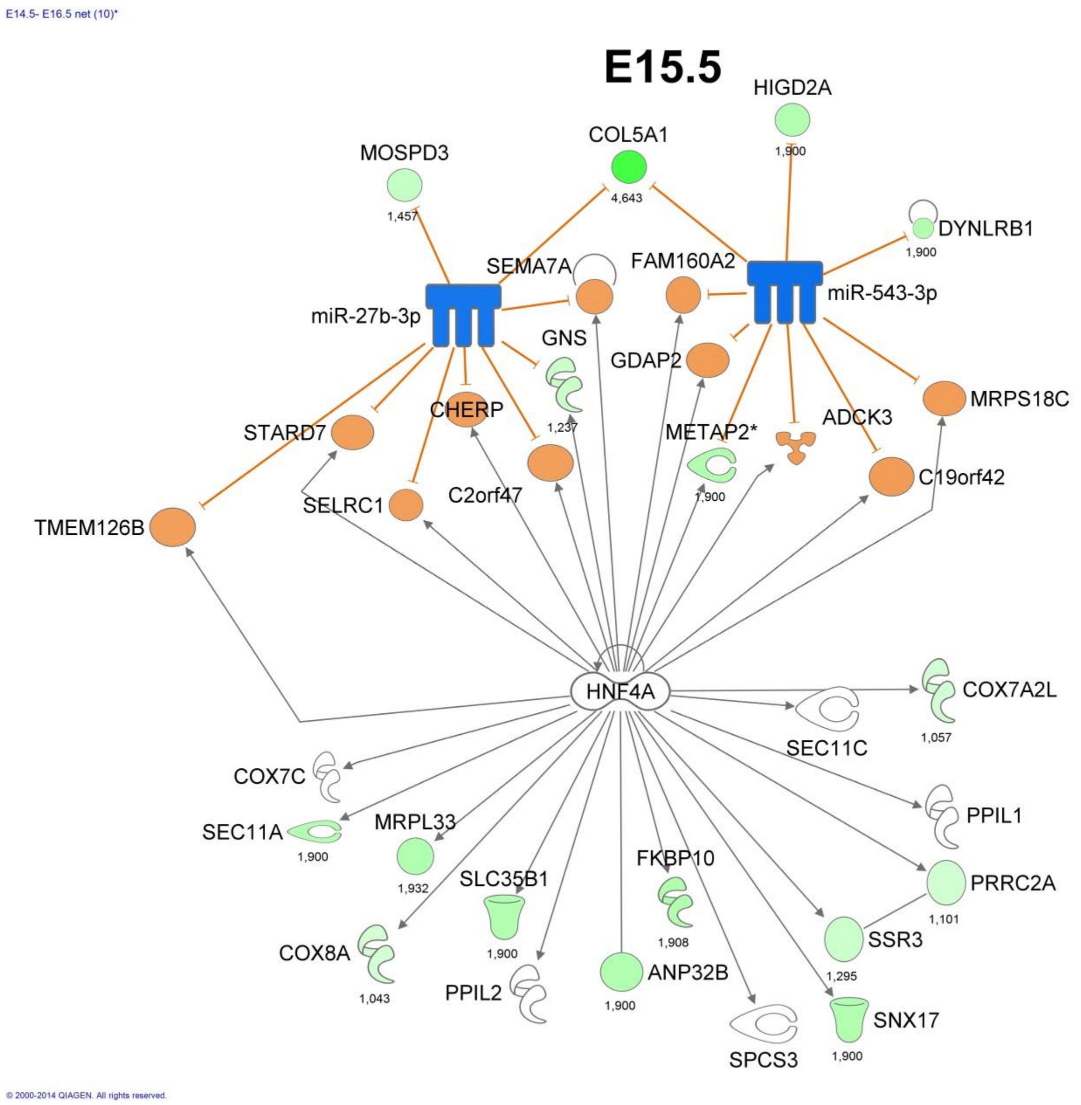

Network 10

Carbohydrate metabolism

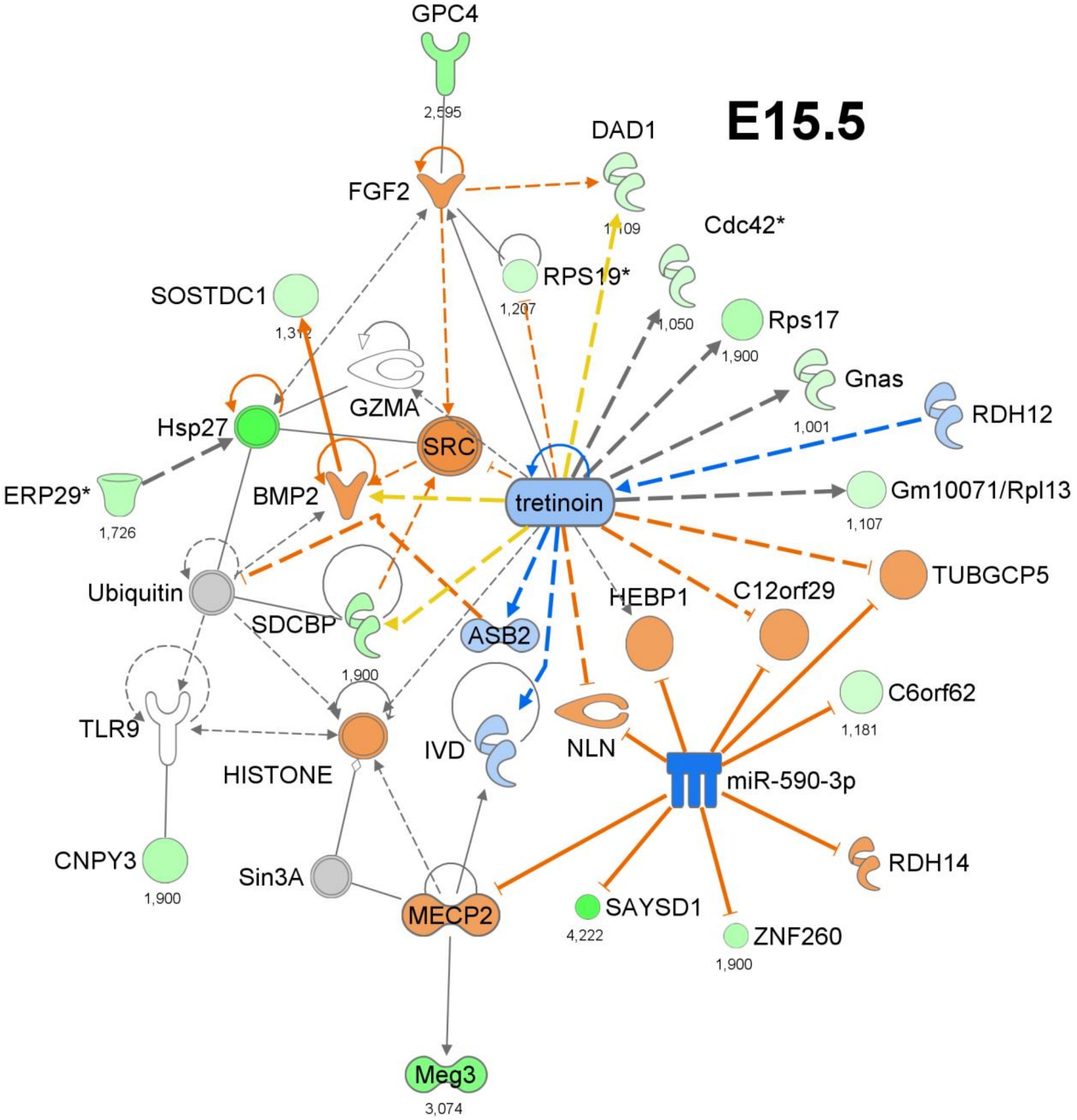

Network 11

Cellular development, cellular growth and proliferation, connective tissue development and function

E14.5-E16.5 net (9)**

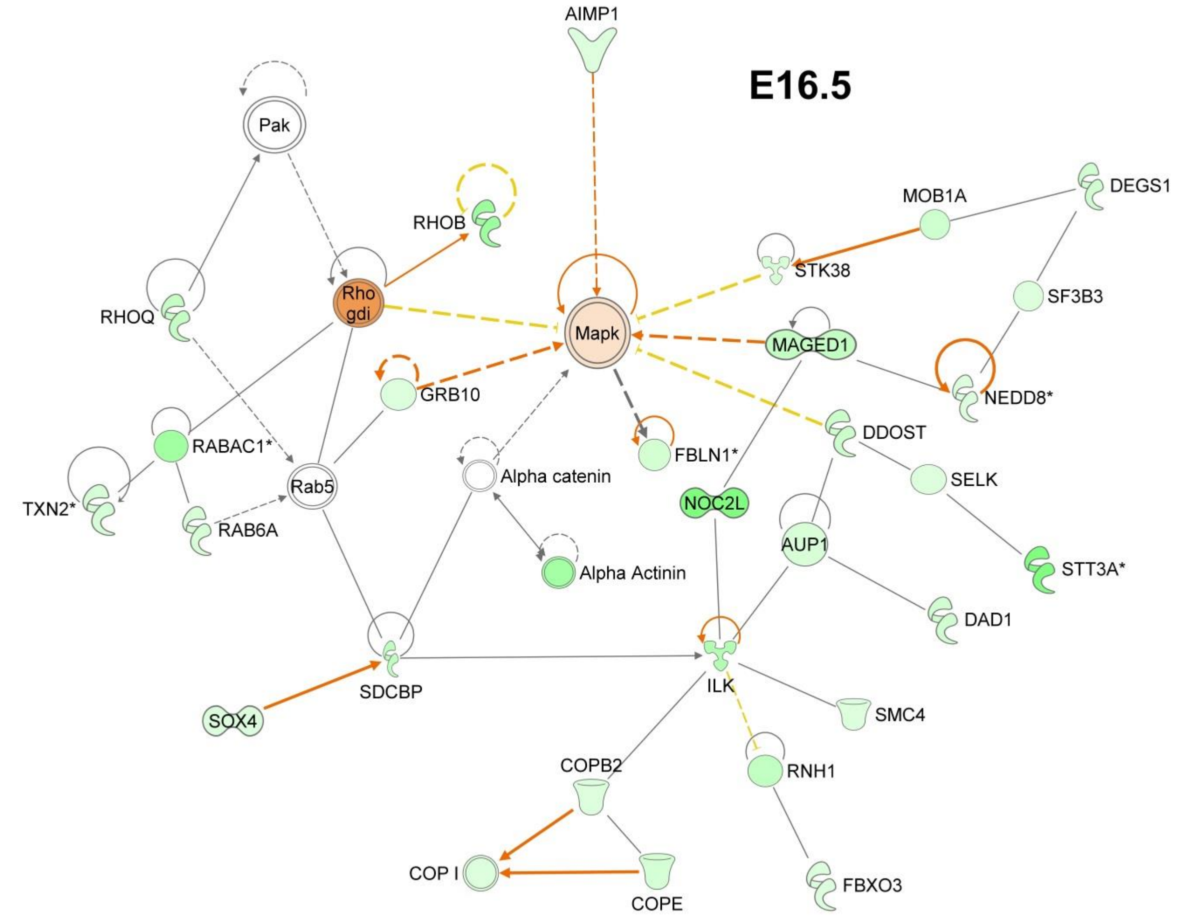

© 2000-2014 QIAGEN. All rights reserved.

Network 1

Cellular movment, cellular development, cellular growth and proliferation

E16.5

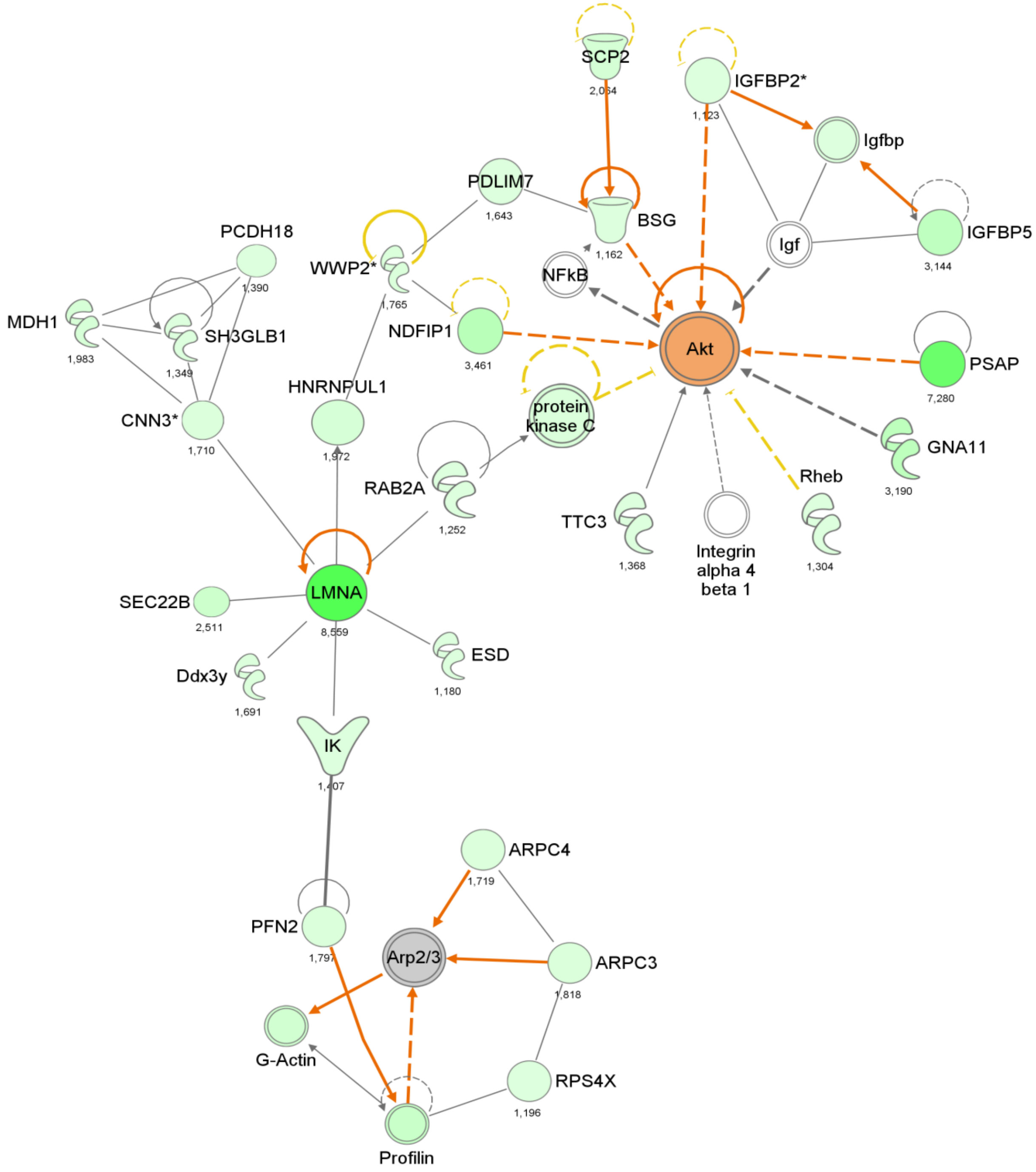

Network 2

Cell morphology

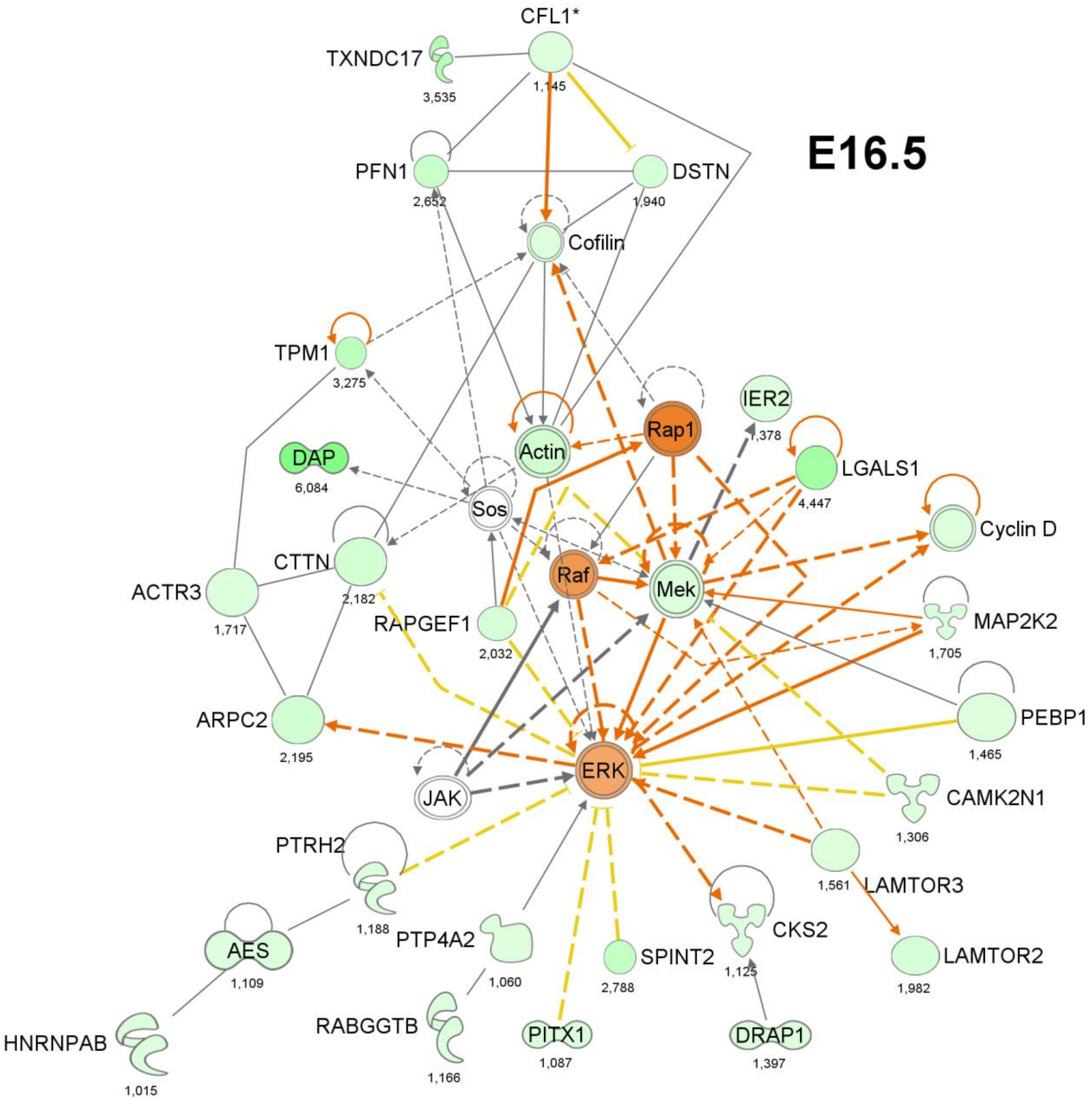

Network 3

Cellular and organization, tissue development, Cellular function and maintenance

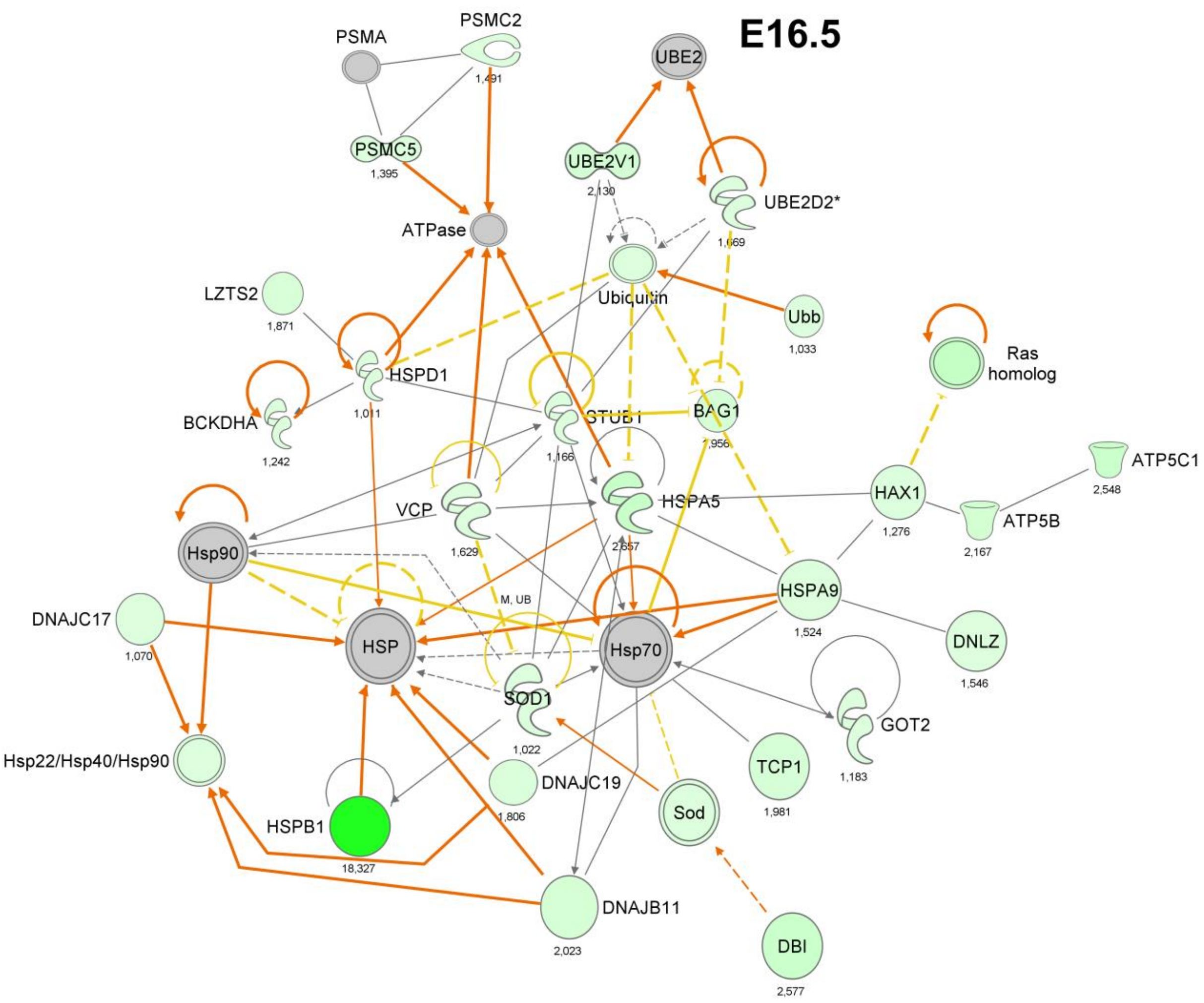

Network 4

DNA replication and recombination, energy production, nucleic acid metabolism

UBC E17.5 netverk

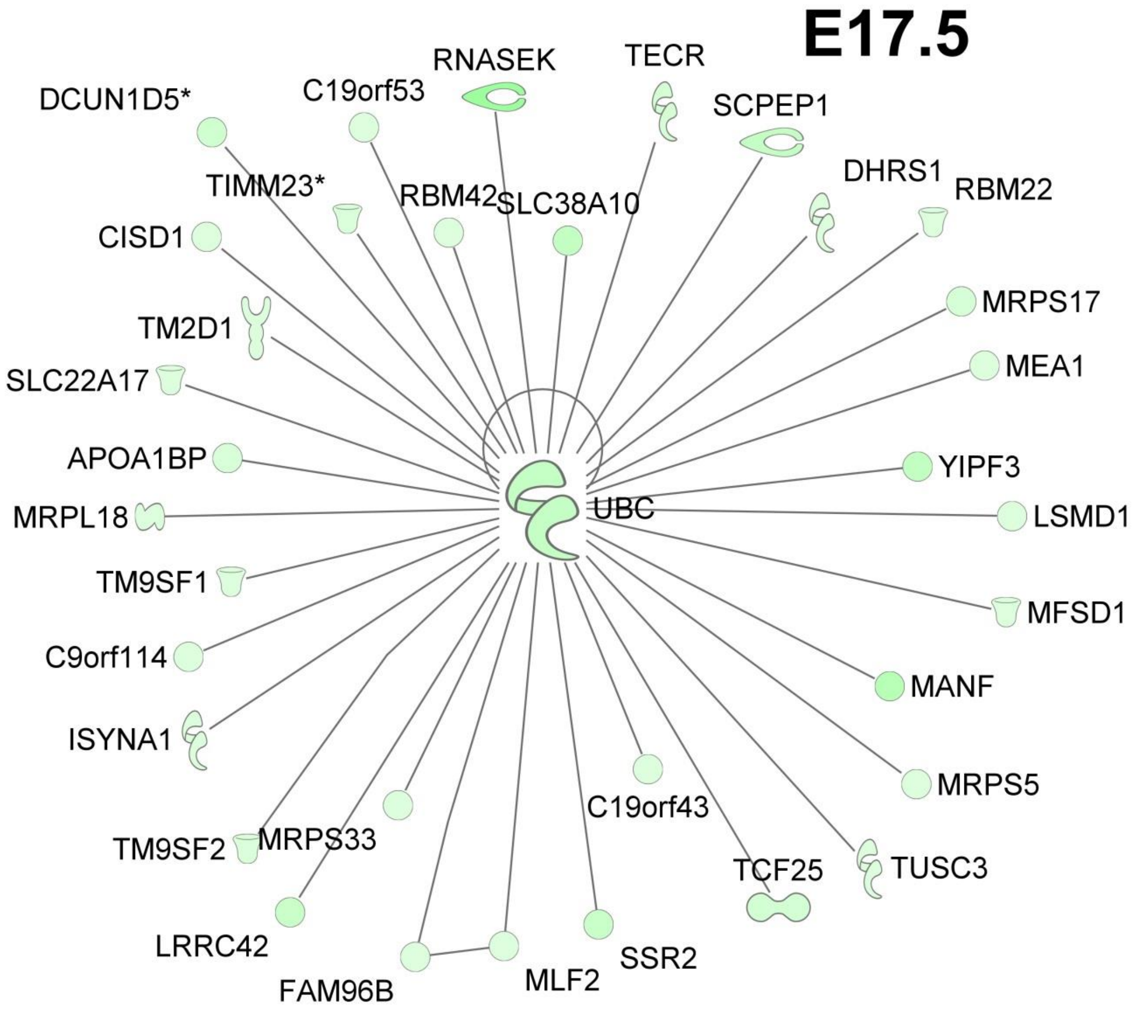

© 2000-2014 QIAGEN. All rights reserved.

Network 1

Lipid metabolism, nucleic acid metabolism, small molecule biochemistry, Senescence

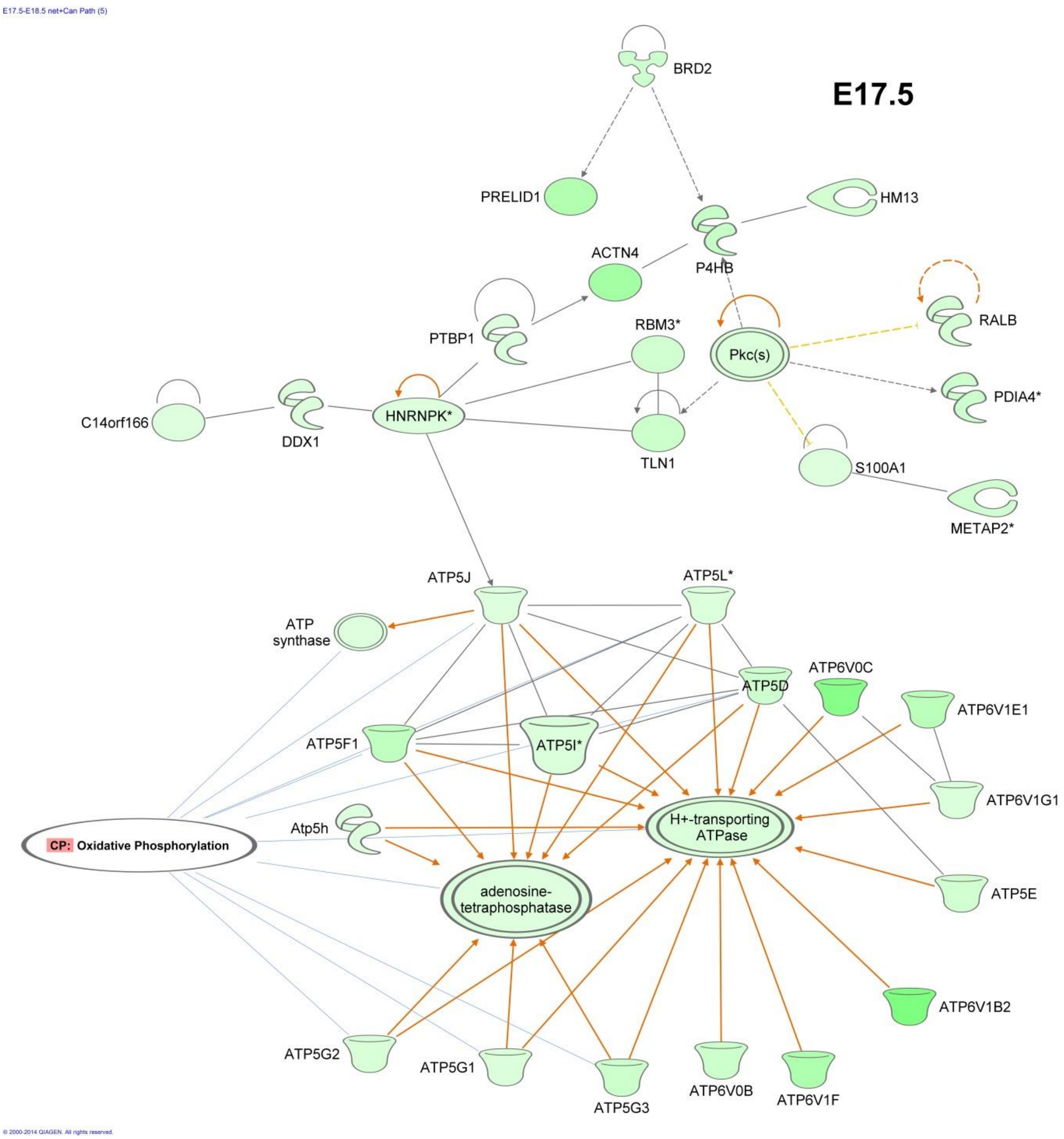

Network 2

Molecular transport, energy production, nucleic acid metabolism

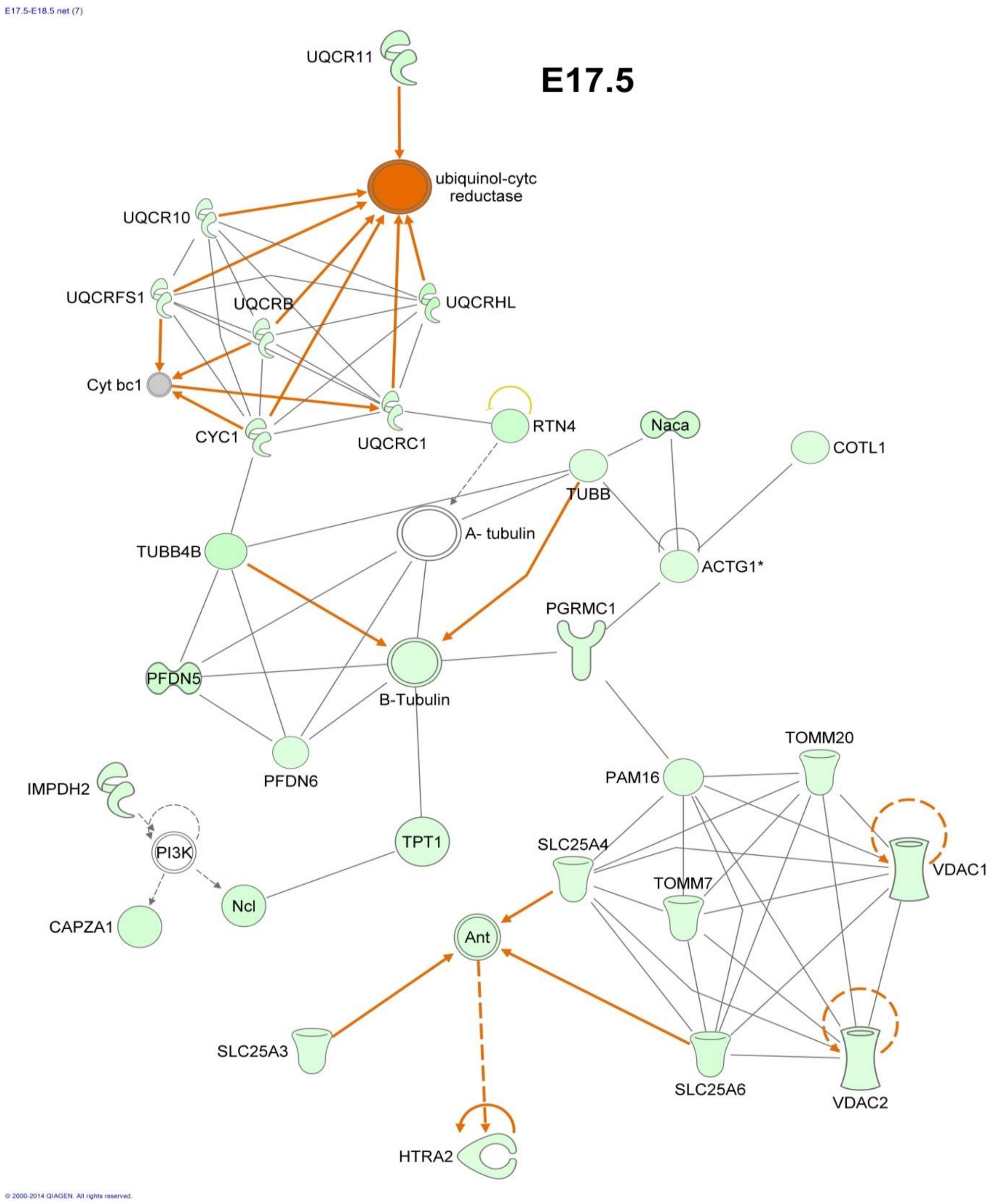

Network 3

Cellular assembly and organization

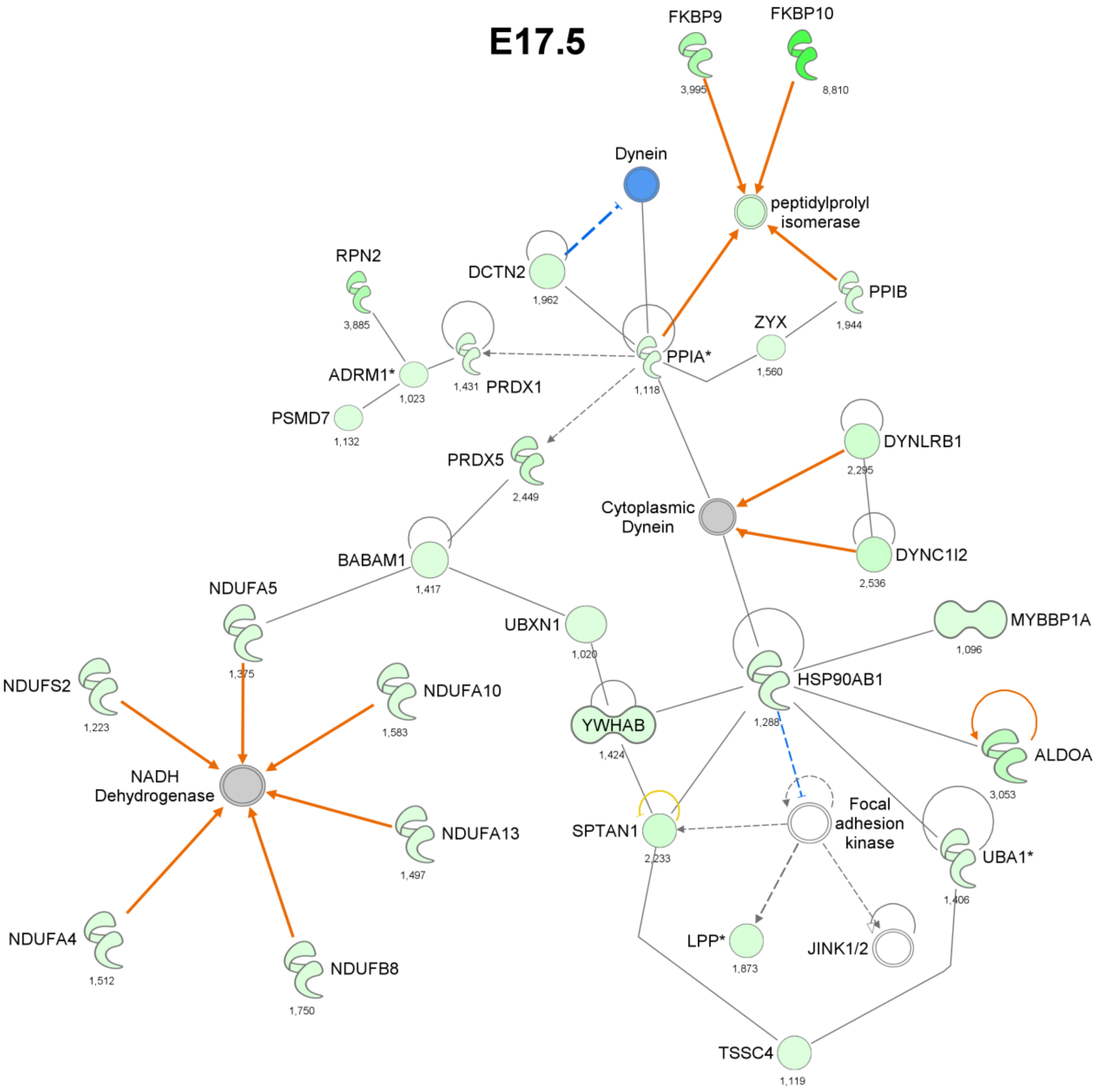

Network 4

Cell death, Small molecule biochemistry

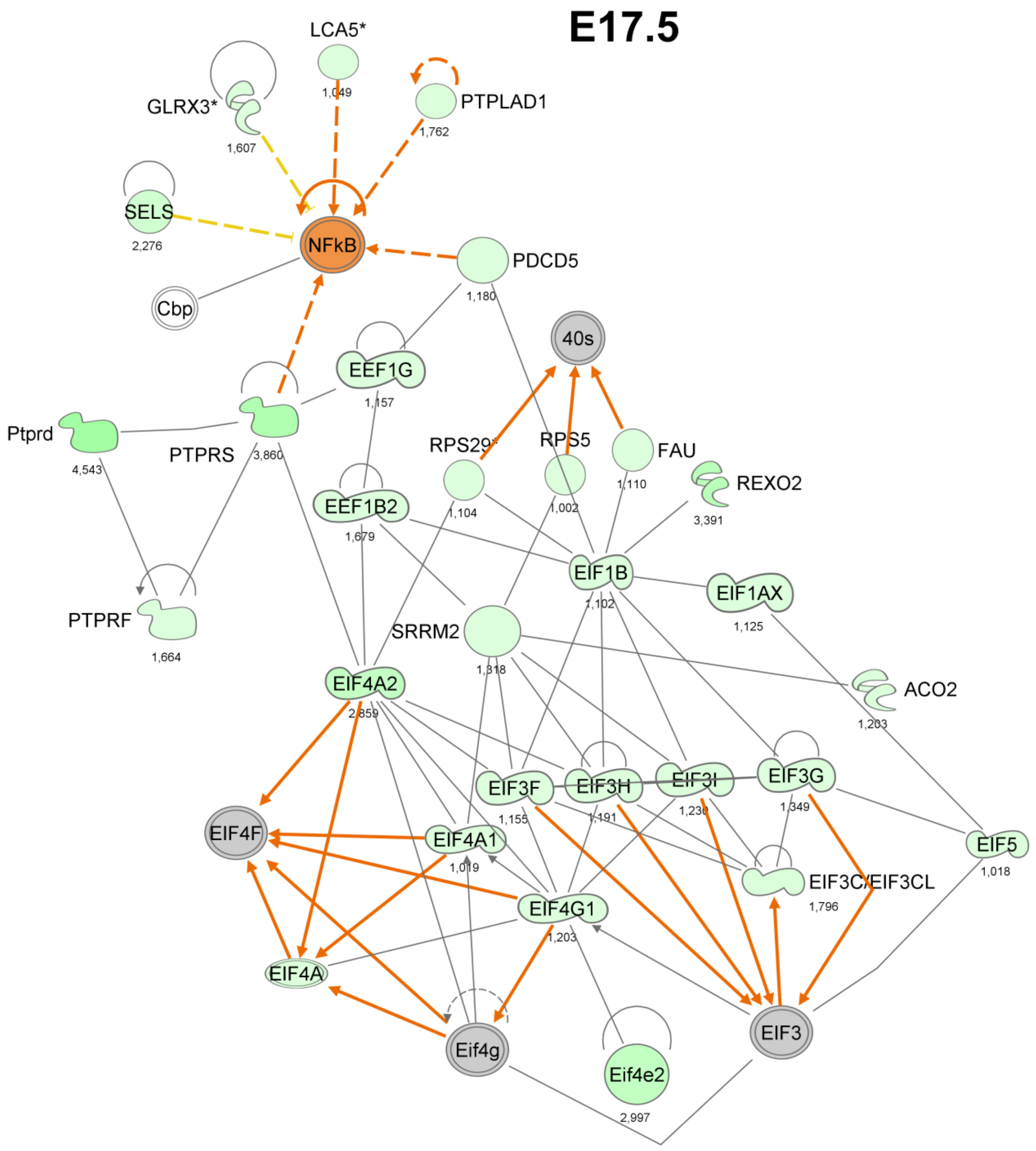

Network 5

Gene expression, protein synthesis

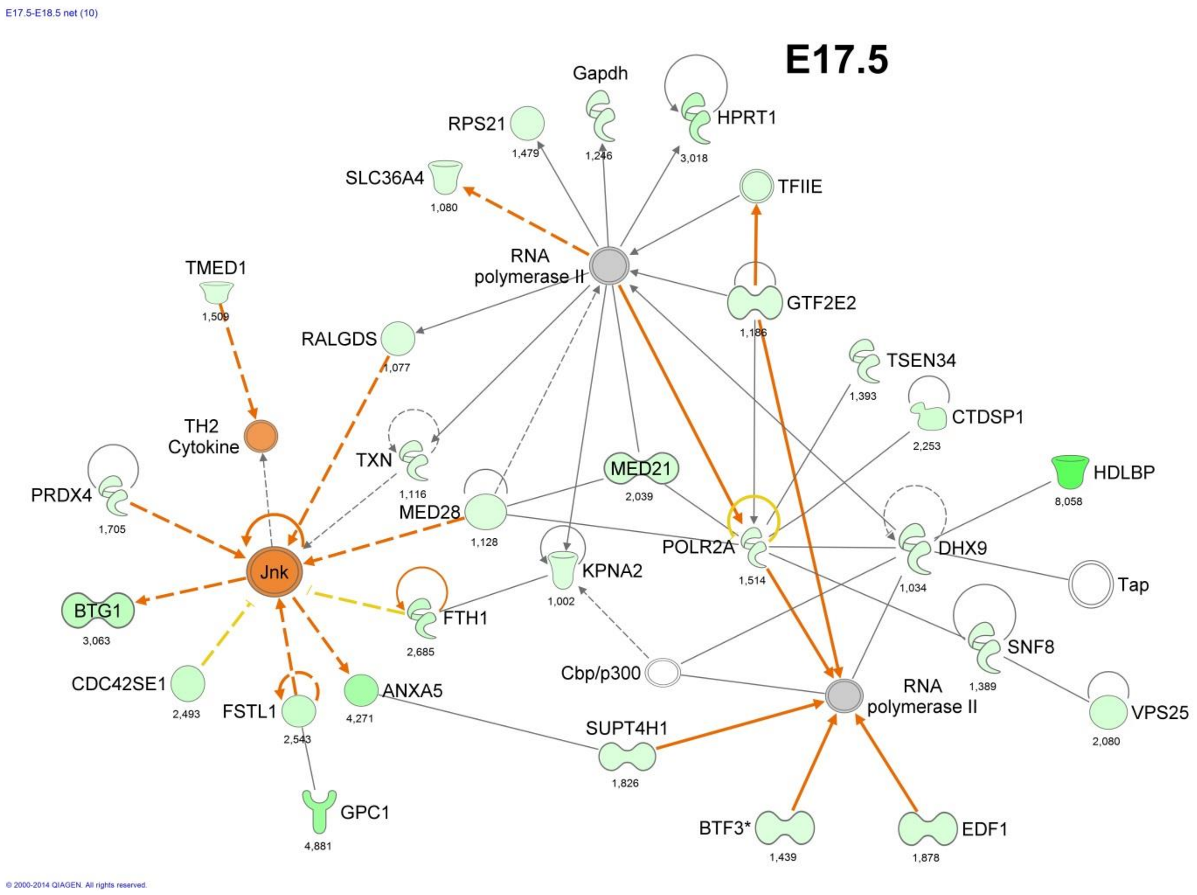

Network 6

Gene expression, cell cycle

E17.5-E18.5 net (11)

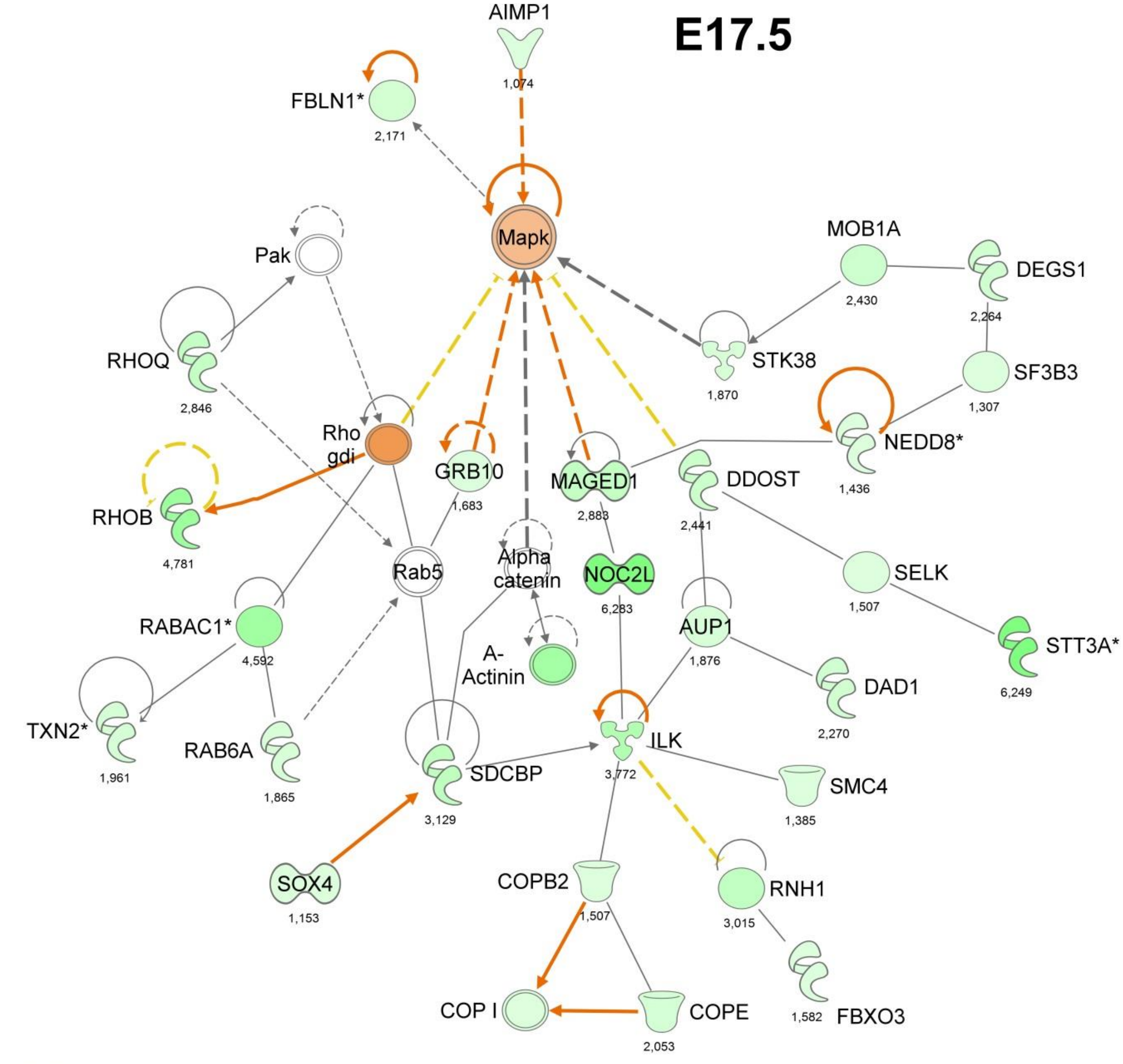

© 2000-2014 QIAGEN. All rights reserved.

Network 7

Cell morphology, cellular growth and proliferation

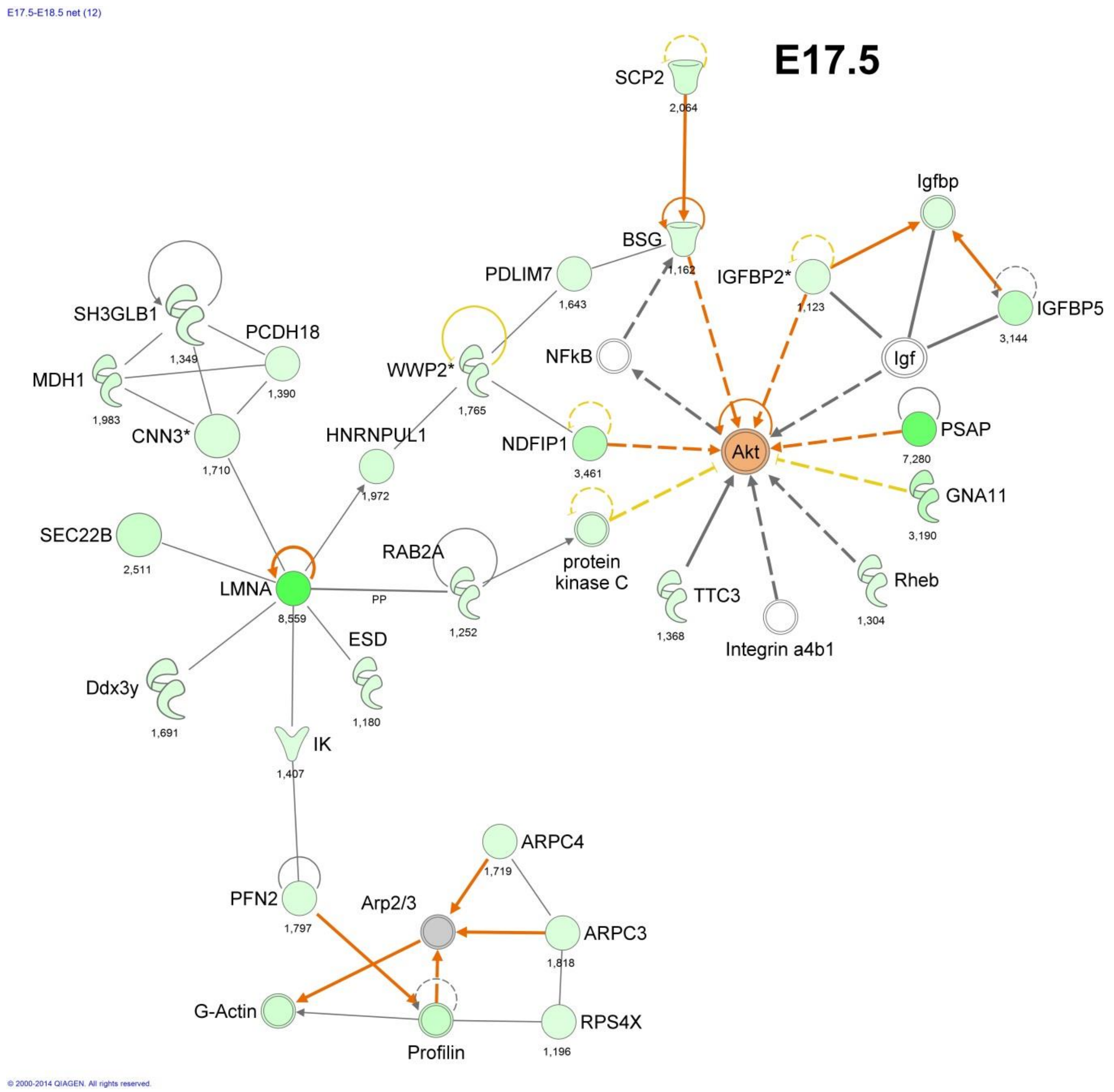

© 2000-2014 QIAGEN. All rights reserved.

Network 8

Cellular morphology, cellular growth and proliferation, embryonic development

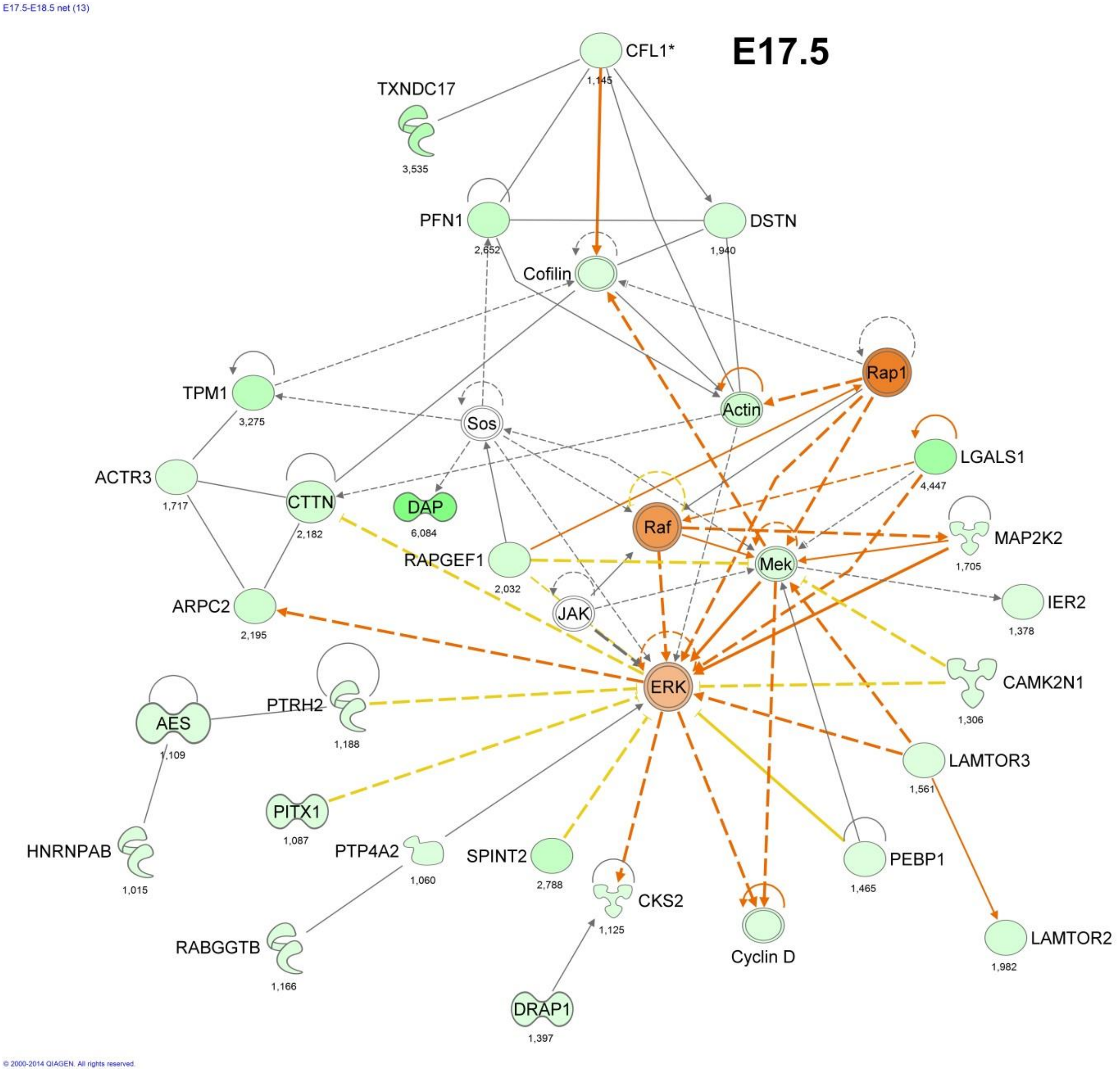

Network 9

Cellular assembly and organization, cellular function maintenance, tissue development

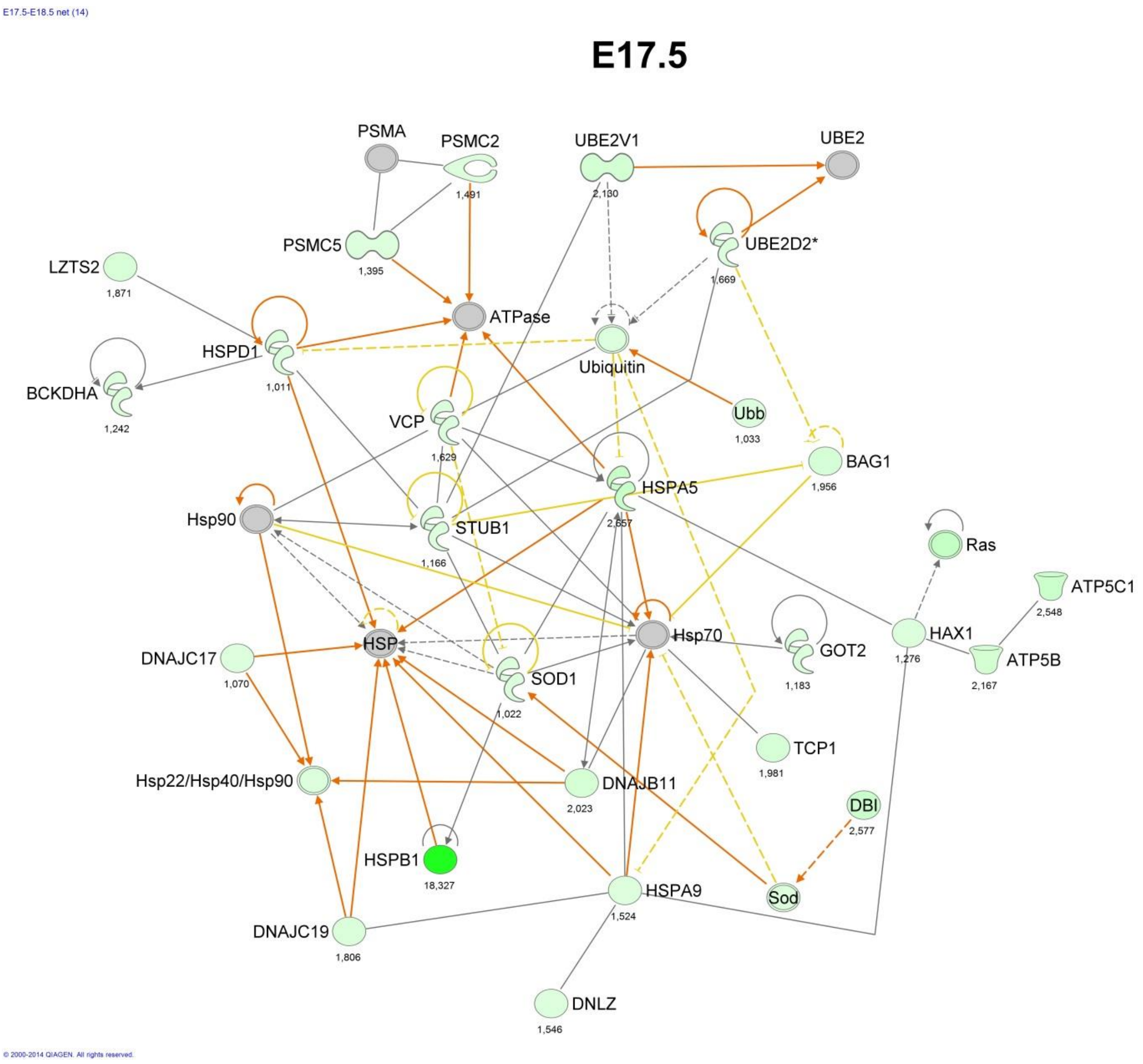

Network 10

DNA replication, recombination and repair, energy production, nucleic acid metabolism

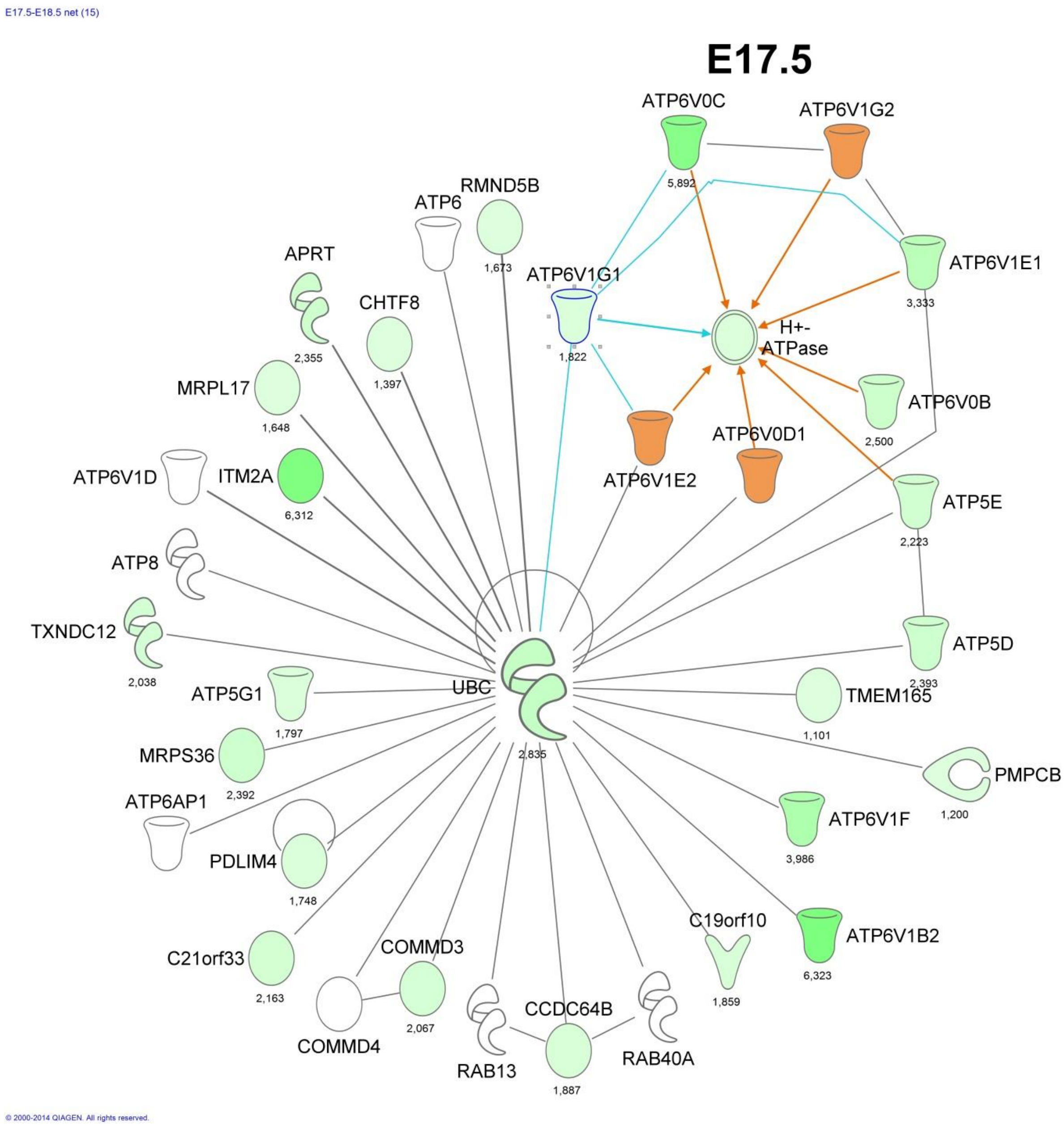

Network 11

Molecular transport, energy production, nucleic acid metabolism

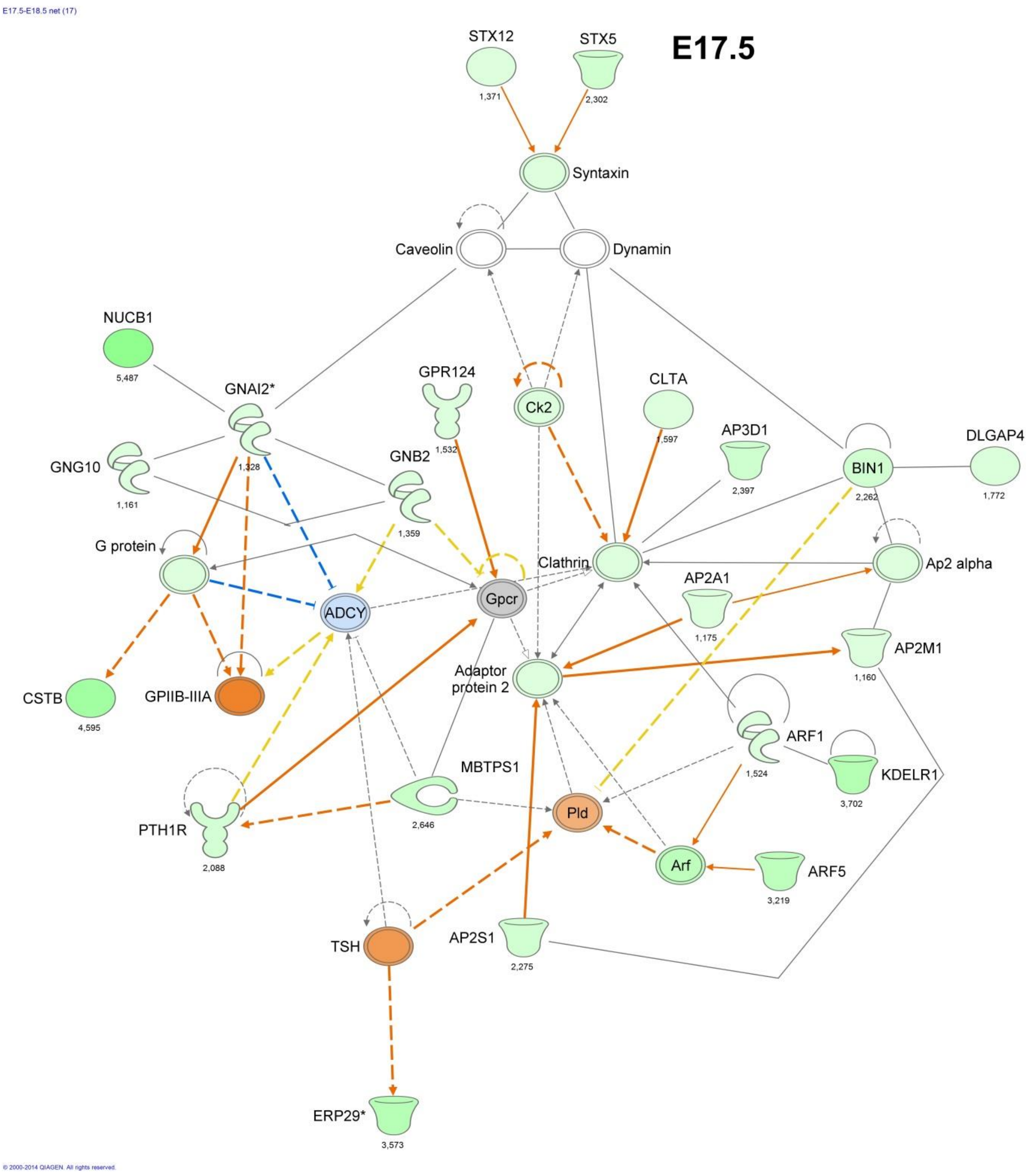

Network 12

Molecular transport, protein trafficking, connective tissue development and functions

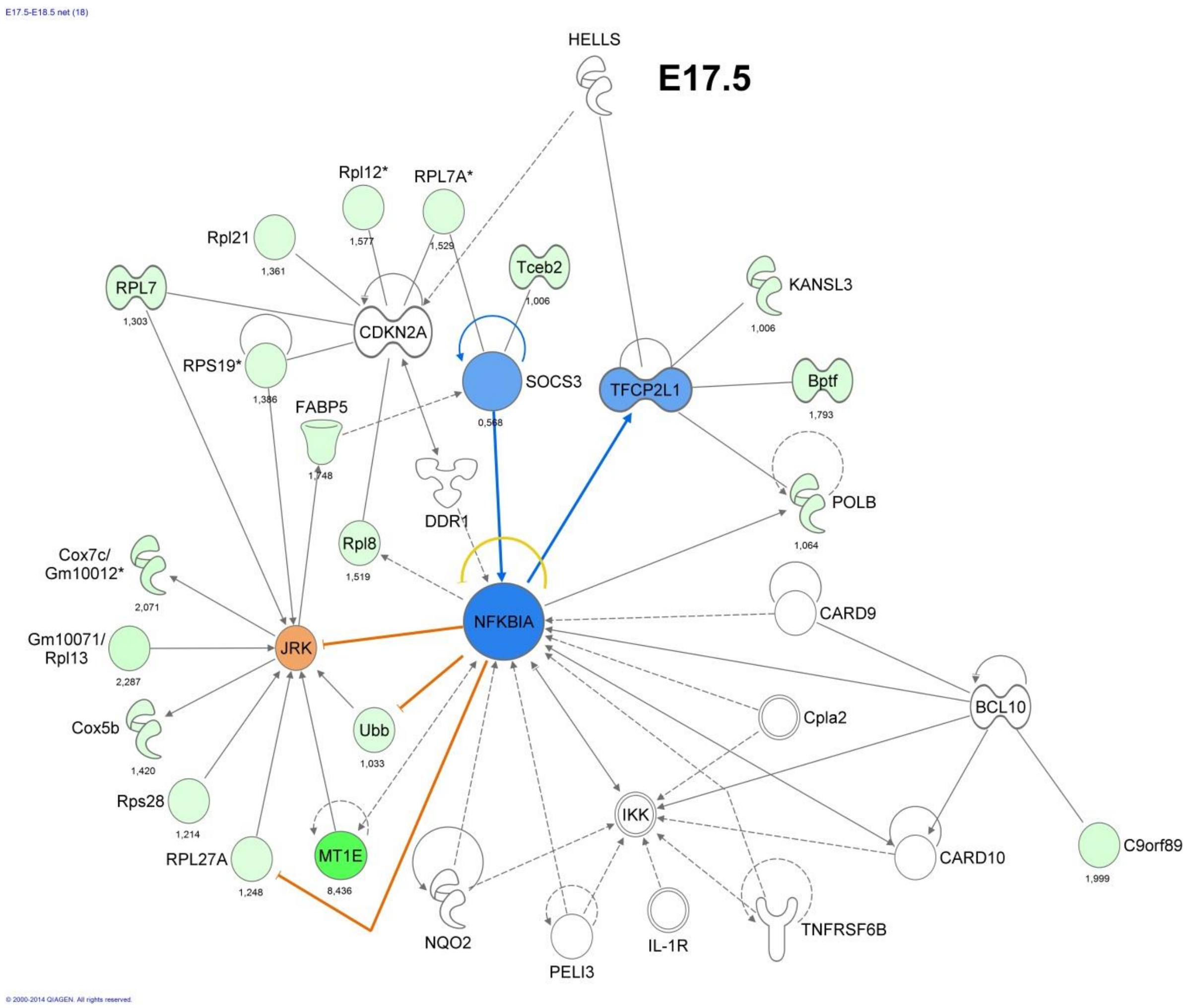

Network 13

Tissue morphology, cellular development, hematological system development and function

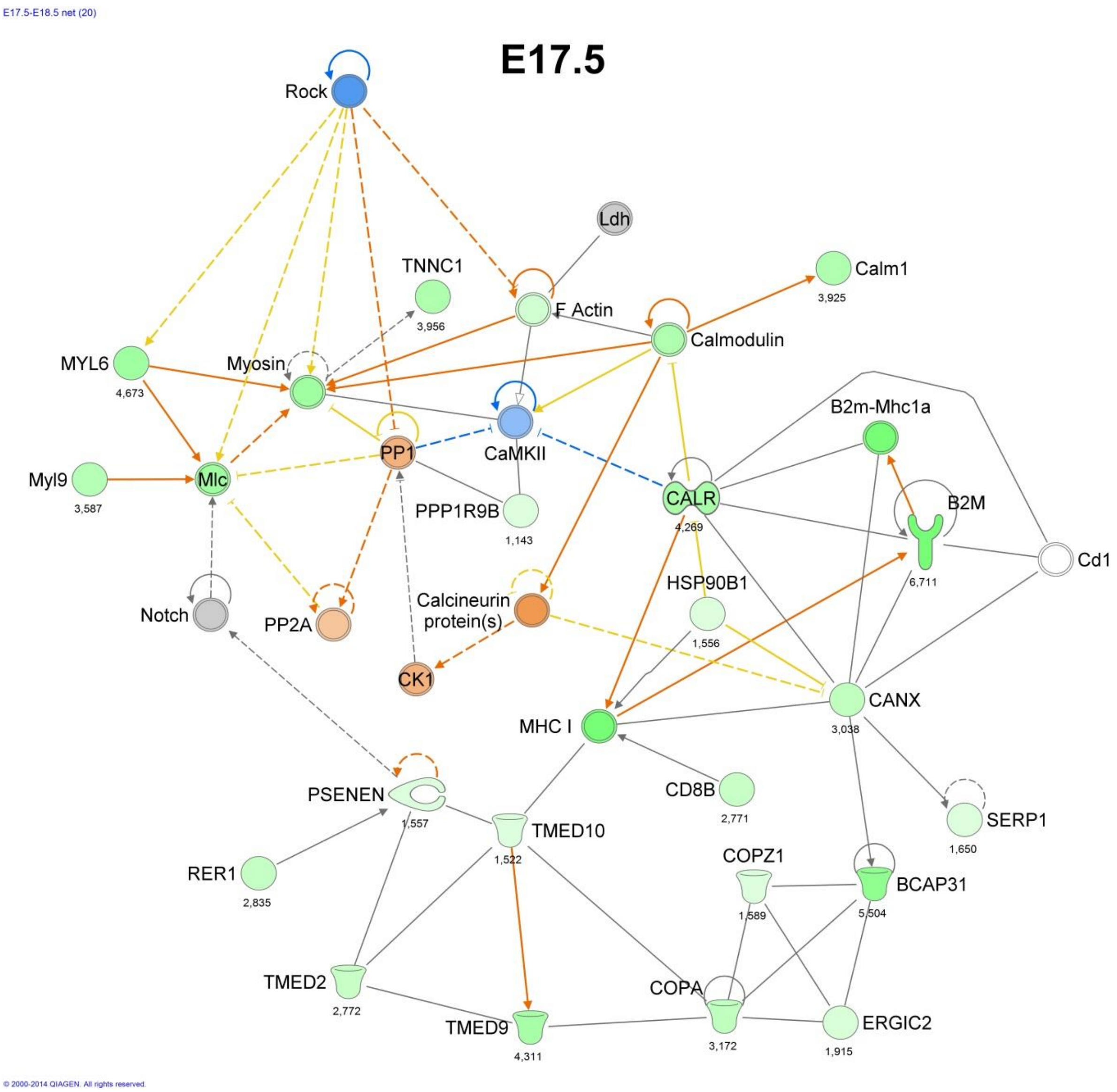

© 2000-2014 QIAGEN. All rights reserved.

Network 14

Cell morphology, lipid metabolism

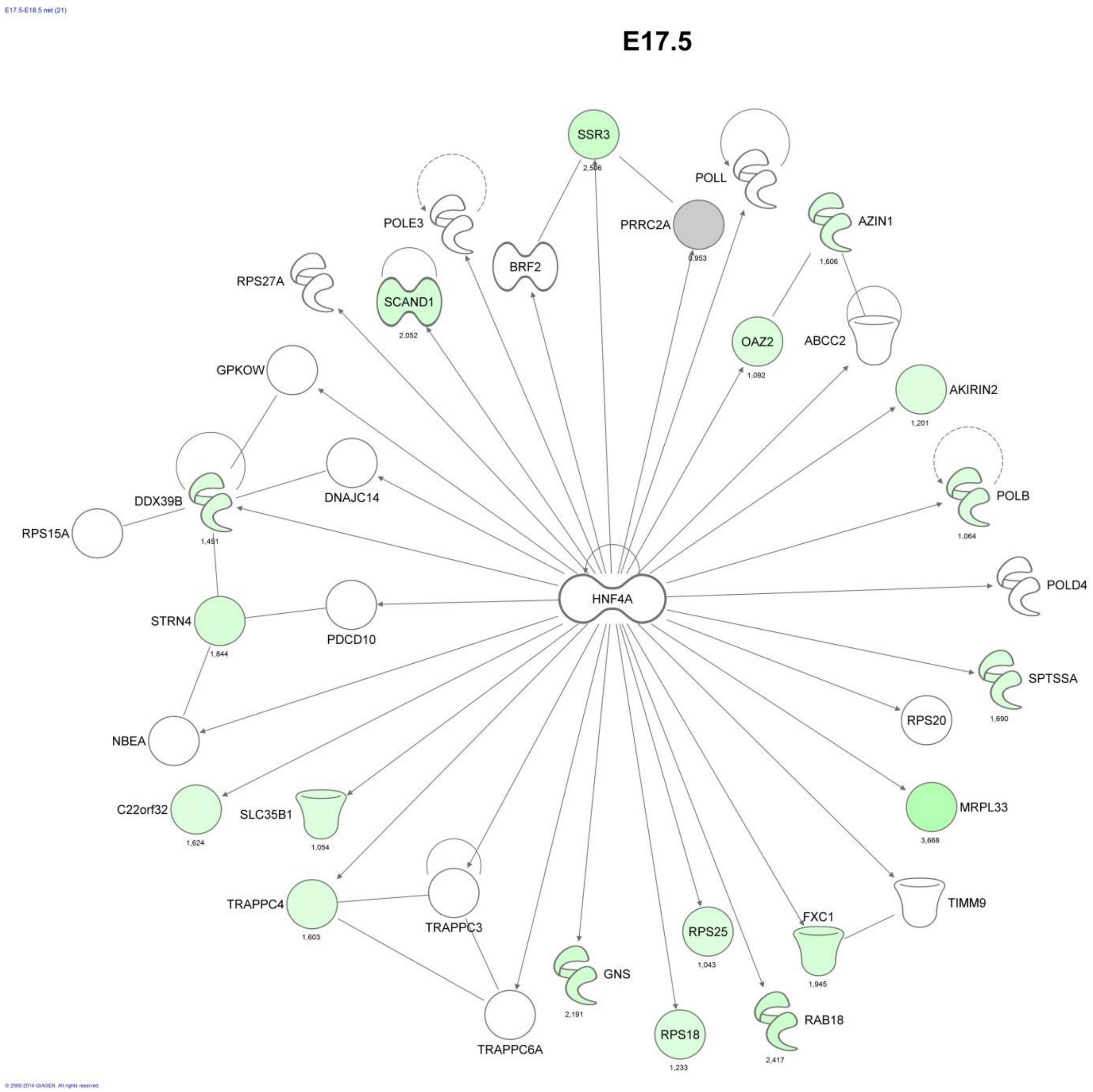

Network 15

Cell morphology, small molecule biochemistry, cellular assembly and organization

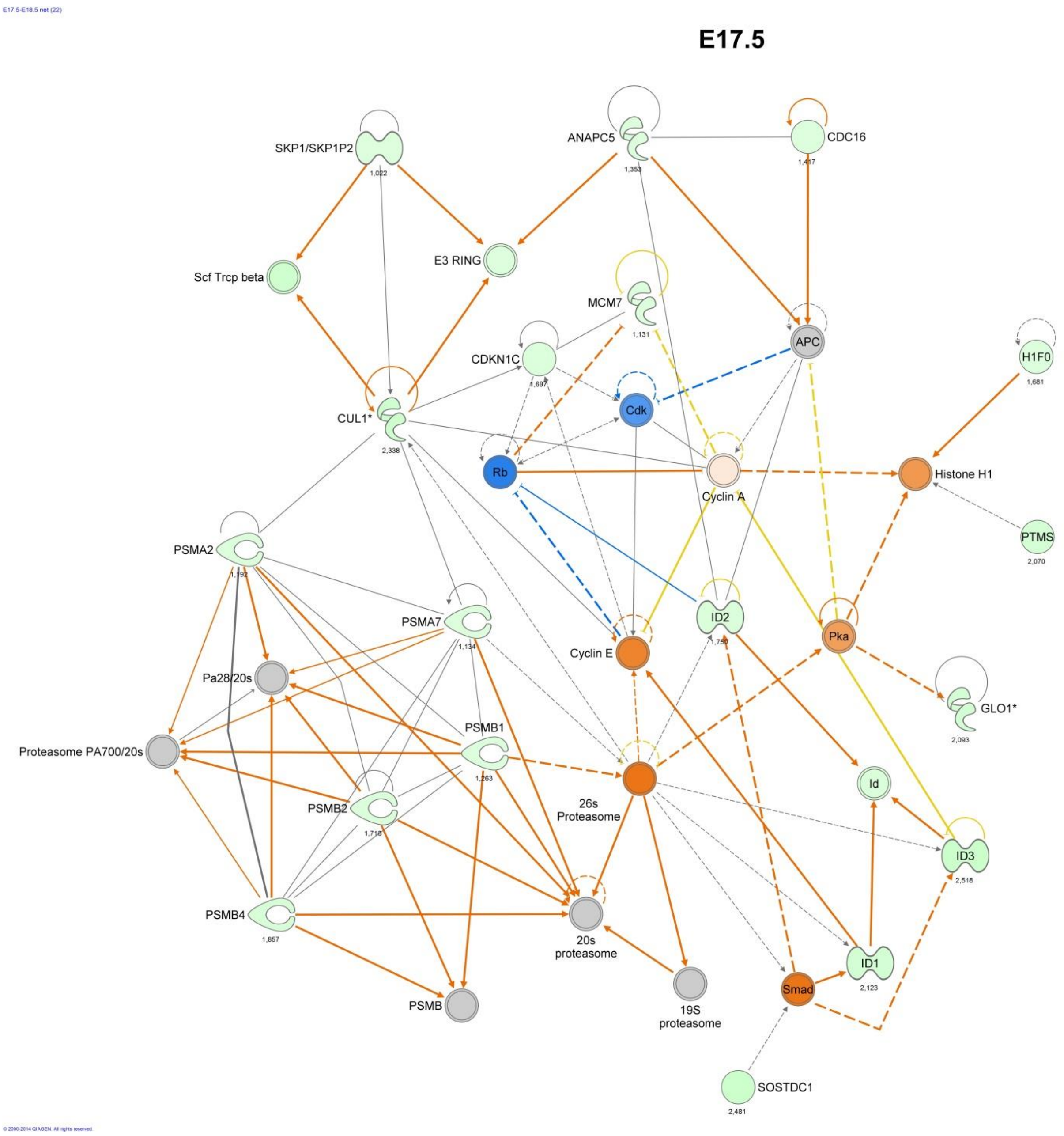

© 2000-2014 QIAGEN. All rights reserved.

Network 16

Gene expression, cellular development, cellular growth and proliferation

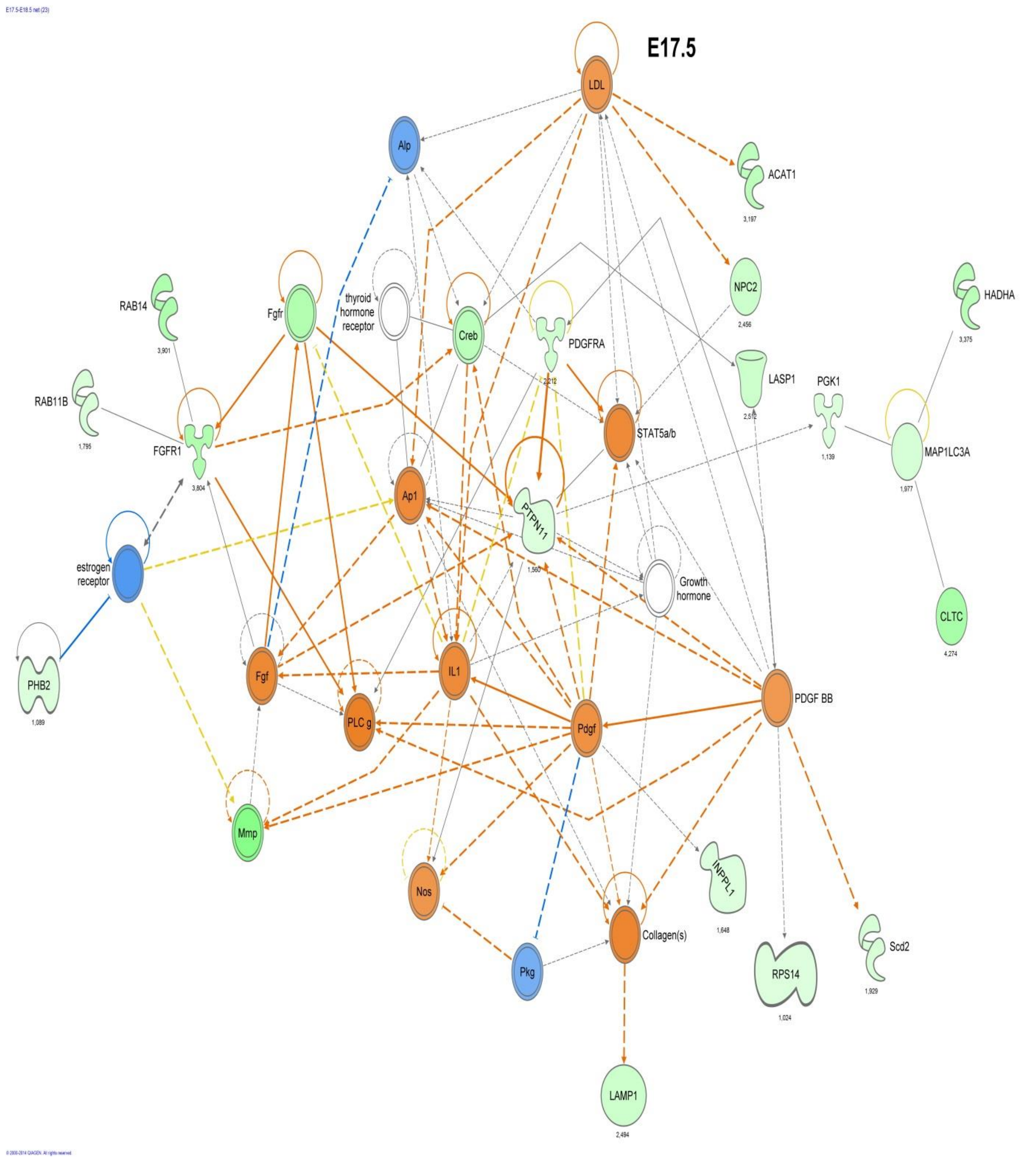

Network 17

Lipid metabolism, small molecule biochemistry, cell cycle

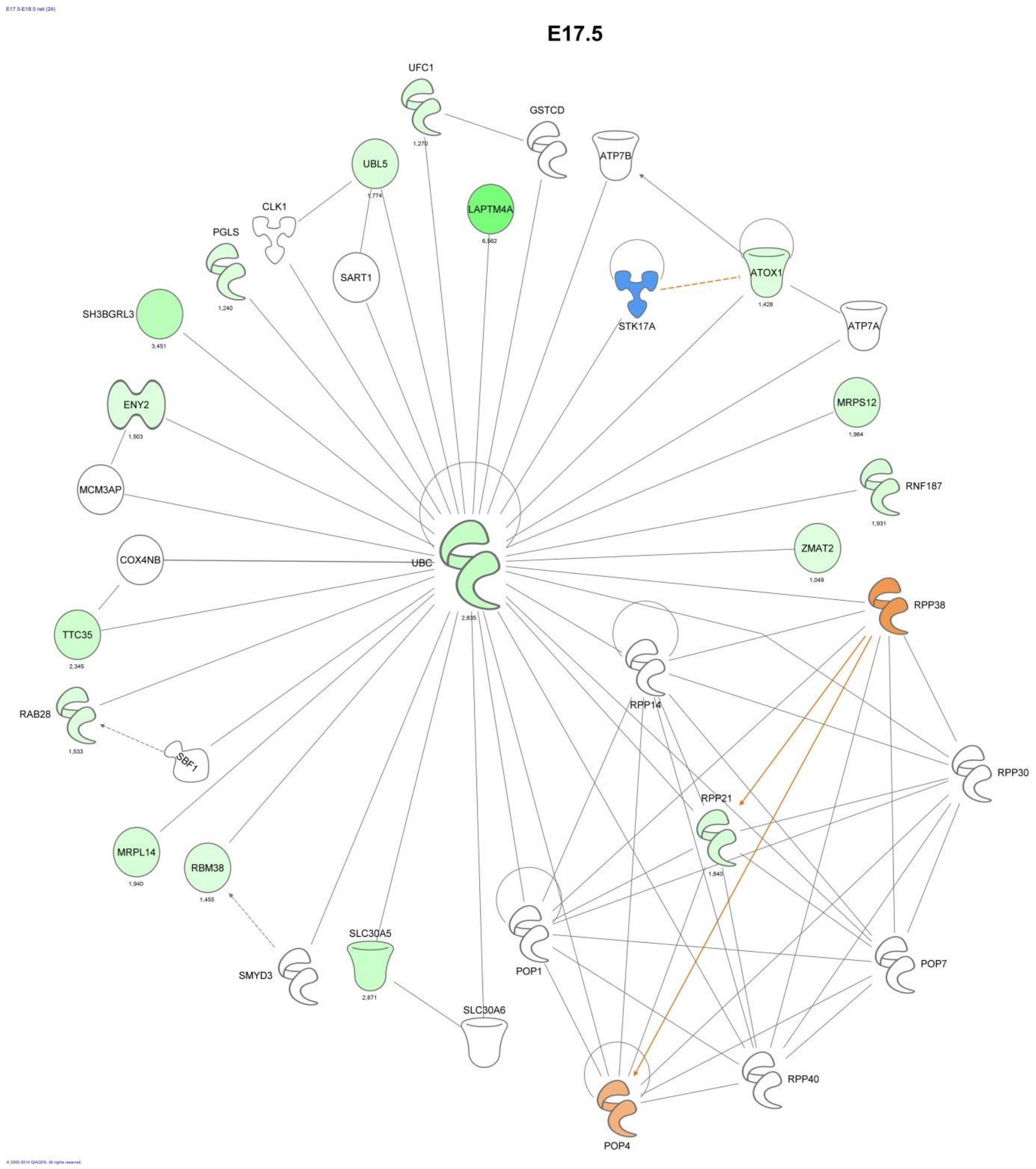

Network 18

Molecular transport, small molecule biochemistry, cellular function and maintenance

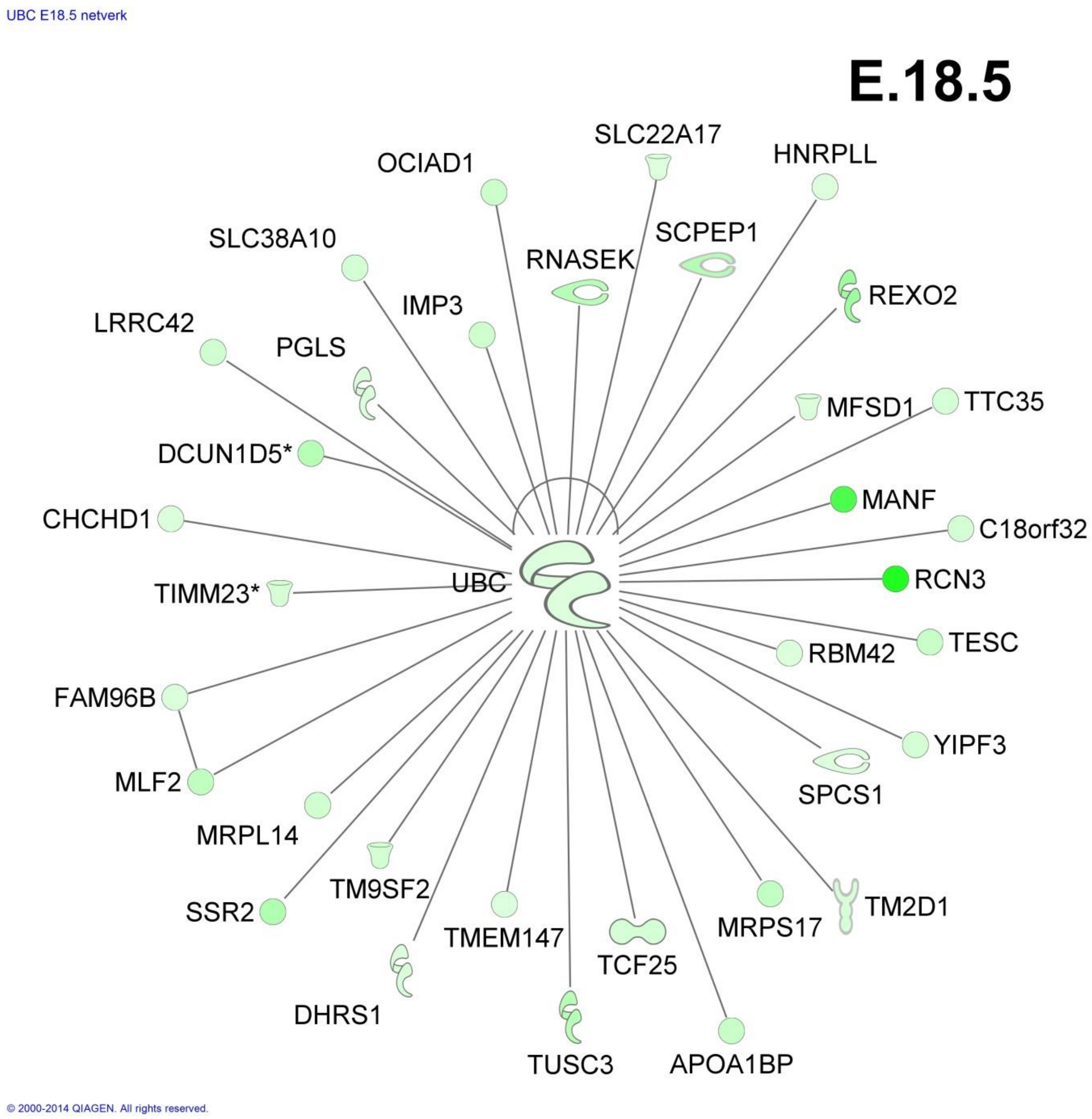

Network 1

Carbohydrate metabolism, nucleic acid metabolism, Senescence

E17.5-E18.5 net (6)

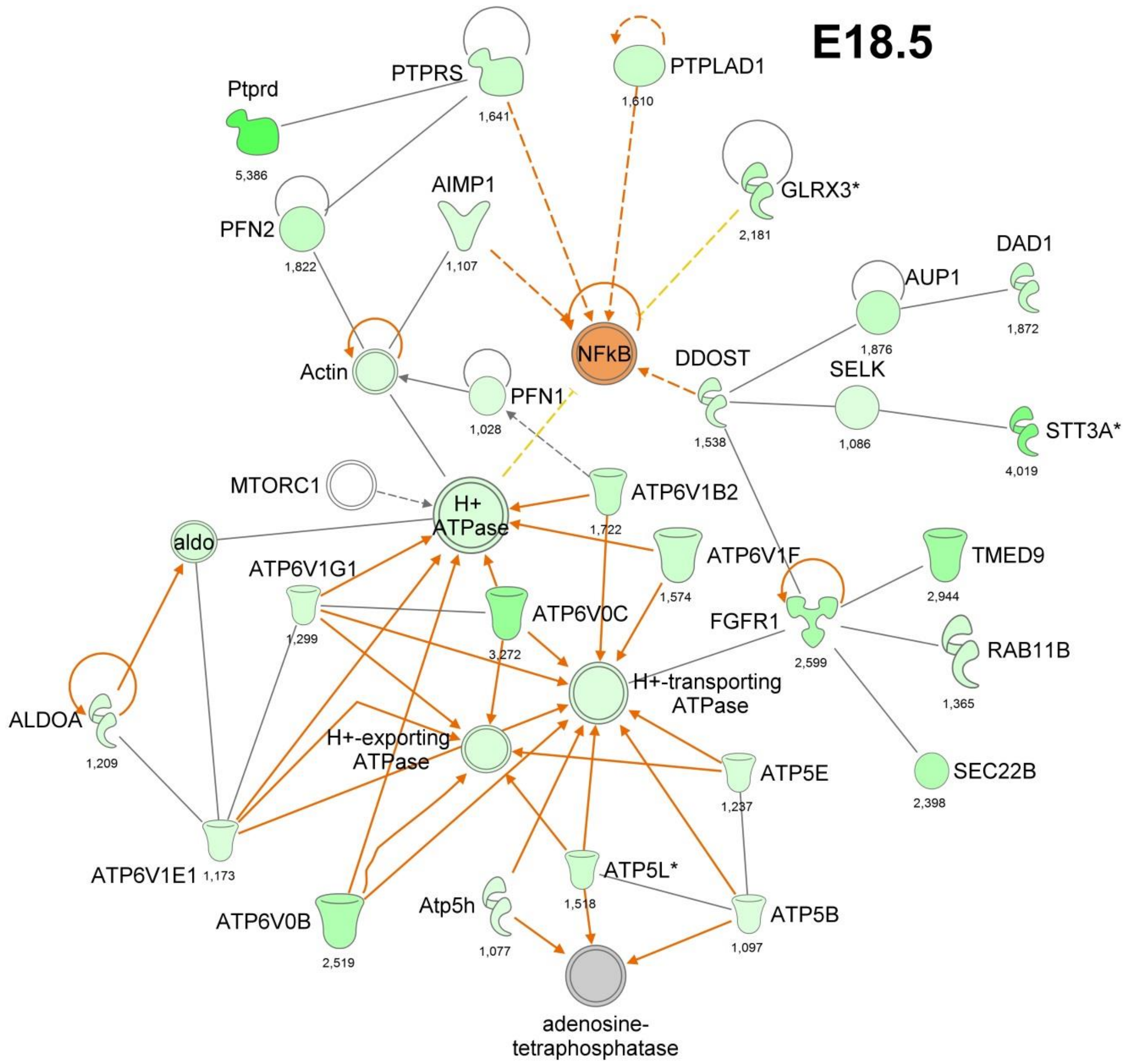

© 2000-2014 QIAGEN. All rights reserved.

Network 2

Molecular transport, cellular compromise

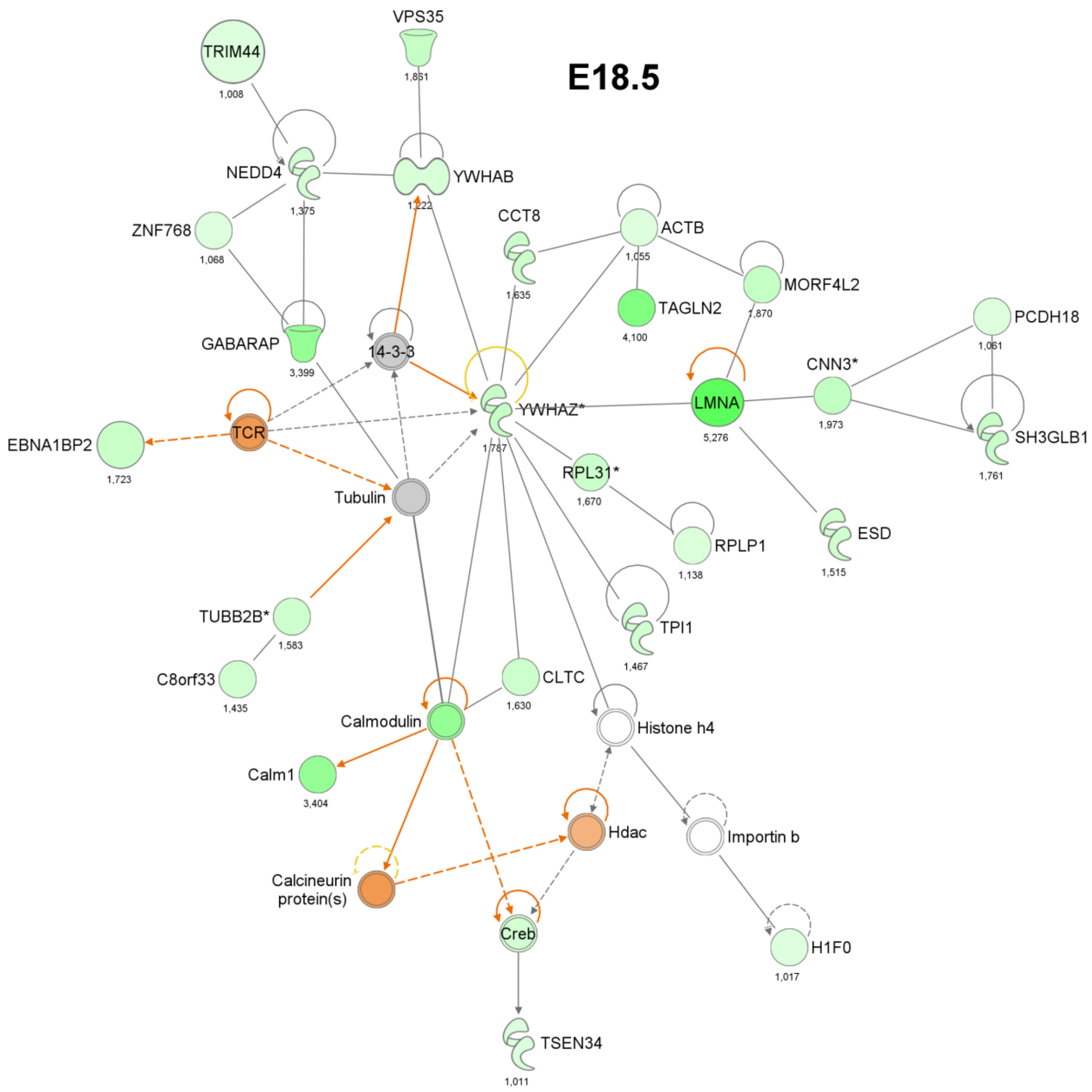

Network 3

Protein trafficking, cell morphology

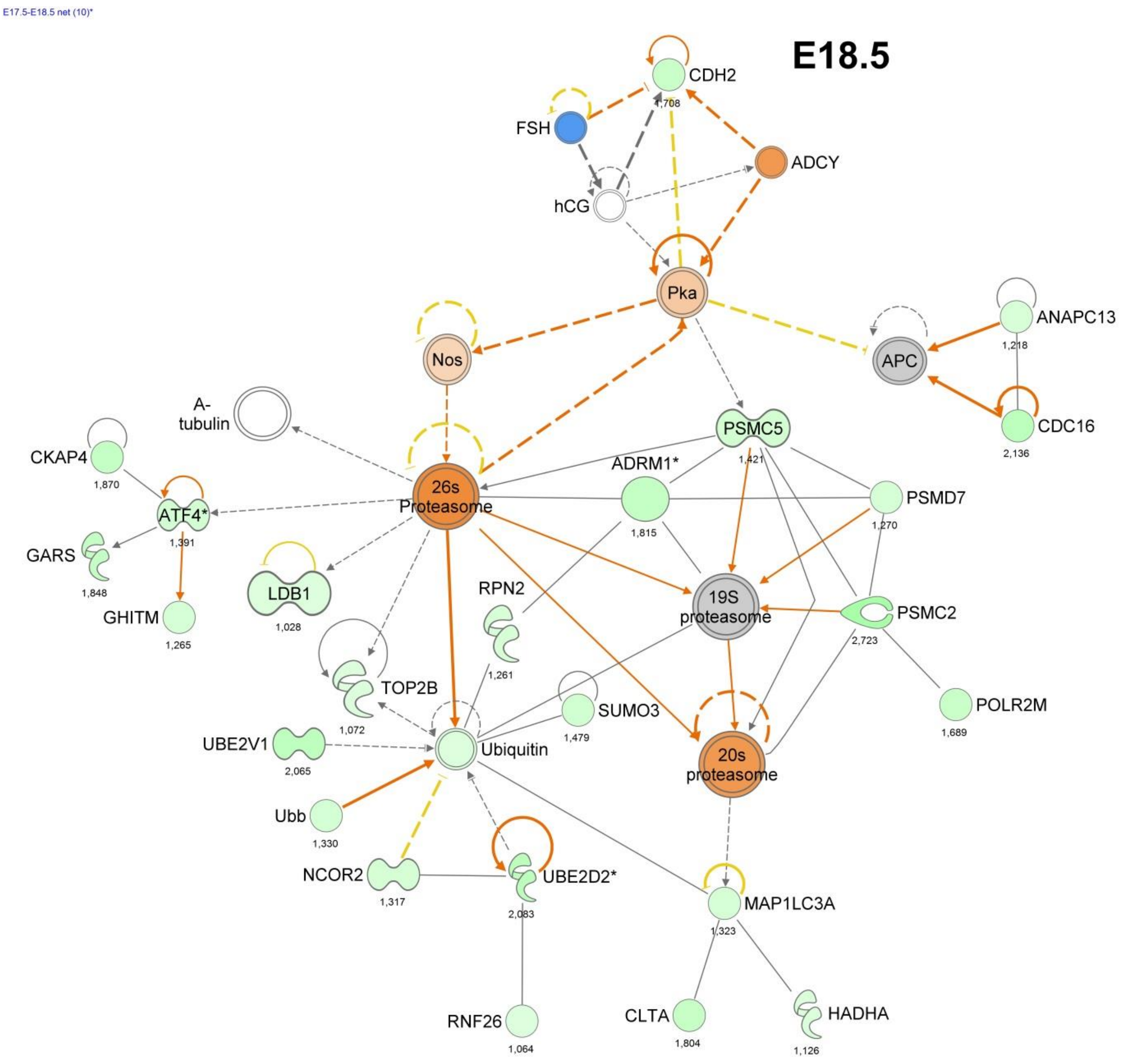

© 2000-2014 QIAGEN. All rights reserved.

Network 4

Organismal development

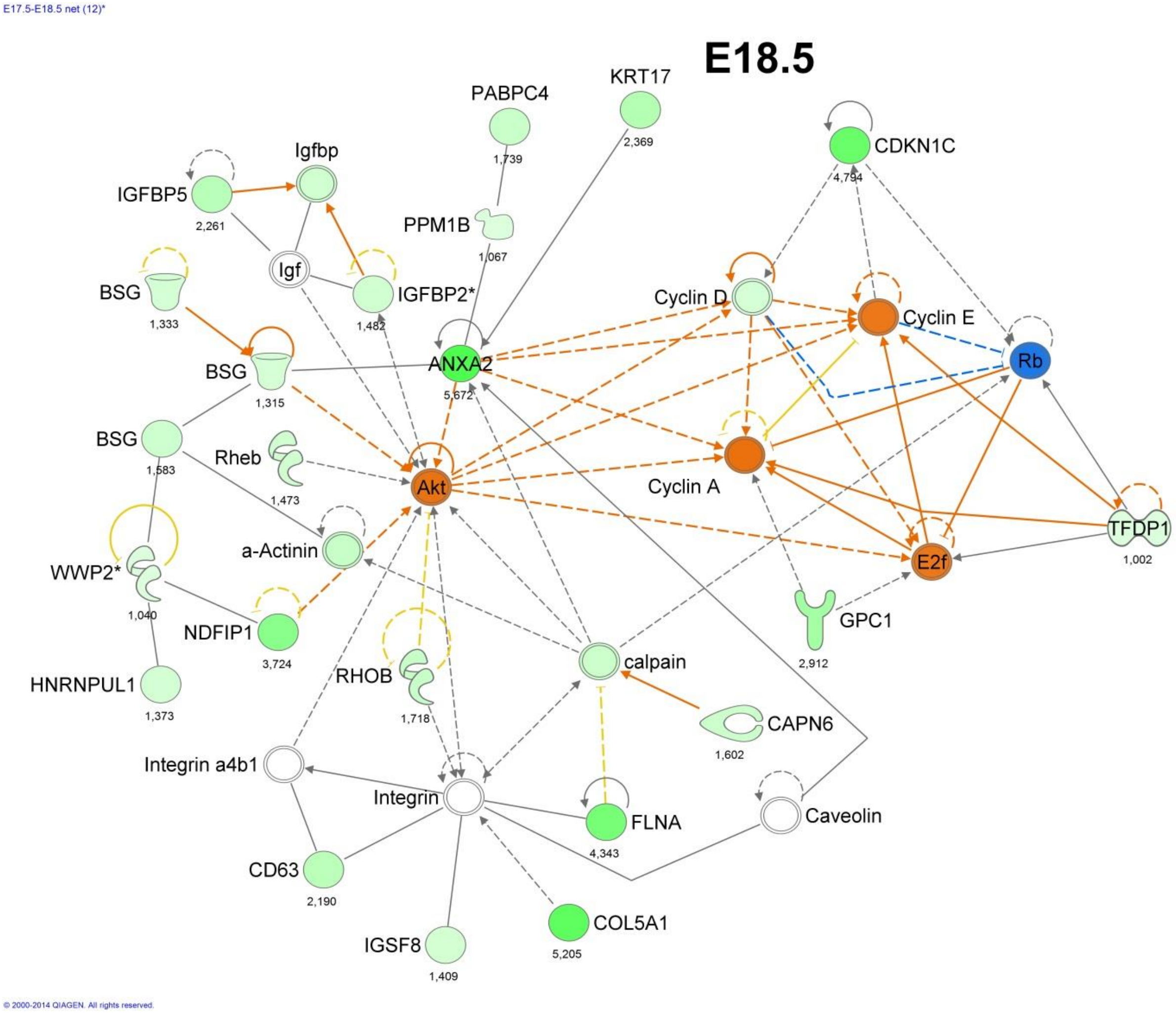

Network 5

Cellular assembly and organization, cellular function maintenance

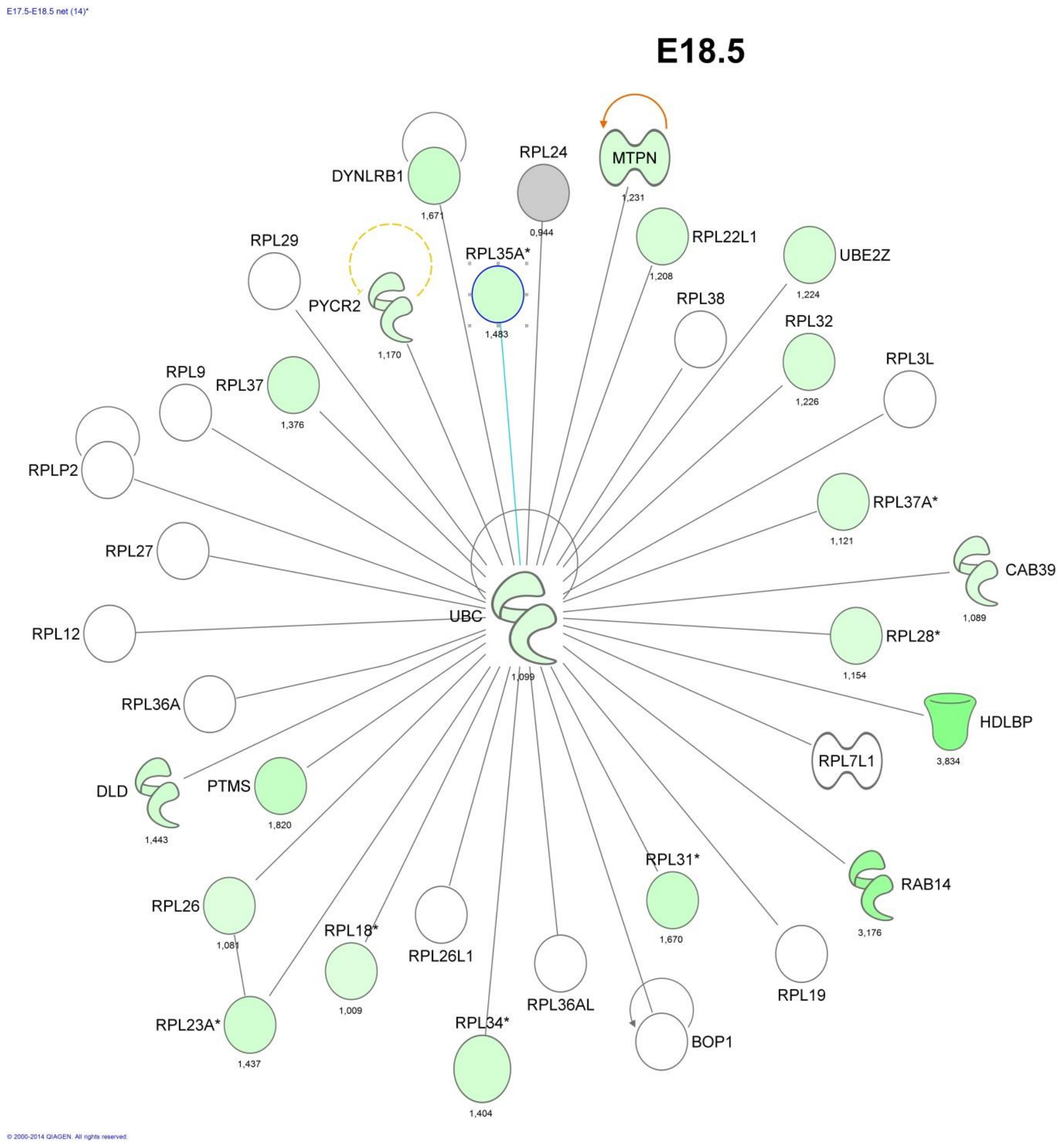

Network 6

RNA post-transcriptiona modification

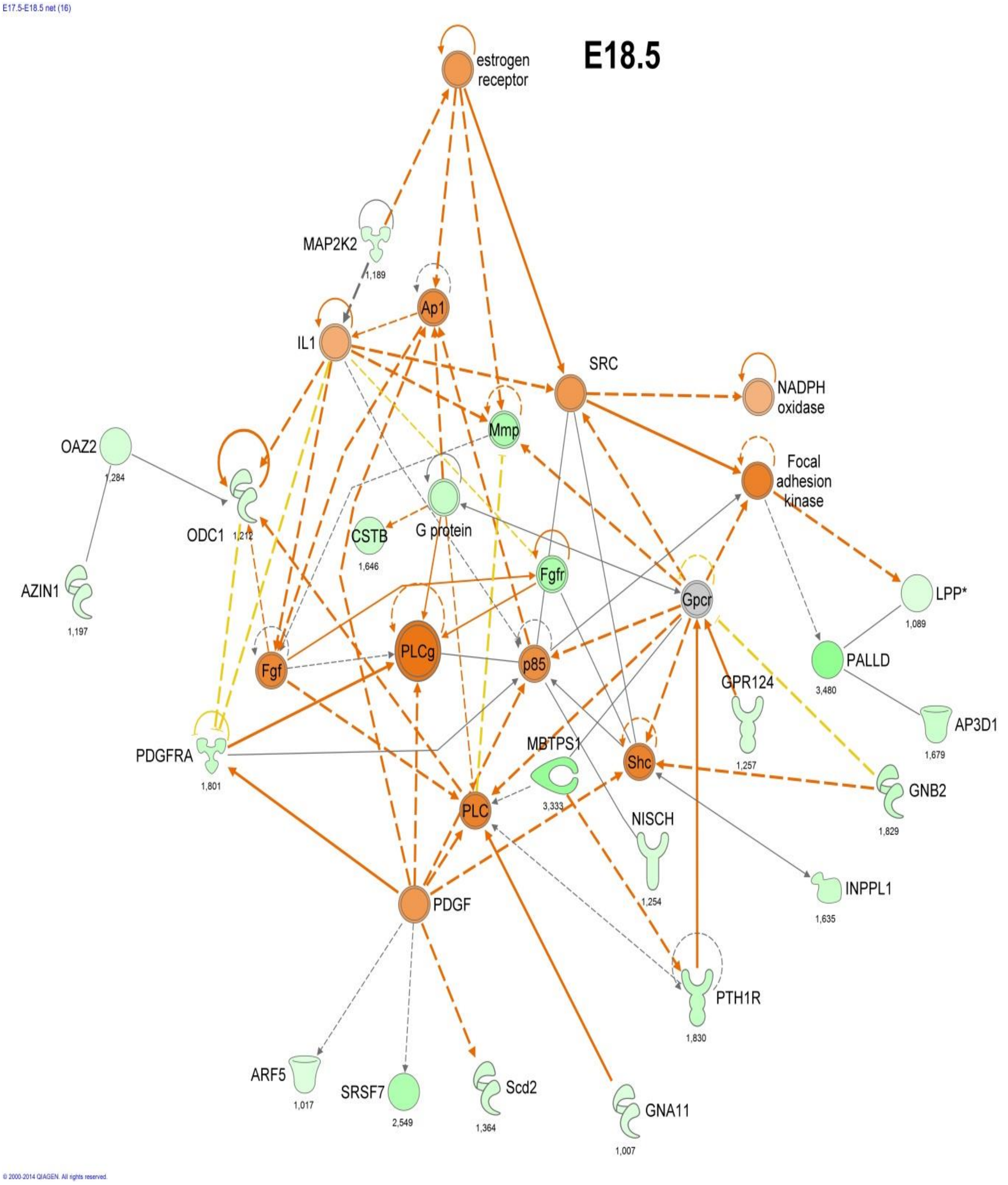

Network 7

Small molecule biochemistry, cellular movement, conective tissue development and function

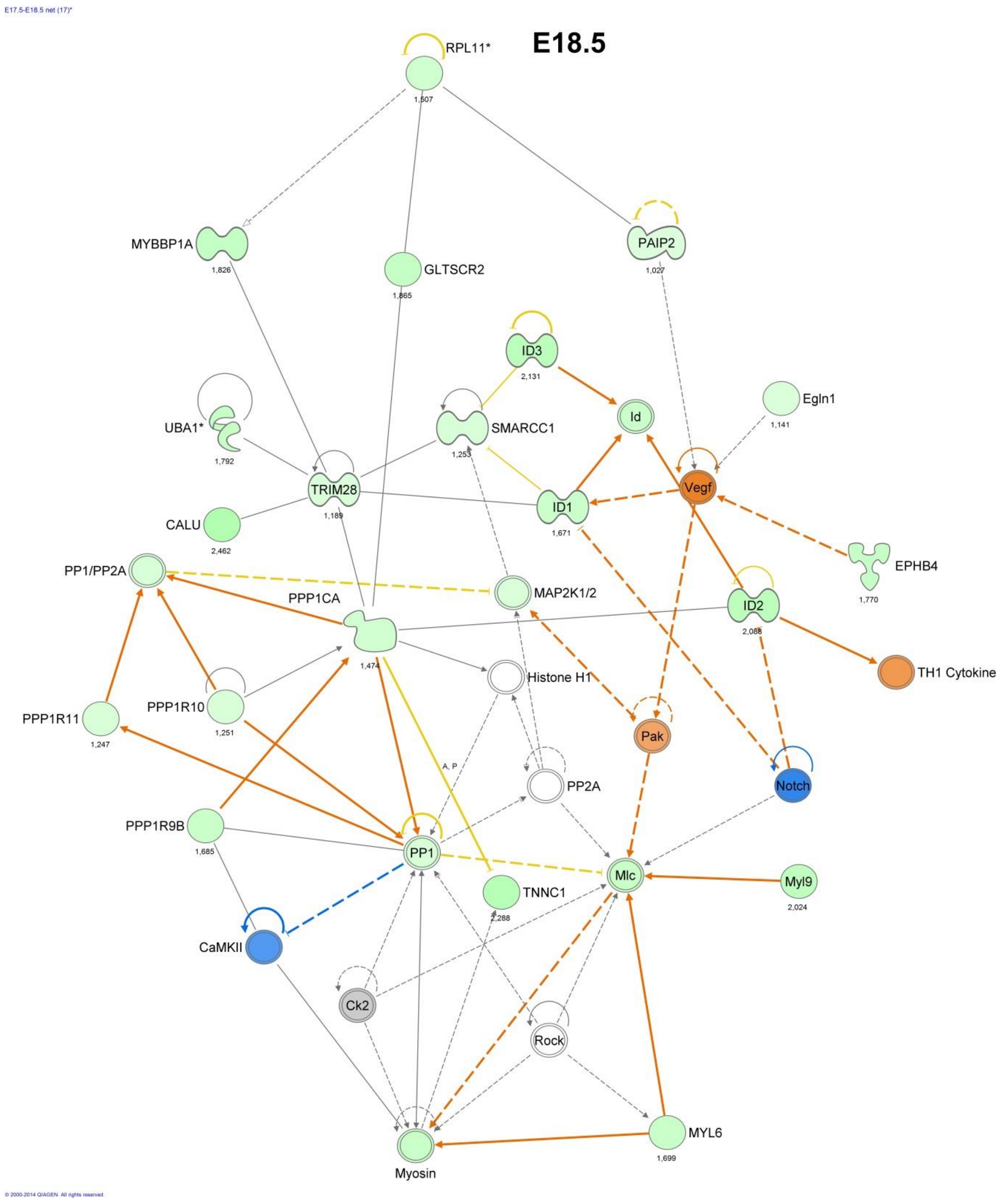

Network 8

Gene expression, cellular development, cellular growth and proliferation

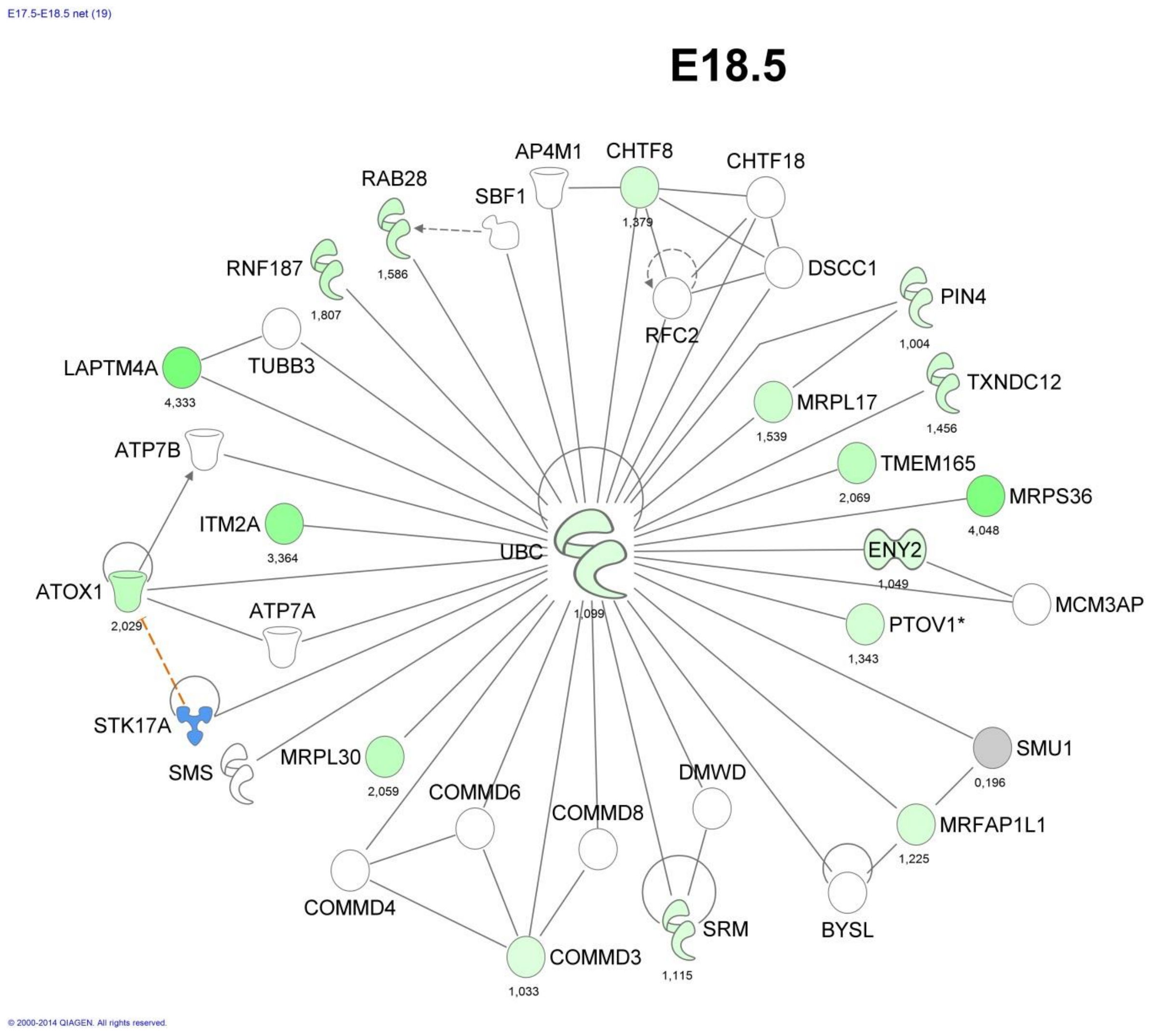

Network 9

Small molecule biochemistry, molecular transport, cellular function and maintenance

E17.5-E18.5 net (20)*

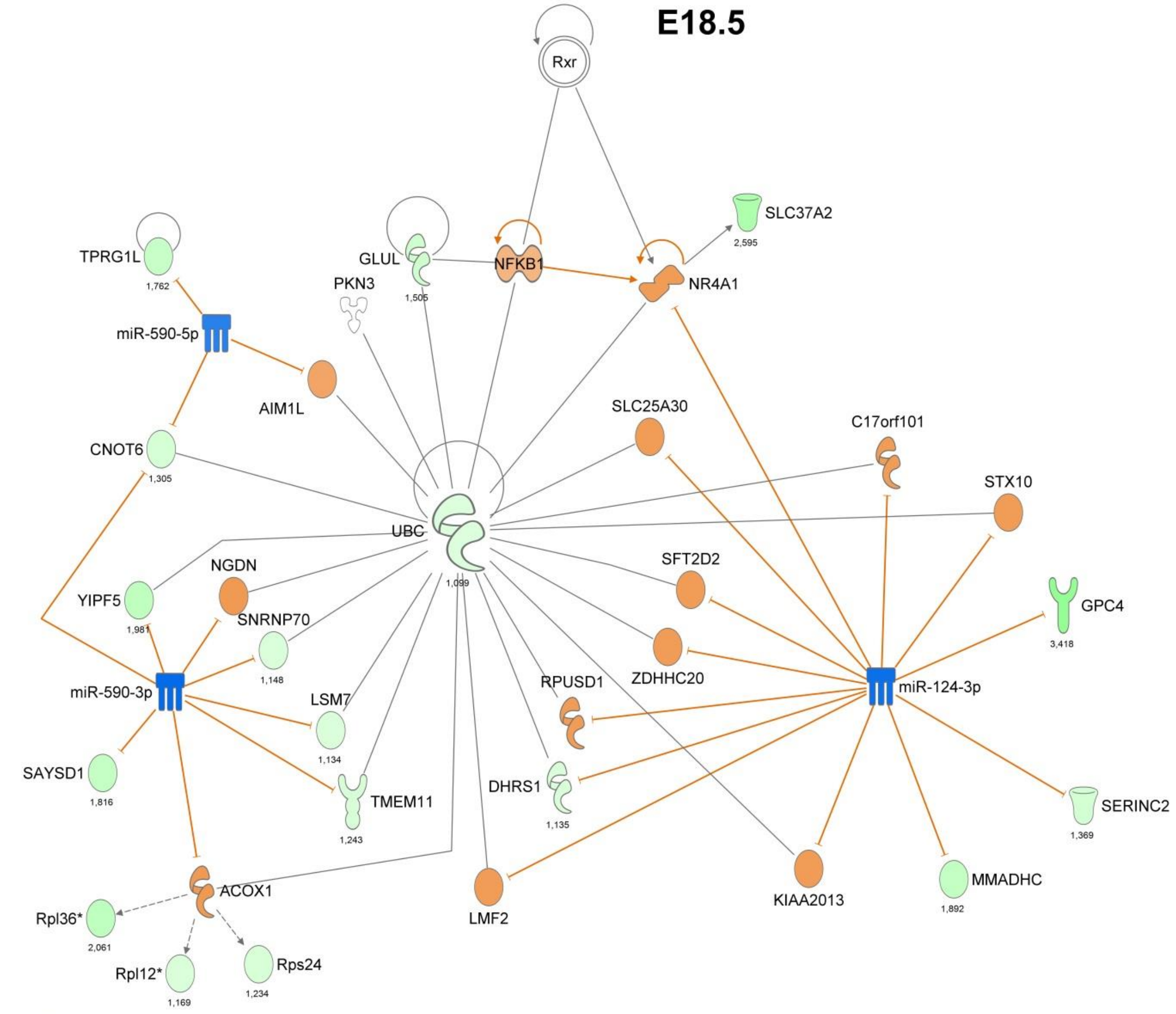

© 2000-2014 QIAGEN. All rights reserved.

Network 10

Post-translational modification, cellular function, Cellular assembly and organization

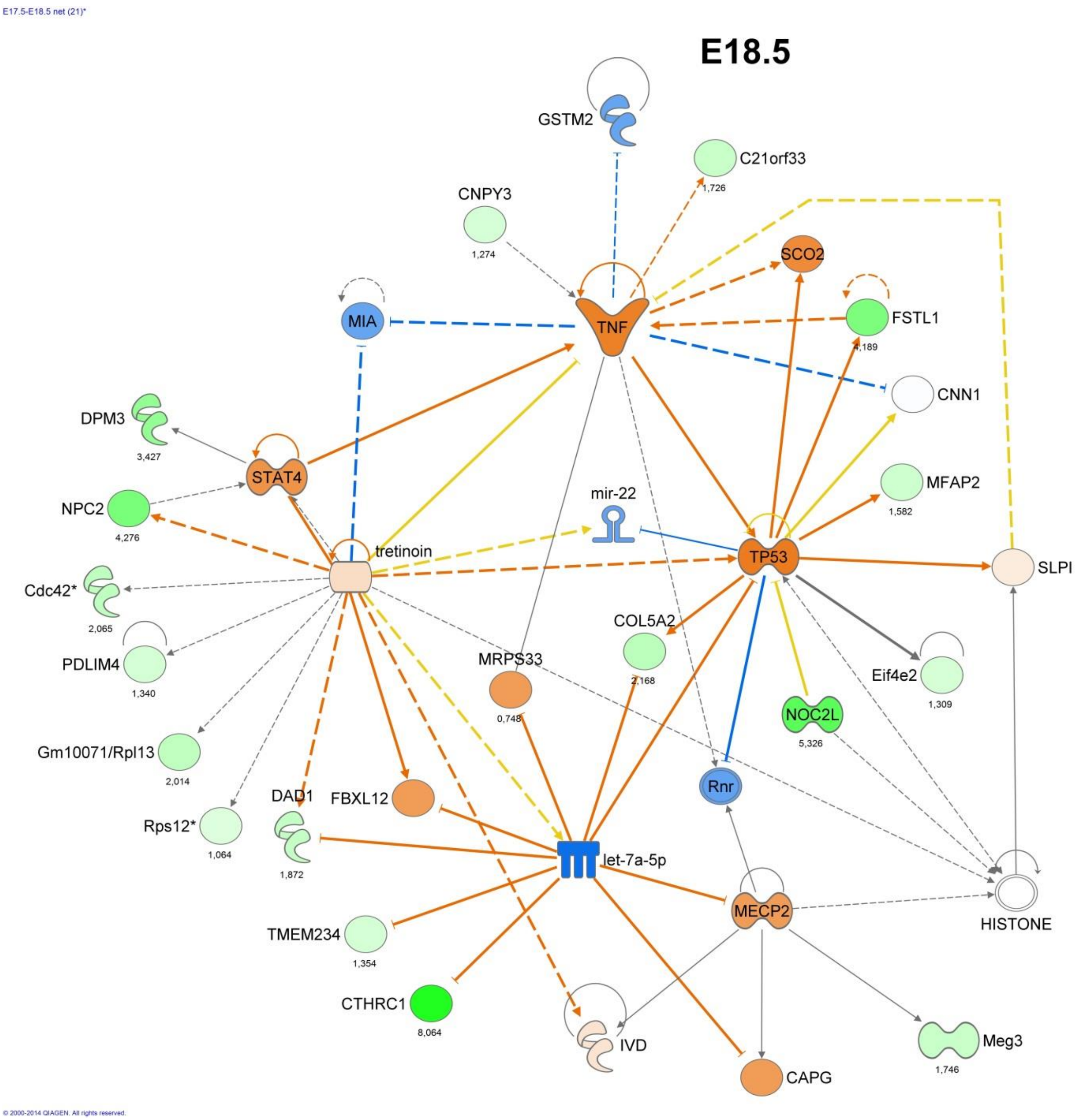

Network 11

Cell death, tissue morphology, connective tissue development and function

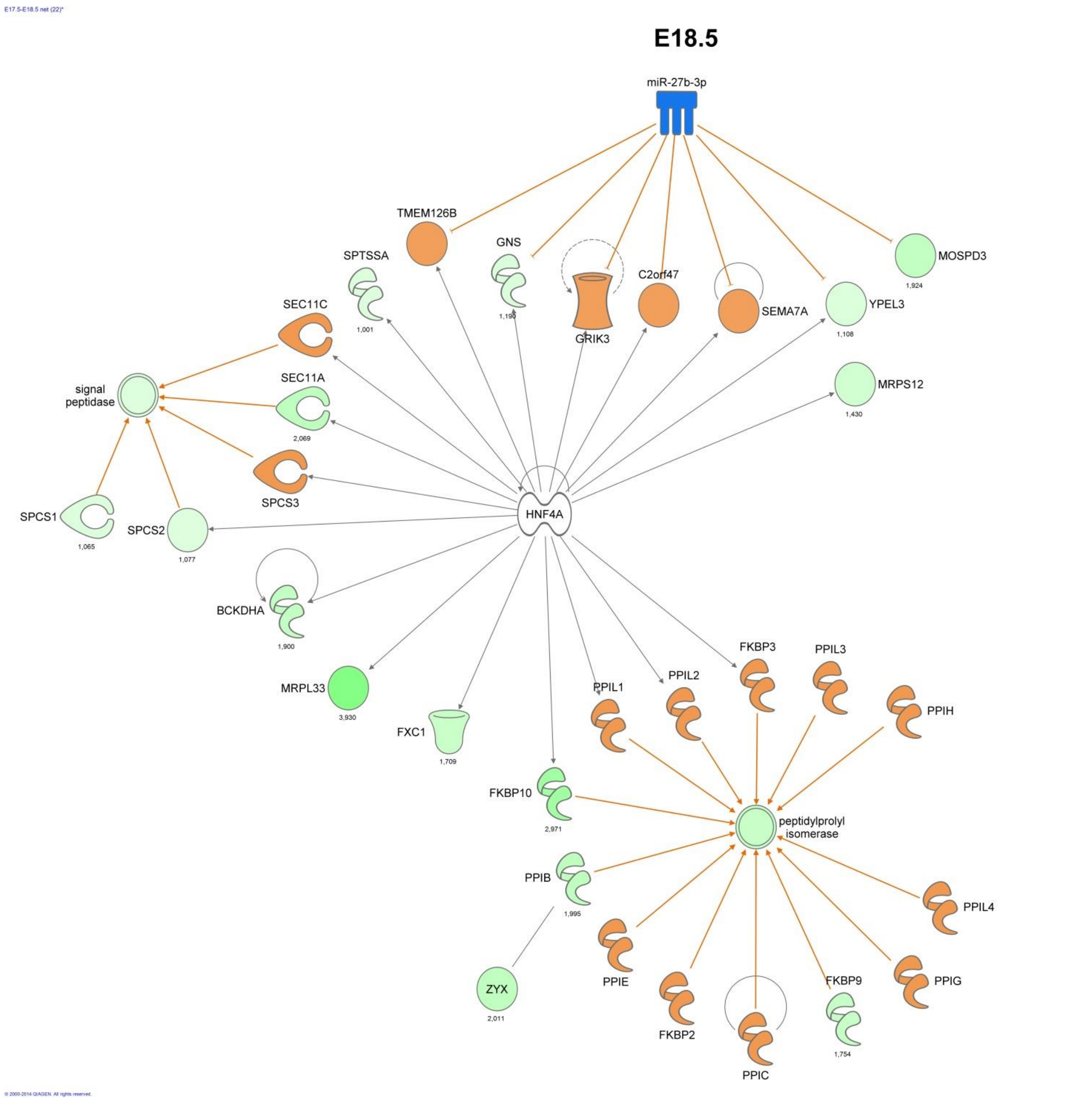

Network 12

Carbohydrate metabolism

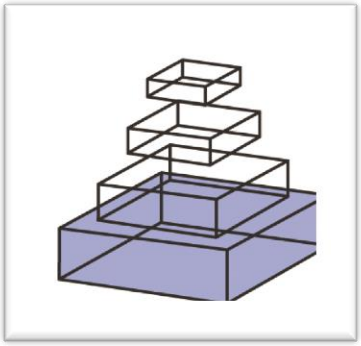

Supplement: Supplementary file 1 [file networkanalysisofpre-natalstages.PDF]
