# Supplementary material for: Mapping the global mRNA transcriptome during development of the murine first molar
Source: Front Genet. 2015 Feb 26;6:47. doi: 10.3389/fgene.2015.00047 (PMC4362327; doi:10.3389/fgene.2015.00047)

Supplementary Material

Mapping the global mRNA transcriptome during development of the murine first molar

Maria A. Landin[1,2], Ståle Nygård[4], Maziar Shabestari[2], Eshrat Babaie[3], Janne E. Reseland[2] and Harald Osmundsen[1]

[1]Department of Oral Biology, Faculty of Dentistry, University of Oslo, Norway

[2]Department of Biomaterials, Institute for Clinical Dentistry, University of Oslo, Norway

[3]The Biotechnology Centre of Oslo, University of Oslo, Norway

[4]Bioinformatics core facility, Institute for medical informatics, Oslo University Hospital and University of Oslo

*** Correspondence:** Maria A. dos Santos Silva Landin, Department of Oral Biology, Faculty of Dentistry,

University of Oslo, Norway

mariaal@odont.uio.no
dosantla@online.no

1. Supplementary Data

Detailed network analysis using IPA for each othe the 16-time-points studied was used as basic tool to try to interpret the complex events occurring during murine tooth development. Due to huge amount of networks generated for each time-point we present the resulting networks as supplementary data after birth are presented as follows: 4) post-natal stages (P0-P7). Results from network analysis are presented with the respective predicted activation/ inhibition state on the molecules/genes of the dataset and on other molecules e.g. miRNA molecules.

2. Supplementary Figures

Legends to supplementary data

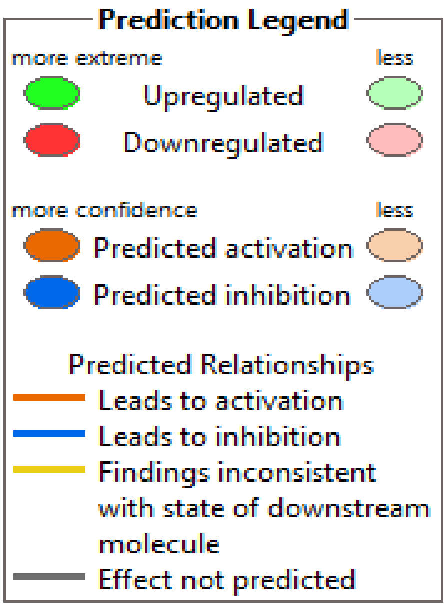

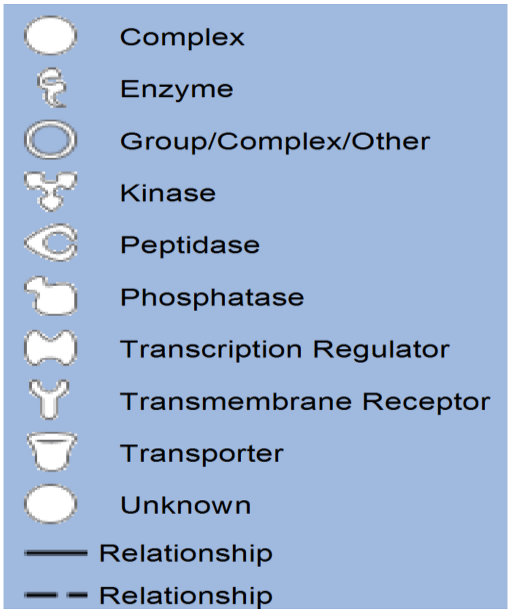

Supplementary Figure 4. **Networks associated with genes expressed at post-natal stages**

P0-P1 net (1)

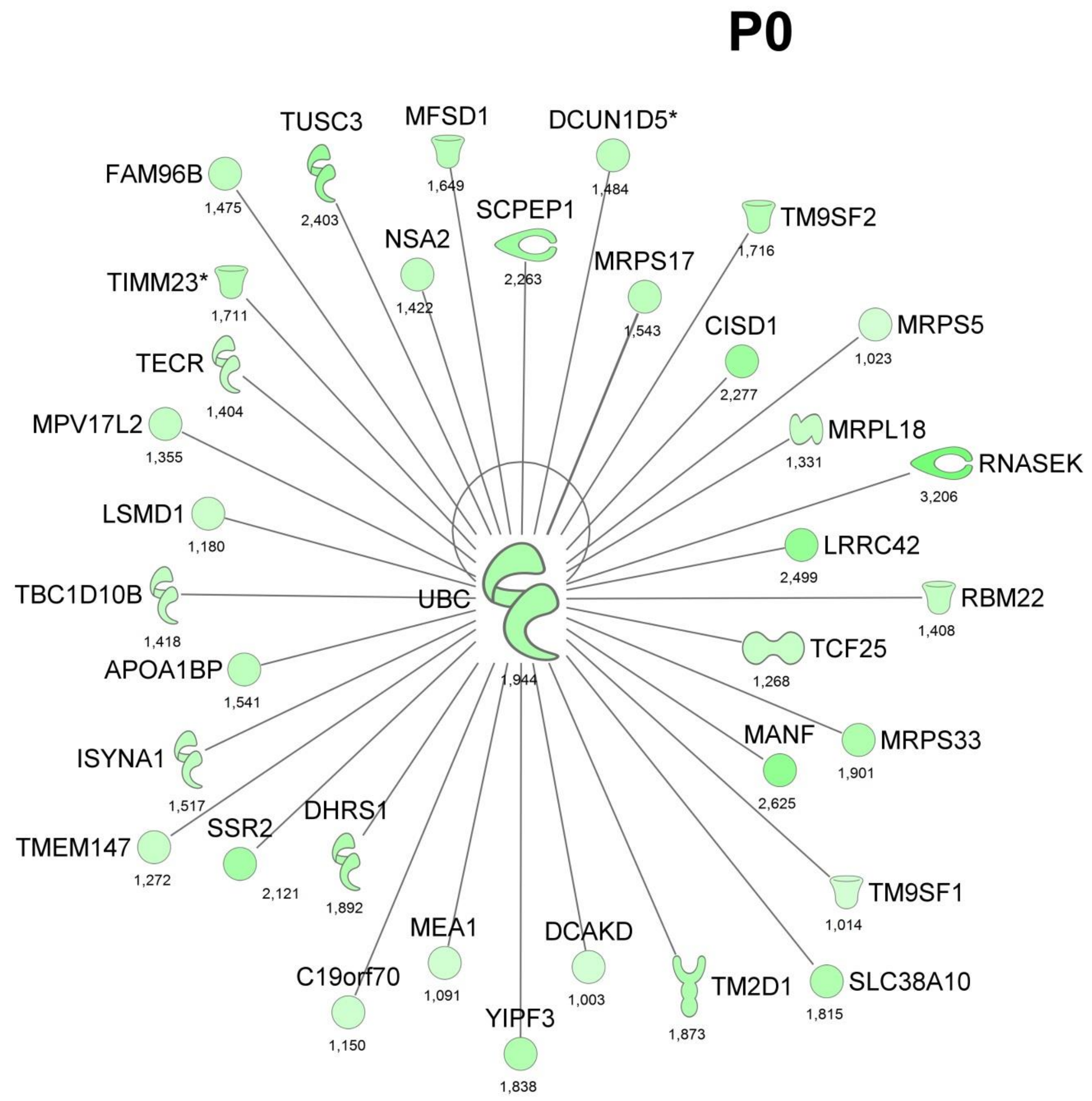

© 2000-2014 QIAGEN. All rights reserved.

Network 1

Lipid metabolism, nucleic acid metabolism, small molecule biochemistry

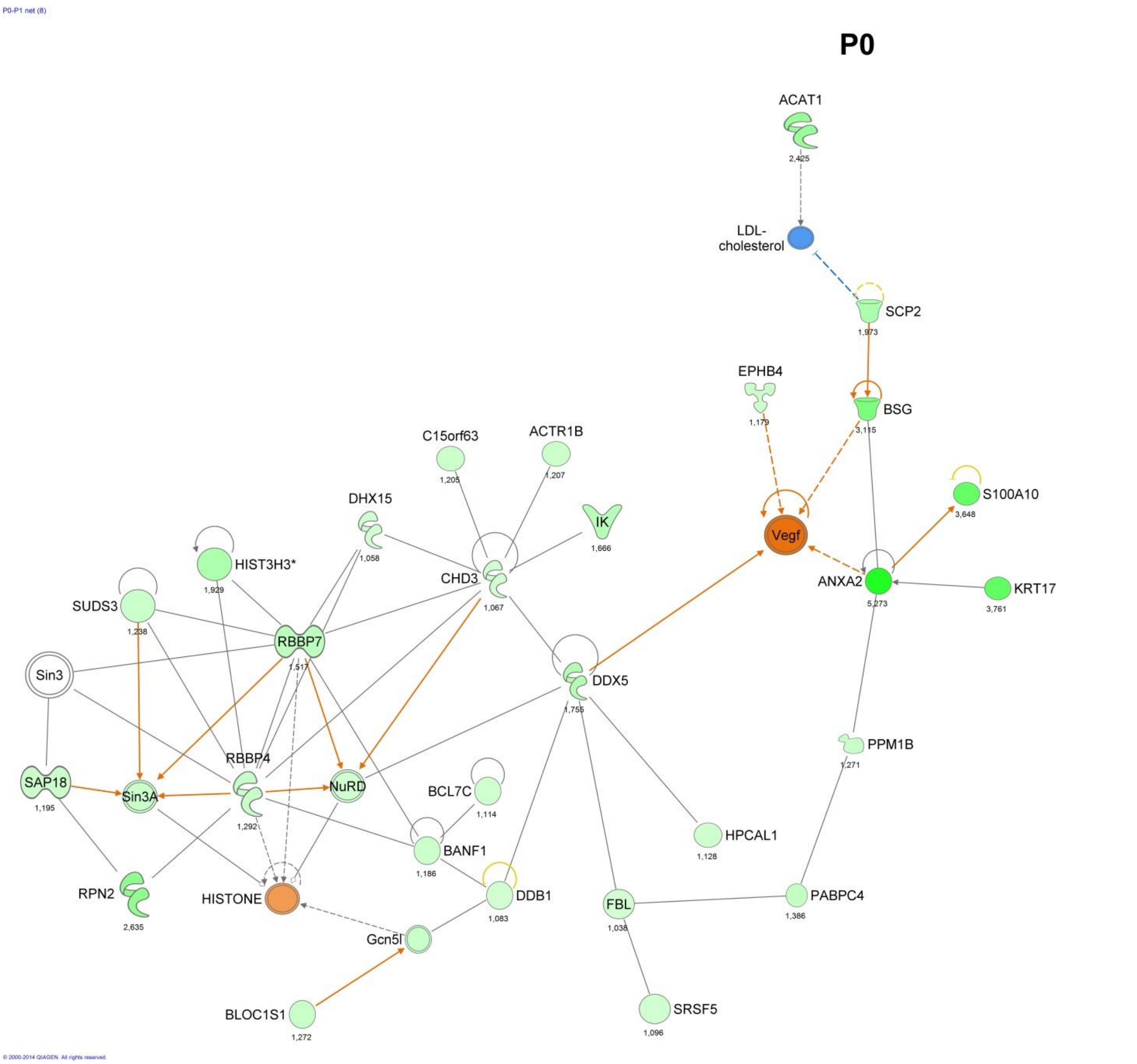

Network 2

Lipid metabolism, molecular transport, small molecule biochemistry

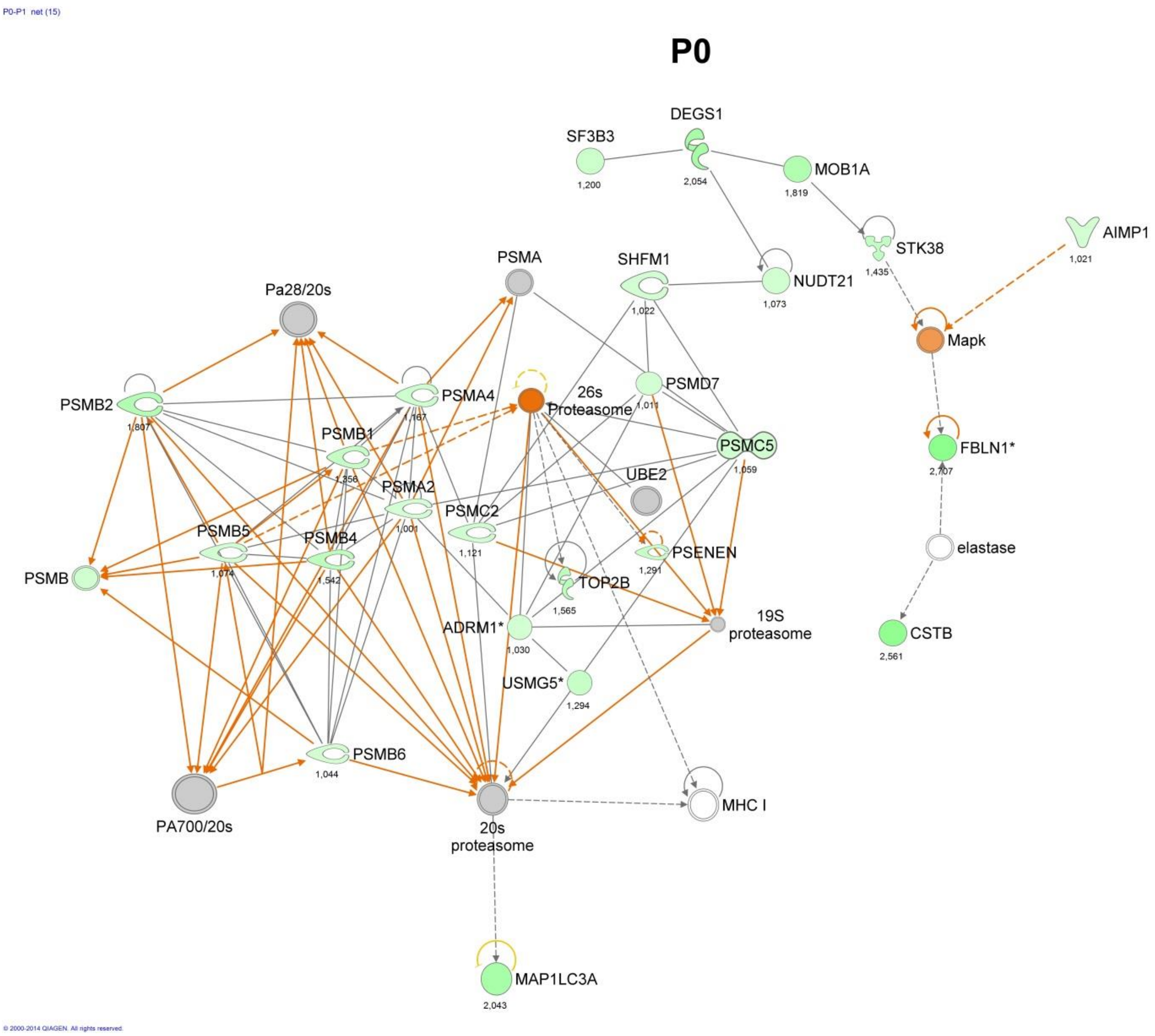

Network 3

Cell cycle, embryonic development, tissue morphology

P0-P1 net (2)

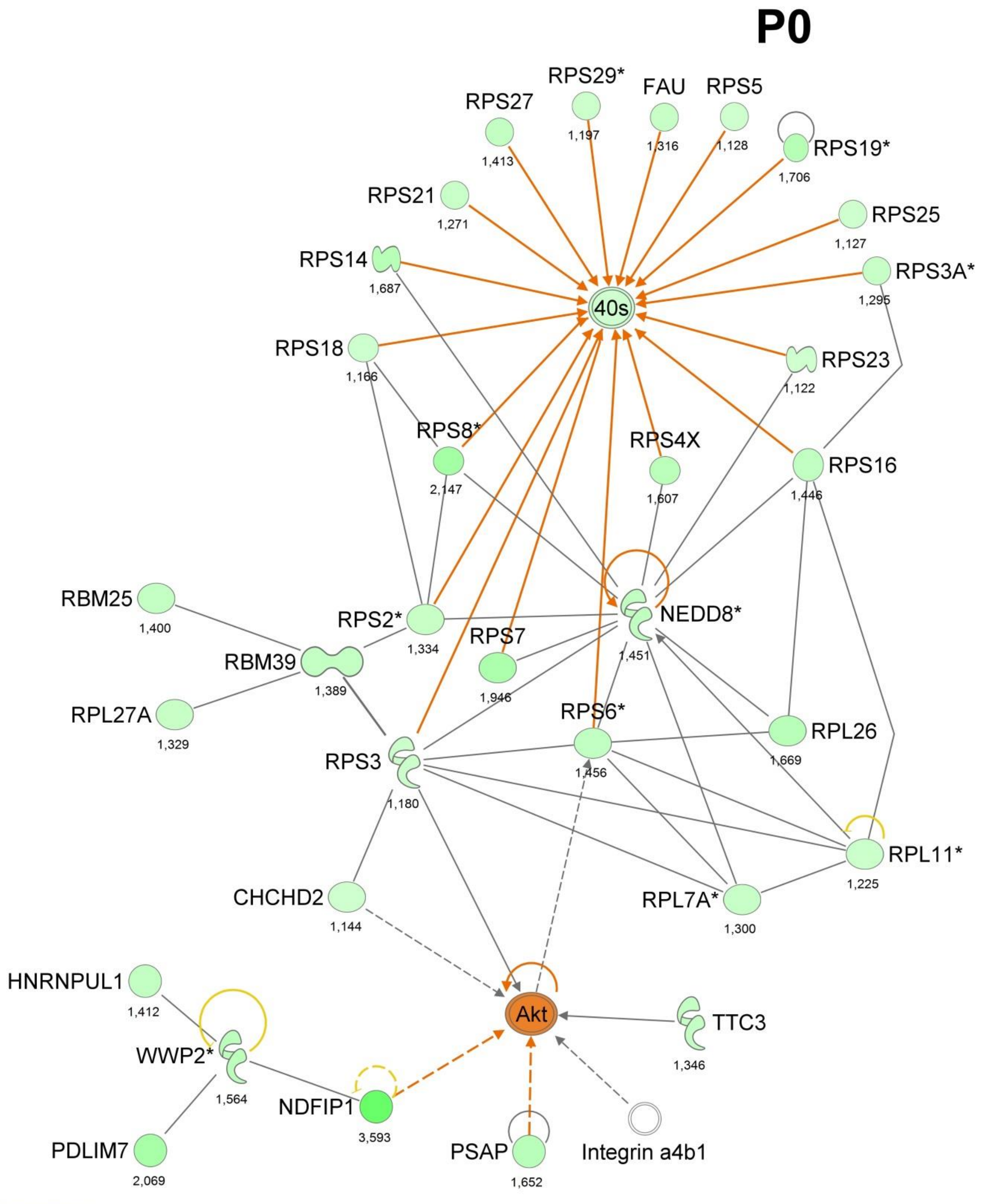

© 2000-2014 QIAGEN. All rights reserved.

Network 4

RNA post-transcriptional modification, protein synthesis, gene expression

P0-P1 net (3)

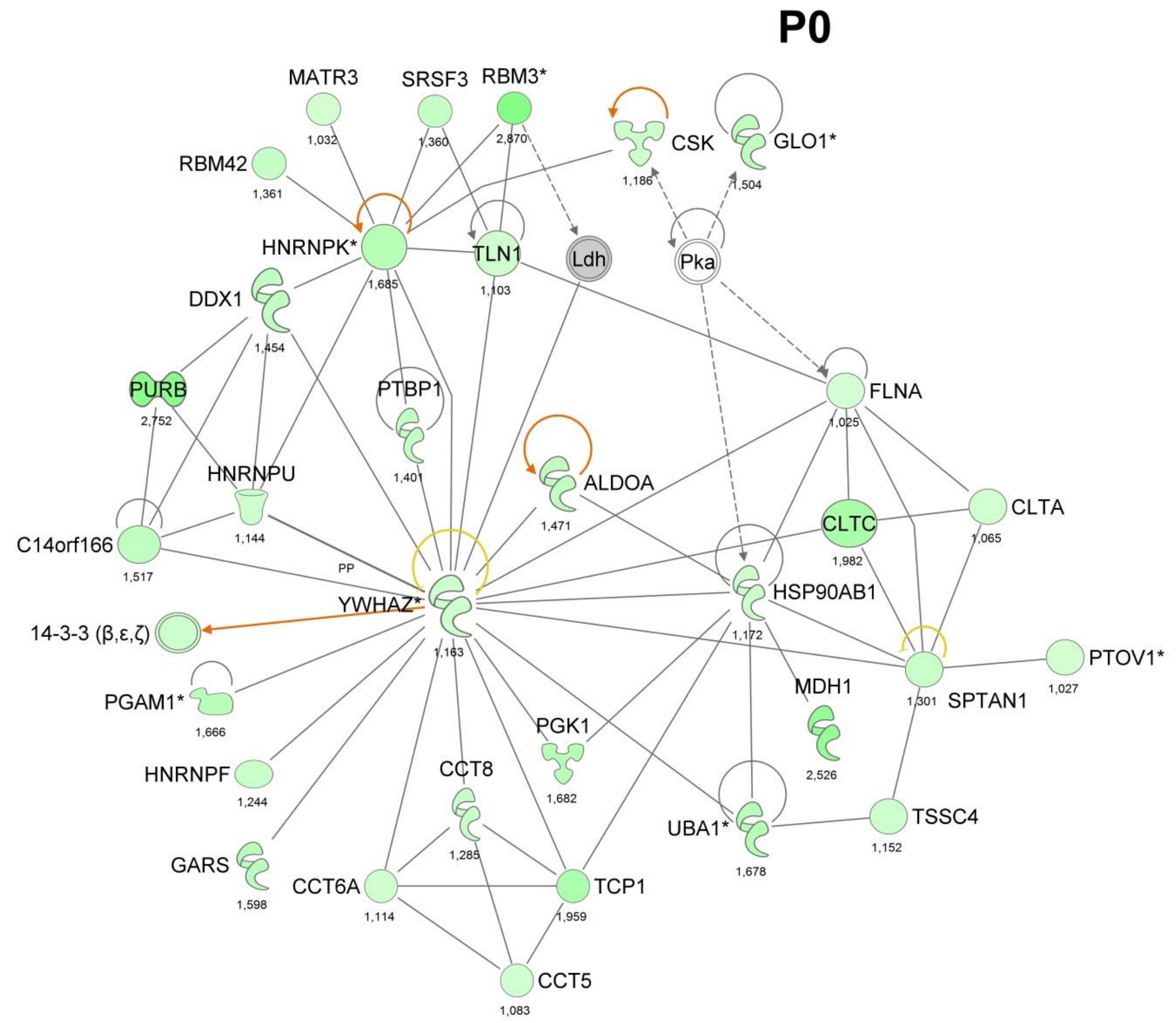

© 2000-2014 QIAGEN. All rights reserved.

Network 5

RNA post-transcriptional modification

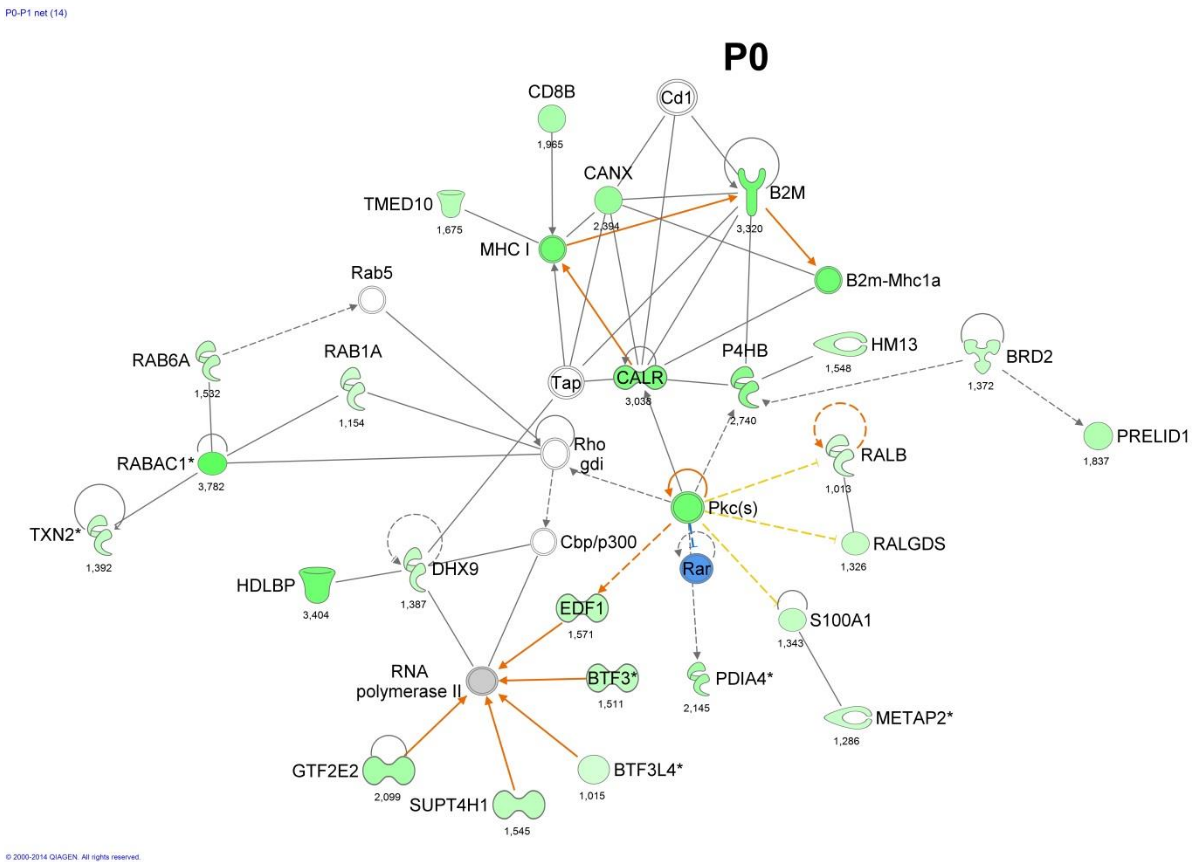

Network 6

RNA post-transcriptional modification, protein folding

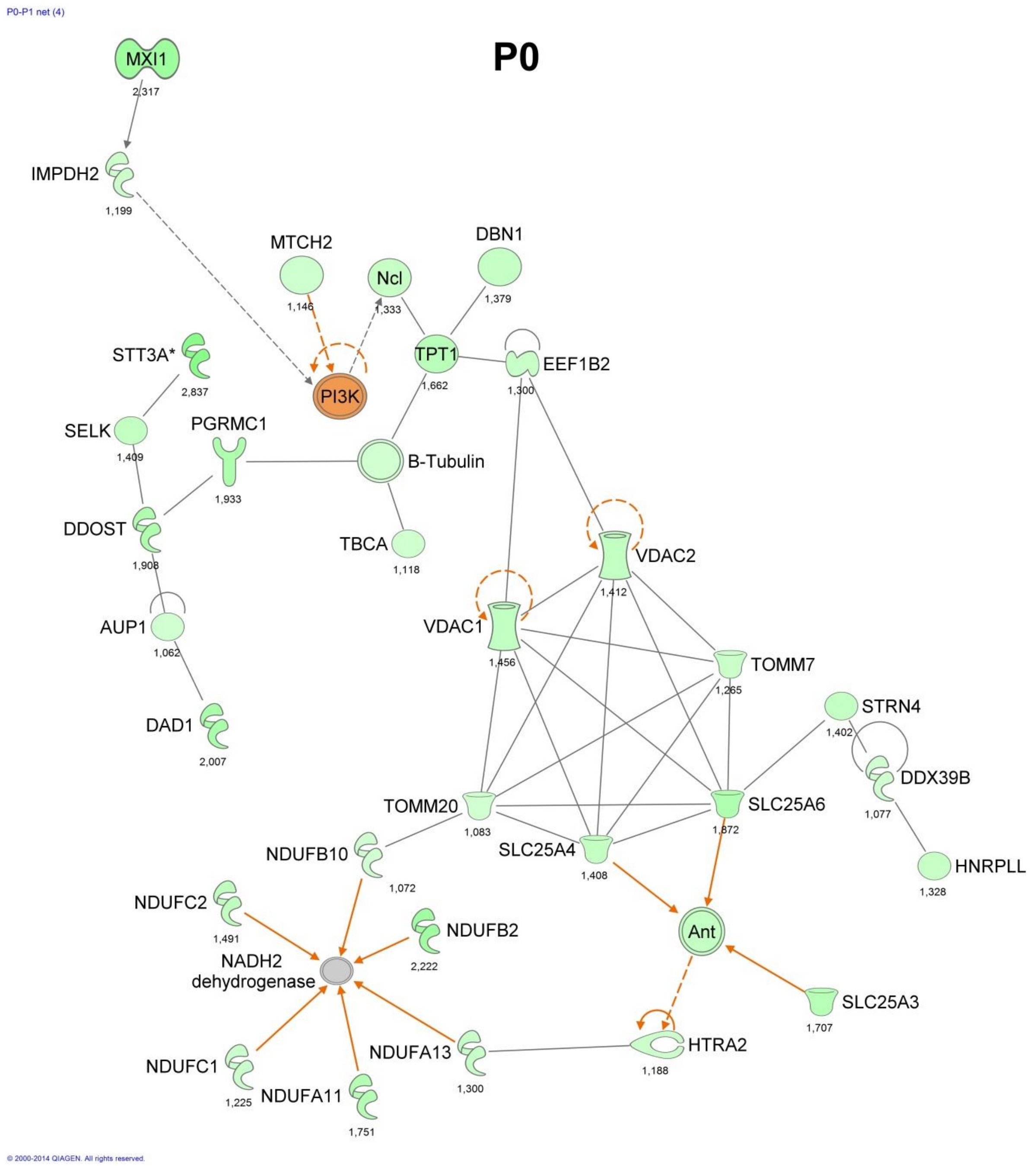

Network 7

Cellular assembly and organization, molecular transport, nucleic acid metabolism

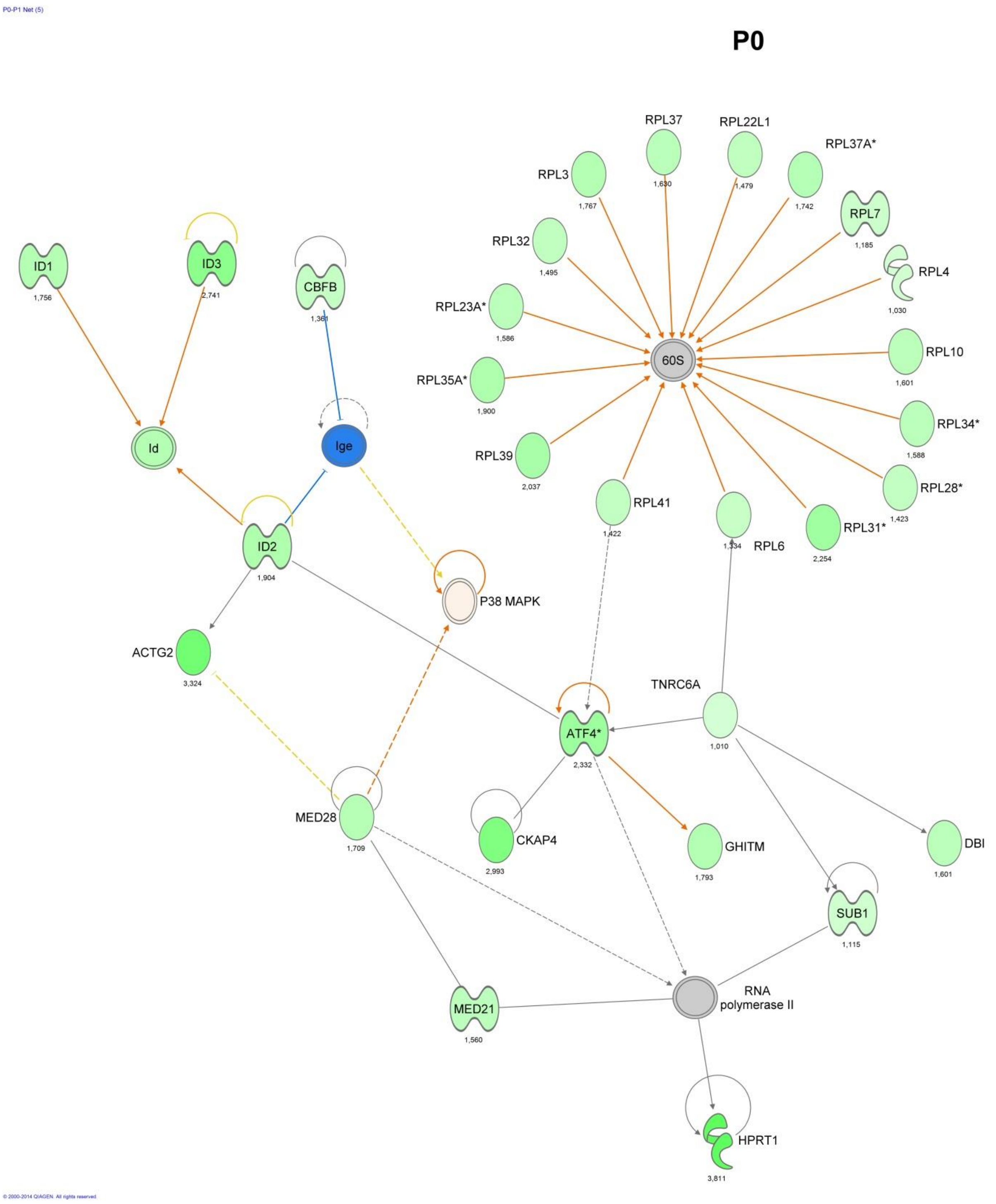

Network 8

Gene expression, cellular development, cellular growth and proliferation

P0-P1 net (9)

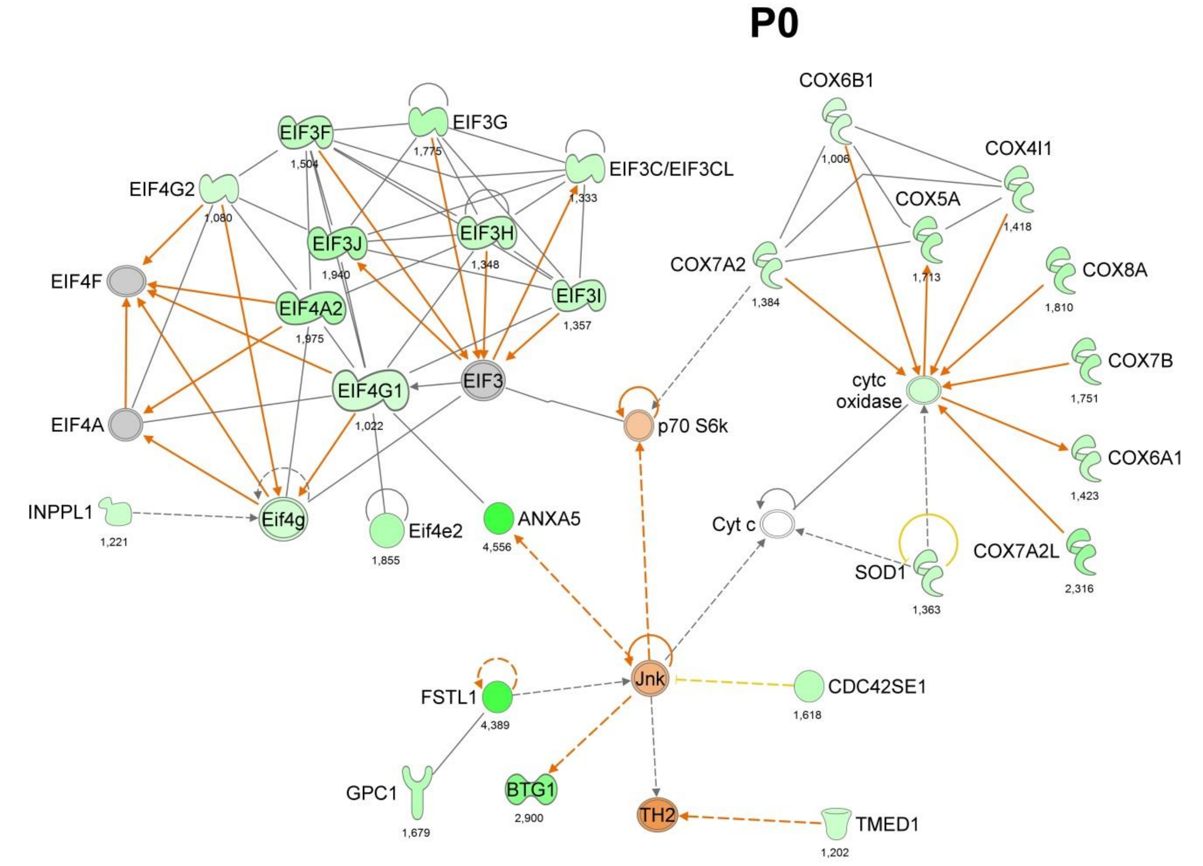

© 2000-2014 QIAGEN. All rights reserved.

Network 9

Gene expression, protein synthesis, carbohydrate metabolism

P0-P1 net (19)

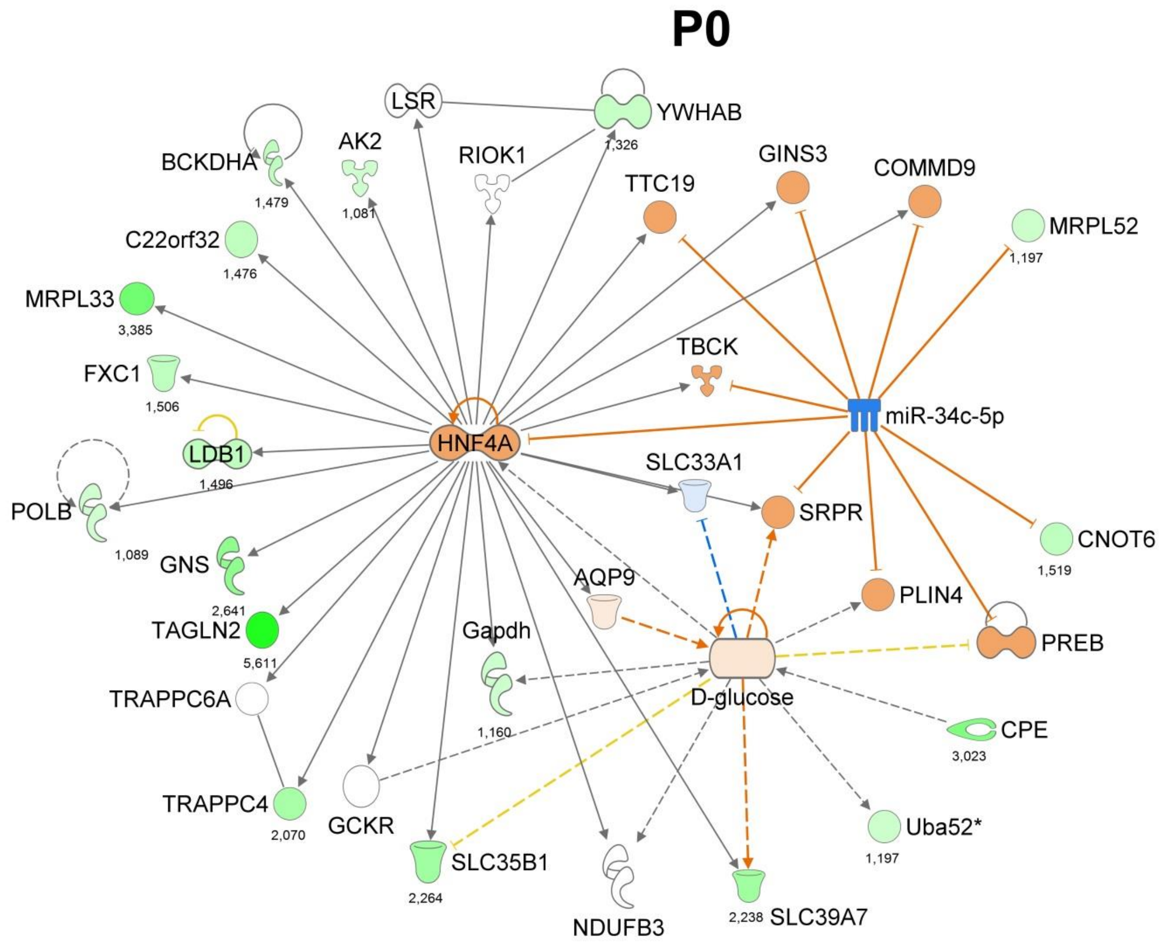

© 2000-2014 QIAGEN. All rights reserved.

Network 10

Carbohydrate metabolism, molecular transport, small molecule biochemistry

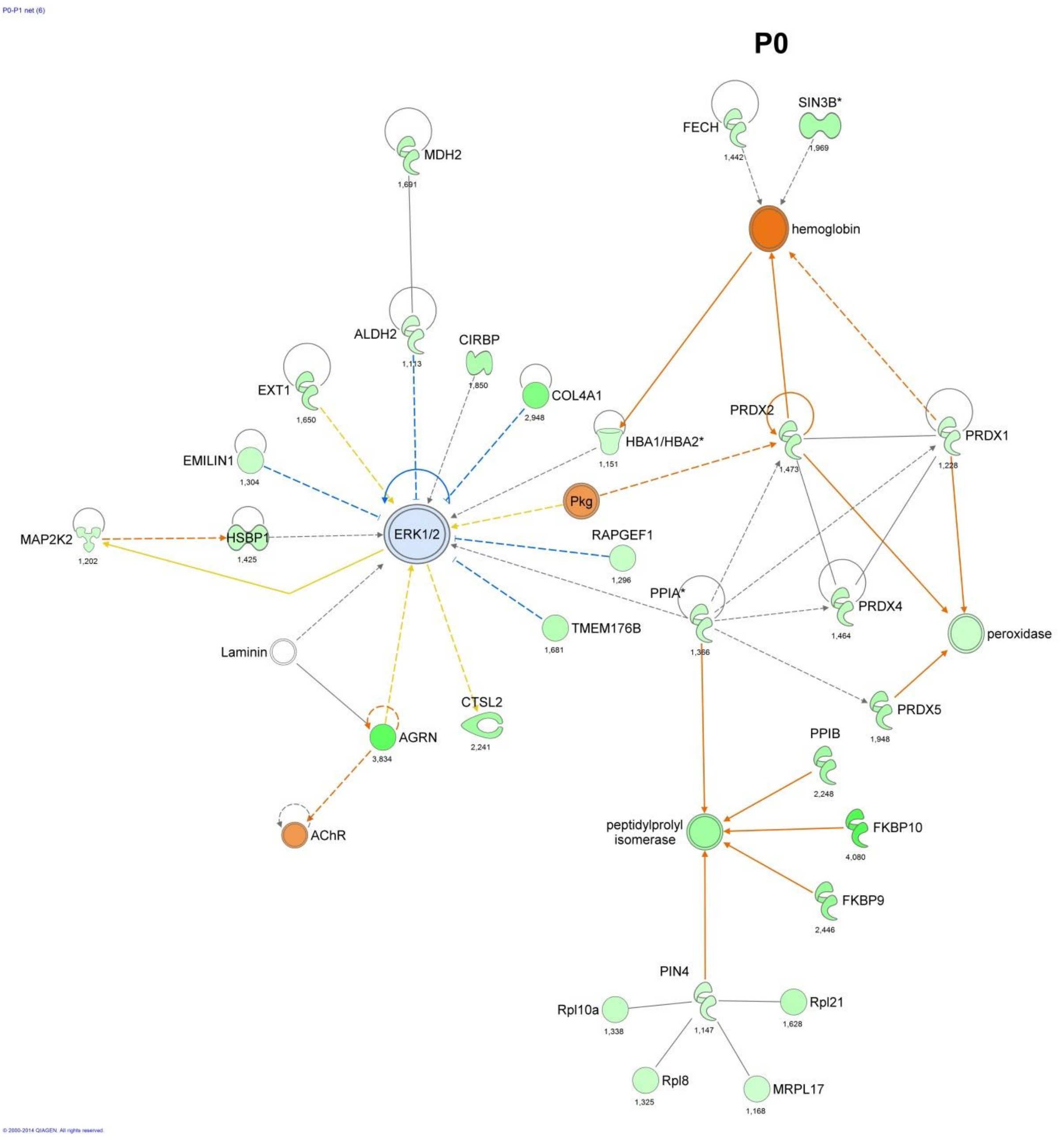

Network 11

Free radical scavenging, small molecule biochemistry, cell death

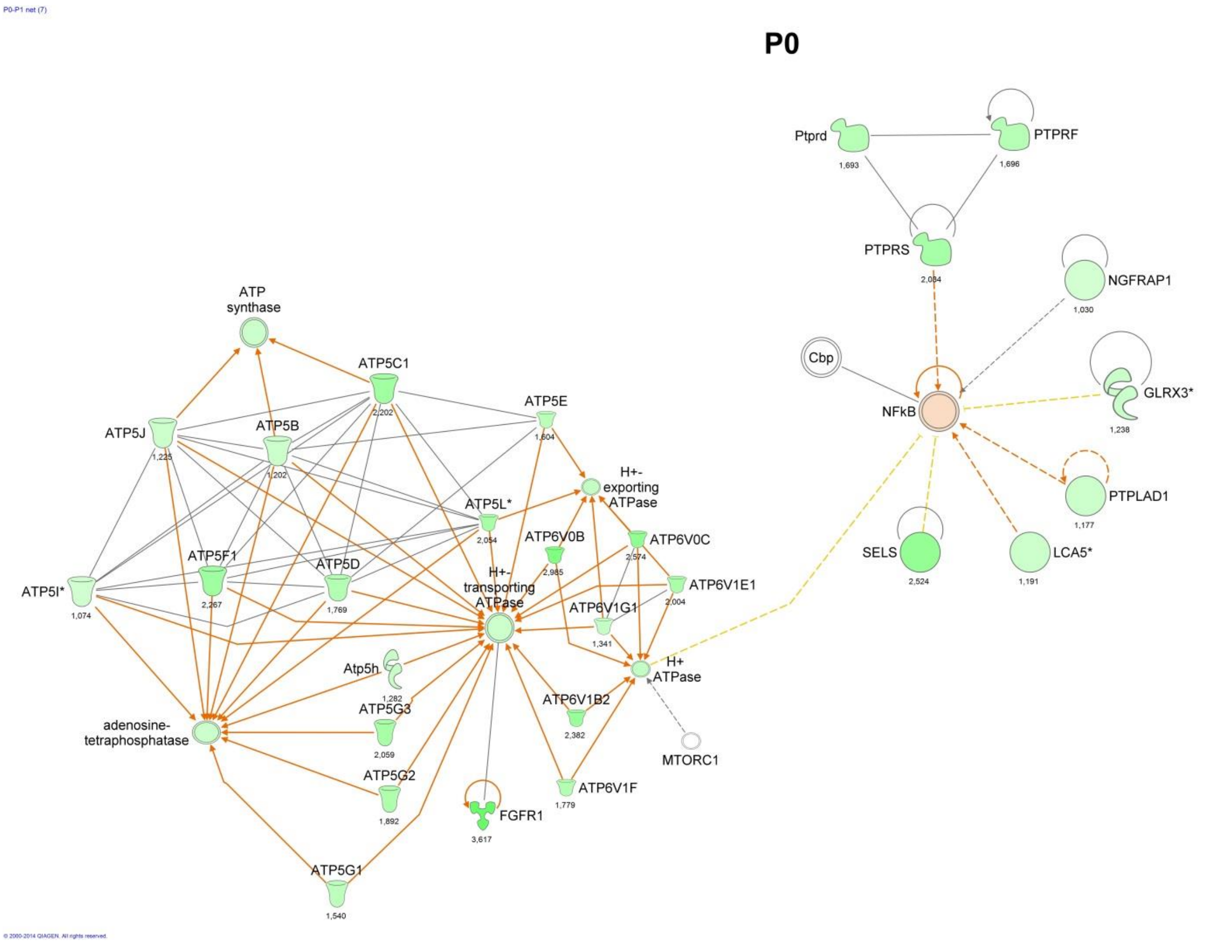

Network 12

Energy production, nucleic acid metabolism, small molecule biochemistry

P0-P1 Net (11)

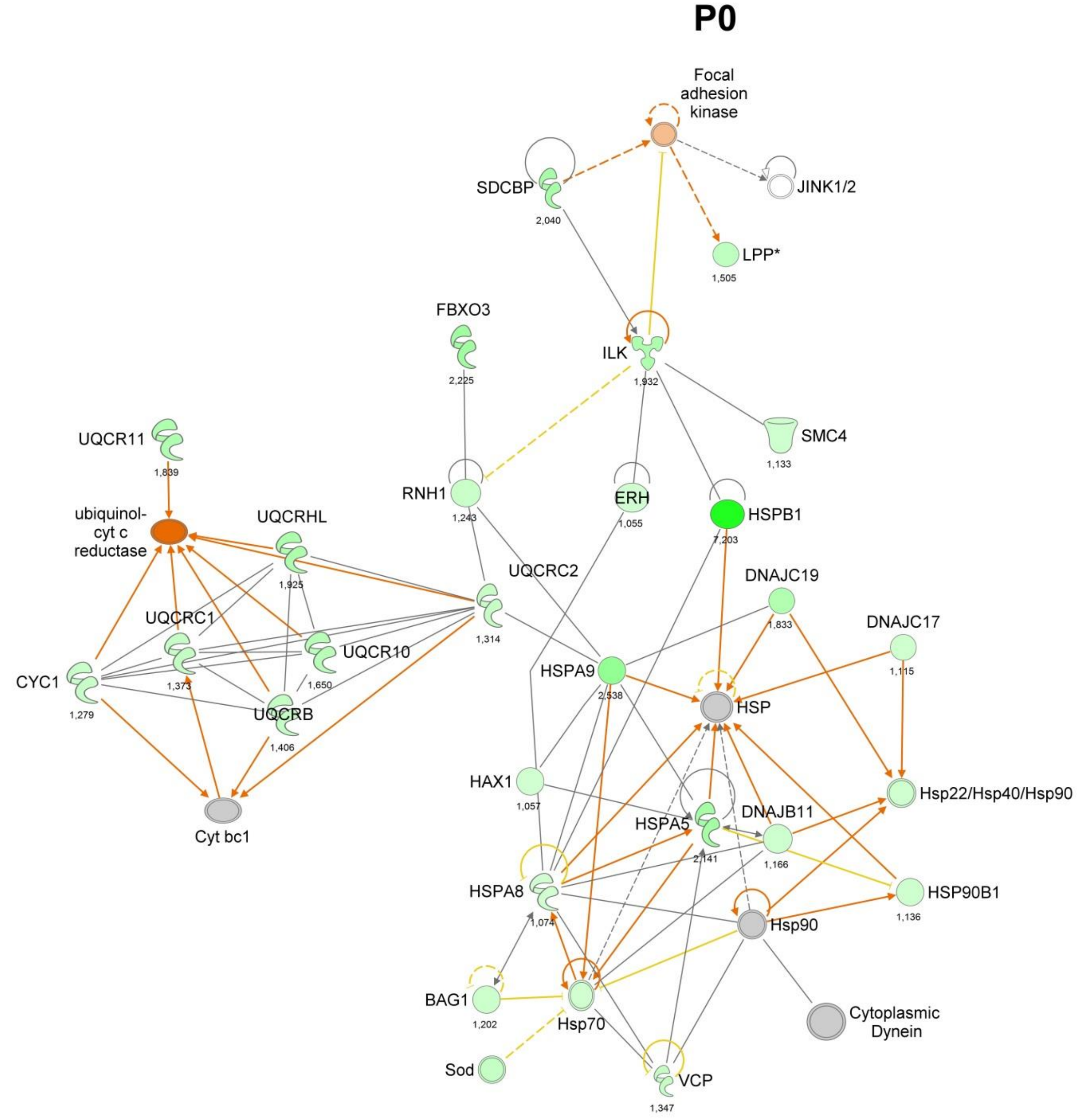

© 2000-2014 QIAGEN. All rights reserved.

Network 13

Cellular compromise, cellular function and maintenance, protein degradation

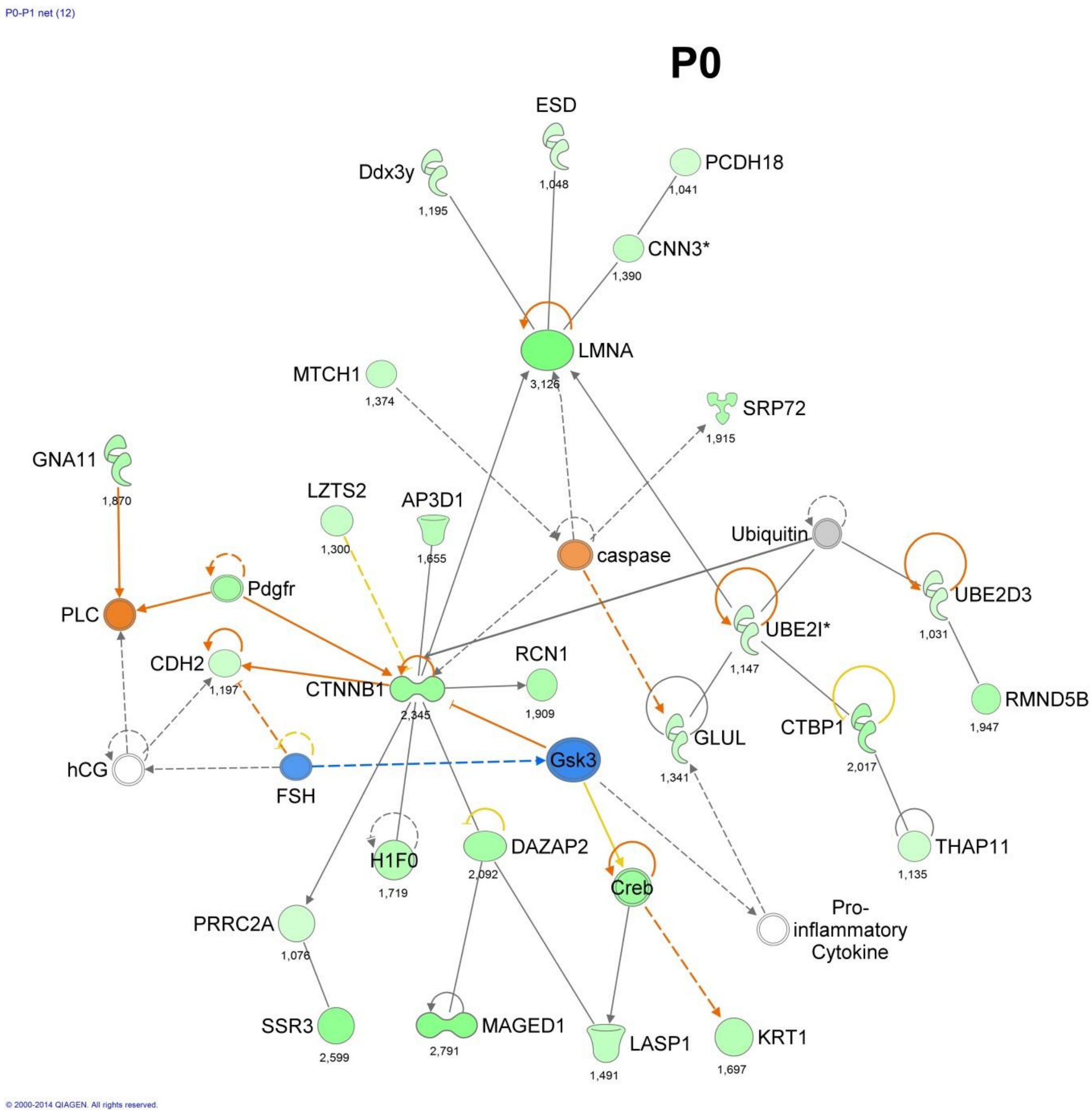

Network 14

Connective tissue development and function, embryonic development

P0-P1 net (13)

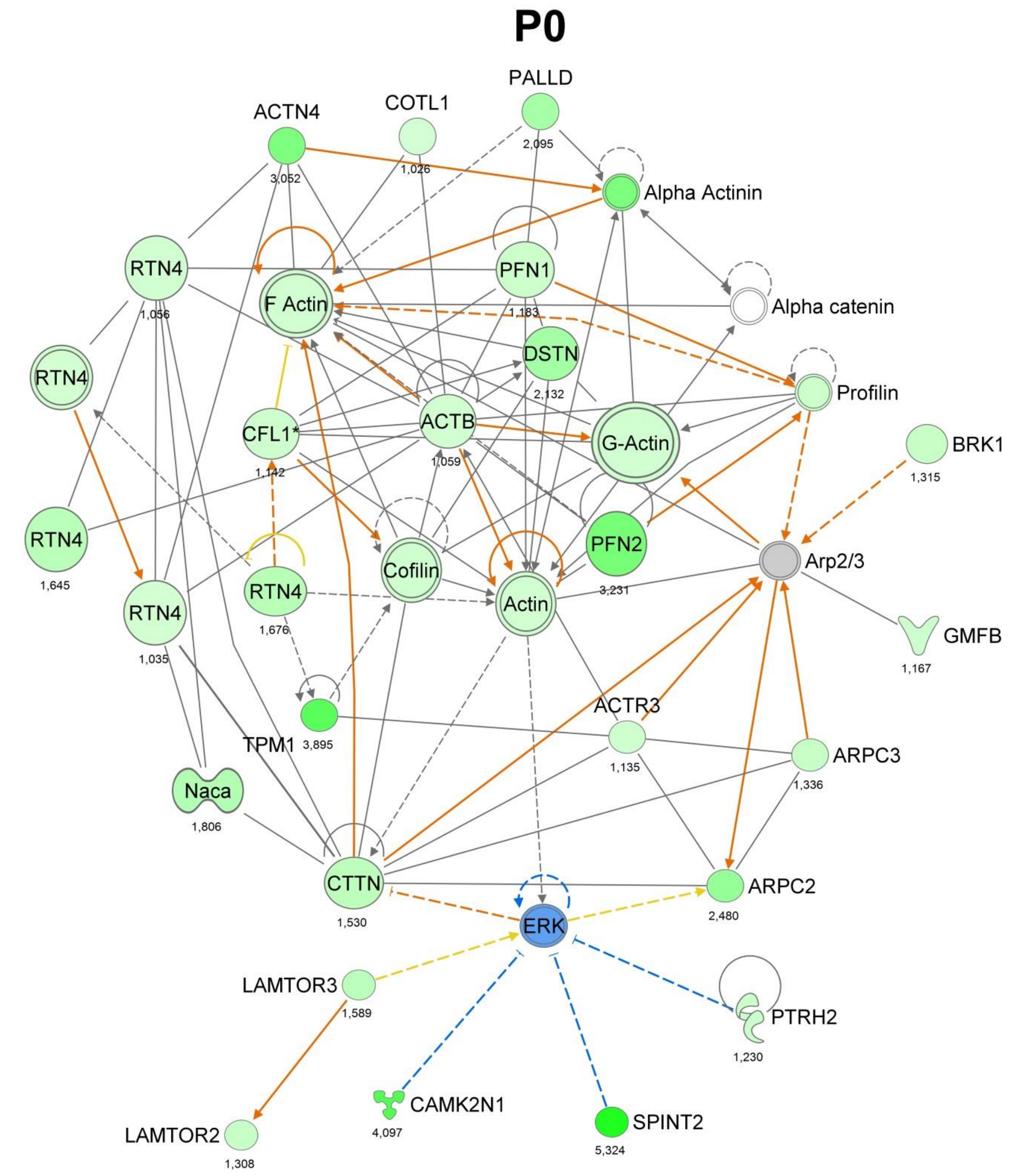

© 2000-2014 QIAGEN. All rights reserved.

Network 15

Cellular assembly and organization, tissue development, cellular function and maintenance

P0-P1 net (20)

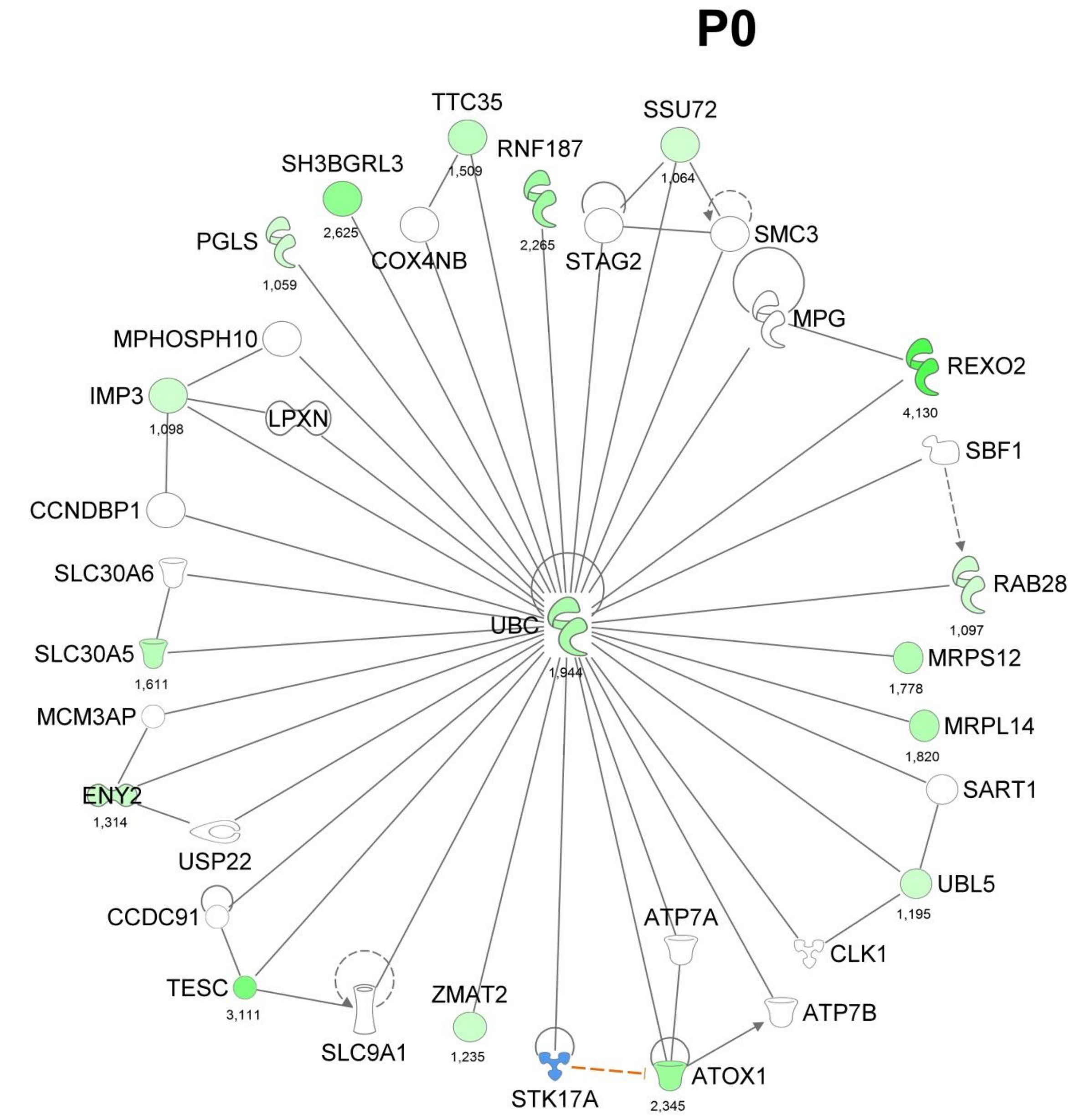

© 2000-2014 QIAGEN. All rights reserved.

Network 16

Molecular transport, cellular function and maintenance, small molecule biochemistry

P0-P1 net (1)*

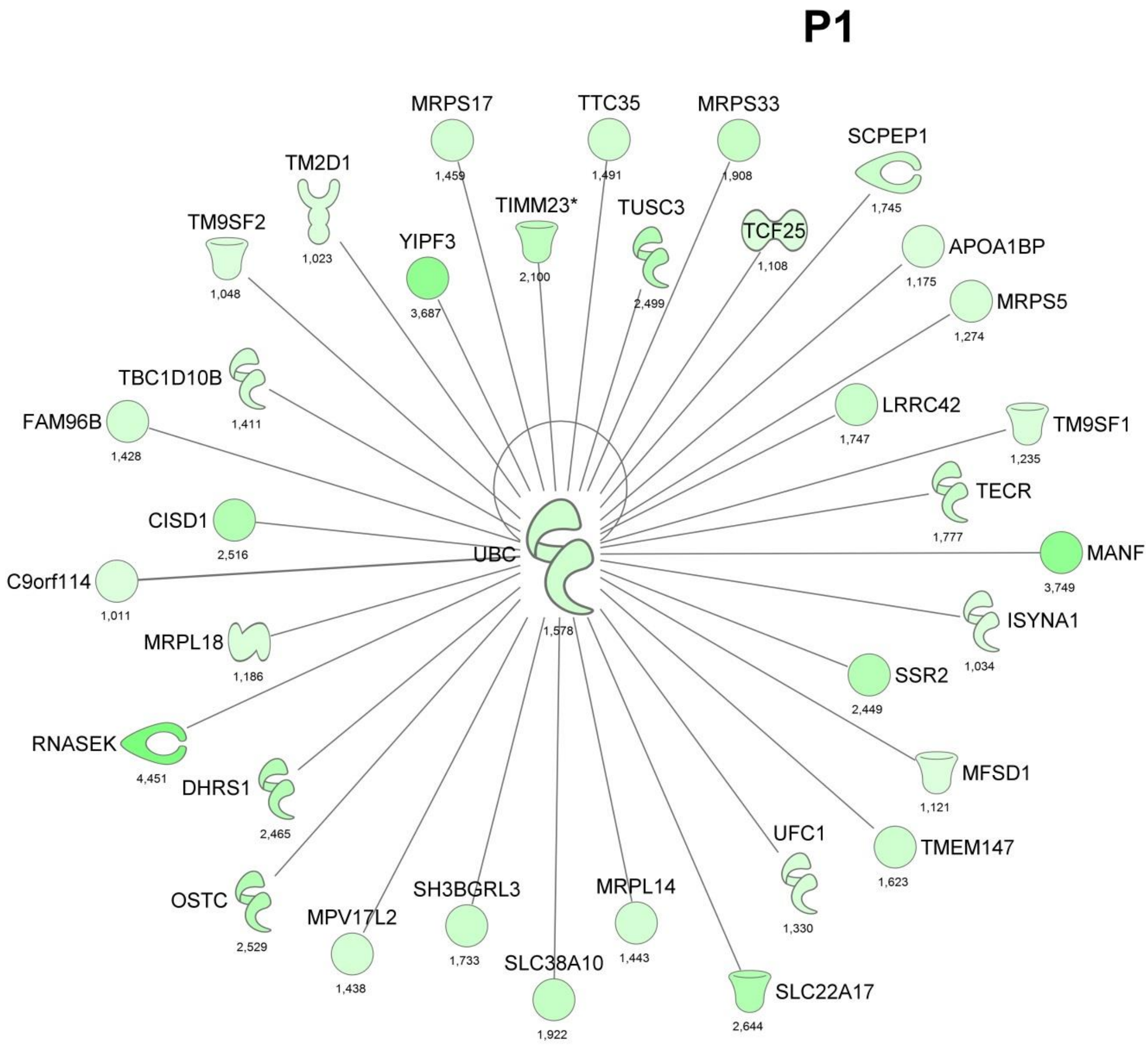

© 2000-2014 QIAGEN. All rights reserved.

Network 1

Lipid metabolism, molecular transport, small molecule biochemistry

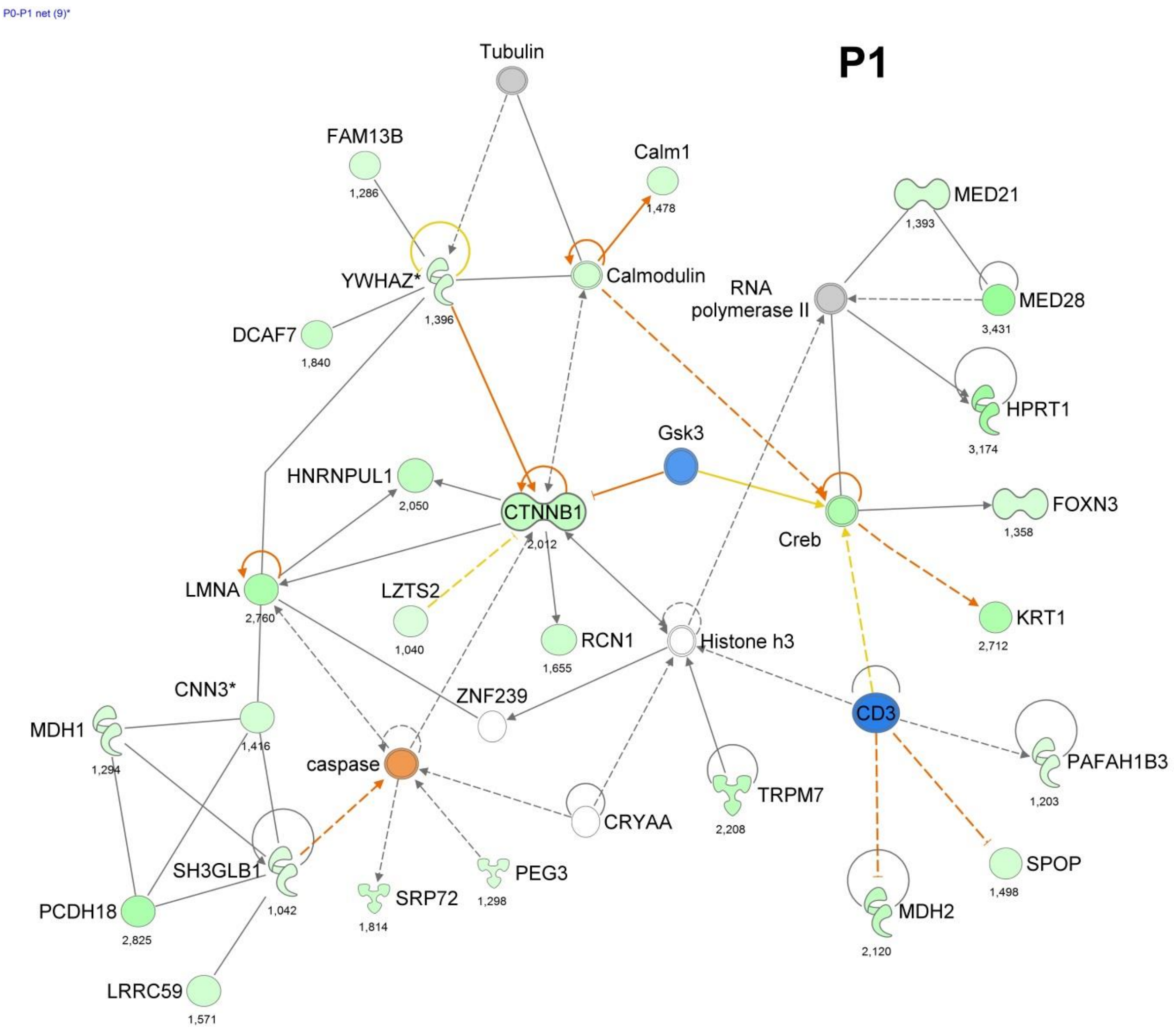

© 2000-2014 QIAGEN. All rights reserved.

Network 2

Lipid metabolism, cellular function and maintenance, energy production

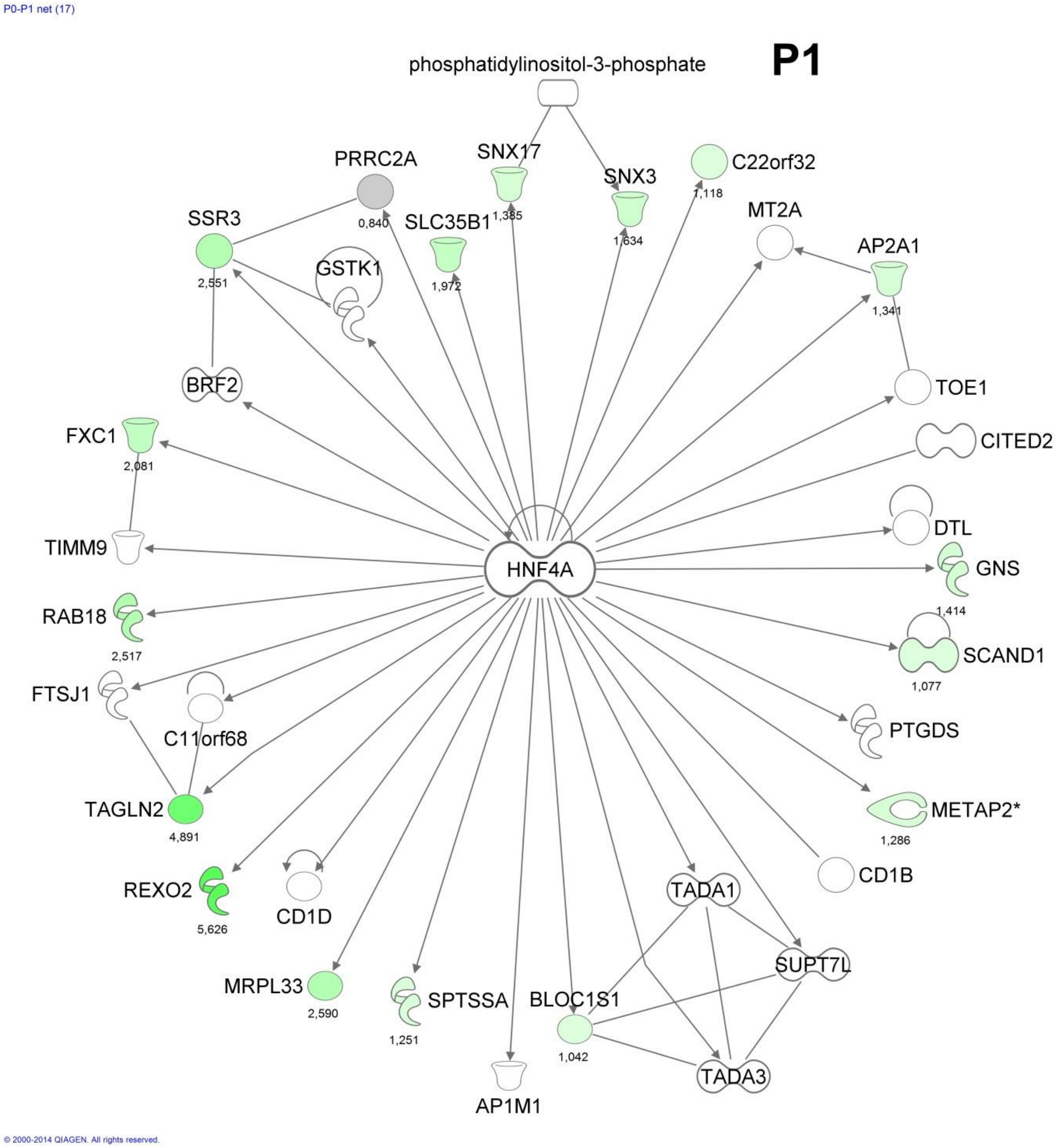

Network 3

Lipid metabolism, small molecule biochemistry, cellular assembly and organization

P0-P1 net (3)*

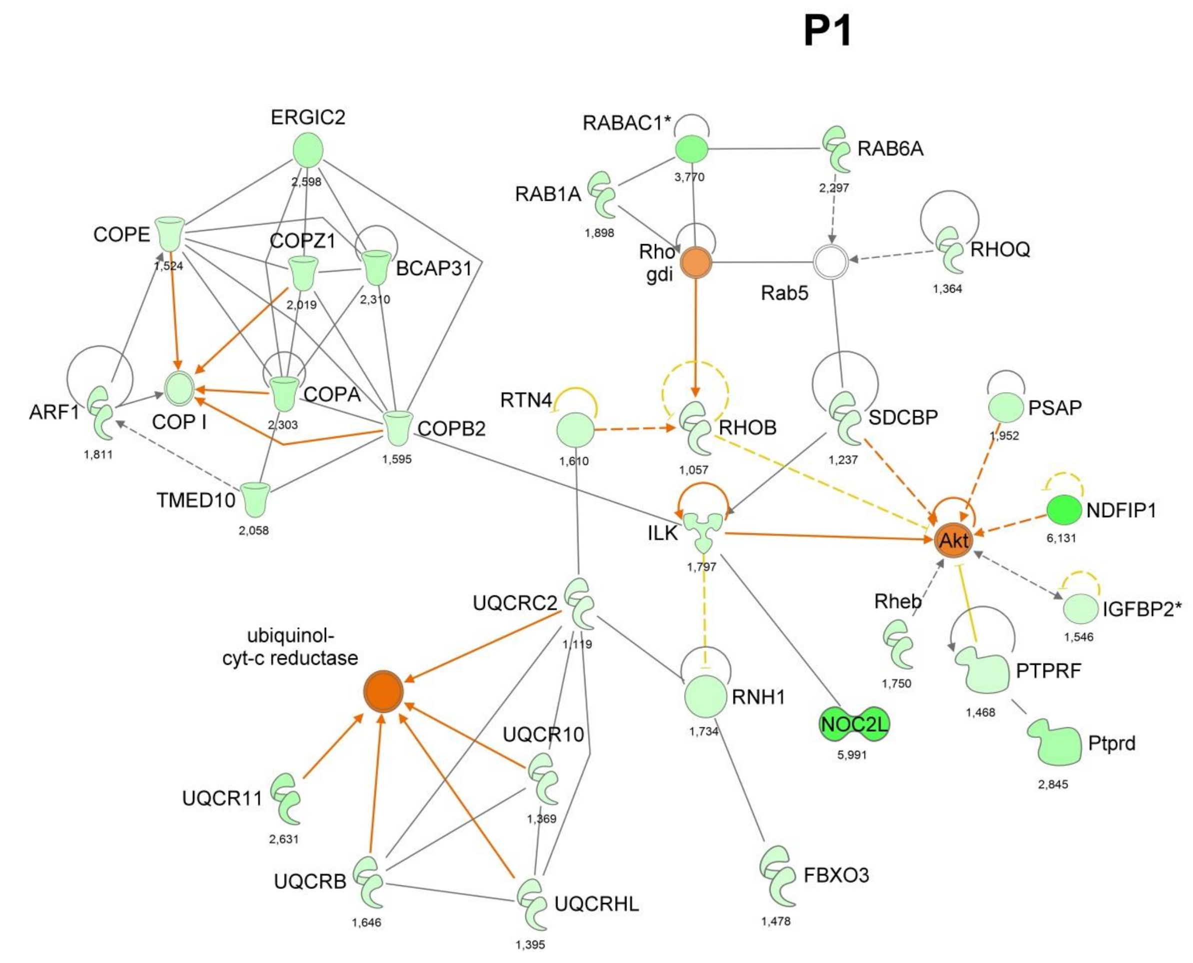

© 2000-2014 QIAGEN. All rights reserved.

Network 4

Cell-to-cell signaling and interaction, cellular function and maintenace

Path Designer Net (4)*

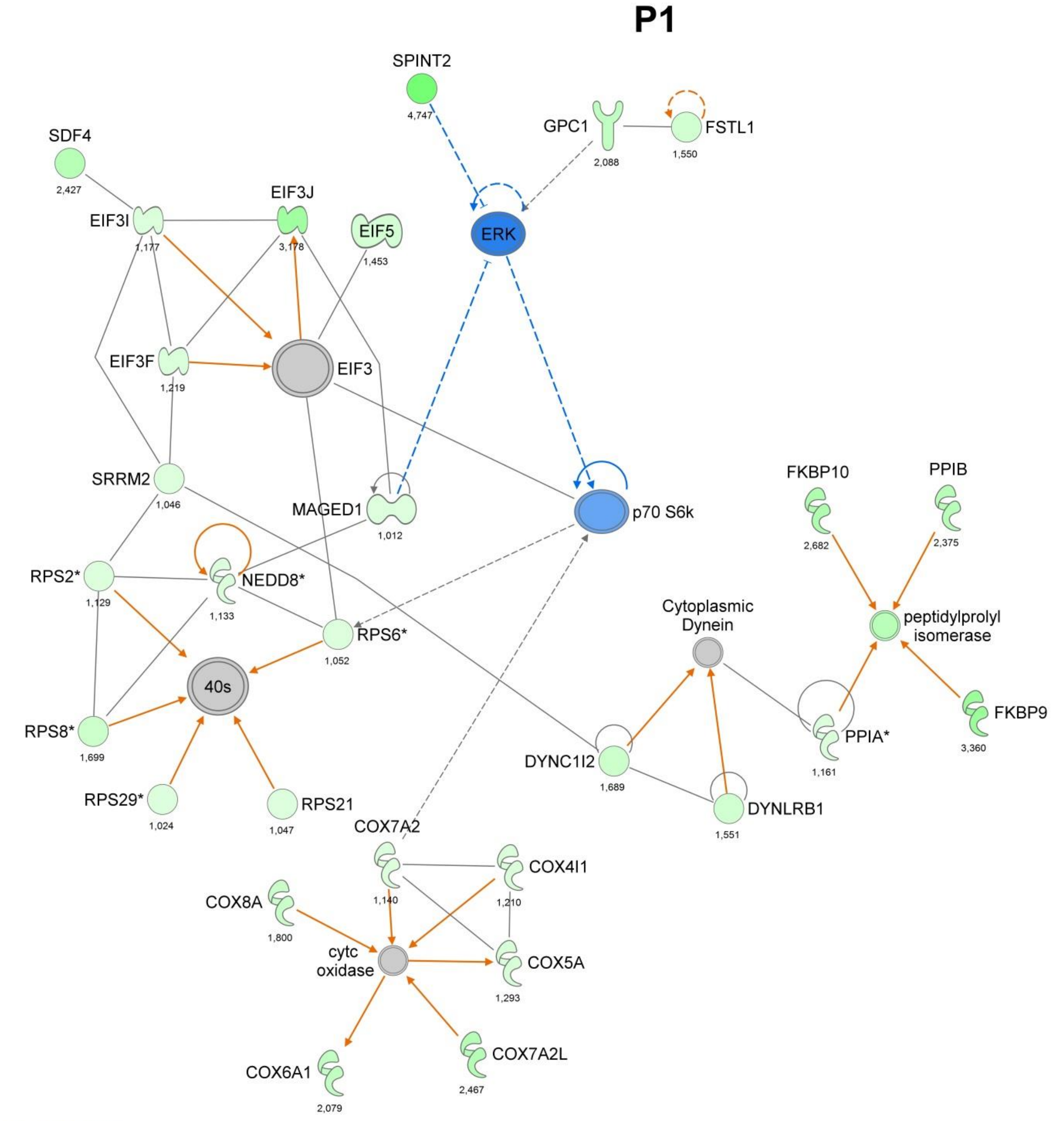

© 2000-2014 QIAGEN. All rights reserved.

Network 5

Cell death, small molecule biochemistry

P0-P1 net (6)*

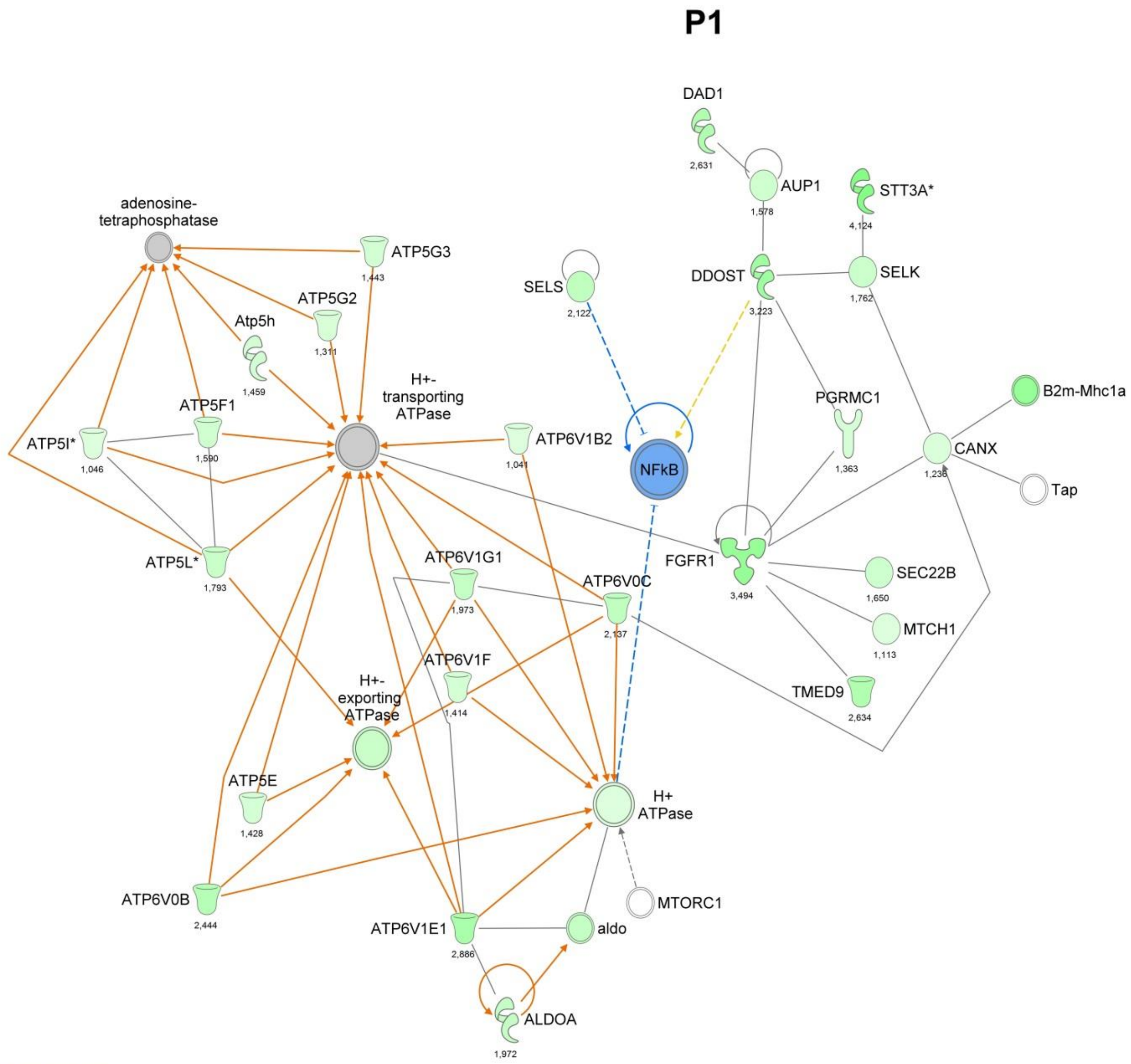

© 2000-2014 QIAGEN. All rights reserved.

Network 6

Molecular transport, DNA replication, energy production

P0-P1 net (7)*

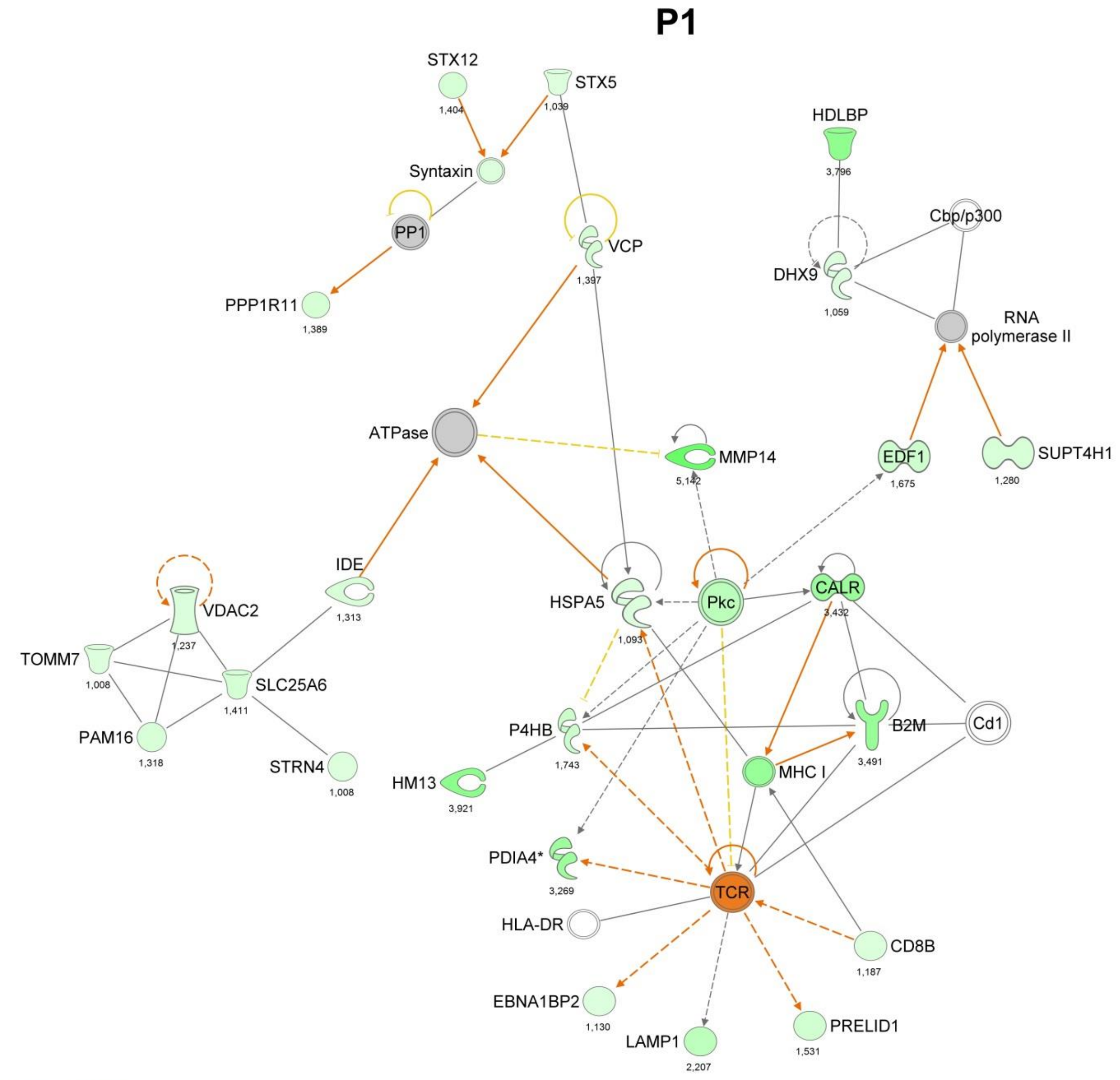

© 2000-2014 QIAGEN. All rights reserved.

Network 7

Cellular compromise, function and maintenance, post transcriptional modification

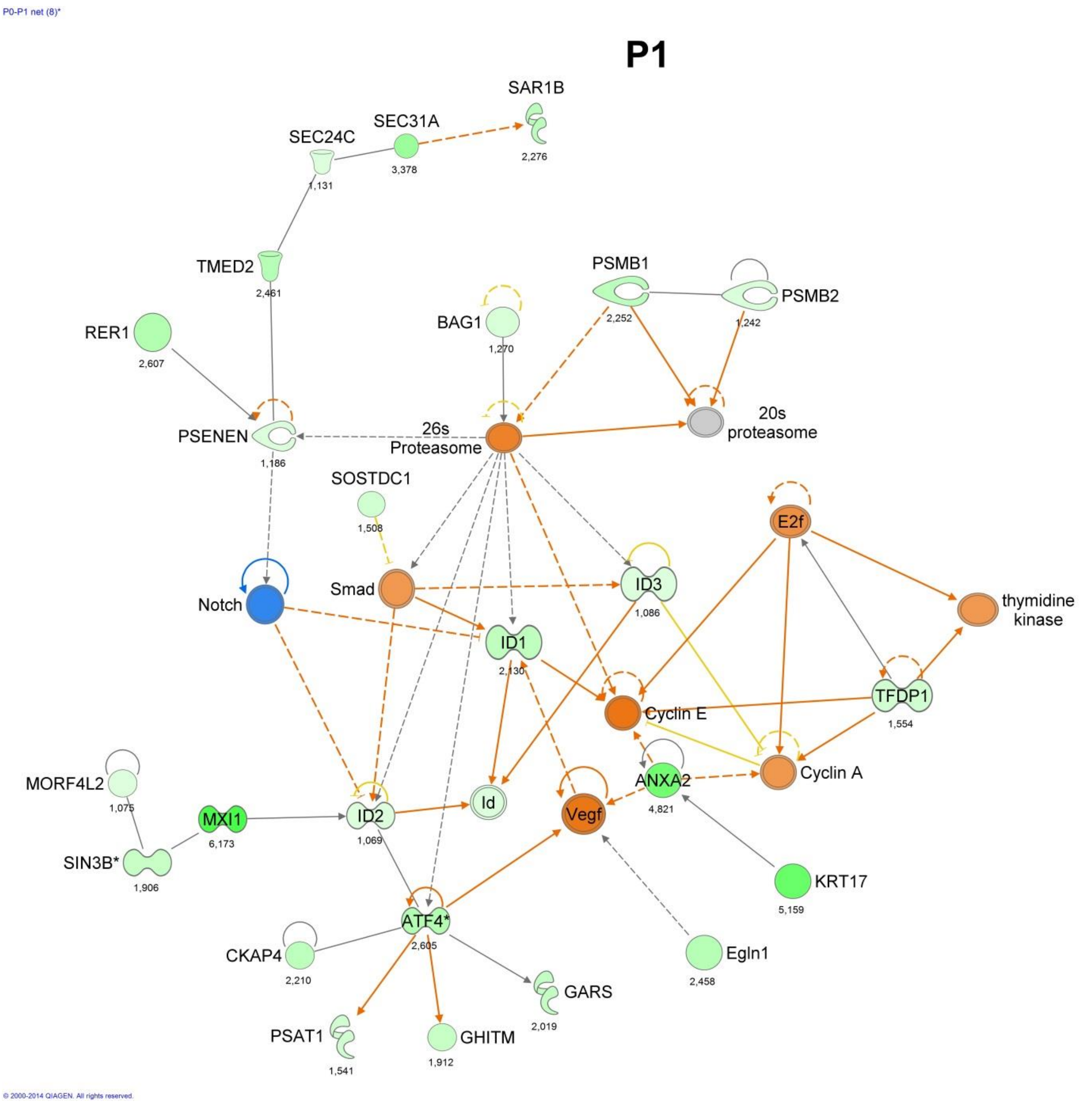

Network 8

Gene expression, cellular development, cellular growth and proliferation

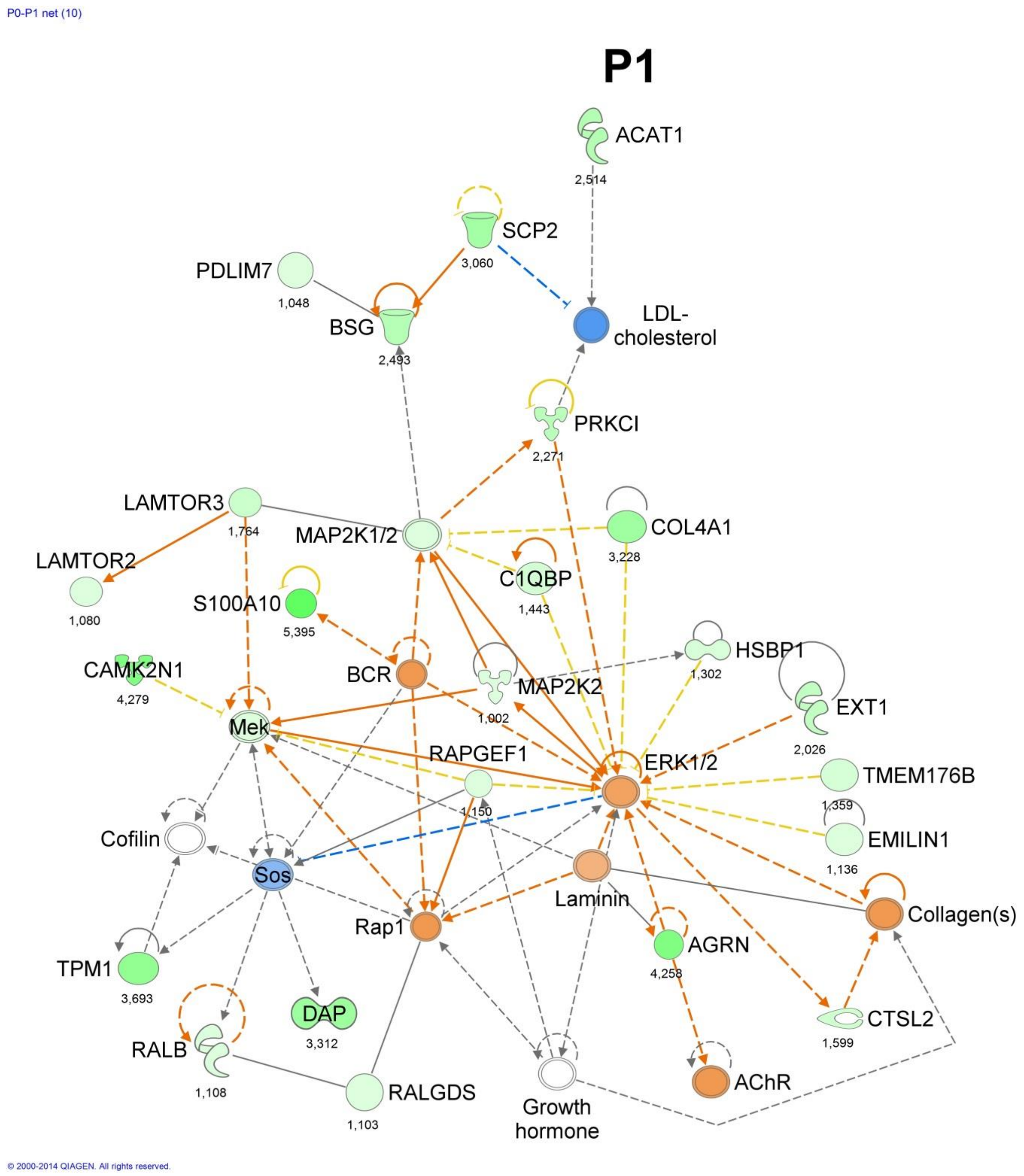

Network 9

Cell morphology, cellular asembly and organization

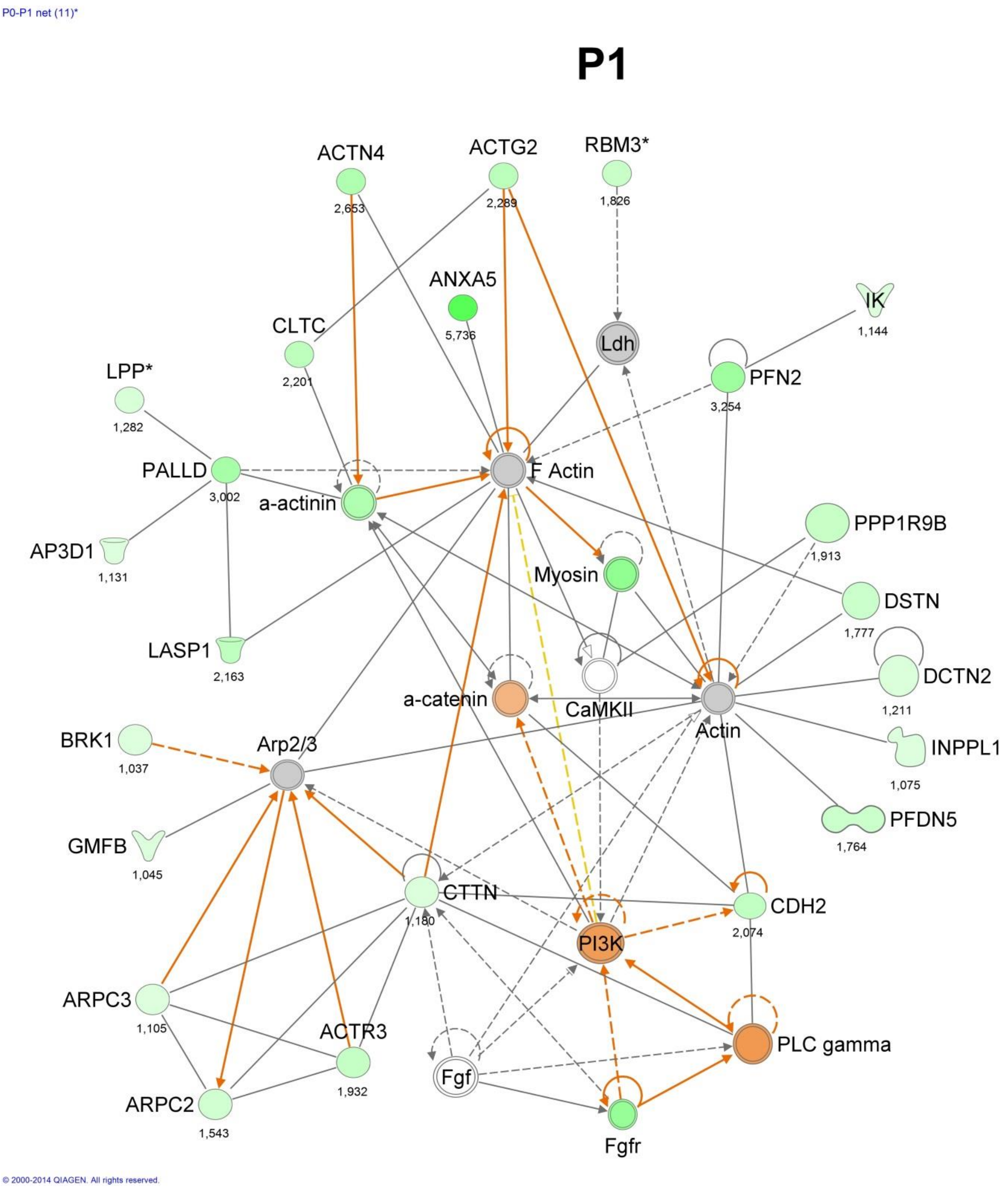

© 2000-2014 QIAGEN. All rights reserved.

Network 10

Cellular assembly and organization, tissue development, cellular function and maintenance

P0-P1 net (12)*

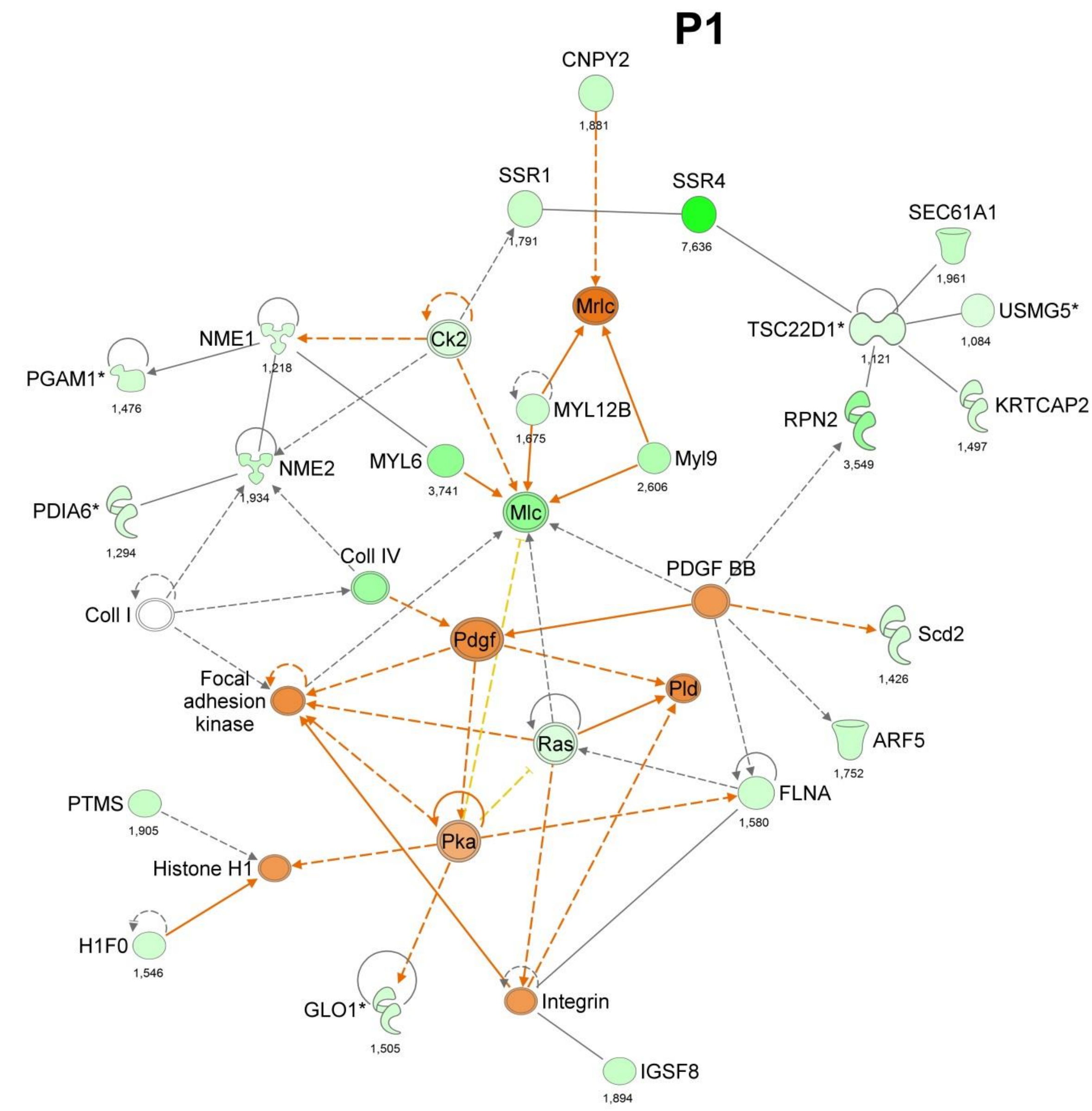

© 2000-2014 QIAGEN. All rights reserved.

Network 11

DNA metabolism, nucleic acid metabolism, small molecule biochemistry

P0-P1 Net (13)*

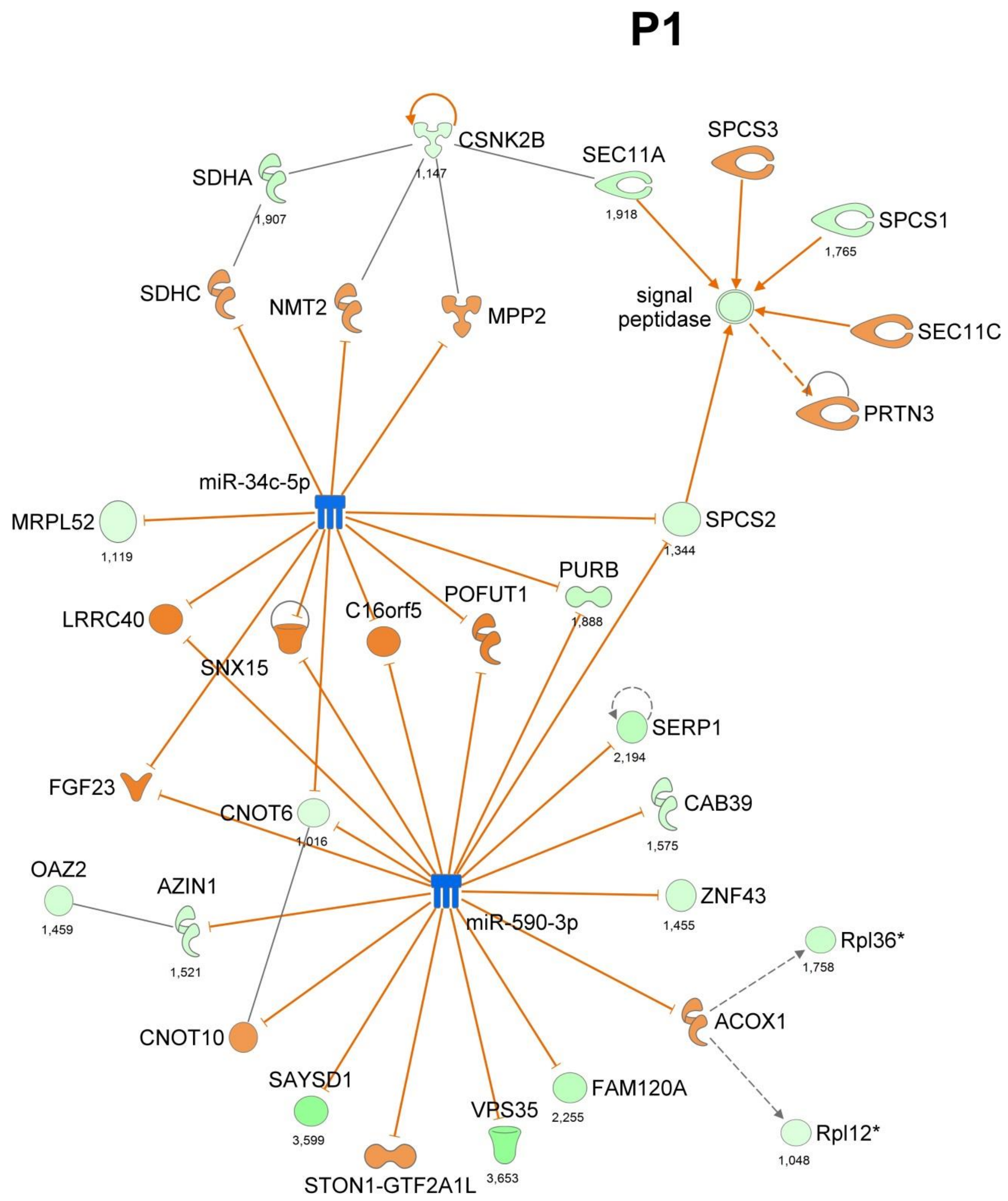

© 2000-2014 QIAGEN. All rights reserved.

Network 12

Cell cycle, cellular development, nucleic acid metabolism

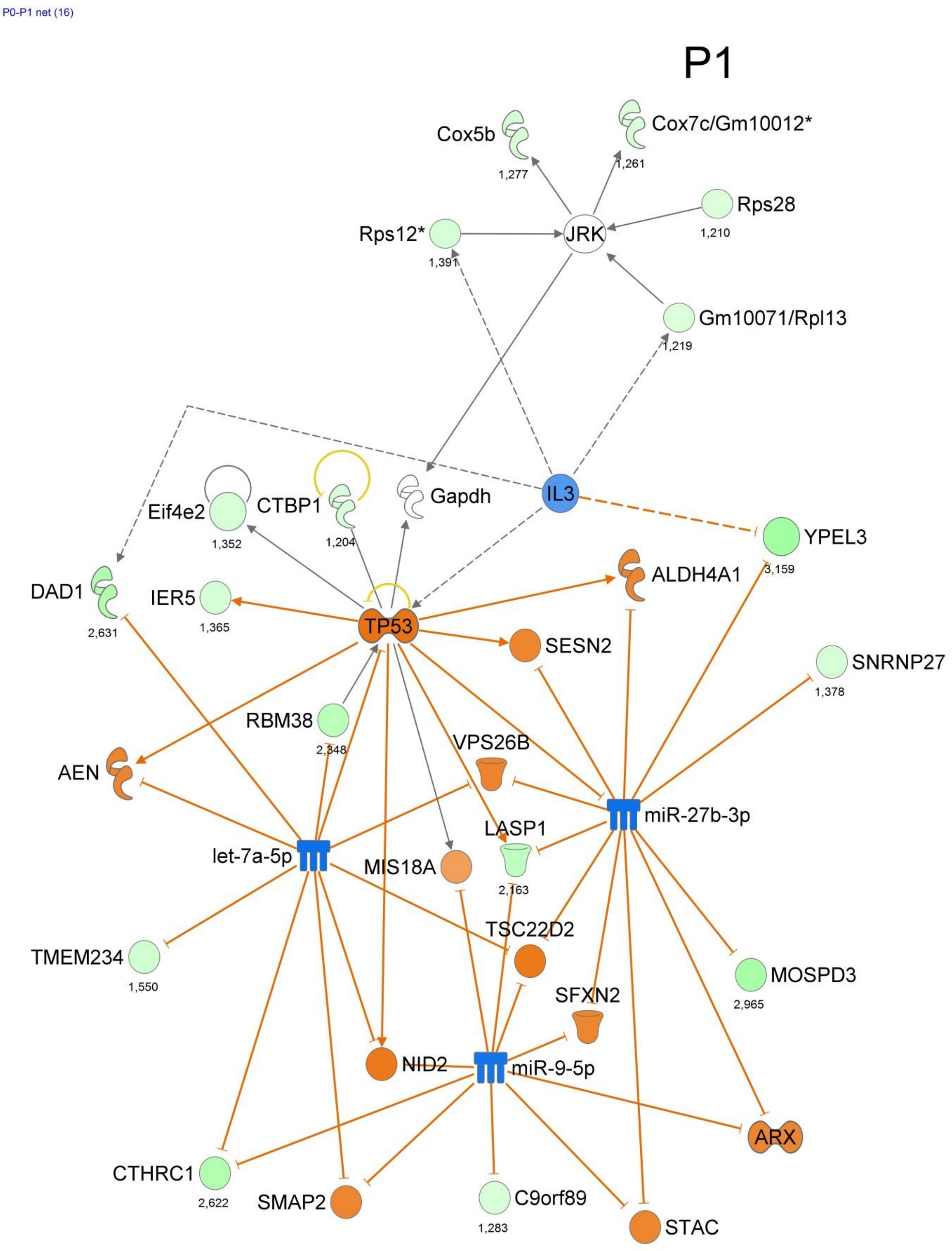

© 2000-2014 QIAGEN. All rights reserved.

Network 13

Cell death, cellular movment

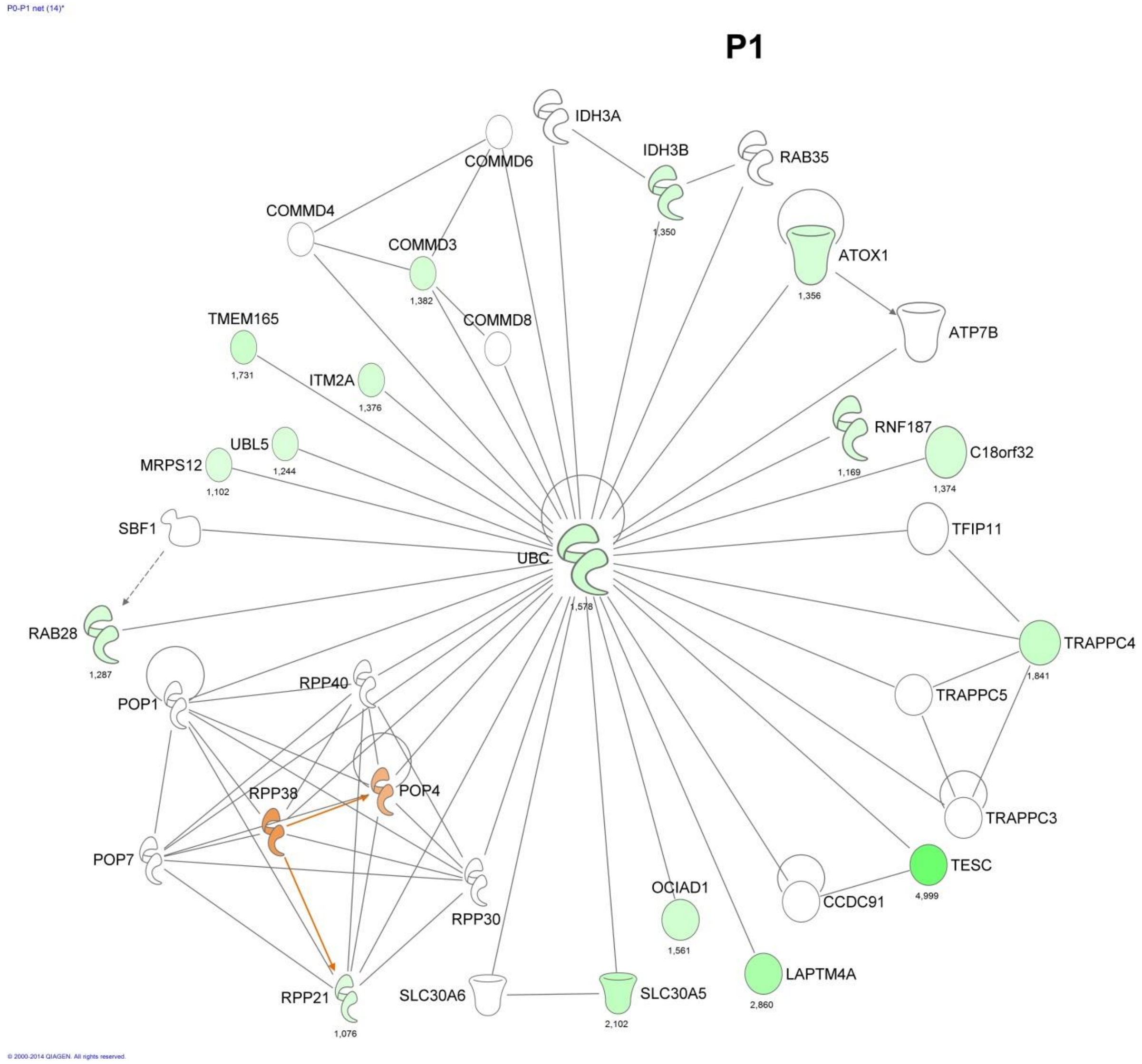

Network 14

Molecular transport, carbohydrate, small molecule biochemistry

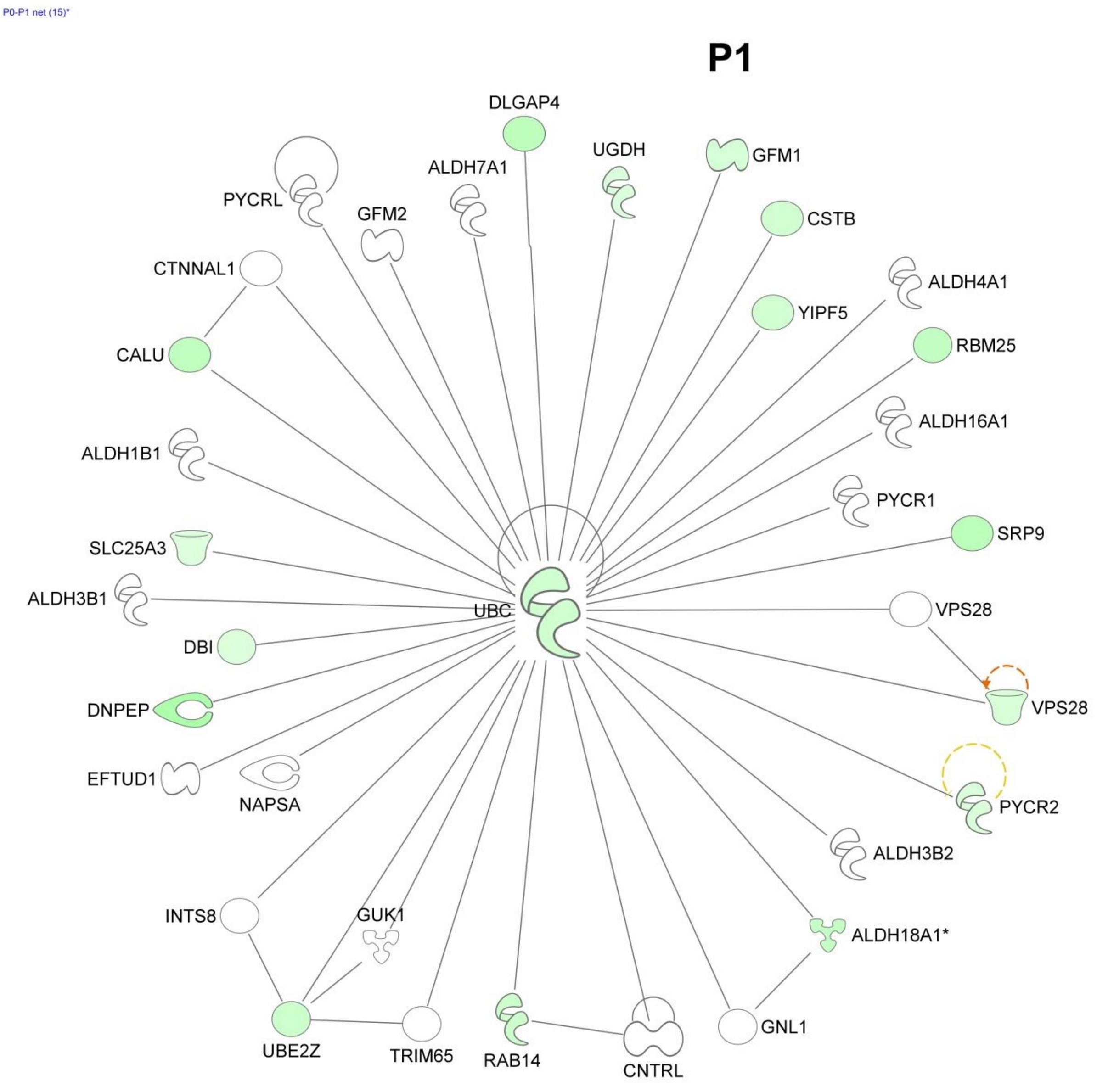

© 2000-2014 QIAGEN. All rights reserved.

Network 15

Carbohydrate, nucleic acid metabolism, small molecule biochemistry

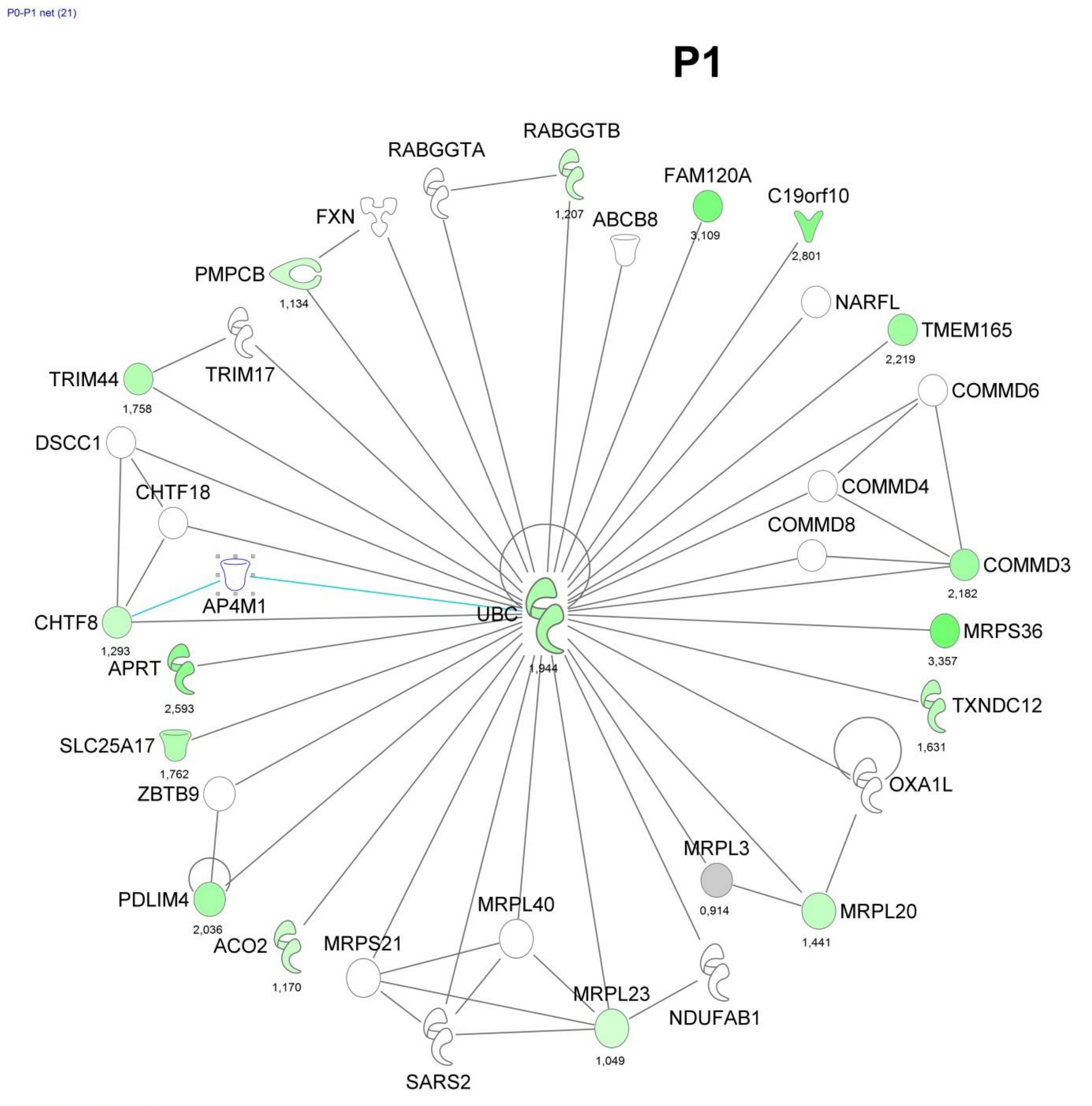

© 2000-2014 QIAGEN. All rights reserved.

Network 16

Post-transcriptional modification, vitamin and mineral metabolism

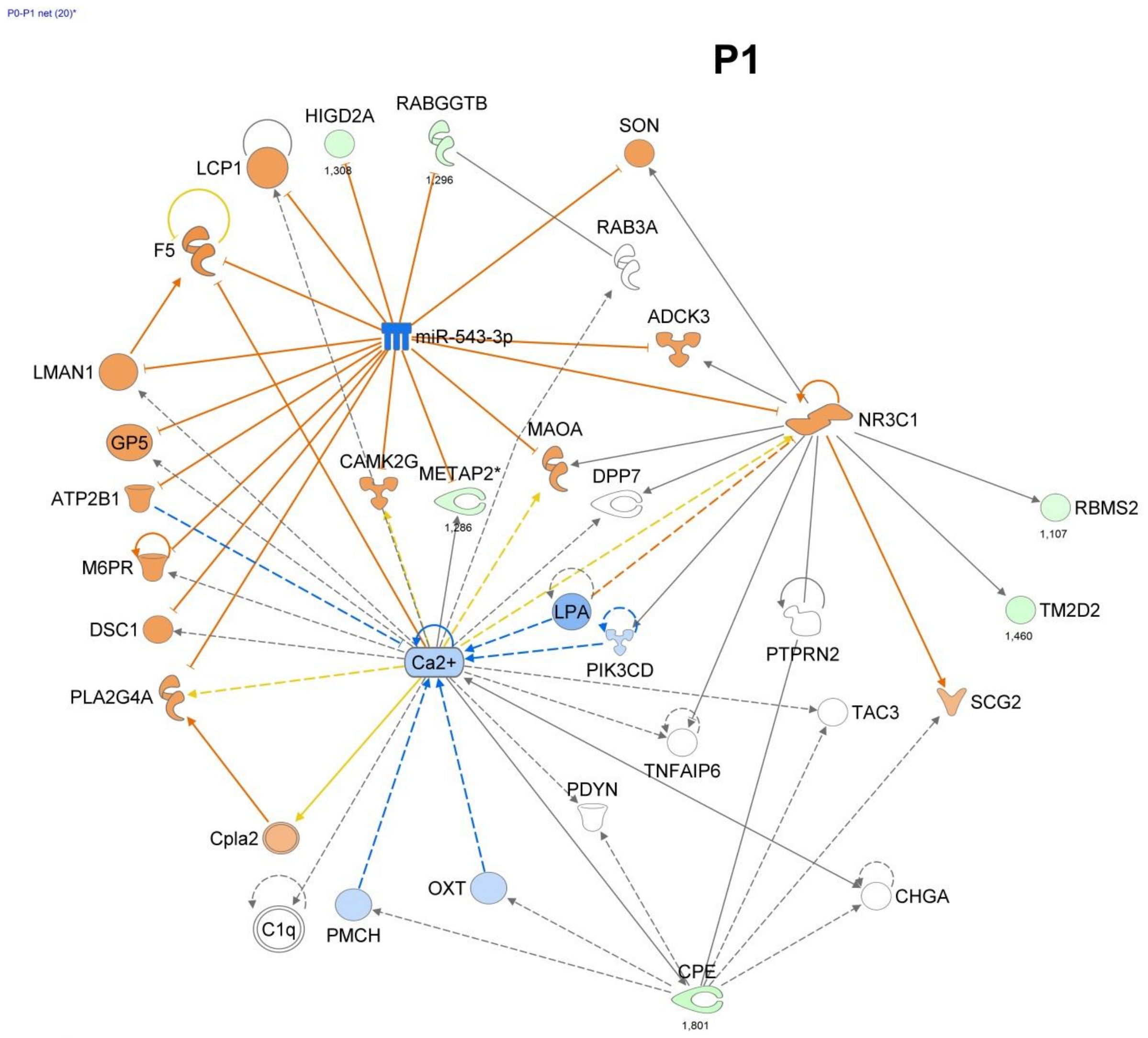

© 2000-2014 QIAGEN. All rights reserved.

Network 17

Molecular transport, cellular function and maintenance, cell-to-cell signaling and interaction

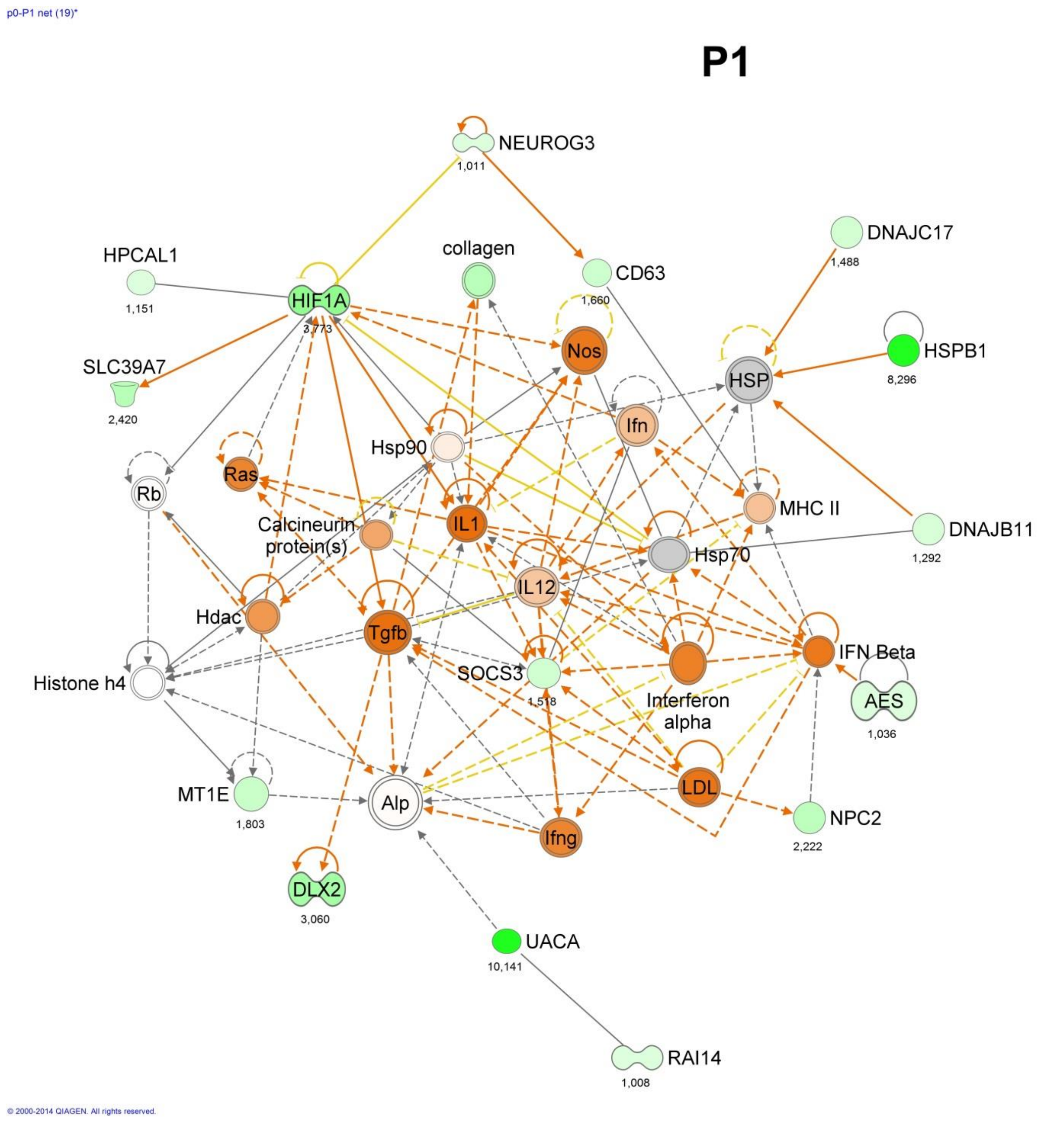

Network 18

Protein synthesis, organ morphology

P0-P1 net (18)*

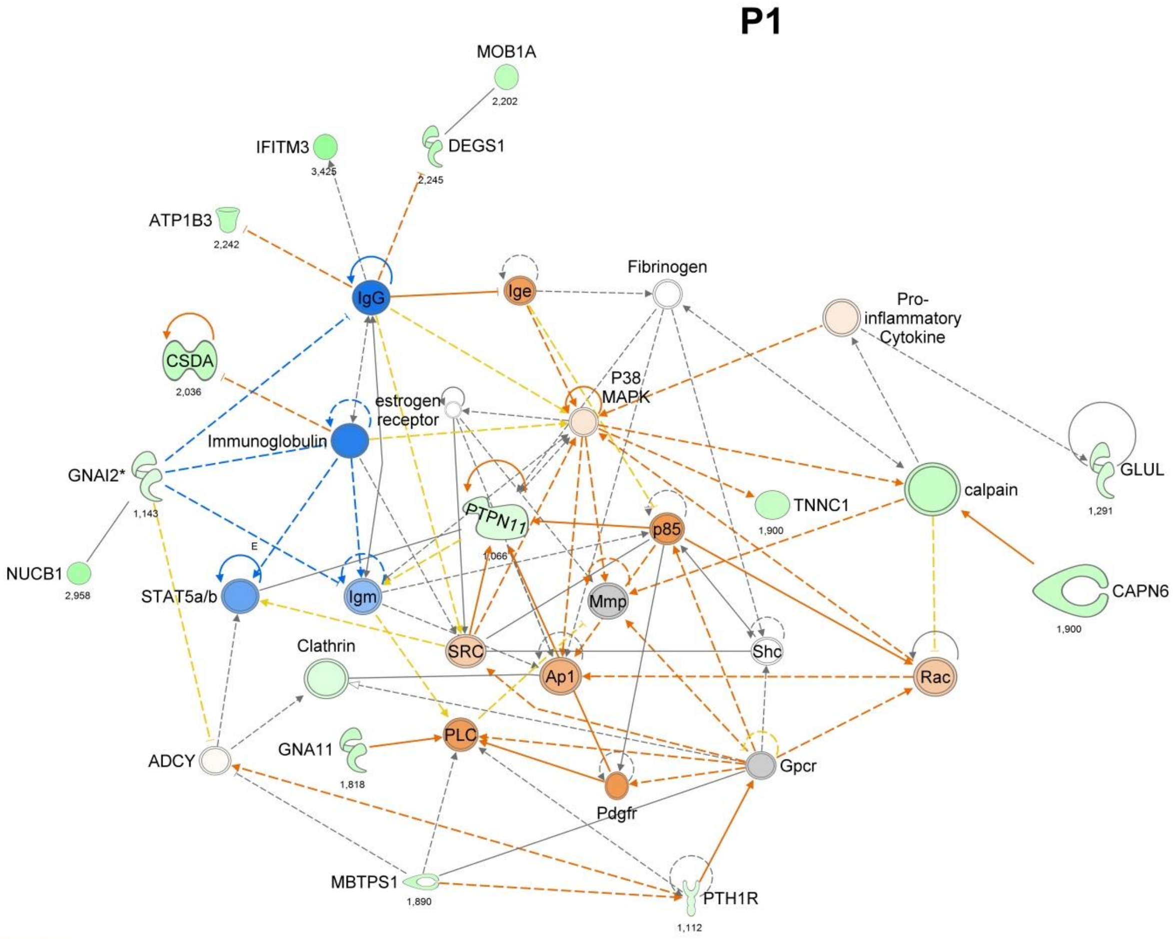

© 2000-2014 QIAGEN. All rights reserved.

Network 19

Connective tissue development and function, embryonic development

P2-P4 net (1) UBC

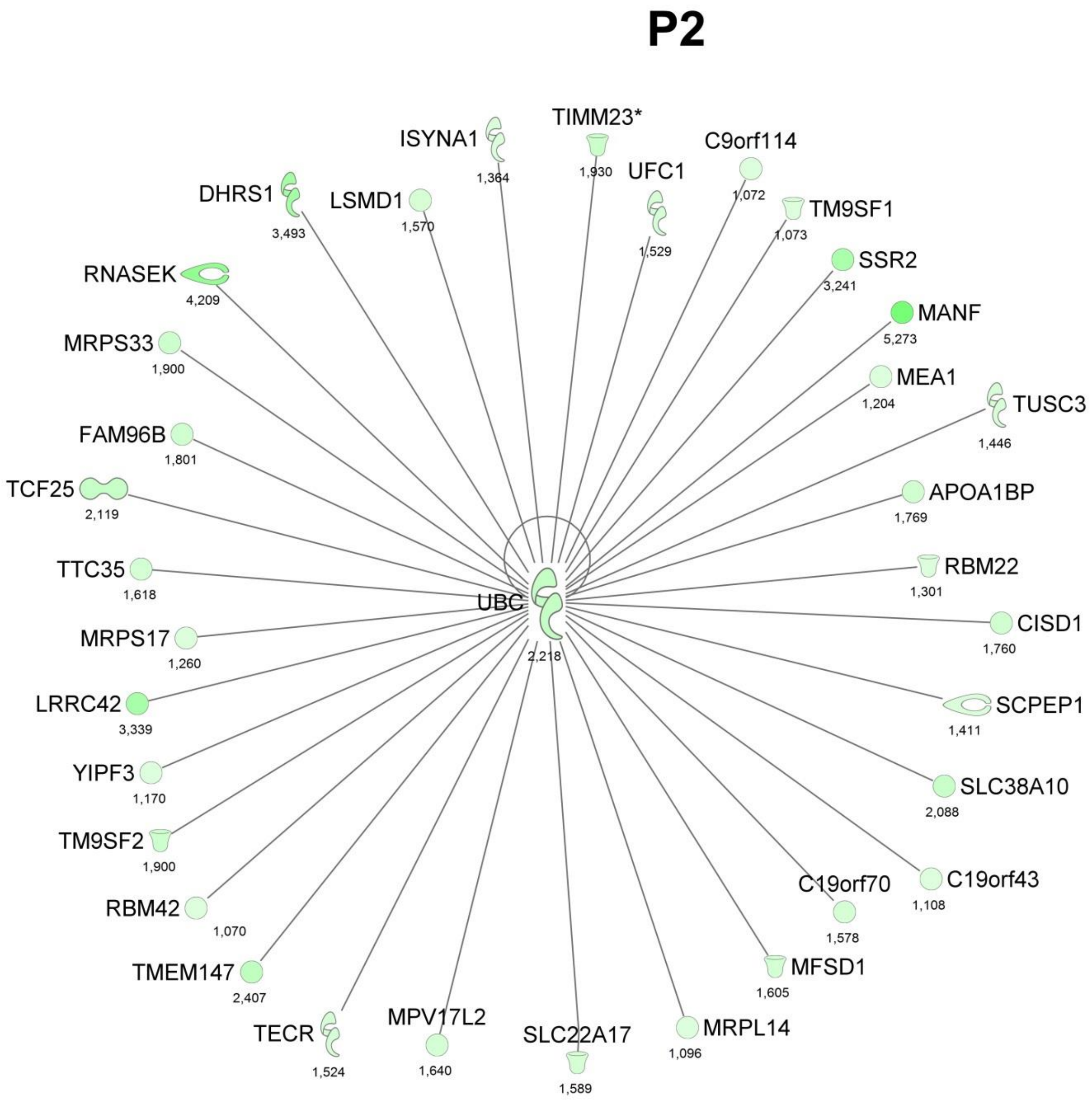

© 2000-2014 QIAGEN. All rights reserved.

Network 1

Lipid metabolism, nucleic acid metabolism, smal molecule biochemistry

P2-P4 Net (3)

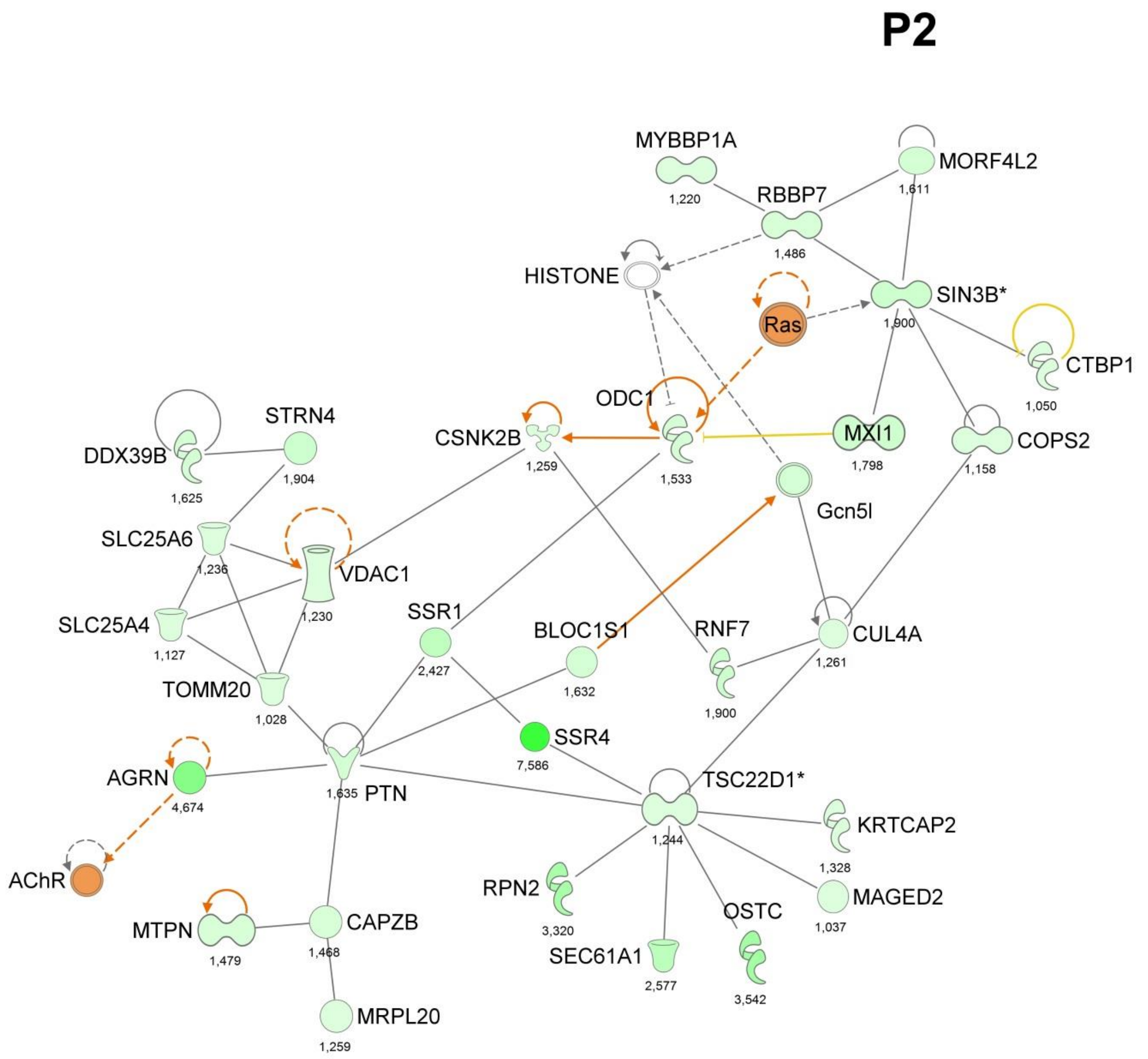

© 2000-2014 QIAGEN. All rights reserved.

Network 2

Cell cycle, cellular assembly and organization

P2-P4 net (5)

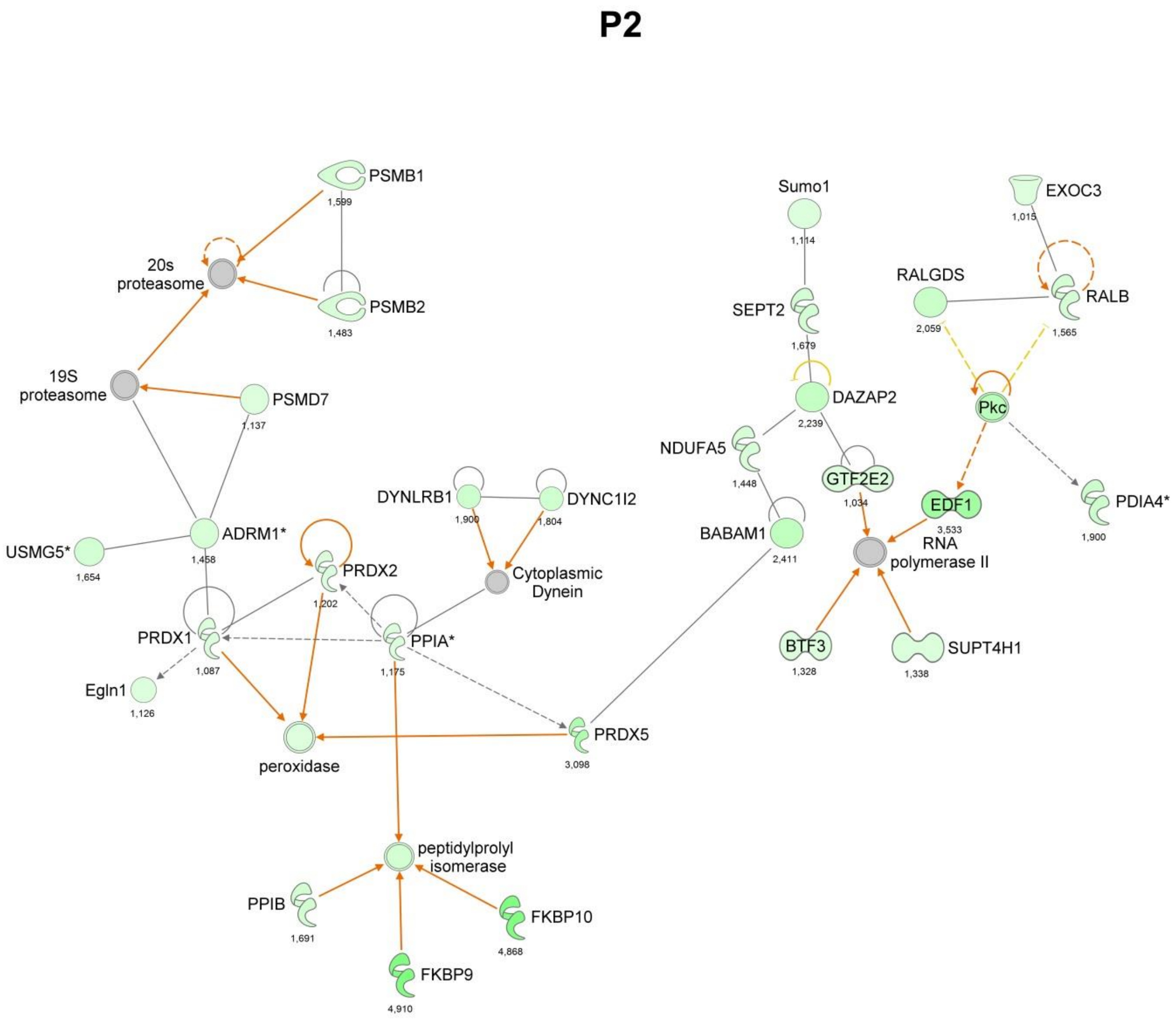

© 2000-2014 QIAGEN. All rights reserved.

Network 3

Free radical scavenging, small molecule biochemistry, cell death

P2-P4 net (6)

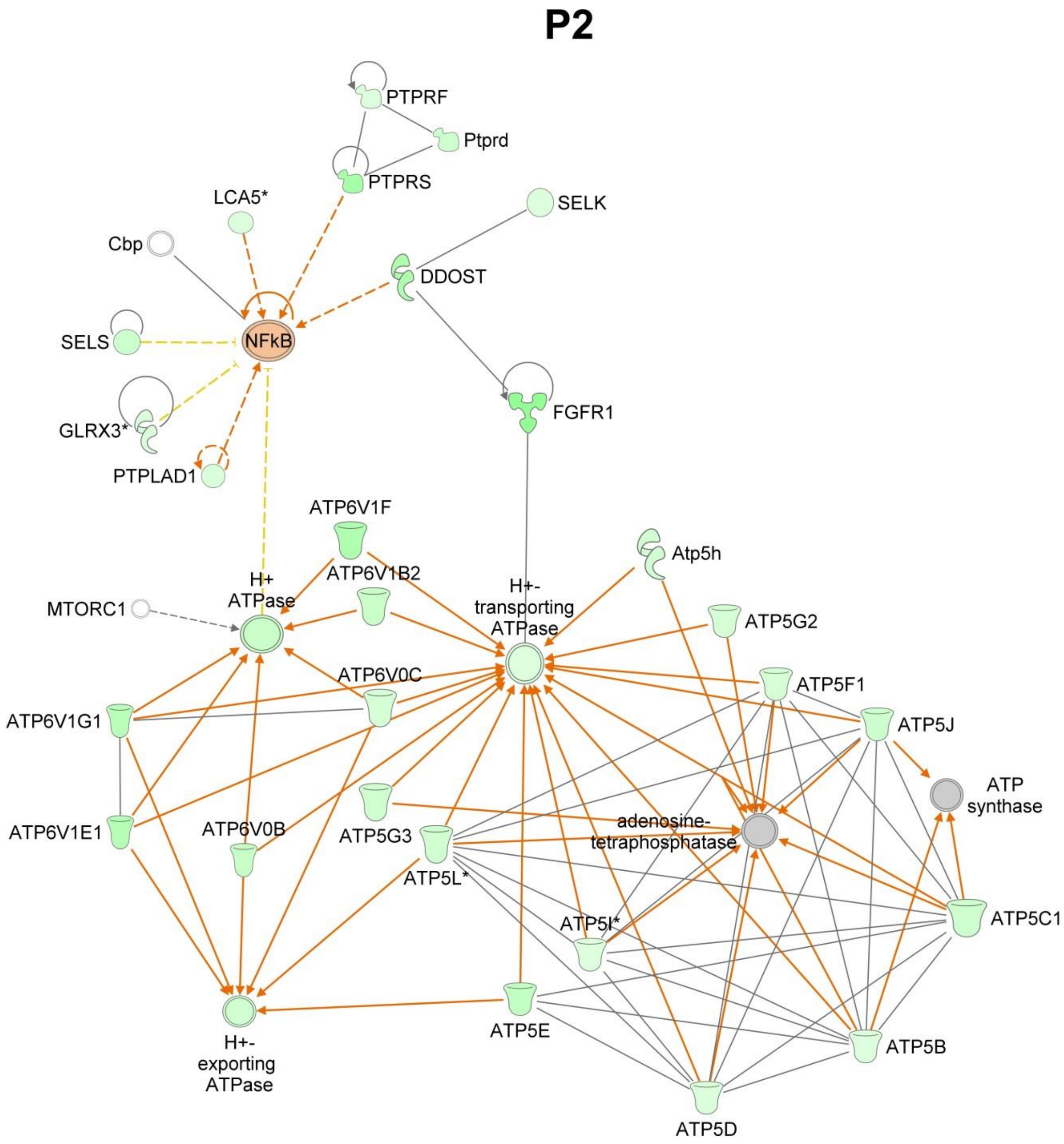

© 2000-2014 QIAGEN. All rights reserved.

Network 4

Molecular transport, DNA replication, energy production

P2-P4 net (8)

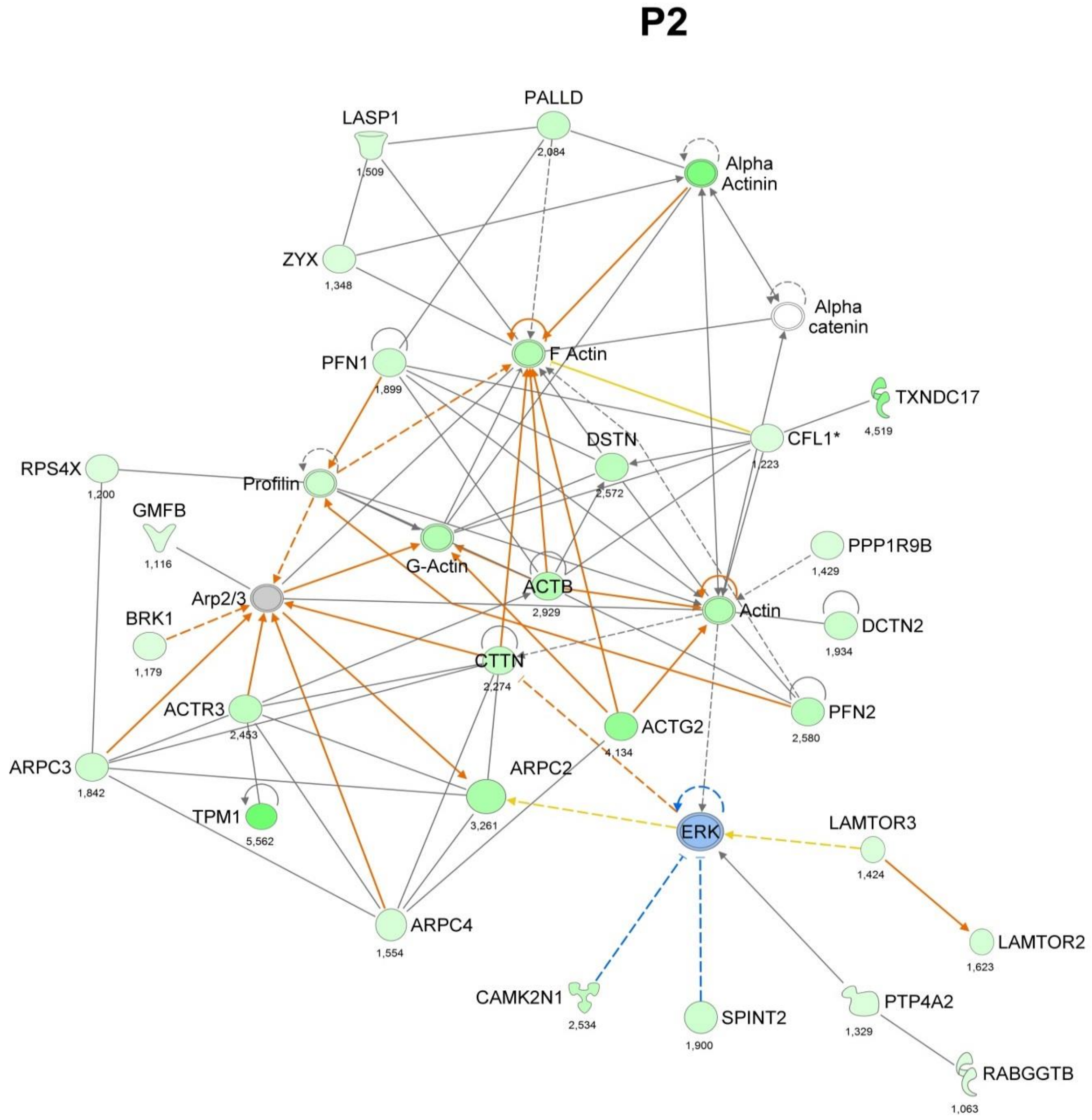

© 2000-2014 QIAGEN. All rights reserved.

Network 5

Cellular assembly and organization, cellular function and maintenance, tissue development

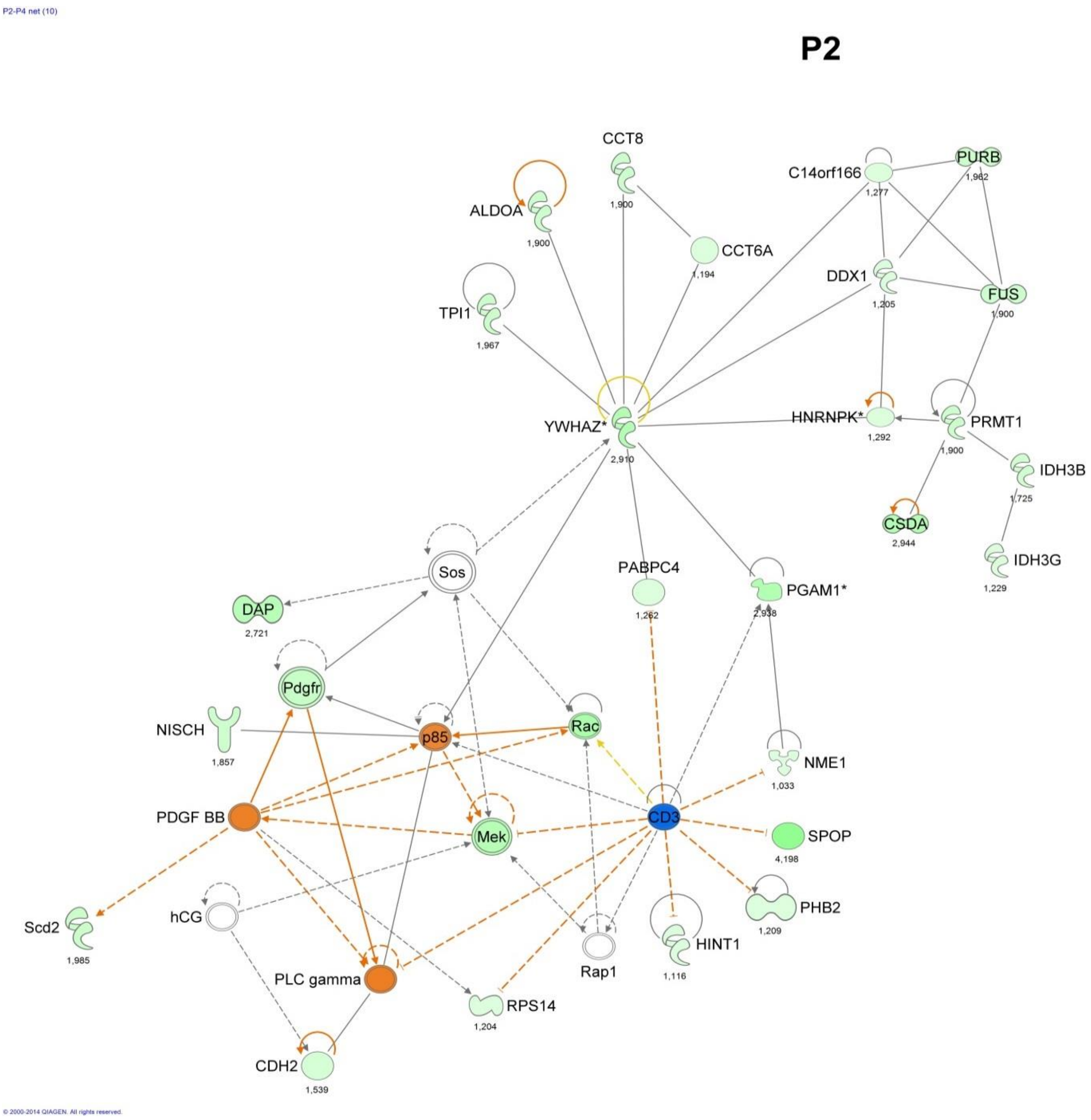

Network 6

Carbohydrate metabolism, small molecule biochemistry, nucleic acid metabolism

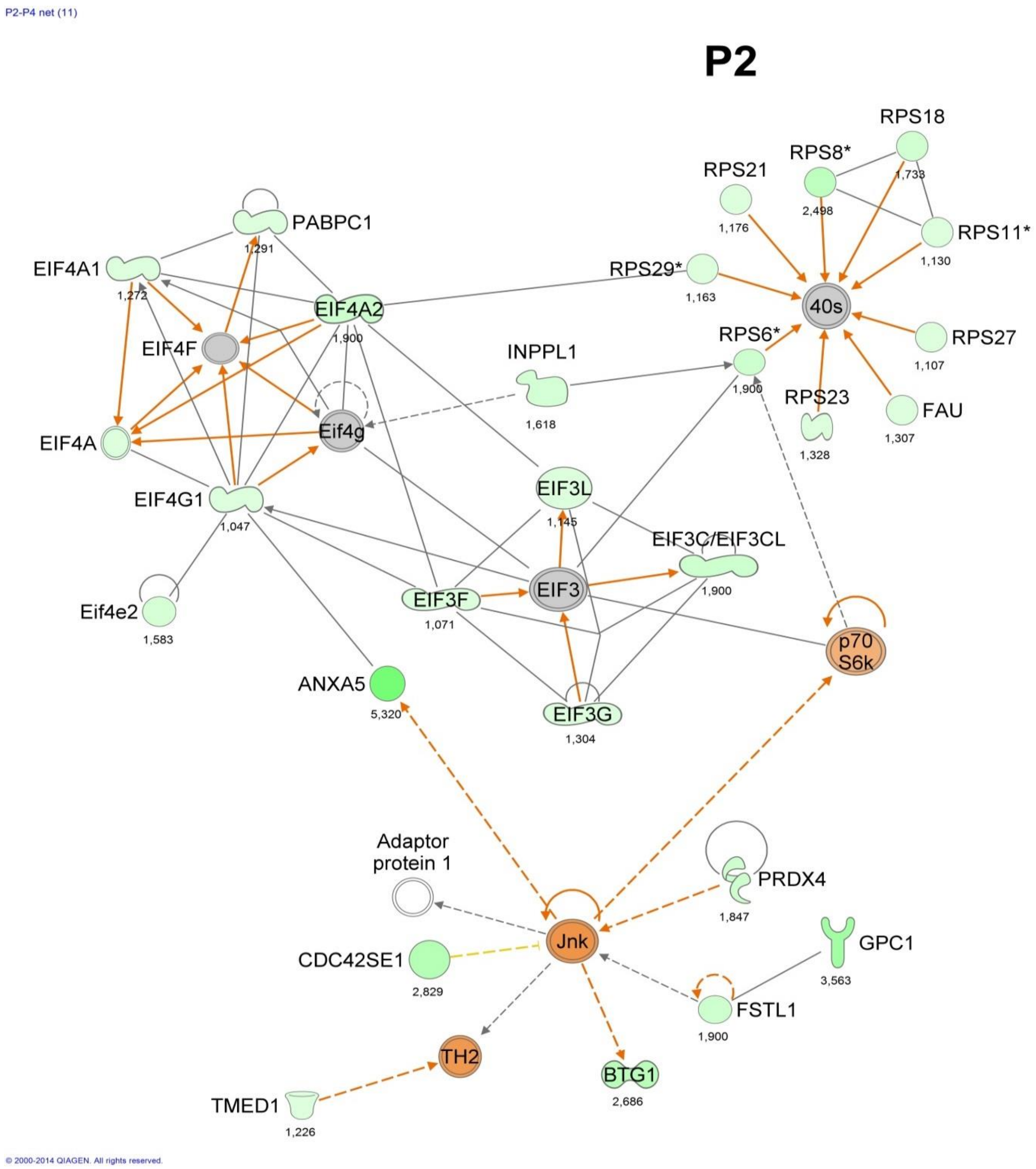

Network 7

Protein synthesis, gene expression, RNA post-transcriptional modification

P2-P4 net (12)

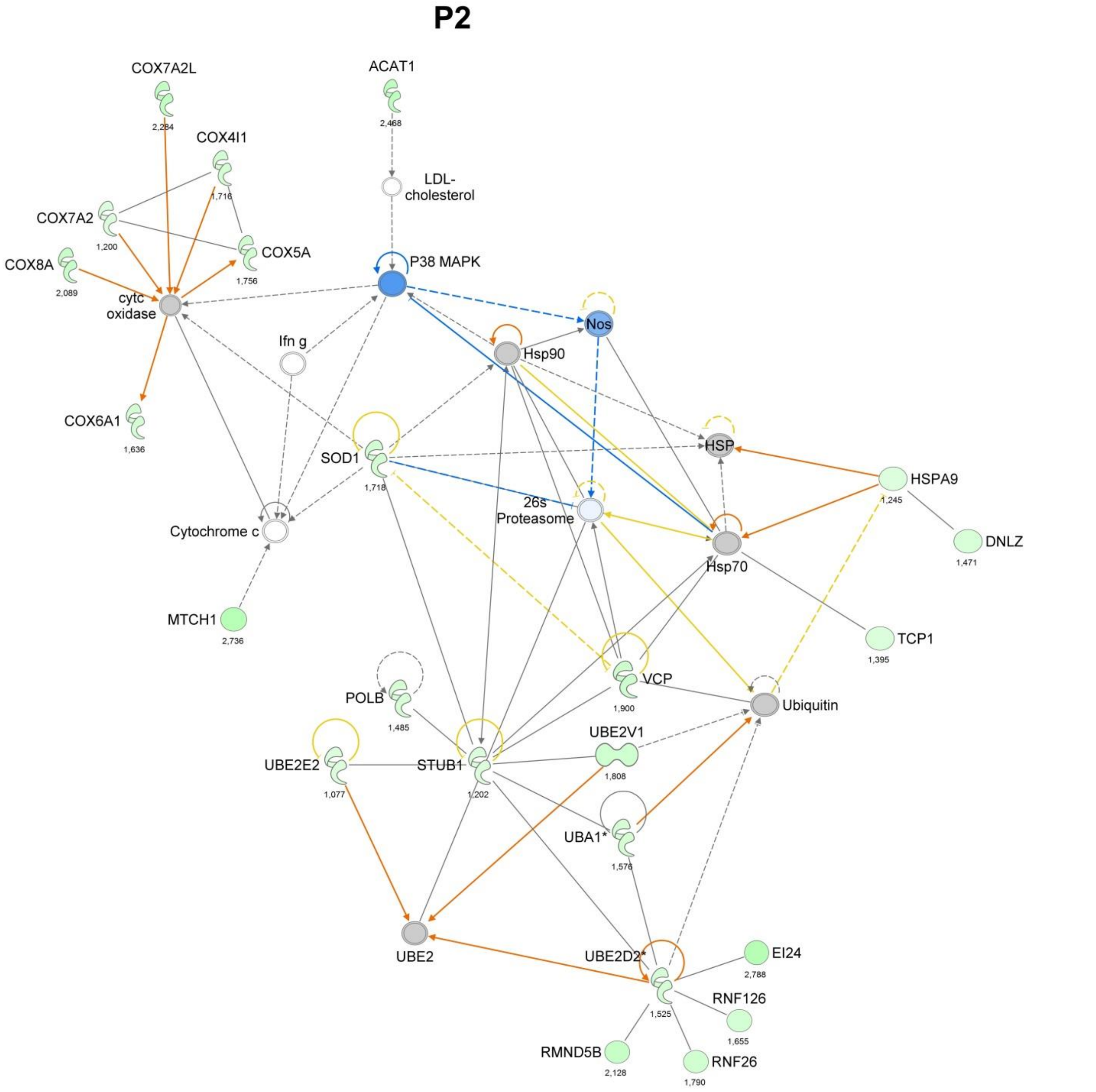

© 2000-2014 QIAGEN. All rights reserved.

Network 8

Post-transcriptional modification, cellular assembly and organization, cellular function and maintenance

P2-P4 net (13)

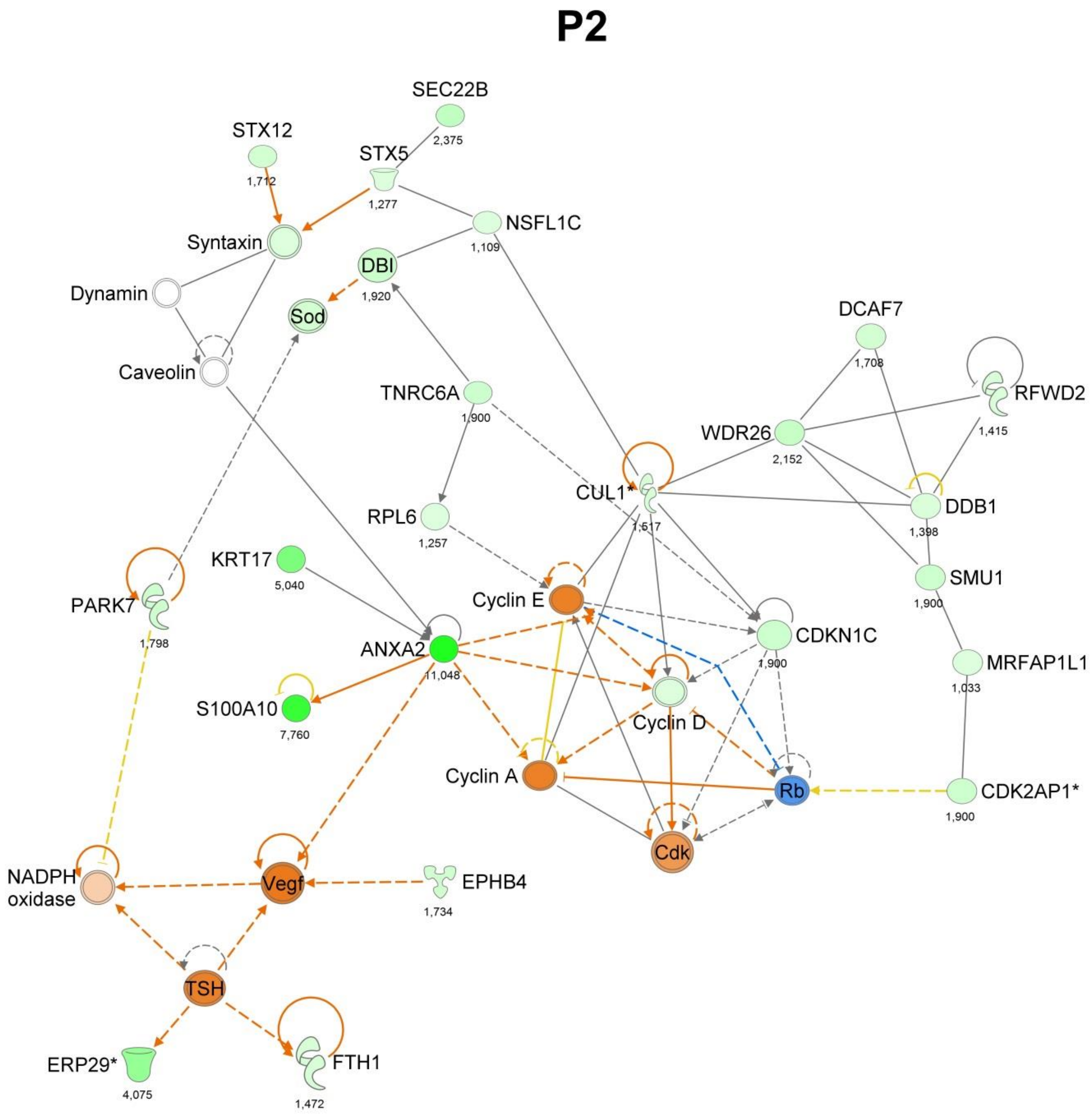

© 2000-2014 QIAGEN. All rights reserved.

Network 9

DNA replication

P2-P4 net(14)

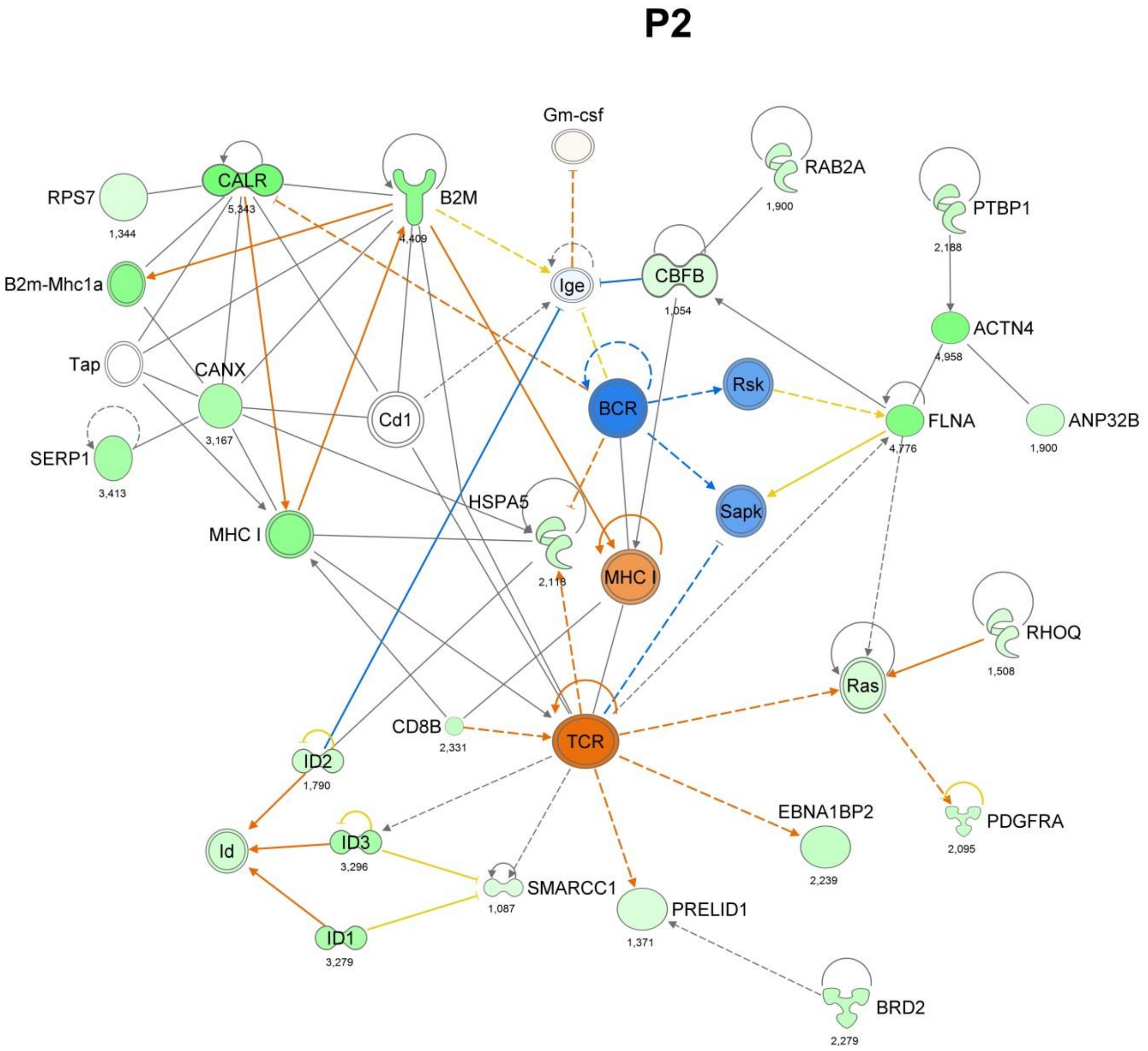

© 2000-2014 QIAGEN. All rights reserved.

Network 10

Gene expression, cellular development, cellular growth and proliferation

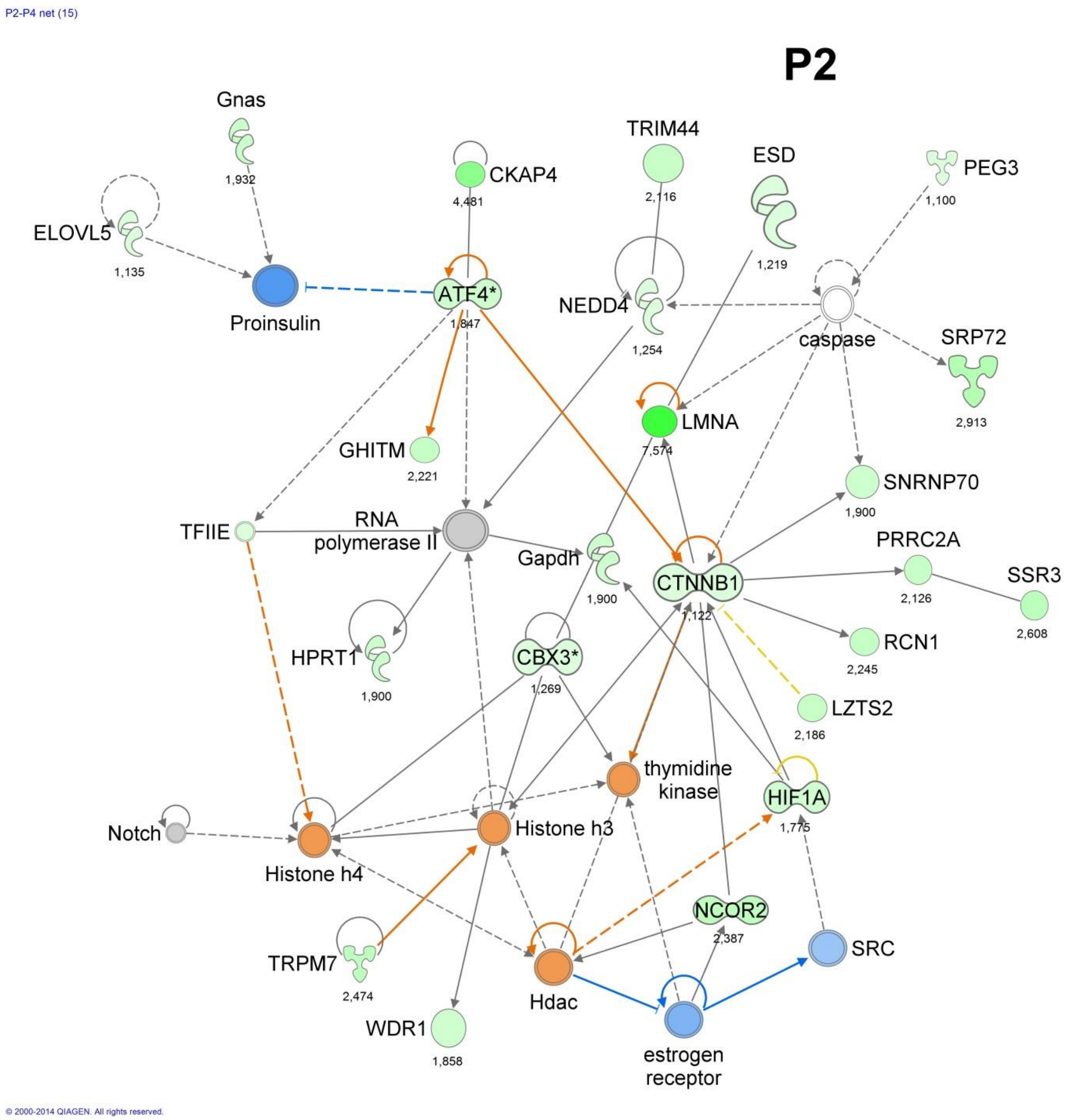

© 2000-2014 QIAGEN. All rights reserved.

Network 11

Cellular development, cellular growth and proliferation

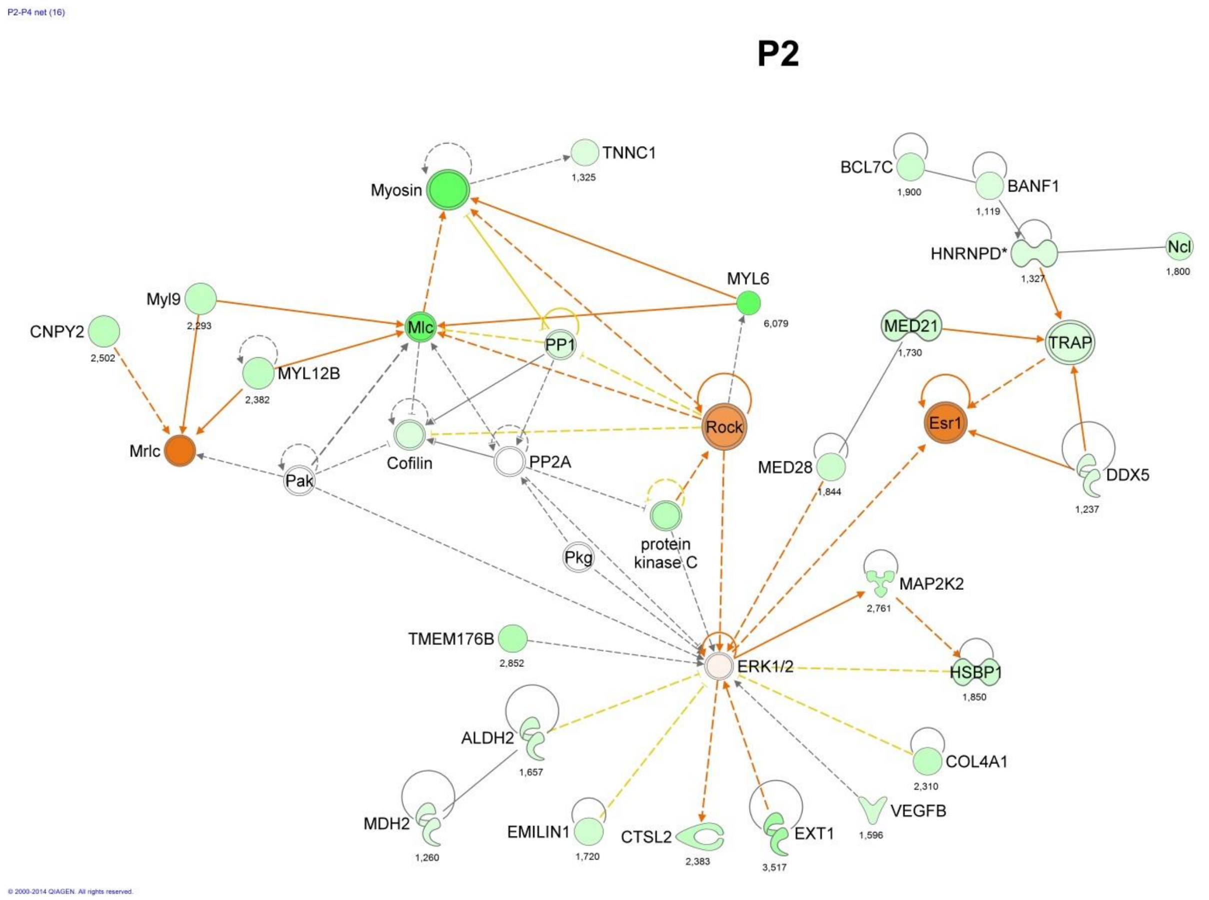

Network 12

Organ morphology

P2-P4 net (18)

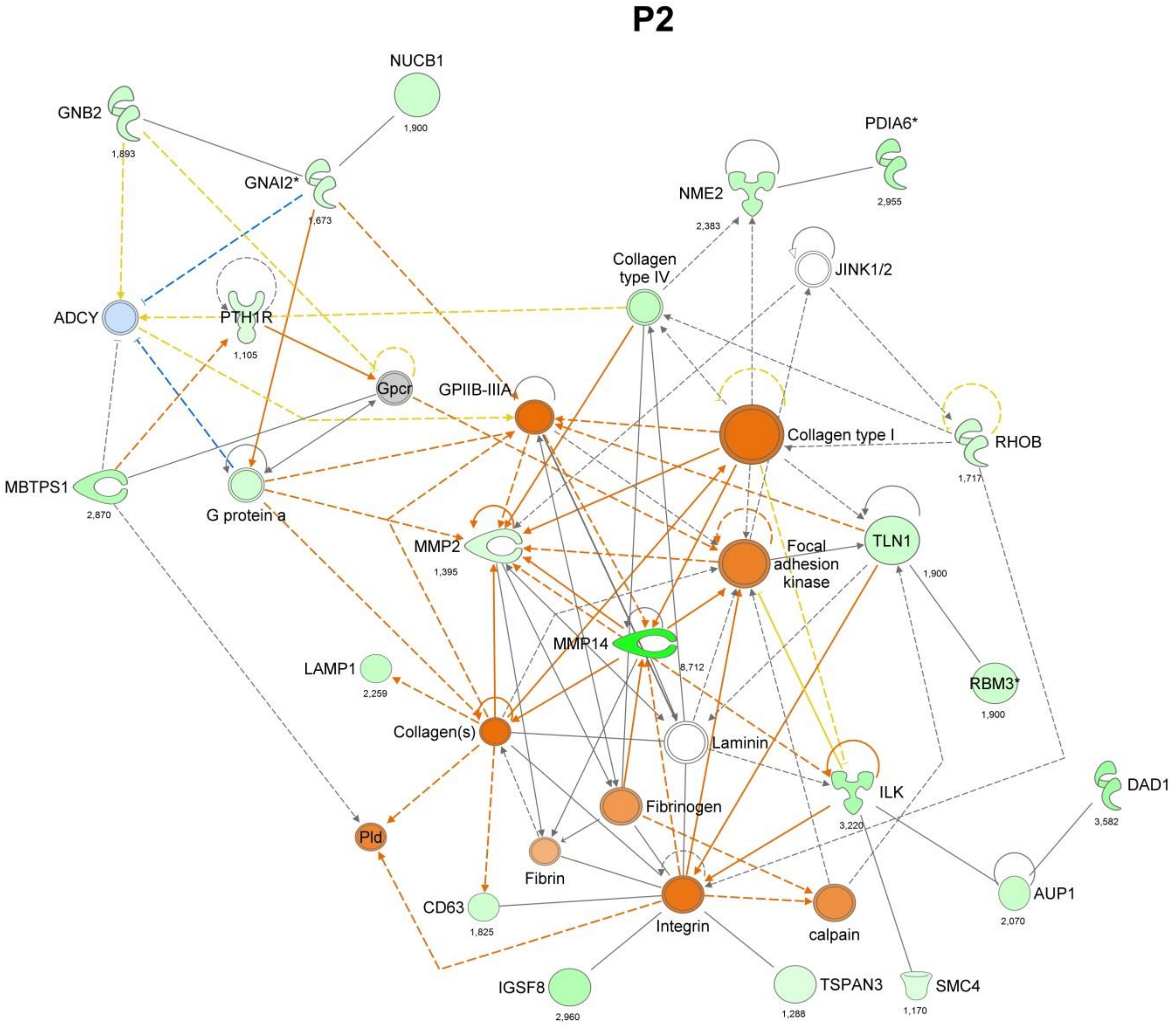

© 2000-2014 QIAGEN. All rights reserved.

Network 13

Embryonic development

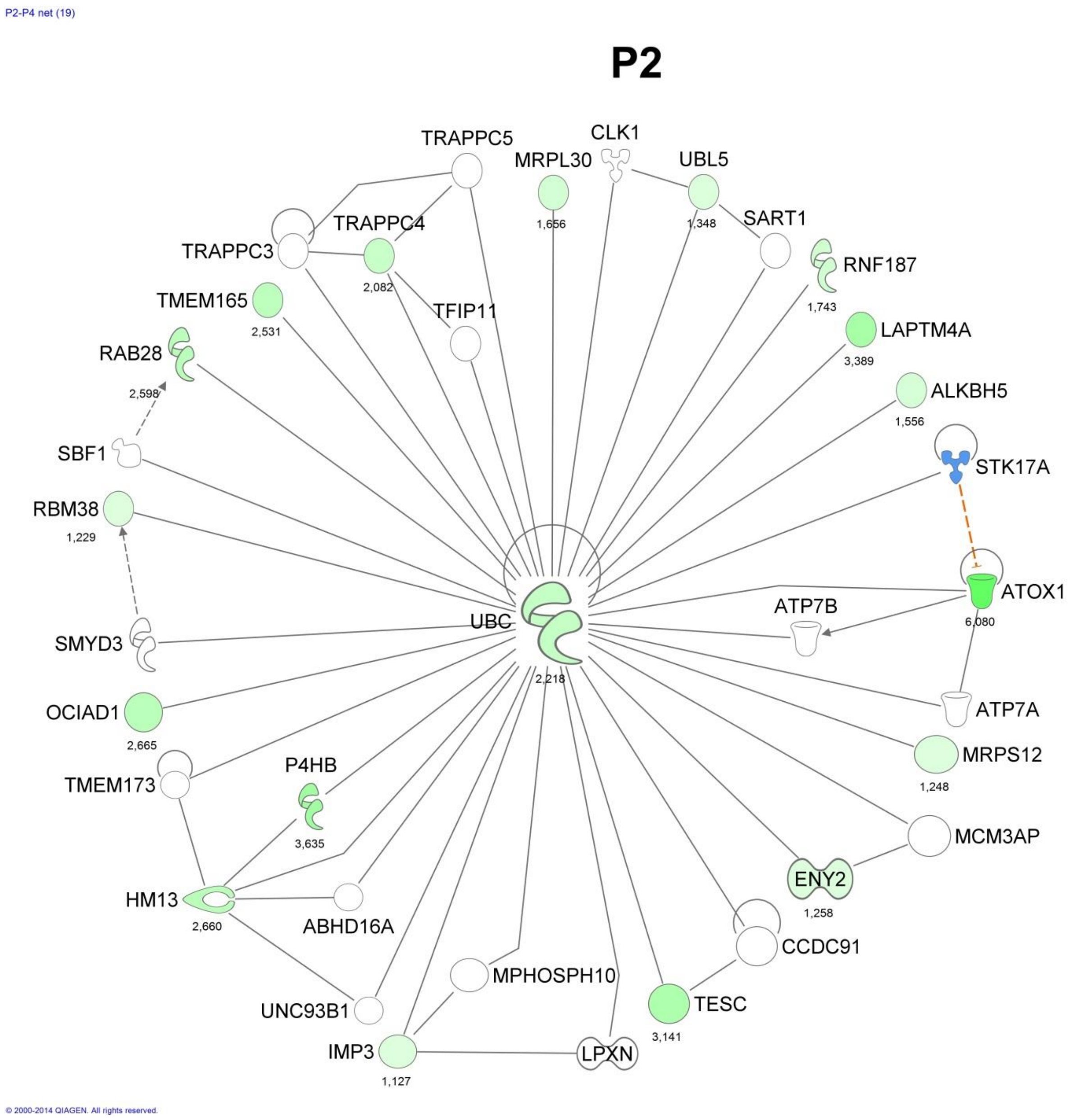

Network 14

Molecular transport, cellular function and maintenance, small molecule biochemistry

P2-P4 net (20)

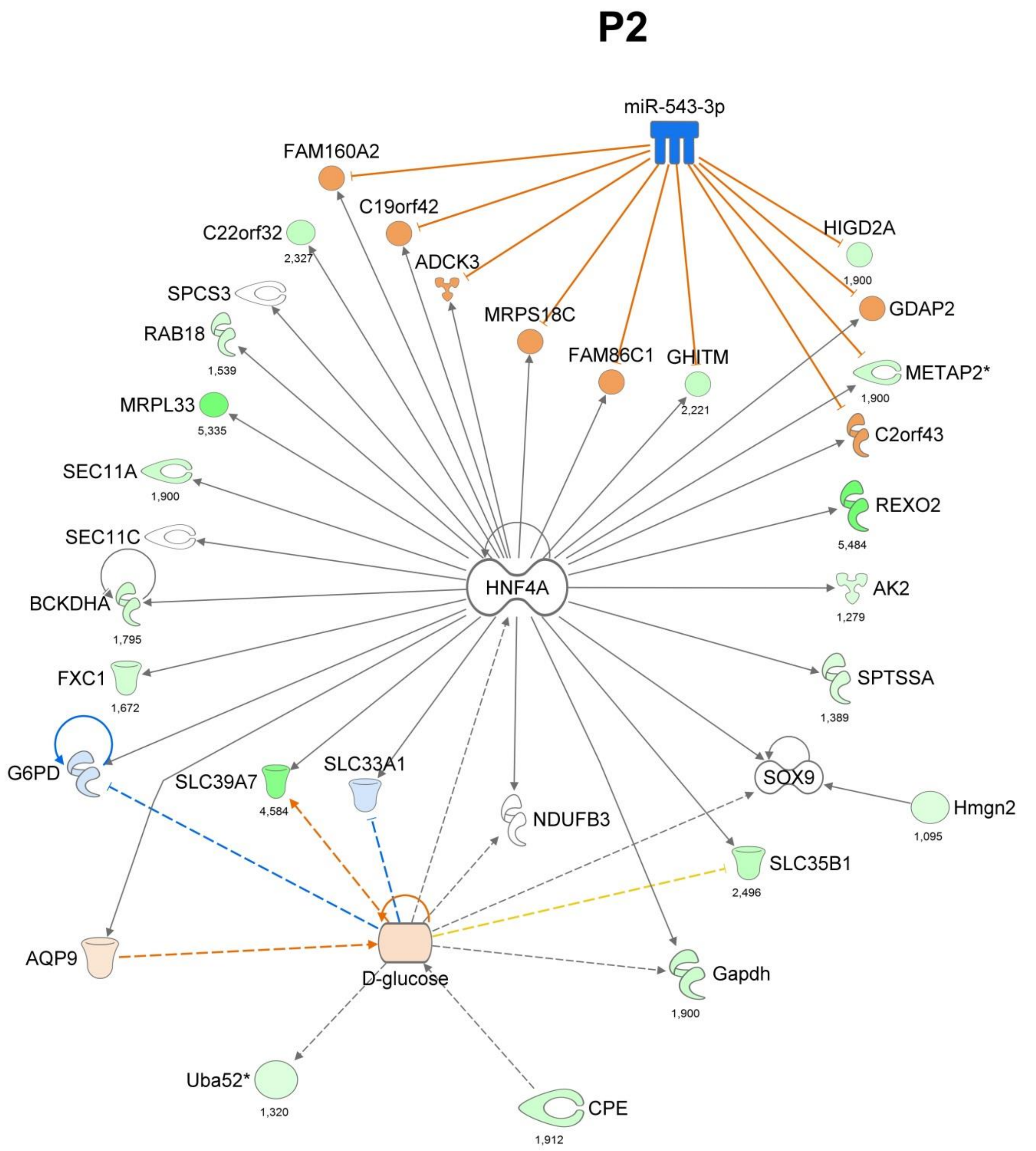

© 2000-2014 QIAGEN. All rights reserved.

Network 15

Nucleic acid metabolism, small molecule biochemistry, molecular transport

P2-P4 net (23)

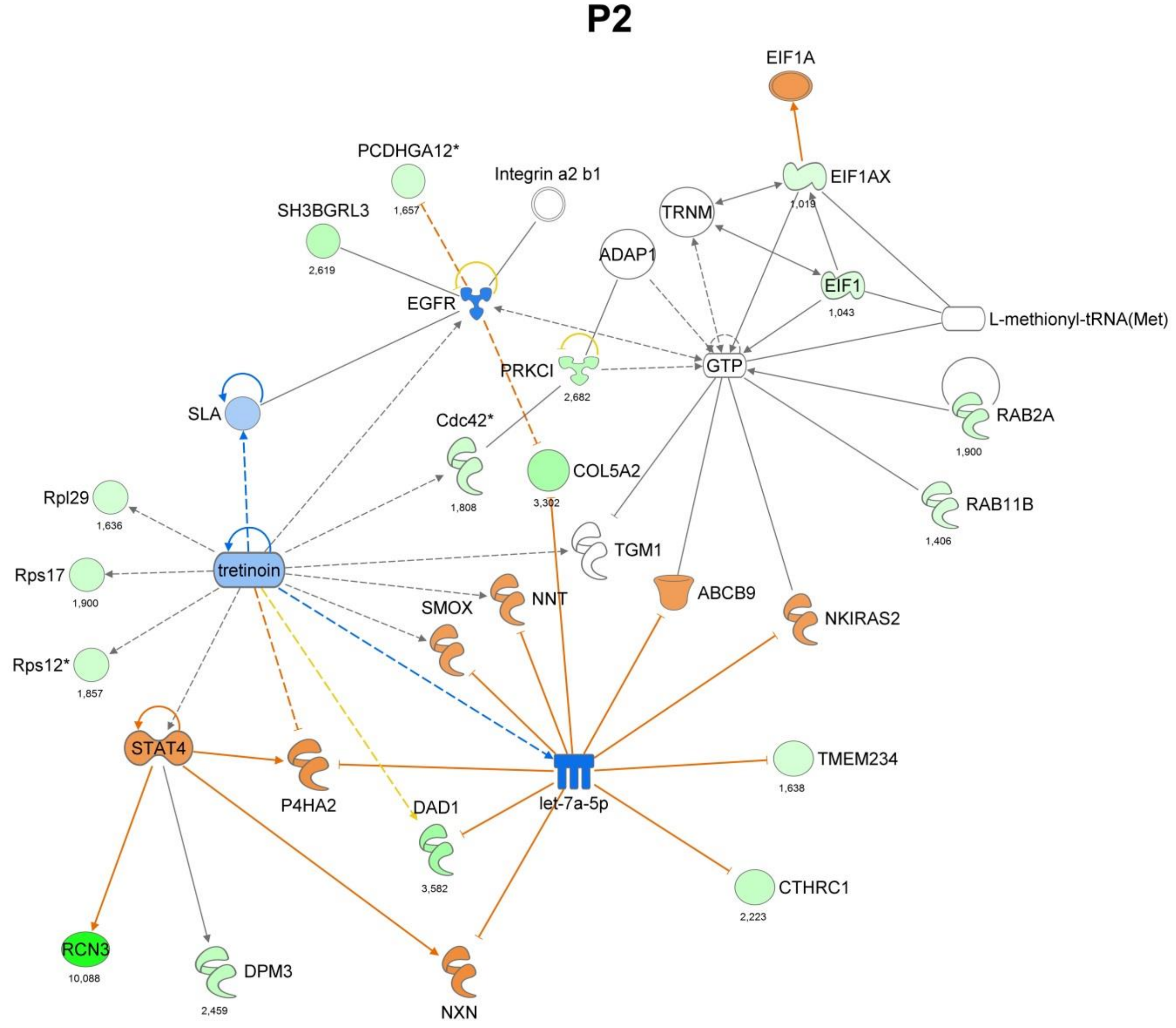

© 2000-2014 QIAGEN. All rights reserved.

Network 16

Protein synthesis, molecular transport, protein trafficking

P2-P4 net (2)**

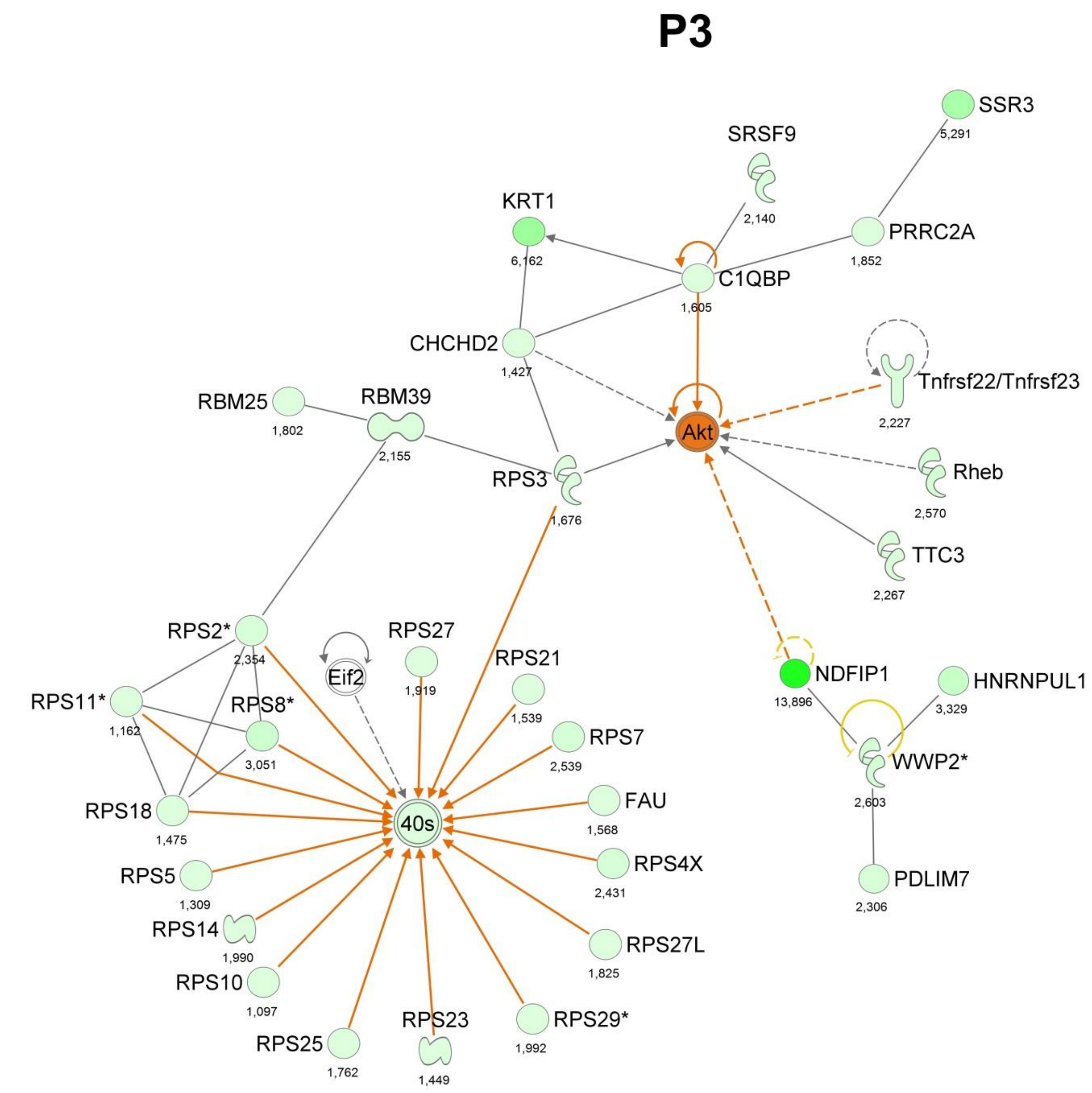

© 2000-2014 QIAGEN. All rights reserved.

Network 1

Gene expression, protein synthesis, small molecule biochemistry

P2-P4 net (3)*

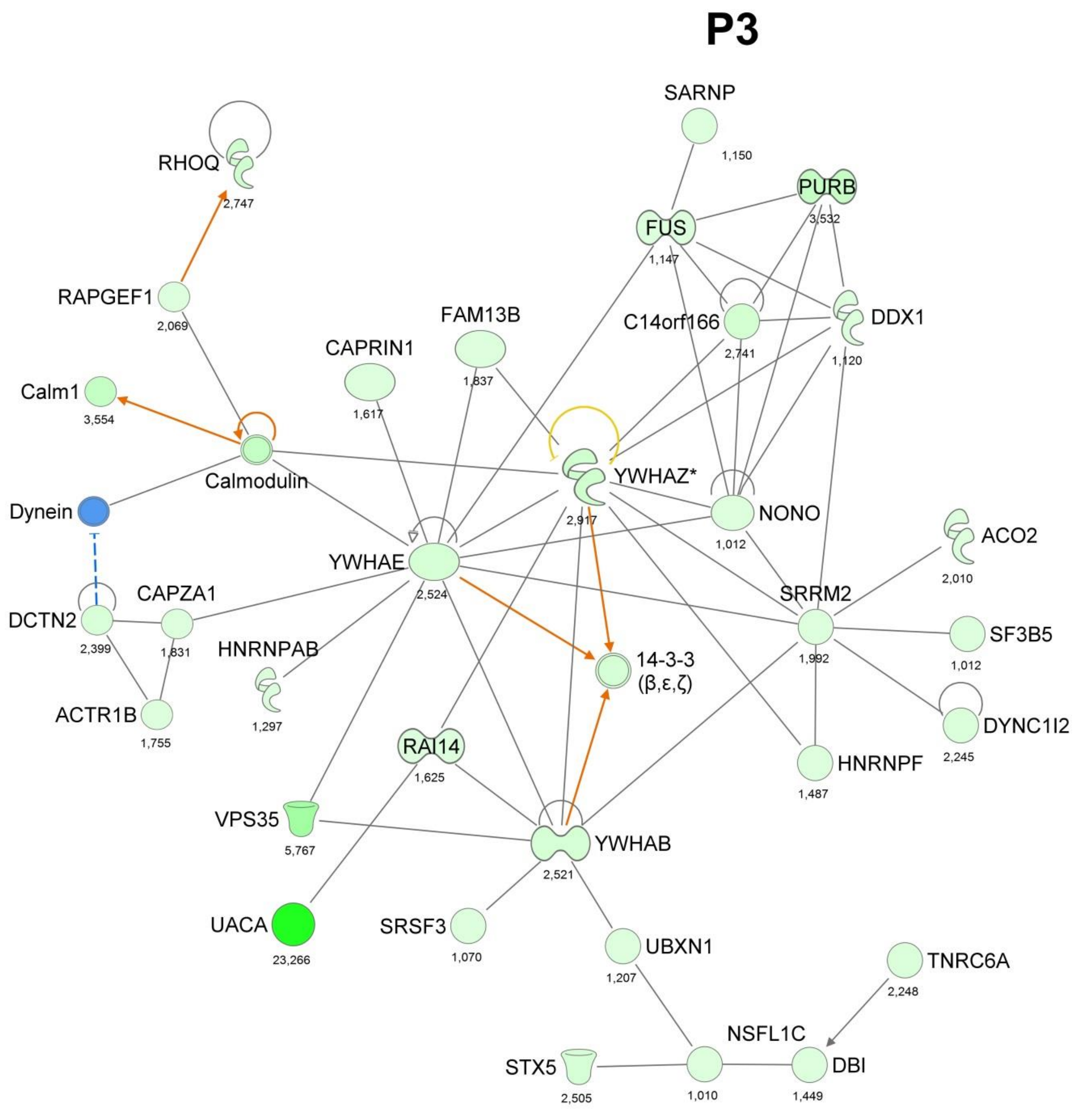

© 2000-2014 QIAGEN. All rights reserved.

Network 2

Cellular assembly and organization, cell cycle, cellular development

P2-P4 net (4)

P3

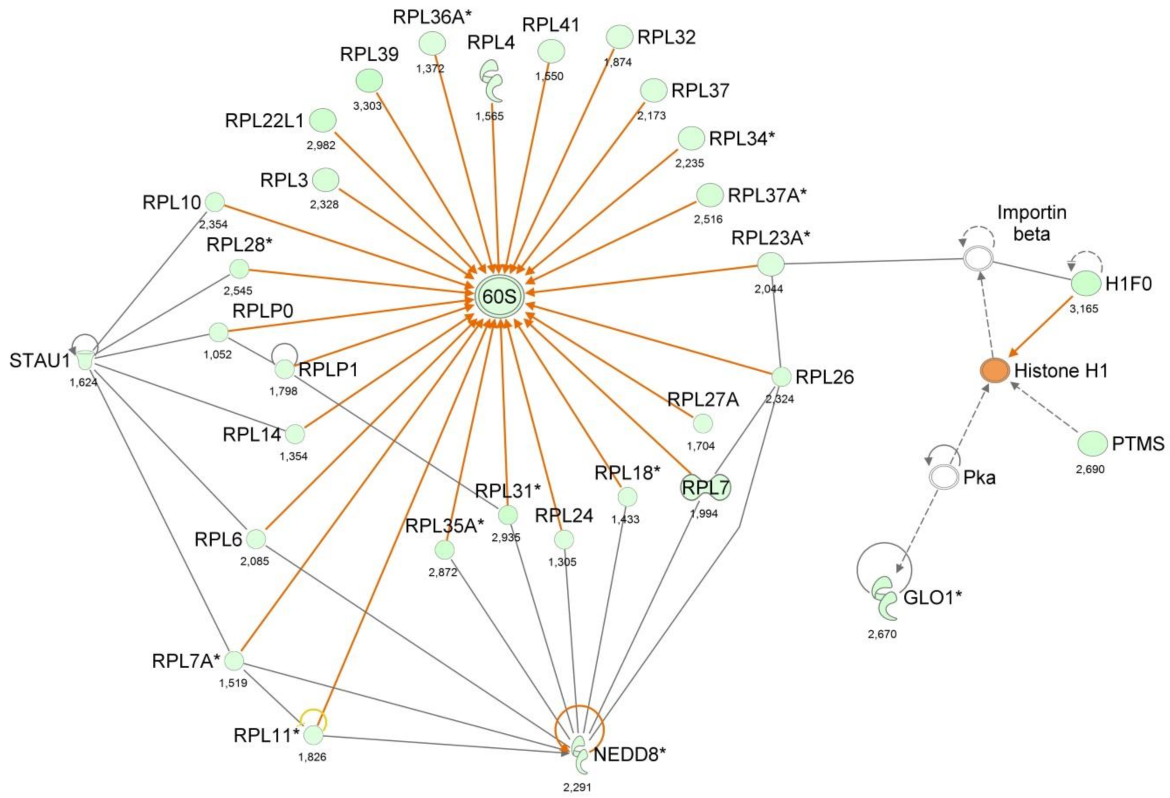

© 2000-2014 QIAGEN. All rights reserved.

Network 3

RNA post-transcriptional modification

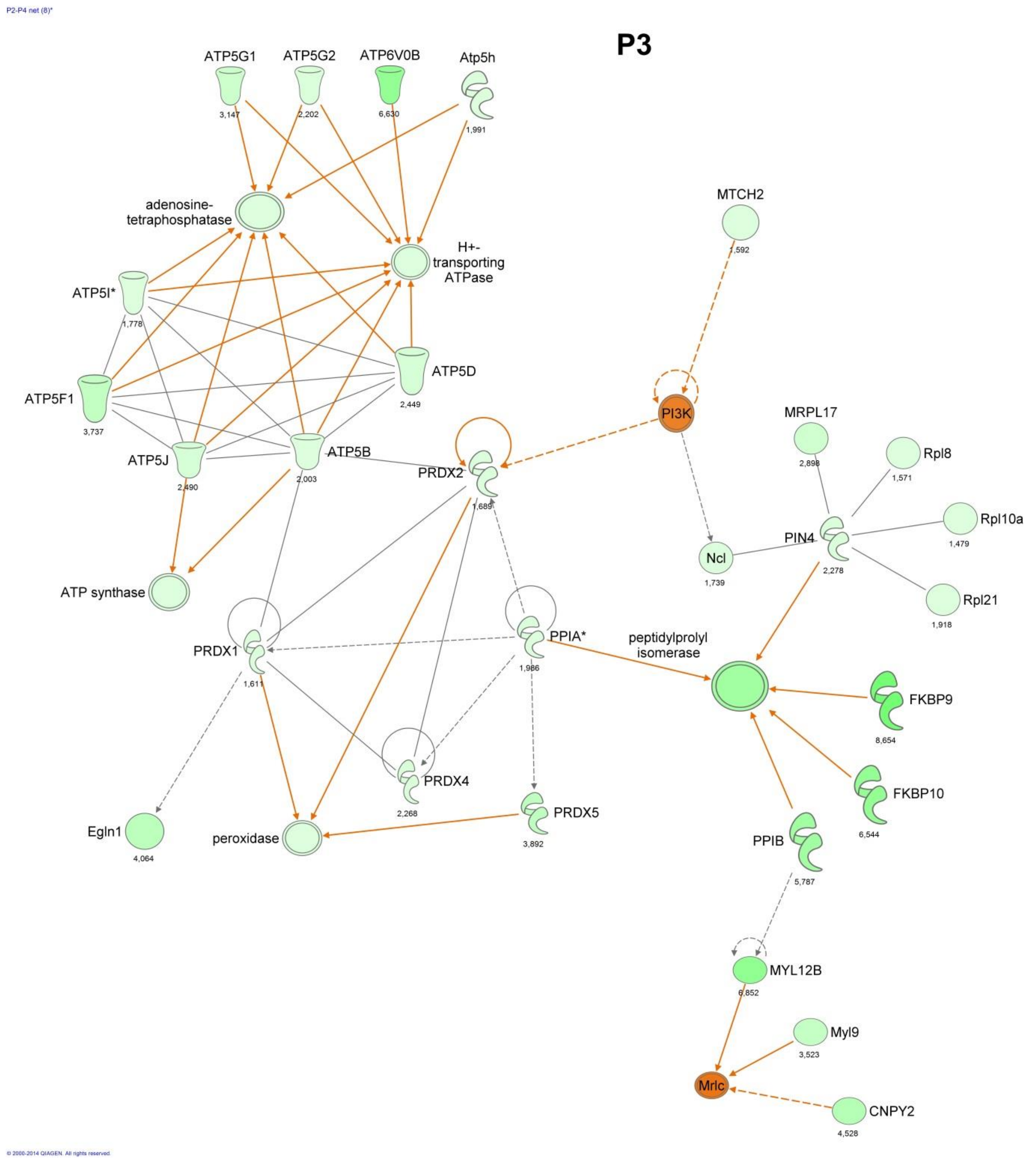

Network 4

Energy production, nucleic acid metabolism, small molecule biochemistry

P2-P4 net (10)*

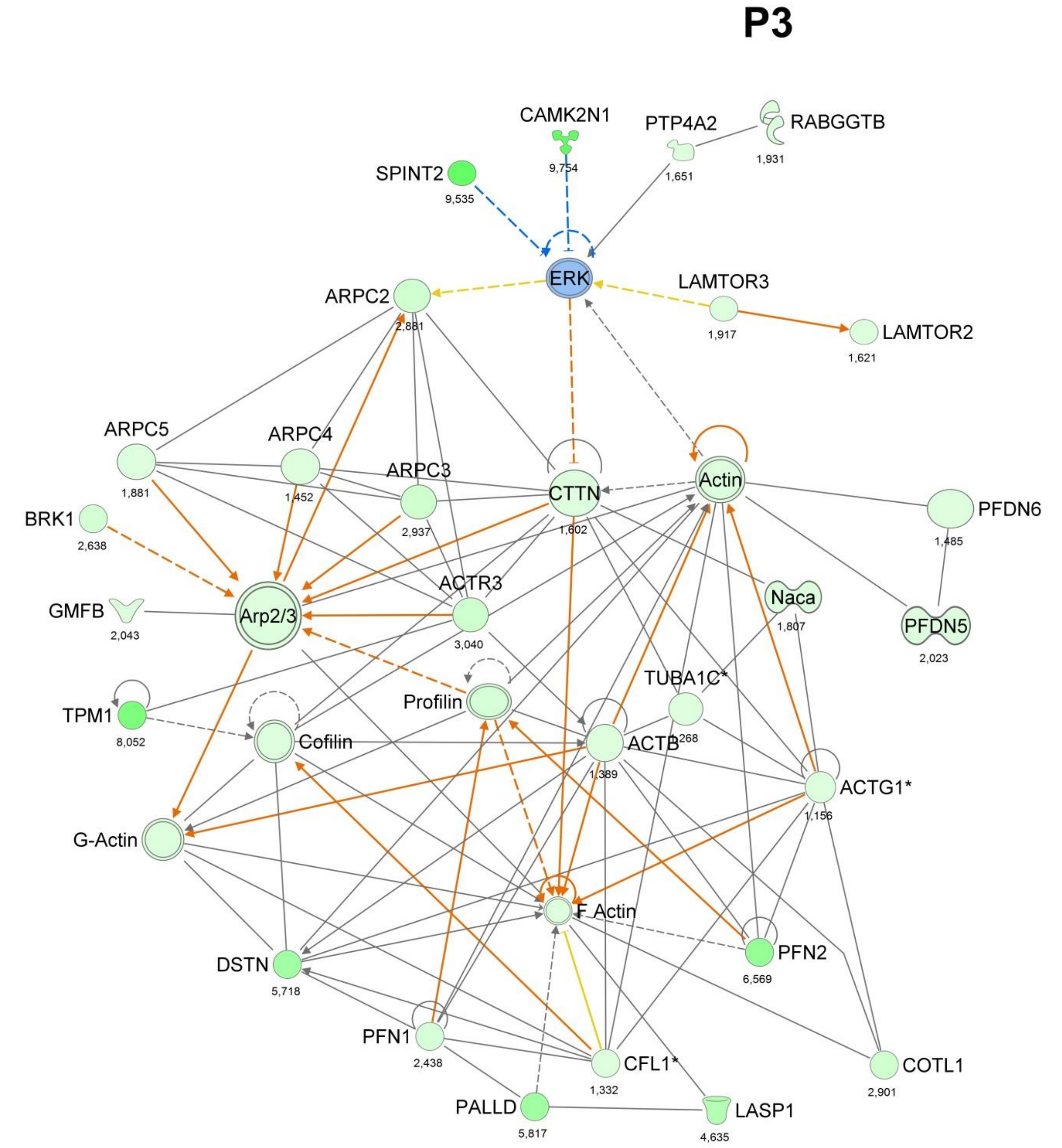

© 2000-2014 QIAGEN. All rights reserved.

Network 5

Cellular assembly and organization, cellular function and maintenance, tissue development

P2-P4 net (12)*

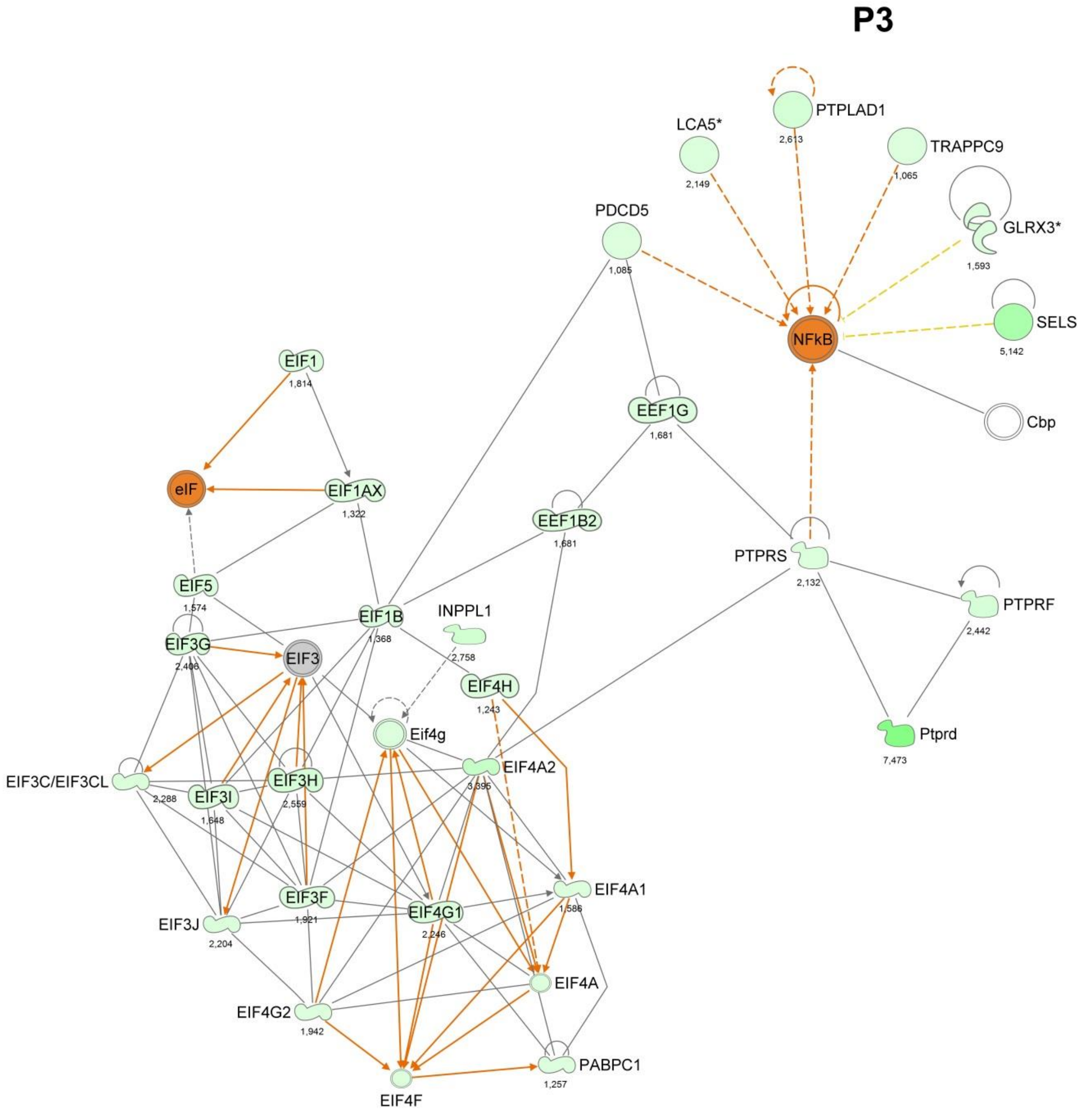

© 2000-2014 QIAGEN. All rights reserved.

Network 6

Gene expression, protein synthesis

P2-P4 net (13)*

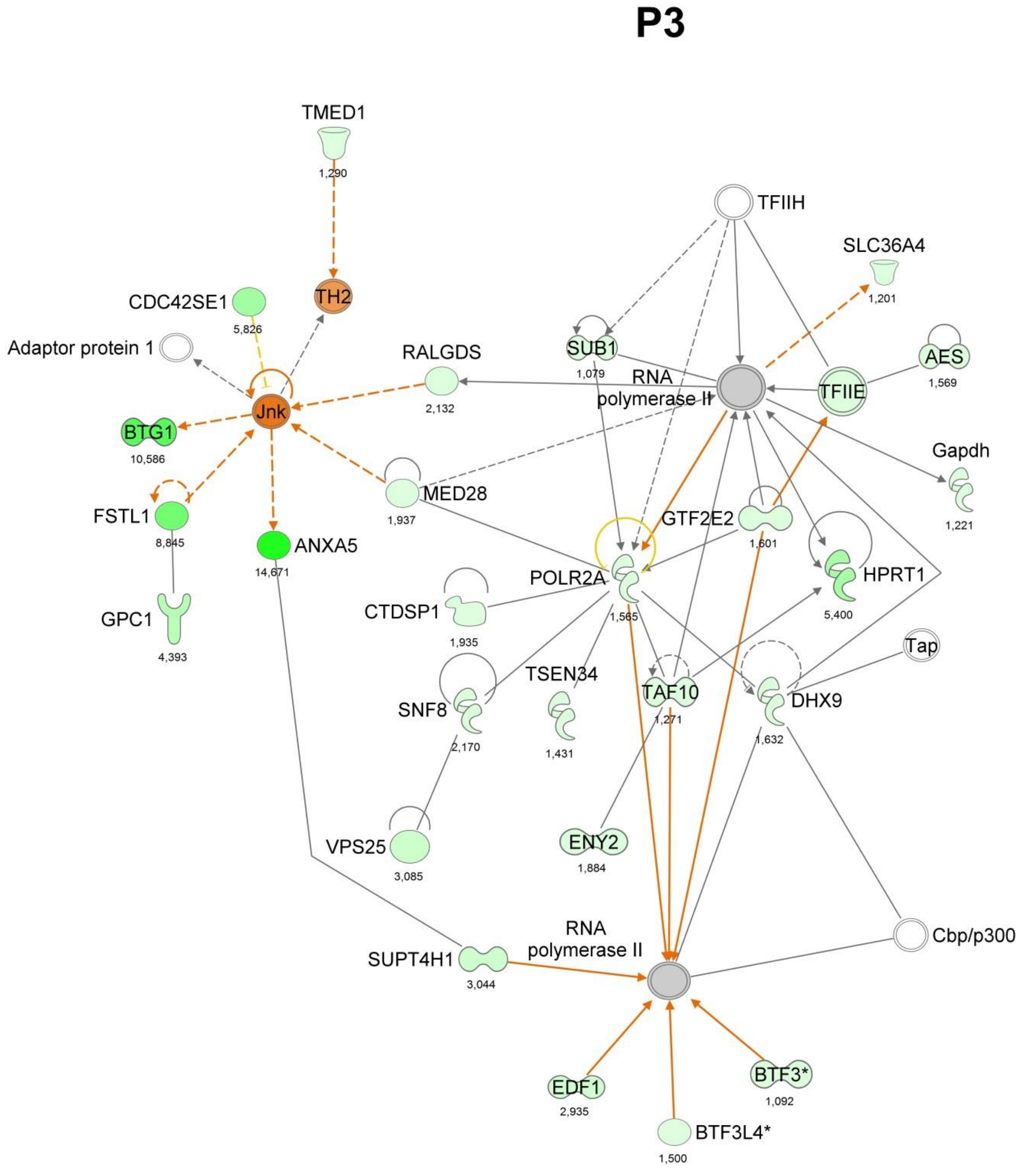

© 2000-2014 QIAGEN. All rights reserved.

Network 7

Gene expression cardiovaascular development, cell cycle

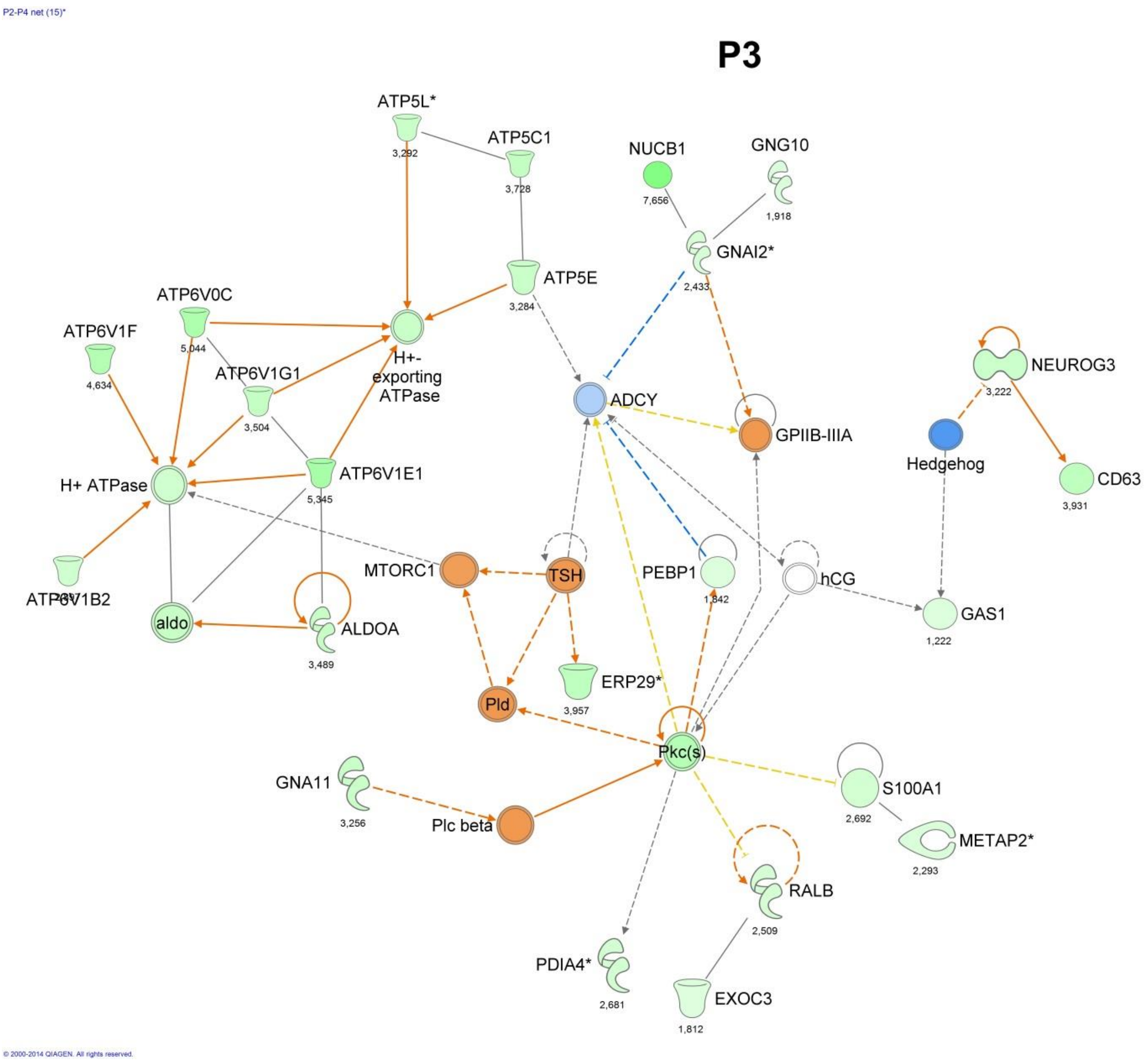

Network 8

Molecular transport, DNA replication, energy production

P2-P4 net (16)*

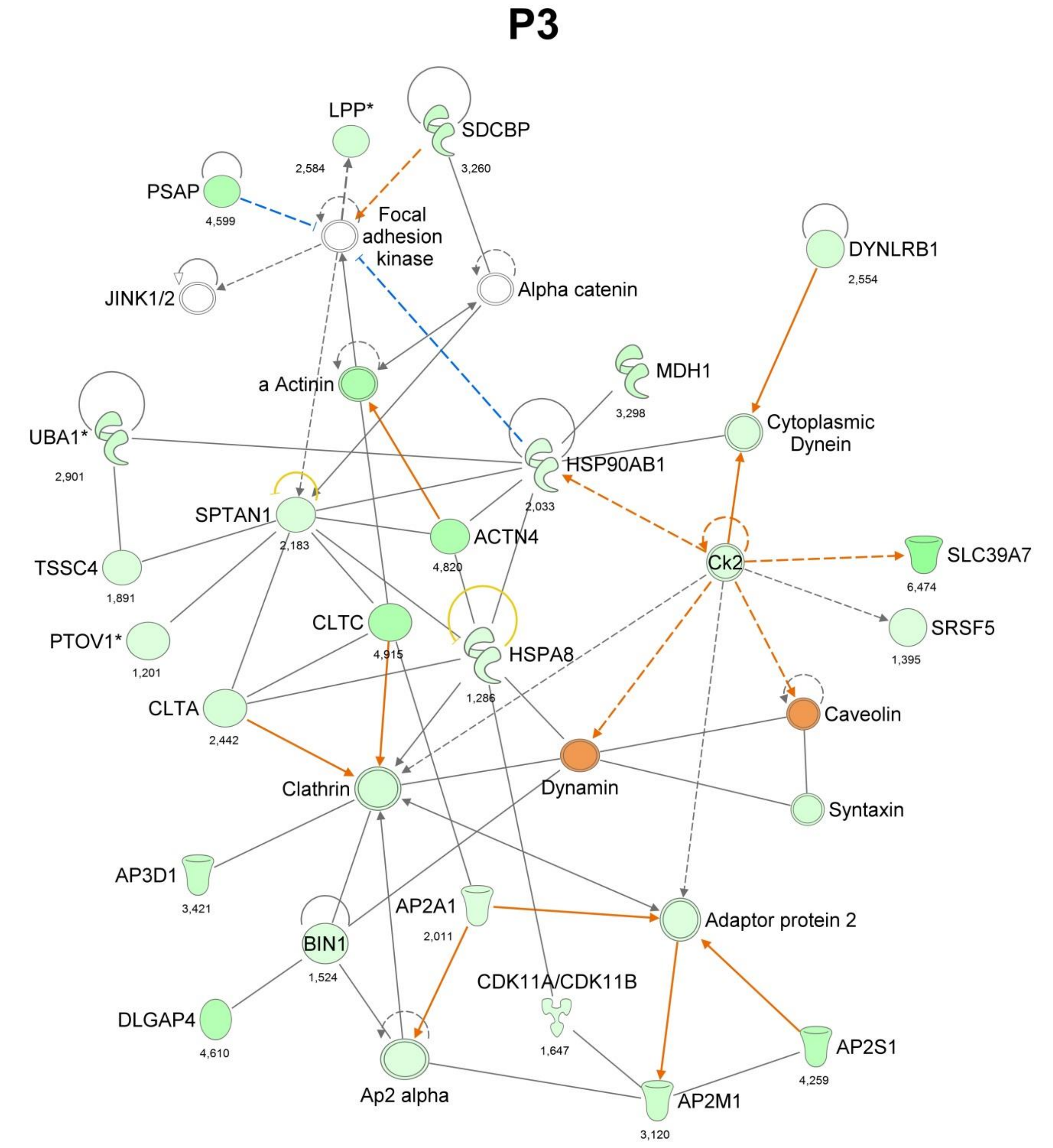

© 2000-2014 QIAGEN. All rights reserved.

Network 9

Cellular function and maintenance

P2-P4 net (17)*

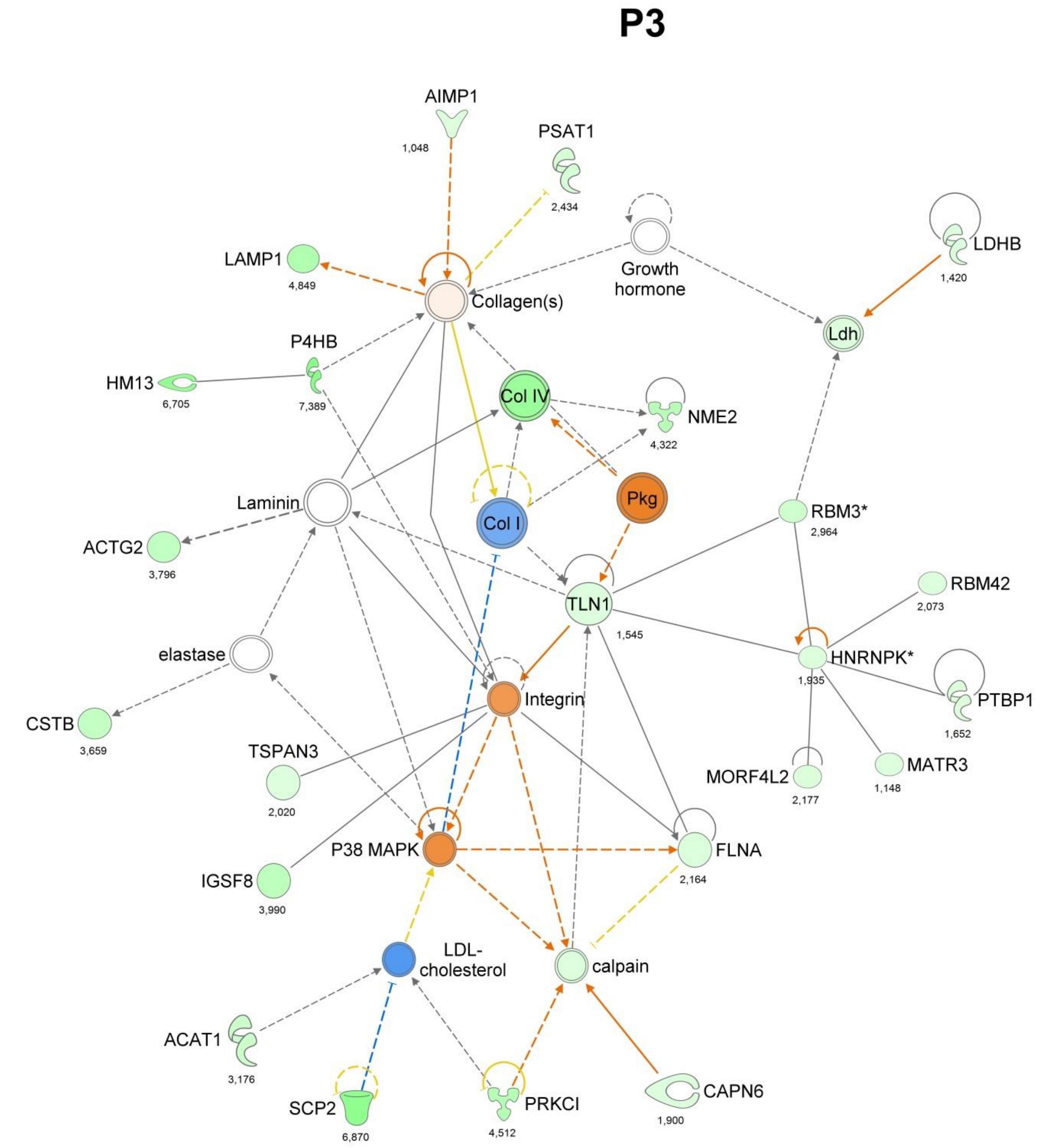

© 2000-2014 QIAGEN. All rights reserved.

Network 10

Lipid metabolism, molecular transport, small molecule biochemistry

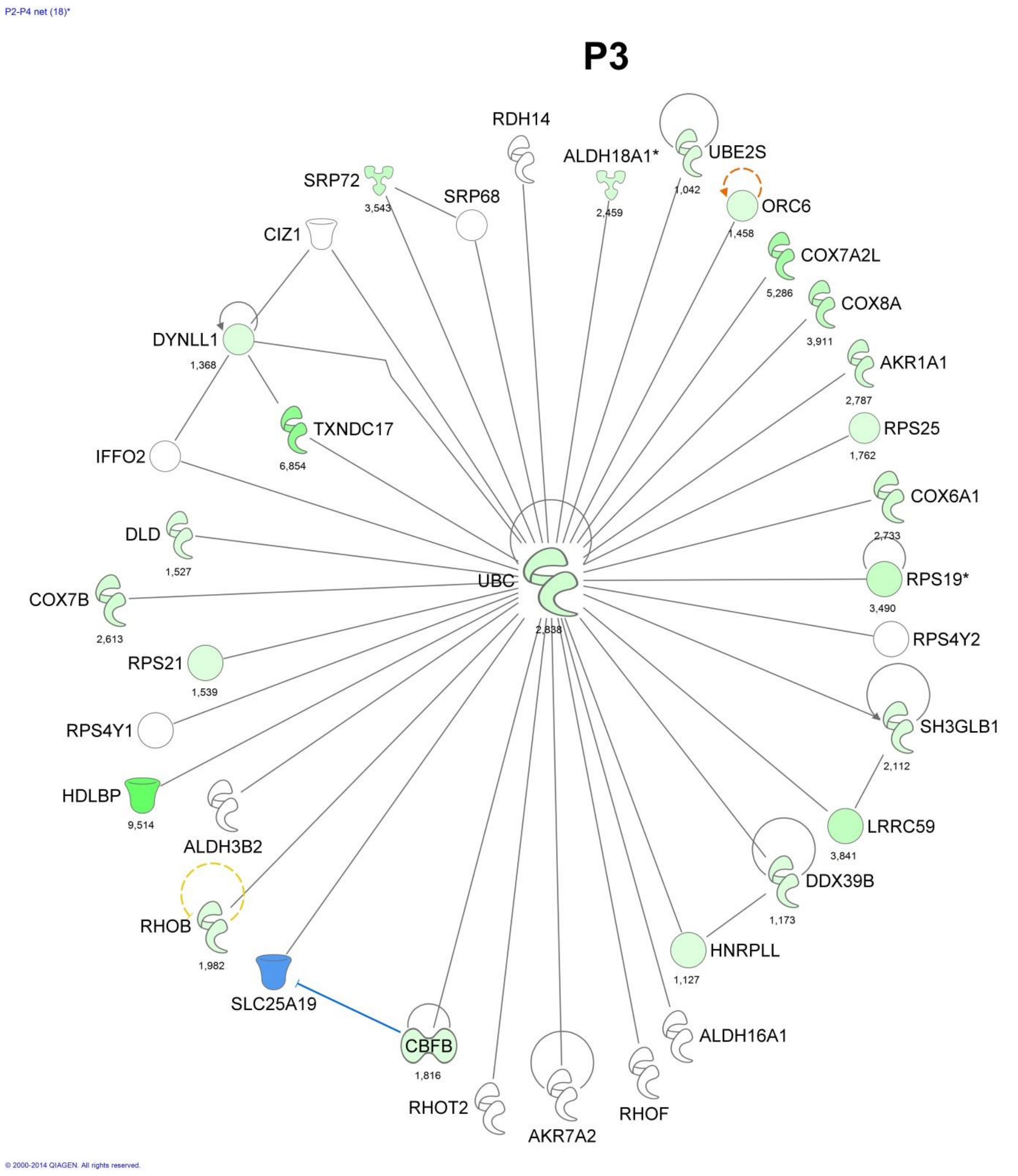

Network 11

Small molecule biochemistry, molecular transport

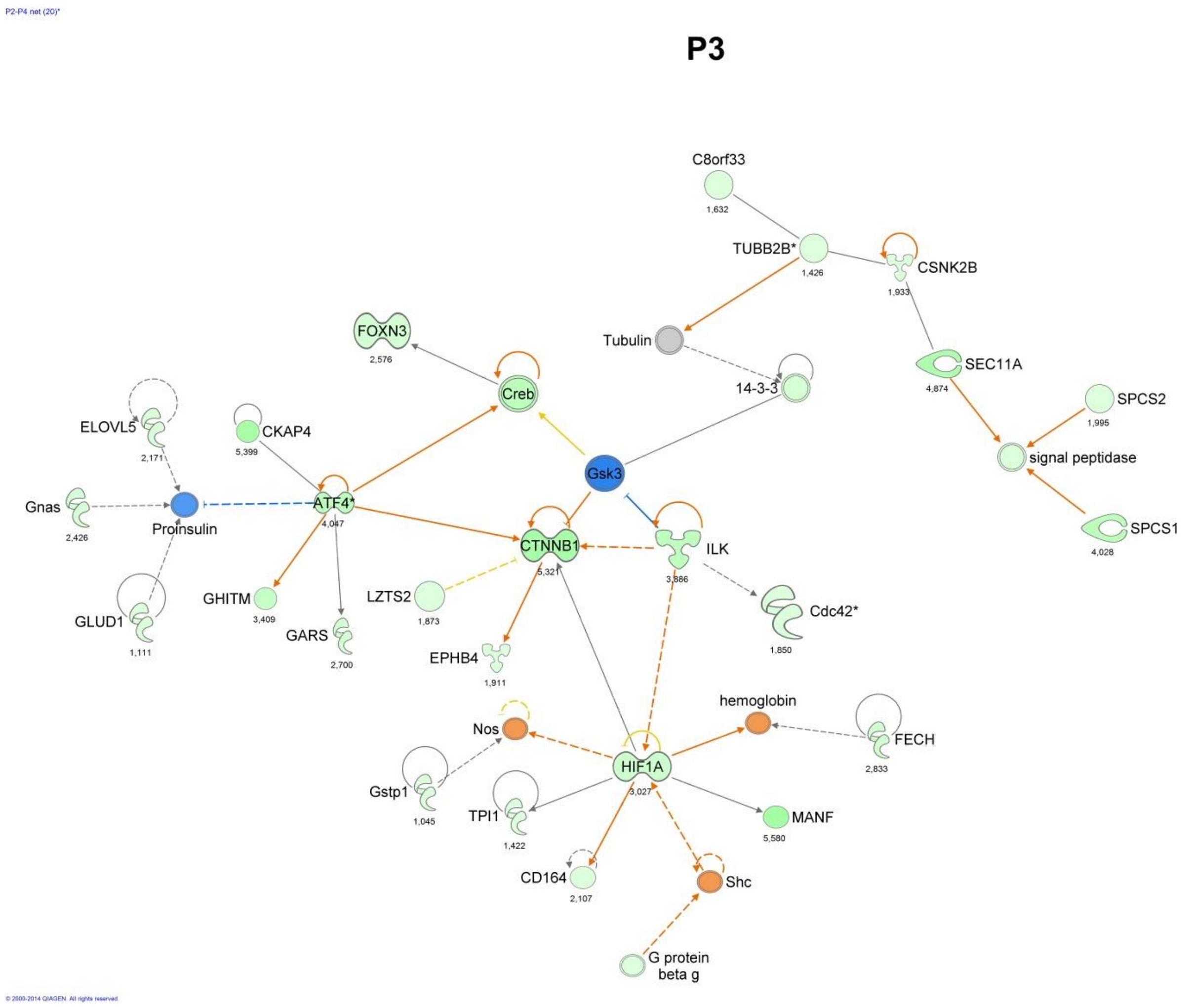

© 2000-2014 QIAGEN. All rights reserved.

Network 12

Cellular development, cellular growth and proliferation, connective tissue development

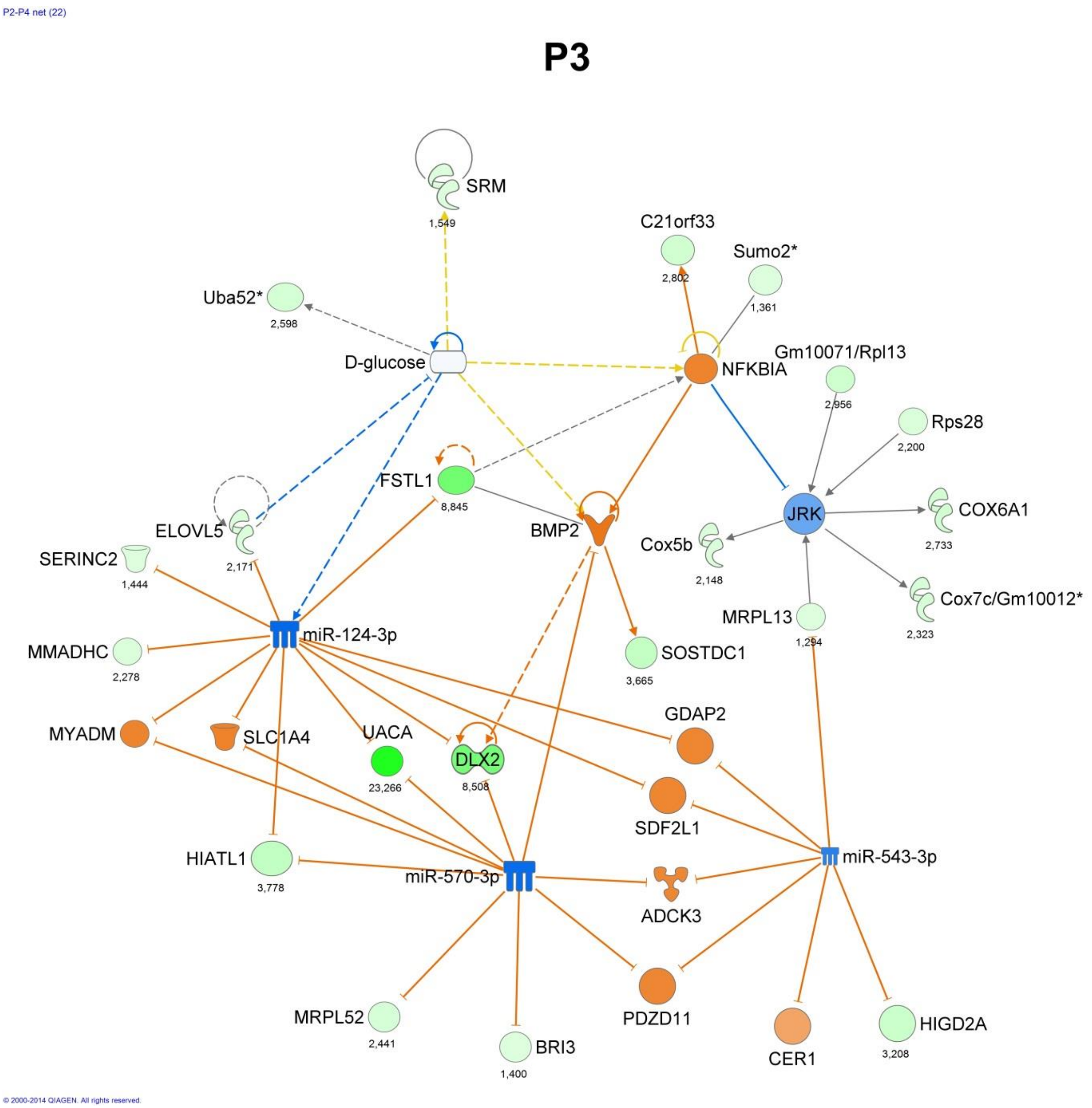

© 2000-2014 QIAGEN. All rights reserved.

Network 13

Amino acid metabolism, molecular transport, small molecule biochemistry

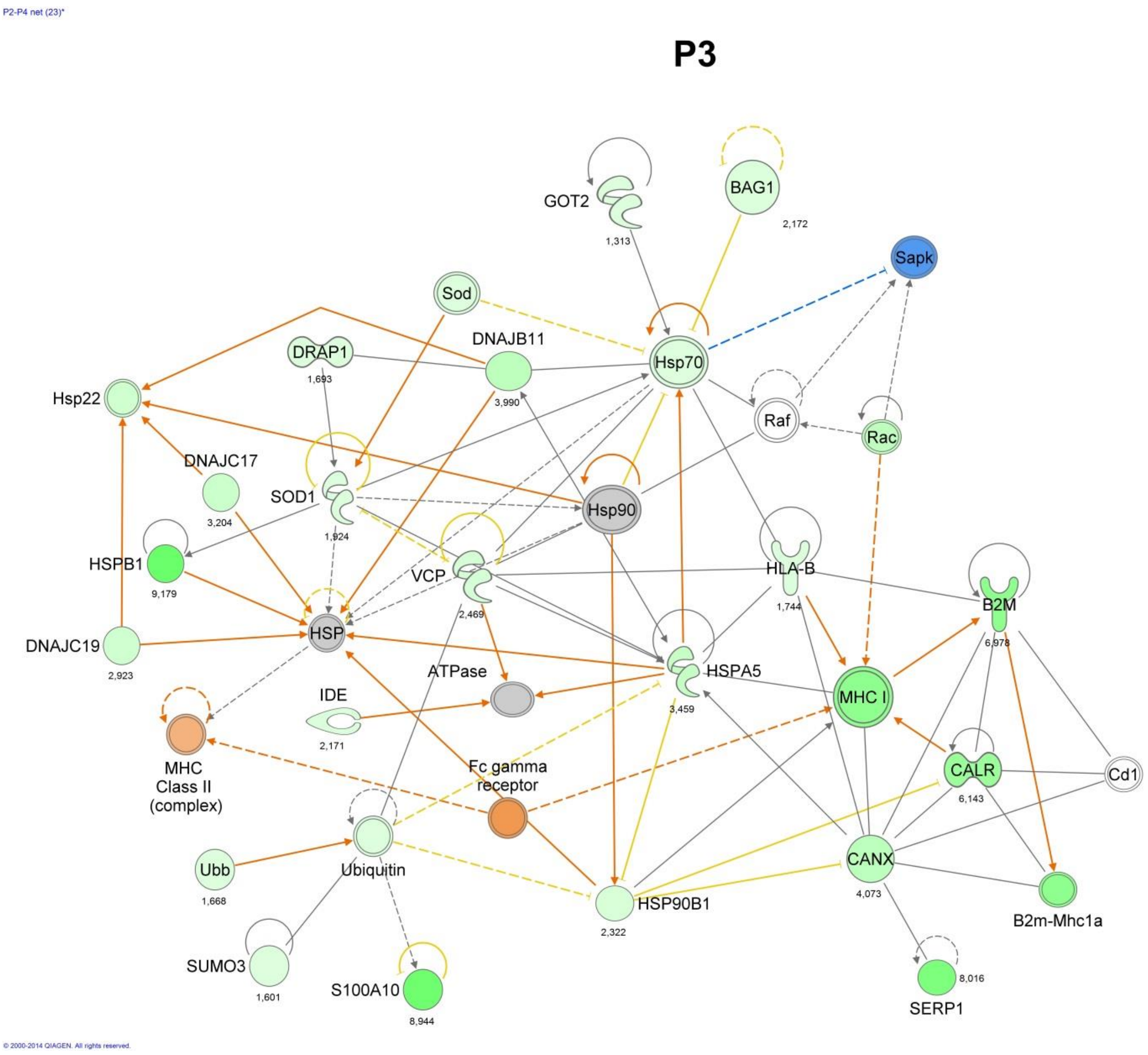

Network 14

Post-transcriptional modification, protein folding, cellular compromise

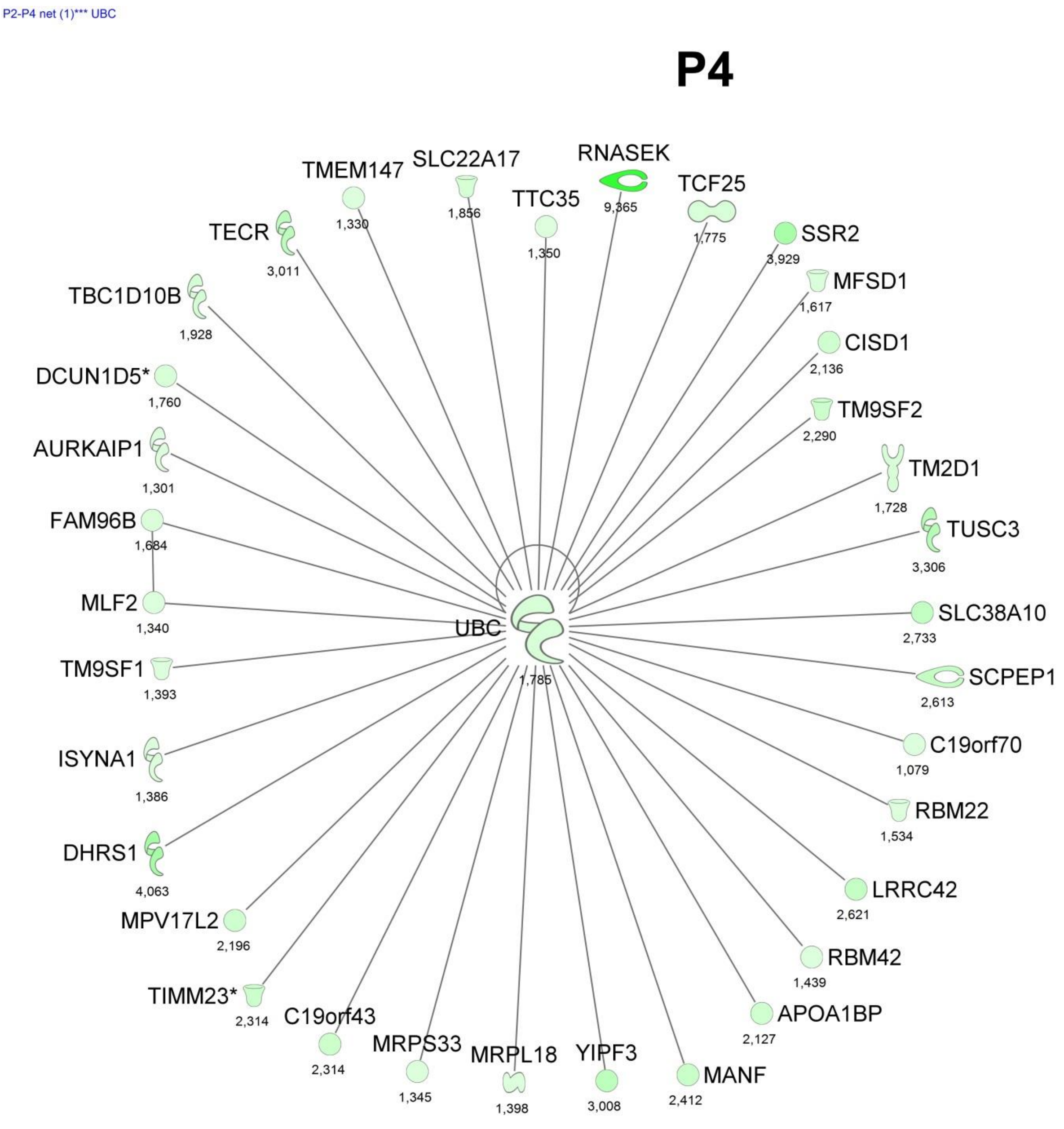

© 2000-2014 QIAGEN. All rights reserved.

Network 1

Lipid metabolism, nucleic acid metabolism, smal molecule biochemistry

P2-P4 net (3)**

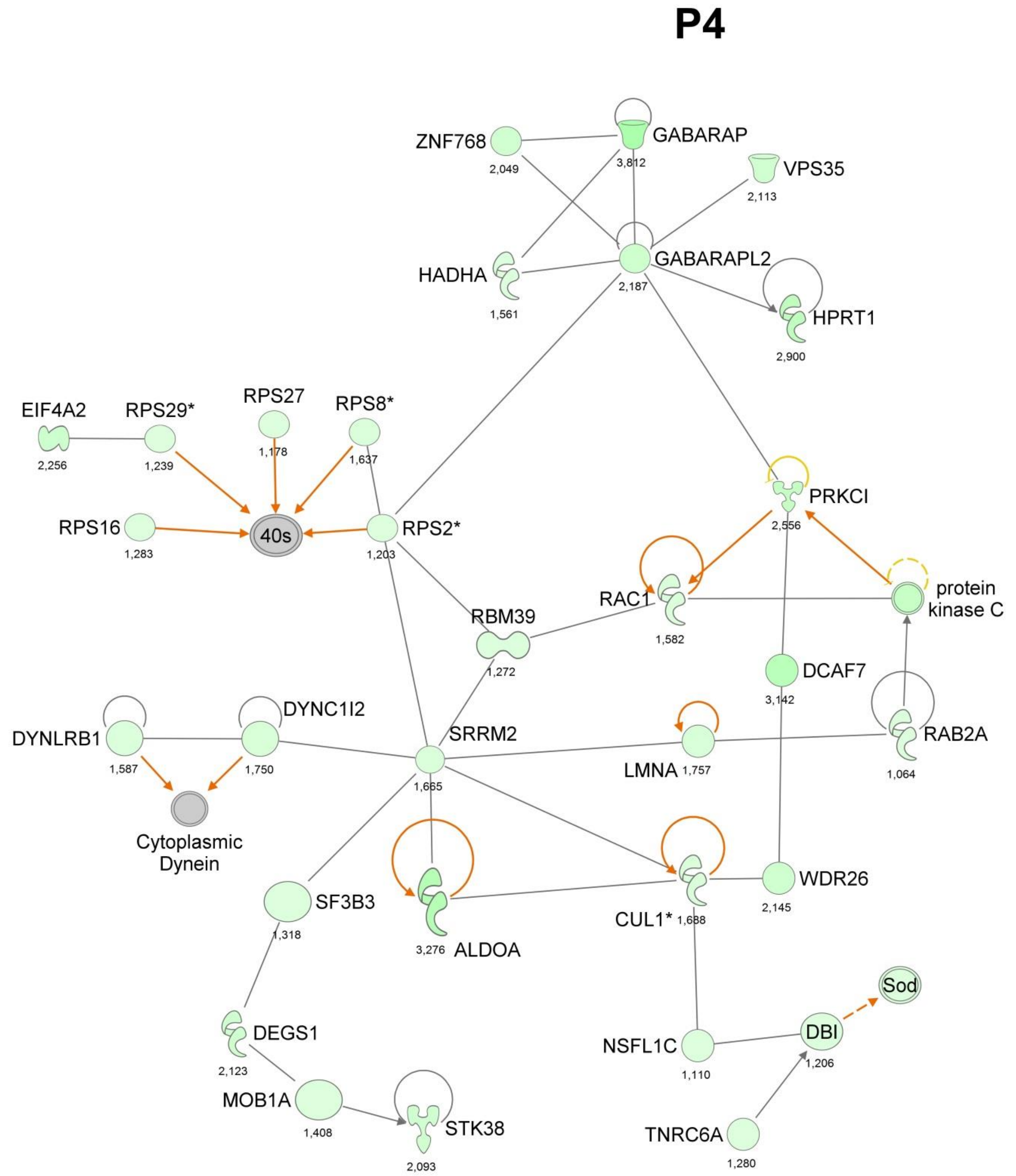

© 2000-2014 QIAGEN. All rights reserved.

Network 2

Molecular transport, RNA trafficking, carbohydrate metabolism

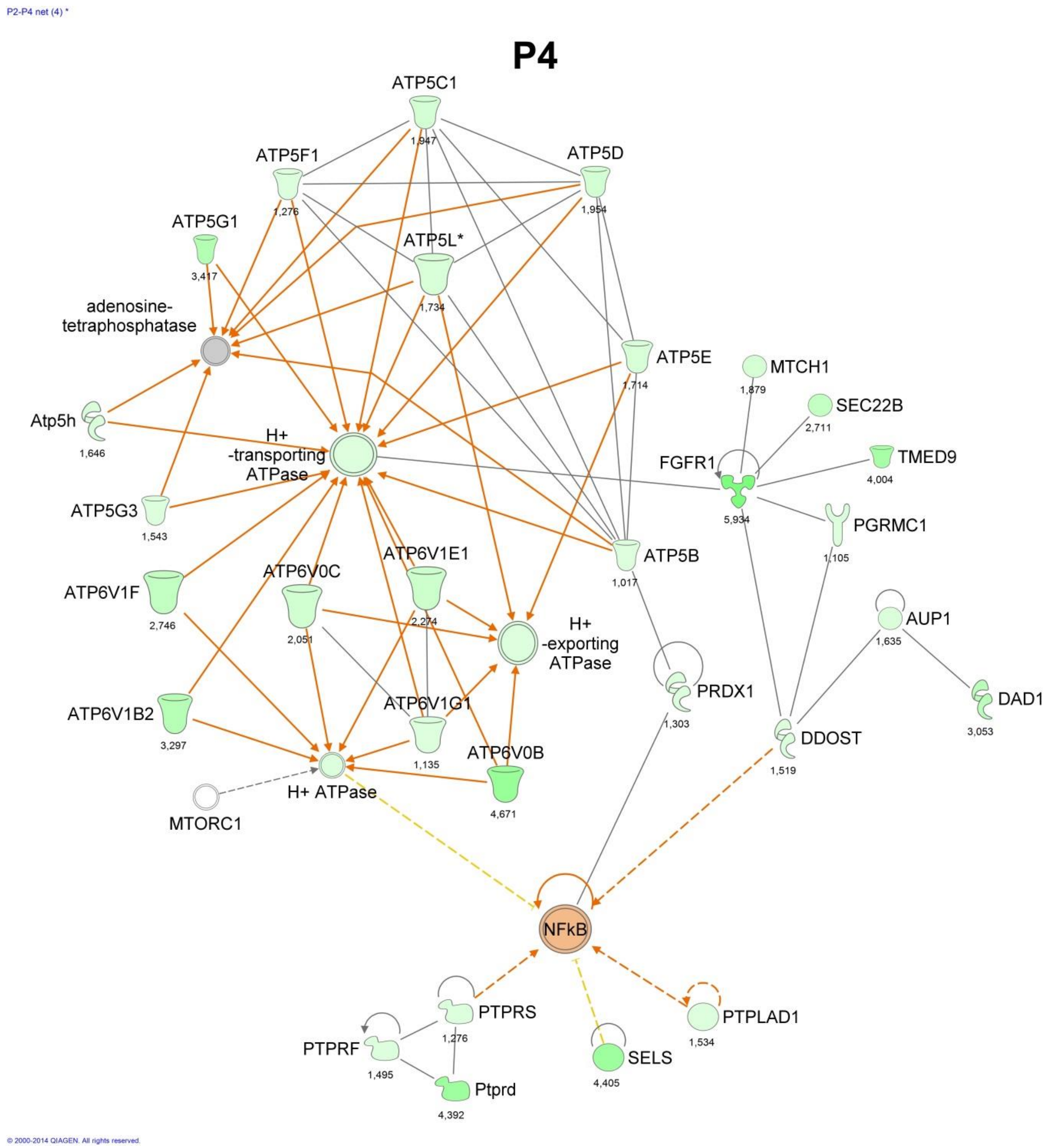

Network 3

Molecular transport, energy production, nucleic acid metabolism

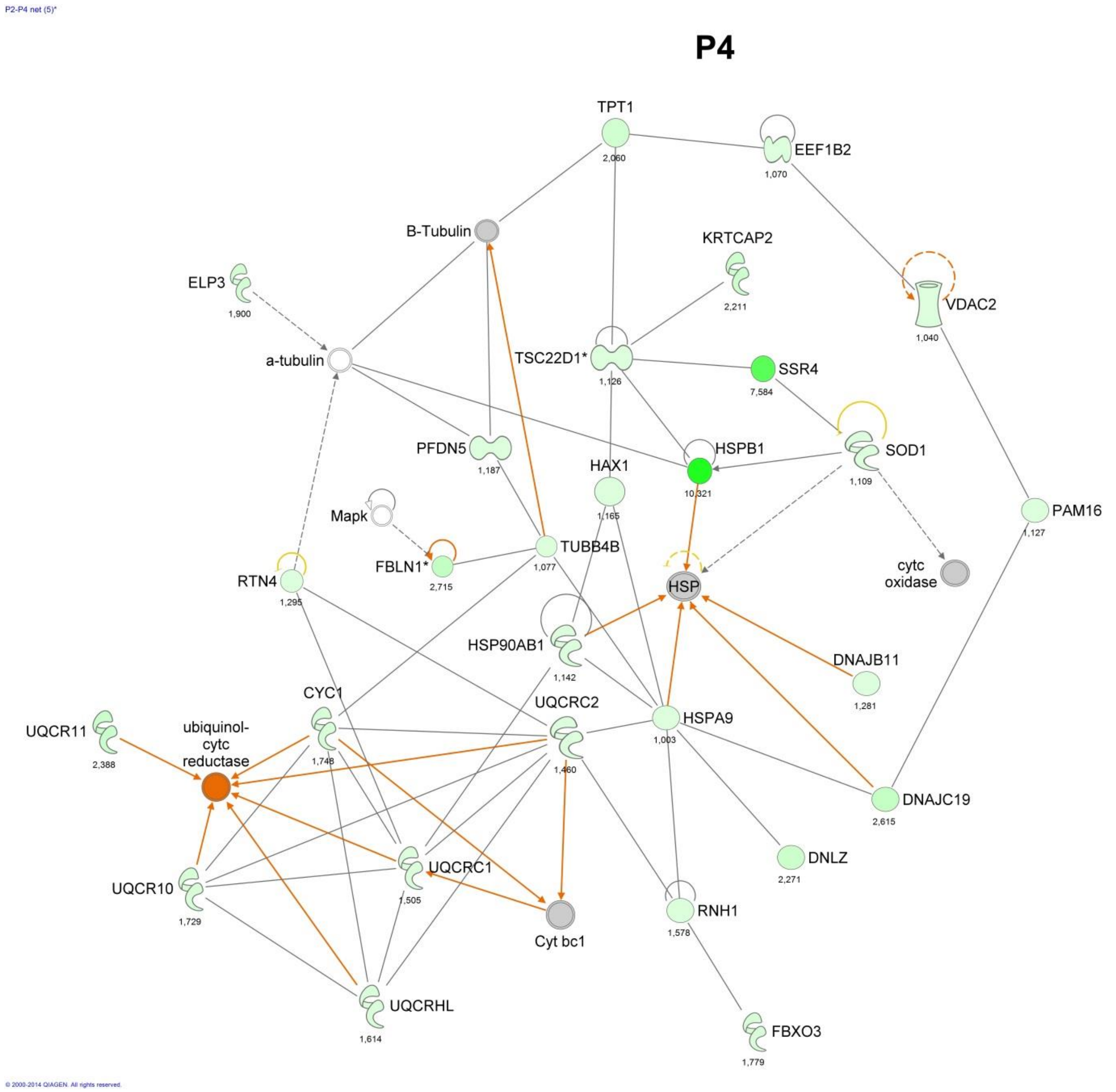

© 2000-2014 QIAGEN. All rights reserved.

Network 4

Molecular transport, nucleic acid metabolism, small molecule biochemistry

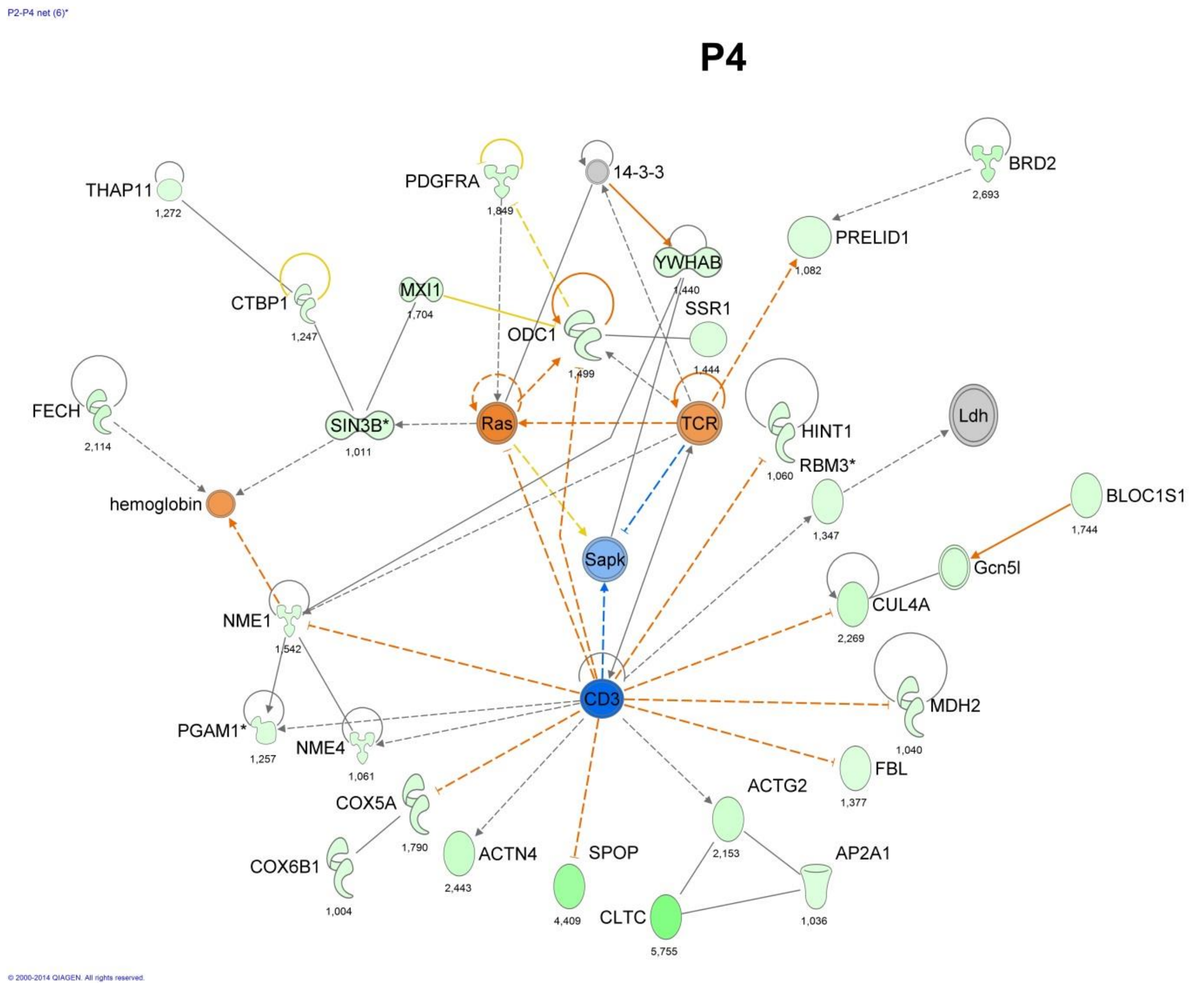

© 2000-2014 QIAGEN. All rights reserved.

Network 5

Nucleic acid metabolism, small molecule biochemistry

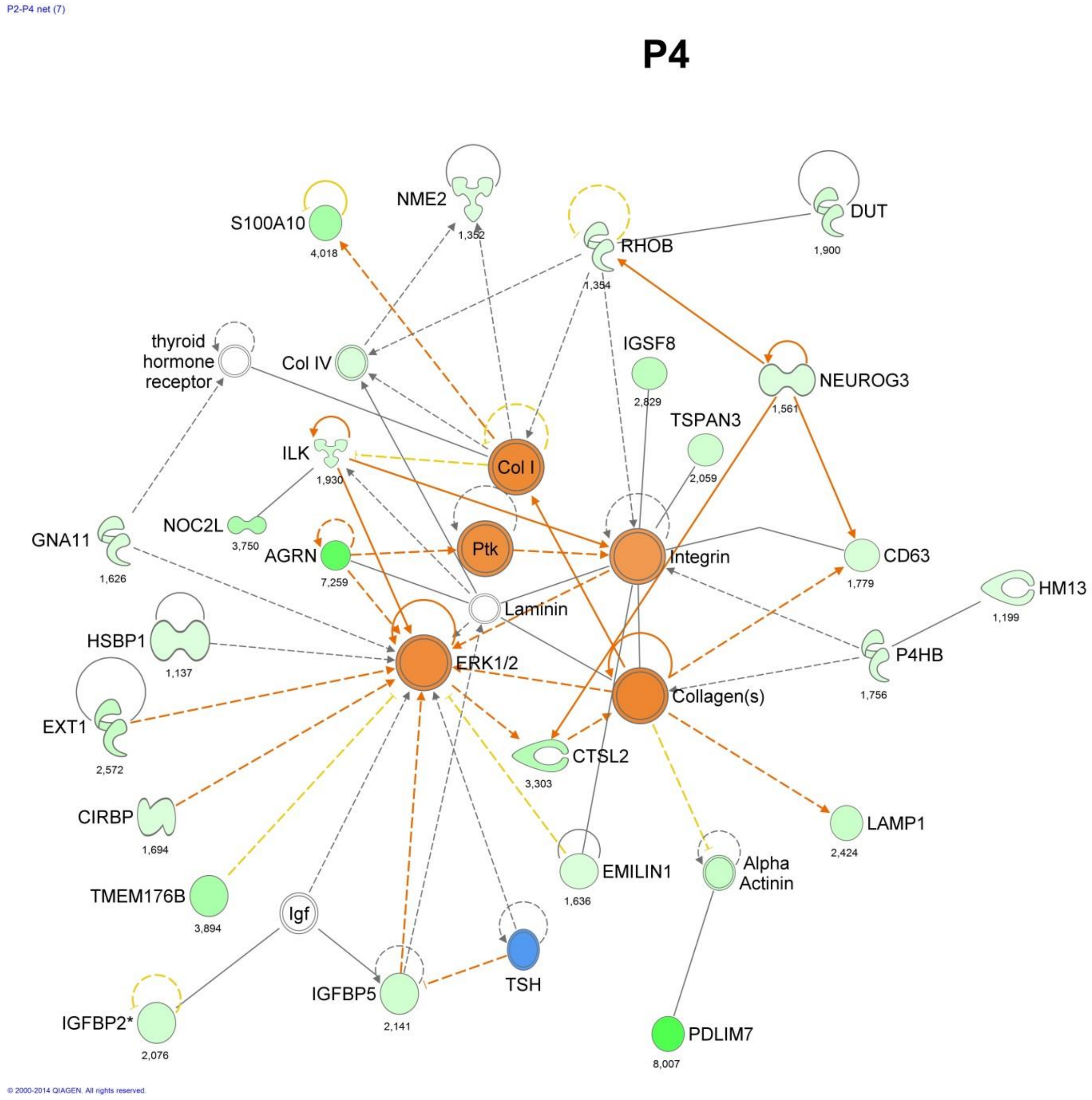

© 2000-2014 QIAGEN. All rights reserved.

Network 6

Cellular movment, skeletal and muscular development

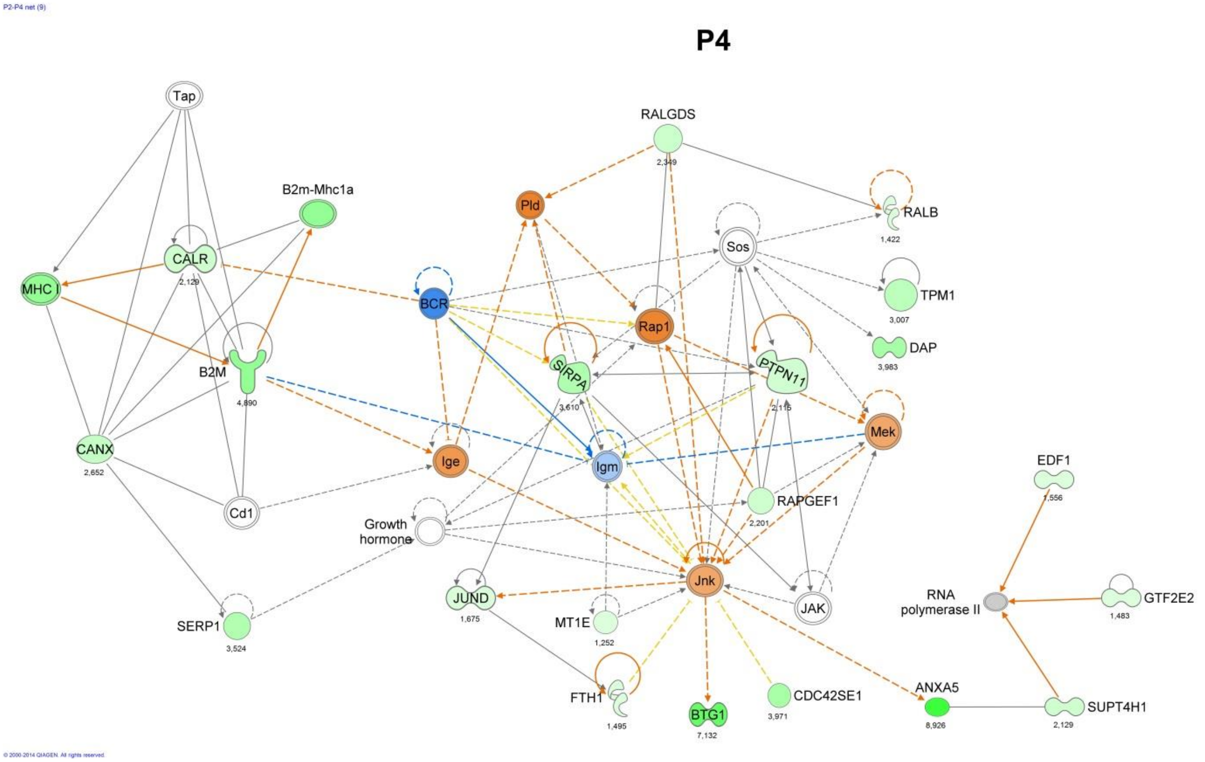

© 2000-2014 QIAGEN. All rights reserved.

Network 7

Molecular transport, cell-to-cell signaling and interaction, cellular movment

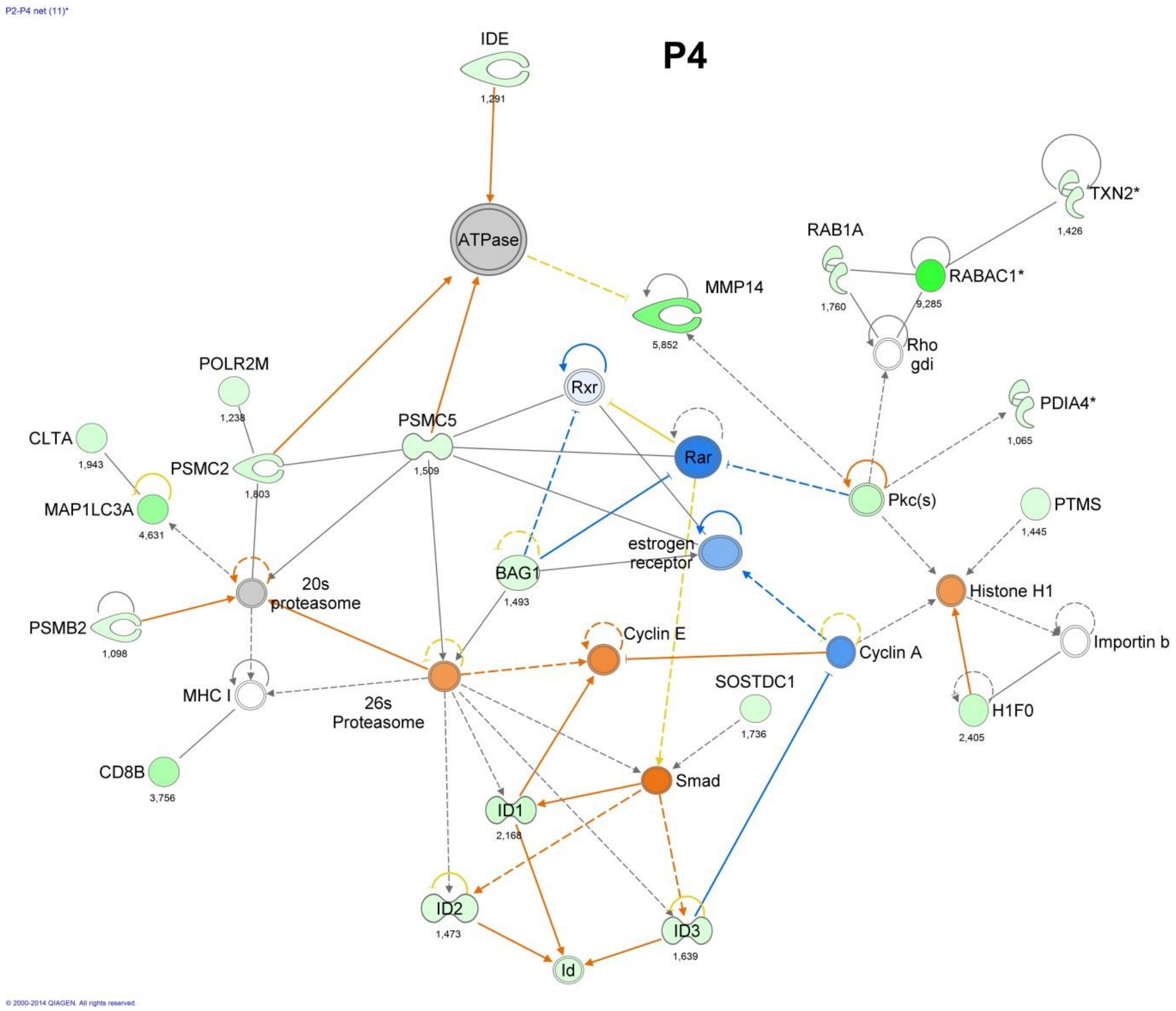

Network 8

Gene expression, cellular development, cellular growth and proliferation

P2-P4 net (12)**

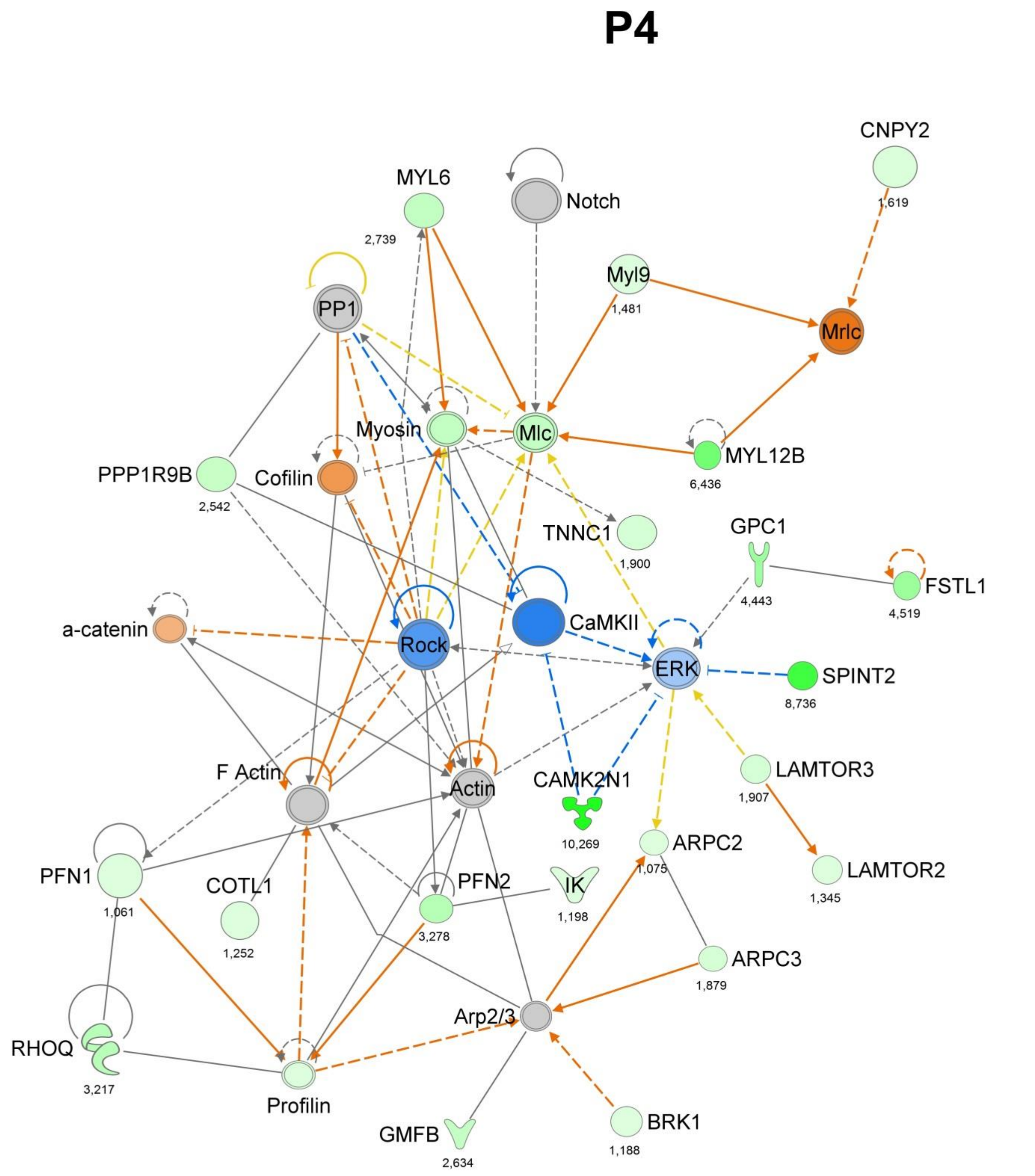

© 2000-2014 QIAGEN. All rights reserved.

Network 9

Cellular assembly and organization, cellular function and maintenance, protein synthesis

P2-P4 net (13)**

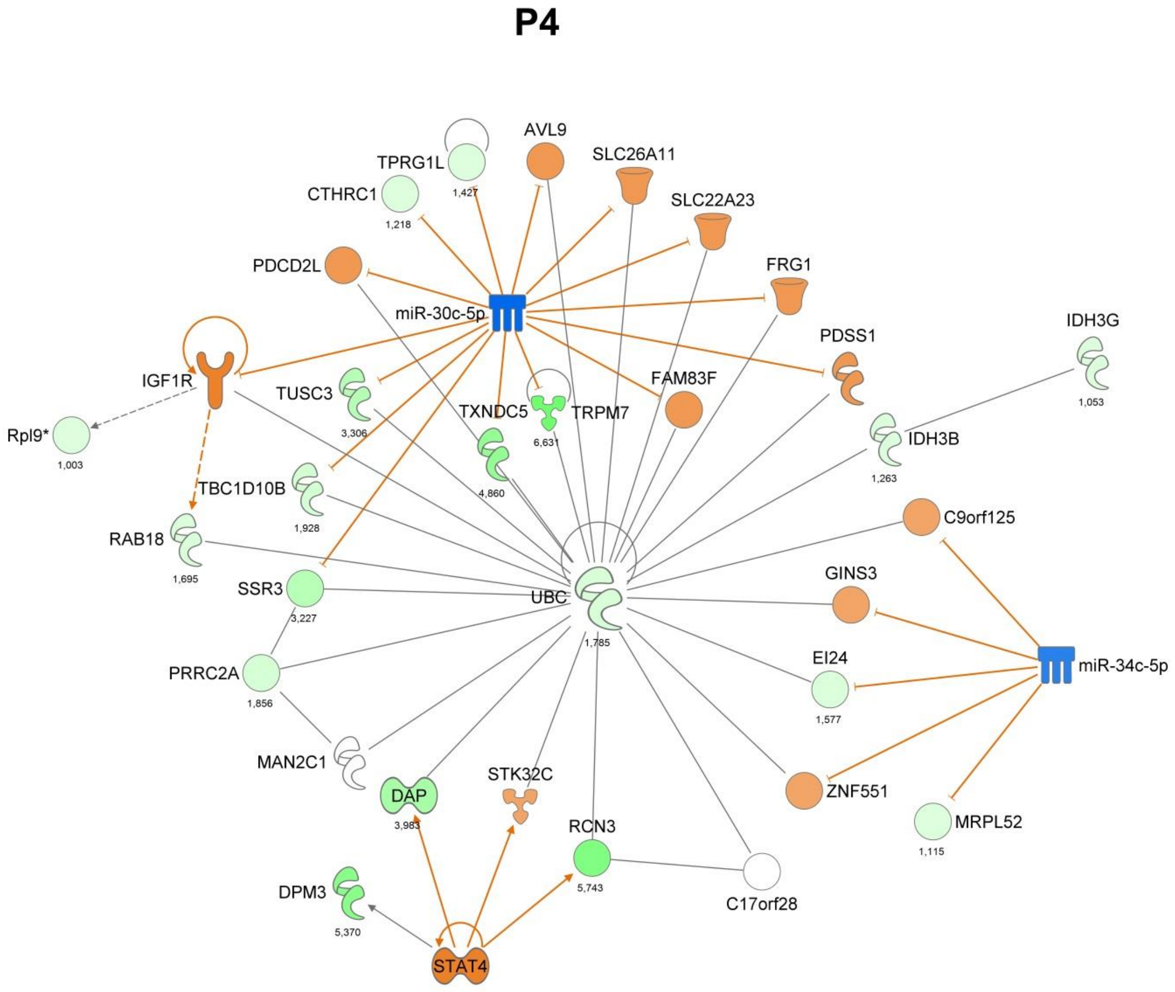

© 2000-2014 QIAGEN. All rights reserved.

Network 10

Carbohydrate metabolism, small molecule biokjemistry, cellular growth and proliferation

P2-P4 net (14)*

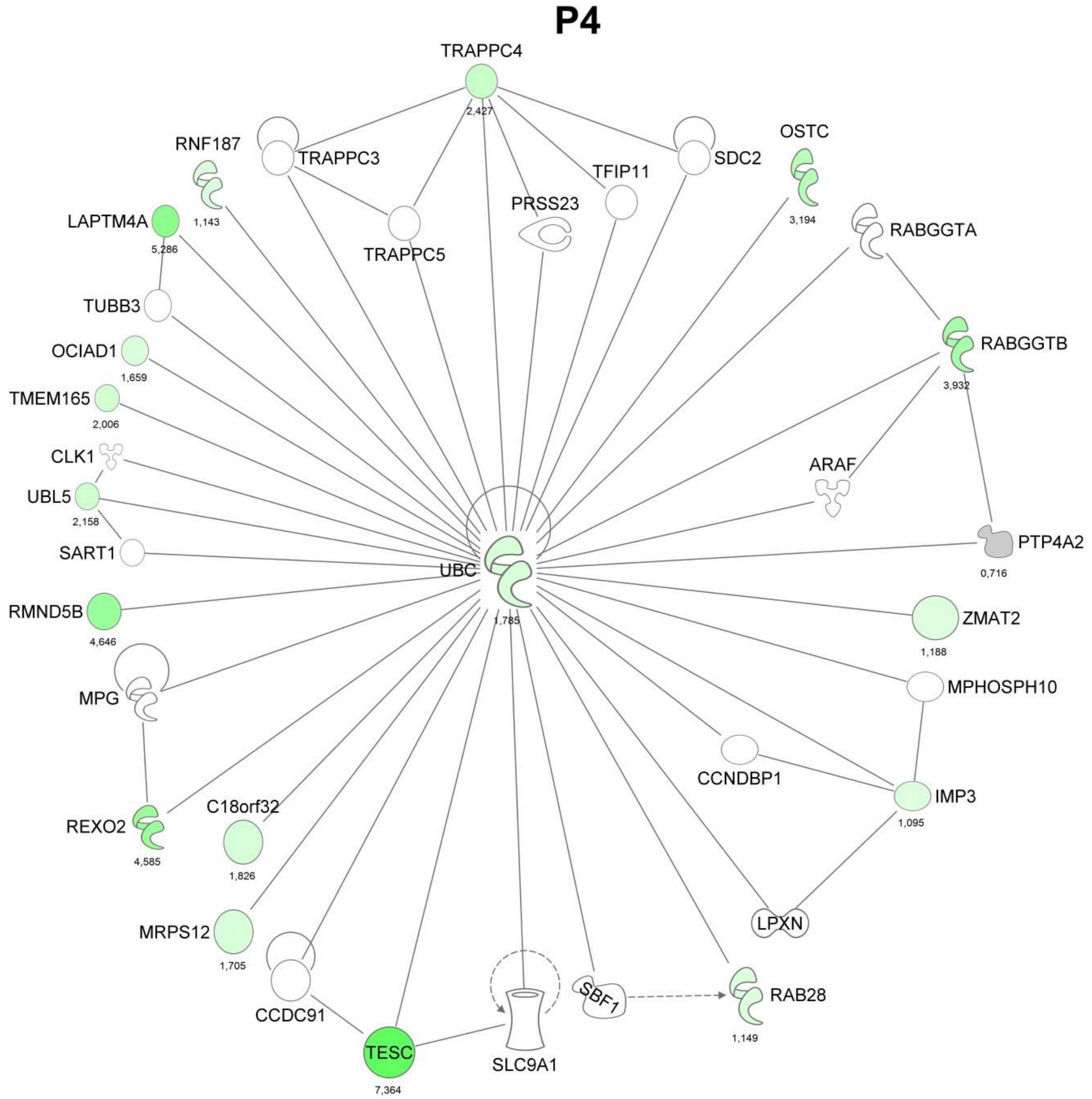

© 2000-2014 QIAGEN. All rights reserved.

Network 11

Post-transcriptional modification, molecular transport, nucleic acid metabolism

P2-P4 net(15)**

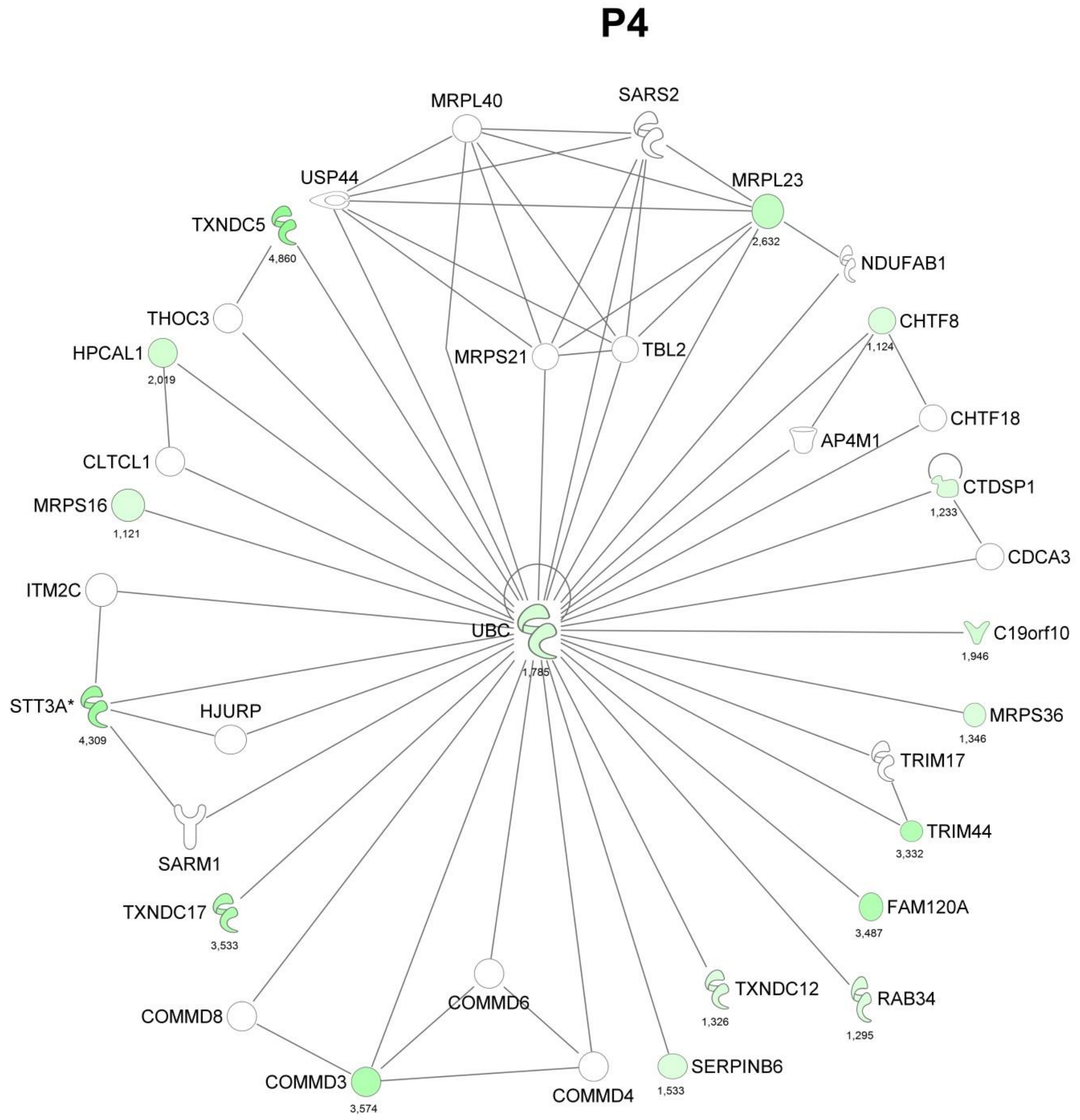

© 2000-2014 QIAGEN. All rights reserved.

Network 12

Cellular assembly, organization, function and maitenance, cellular compromise

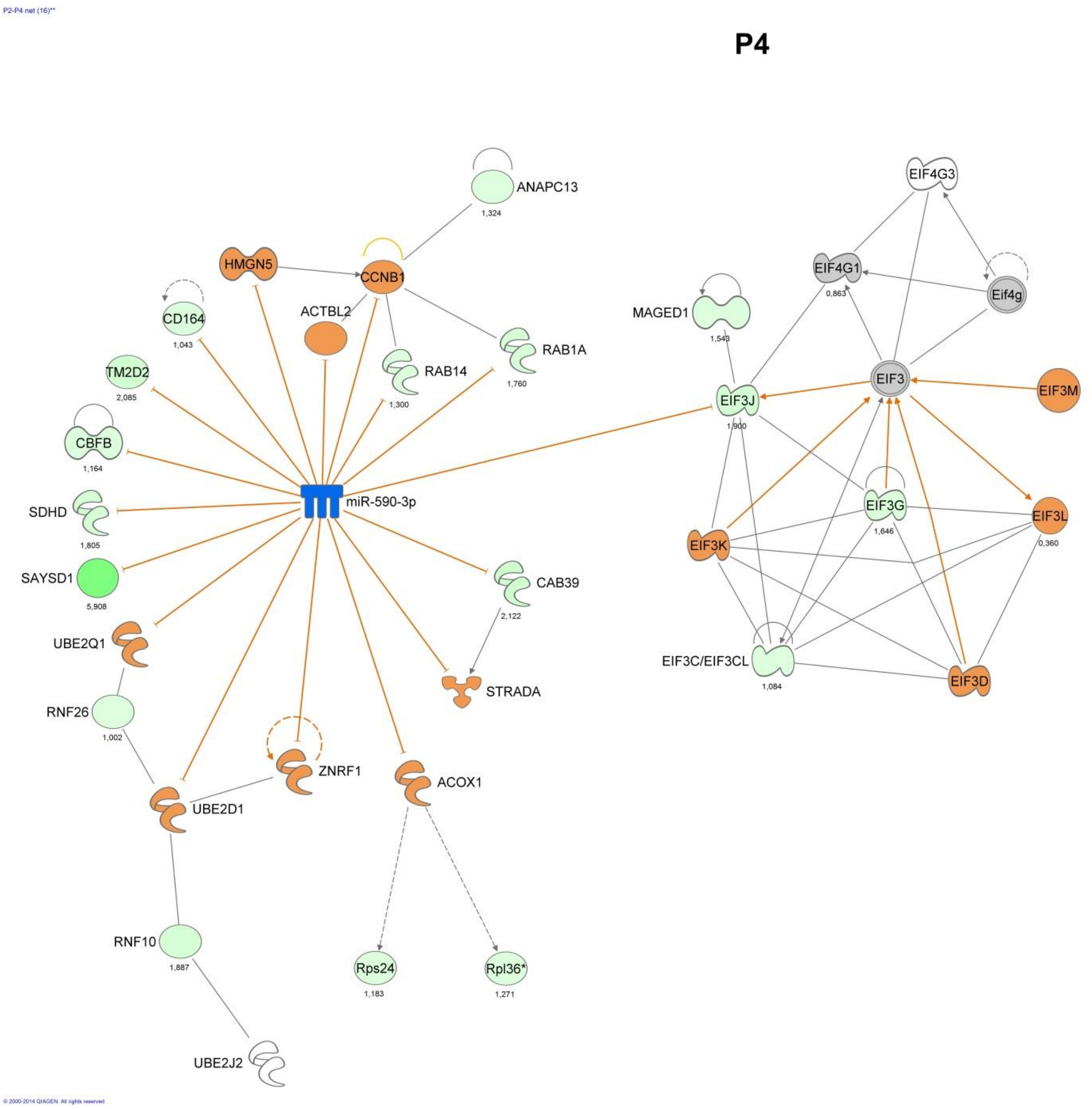

Network 13

Gene expression, protein synthesis

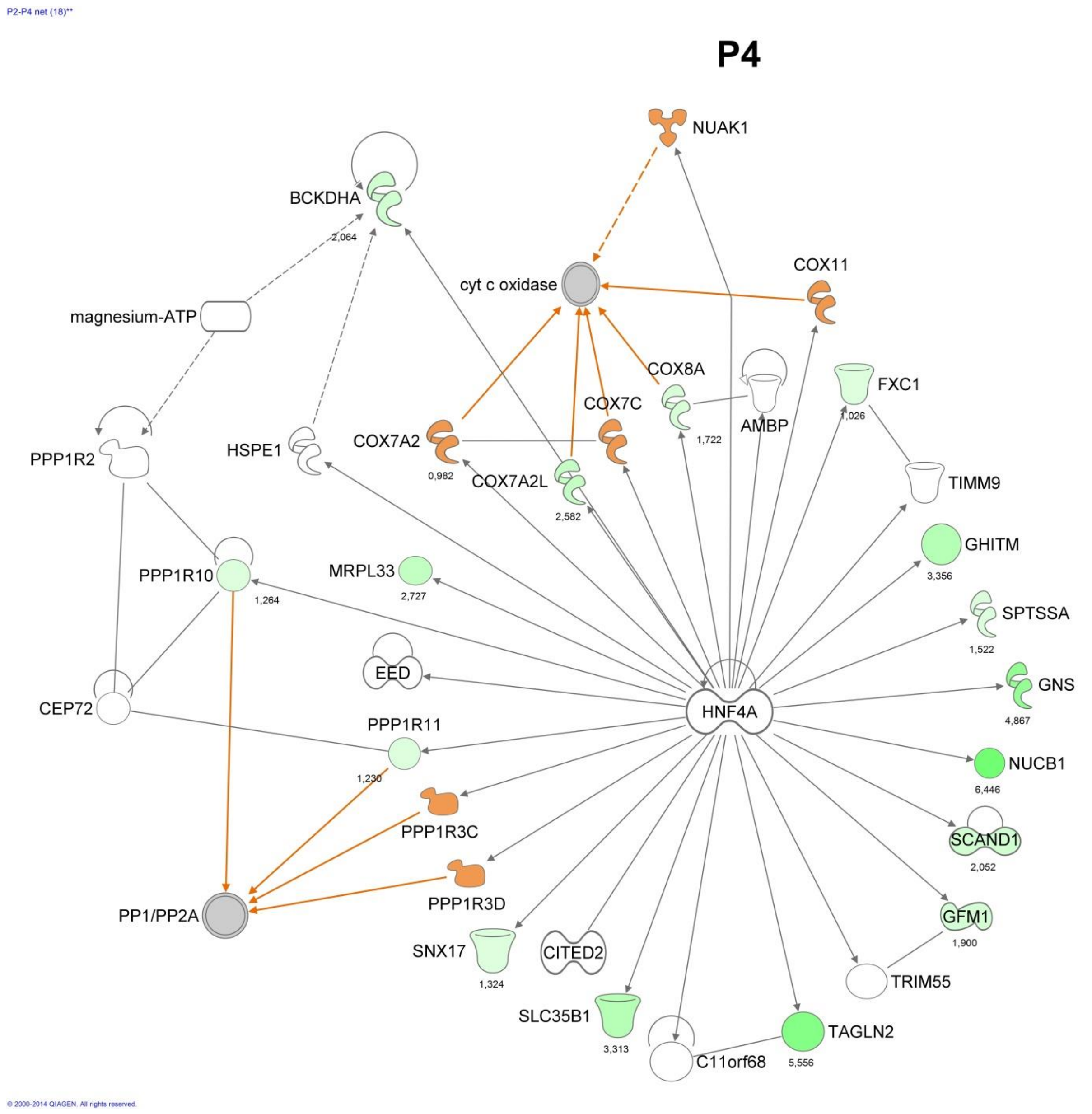

Network 14

Carbohydrate metabolism, molecular transport

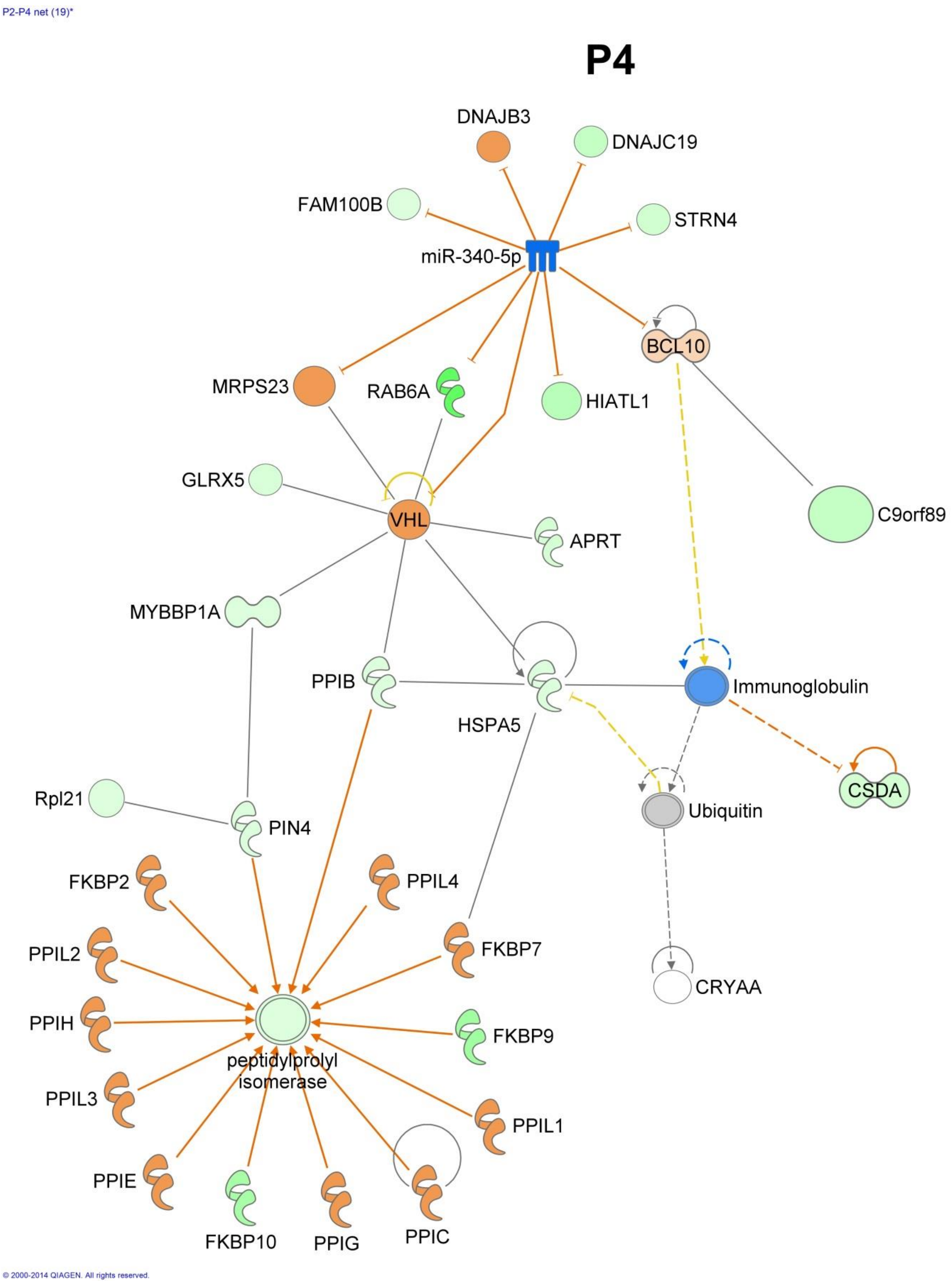

Network 15

Cell death, post-transcriptional modification, protein folding

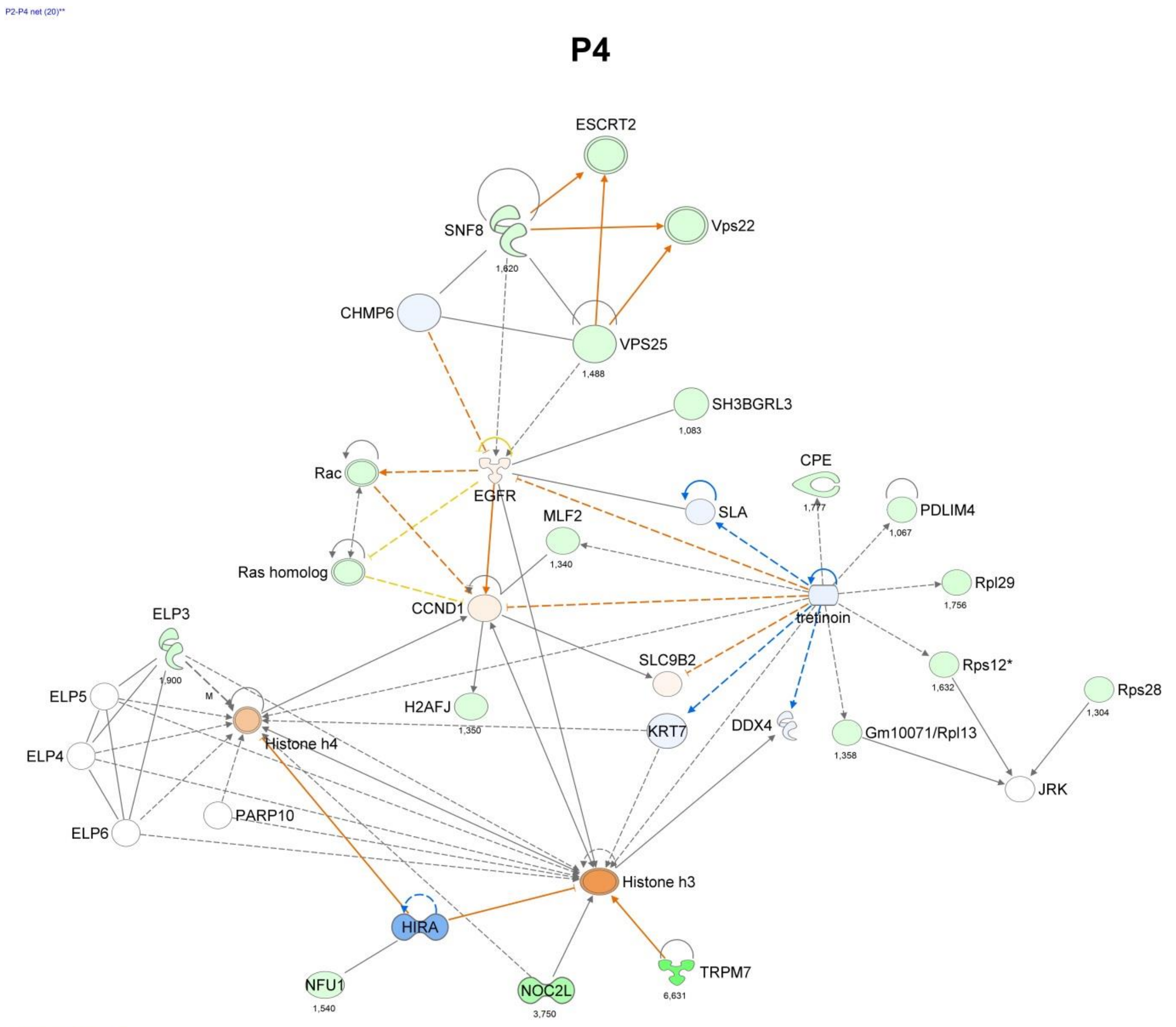

© 2000-2014 QIAGEN. All rights reserved.

Network 16

Cellular development, cellular growth and proliferation, cell cycle

P2-P4 net (21)

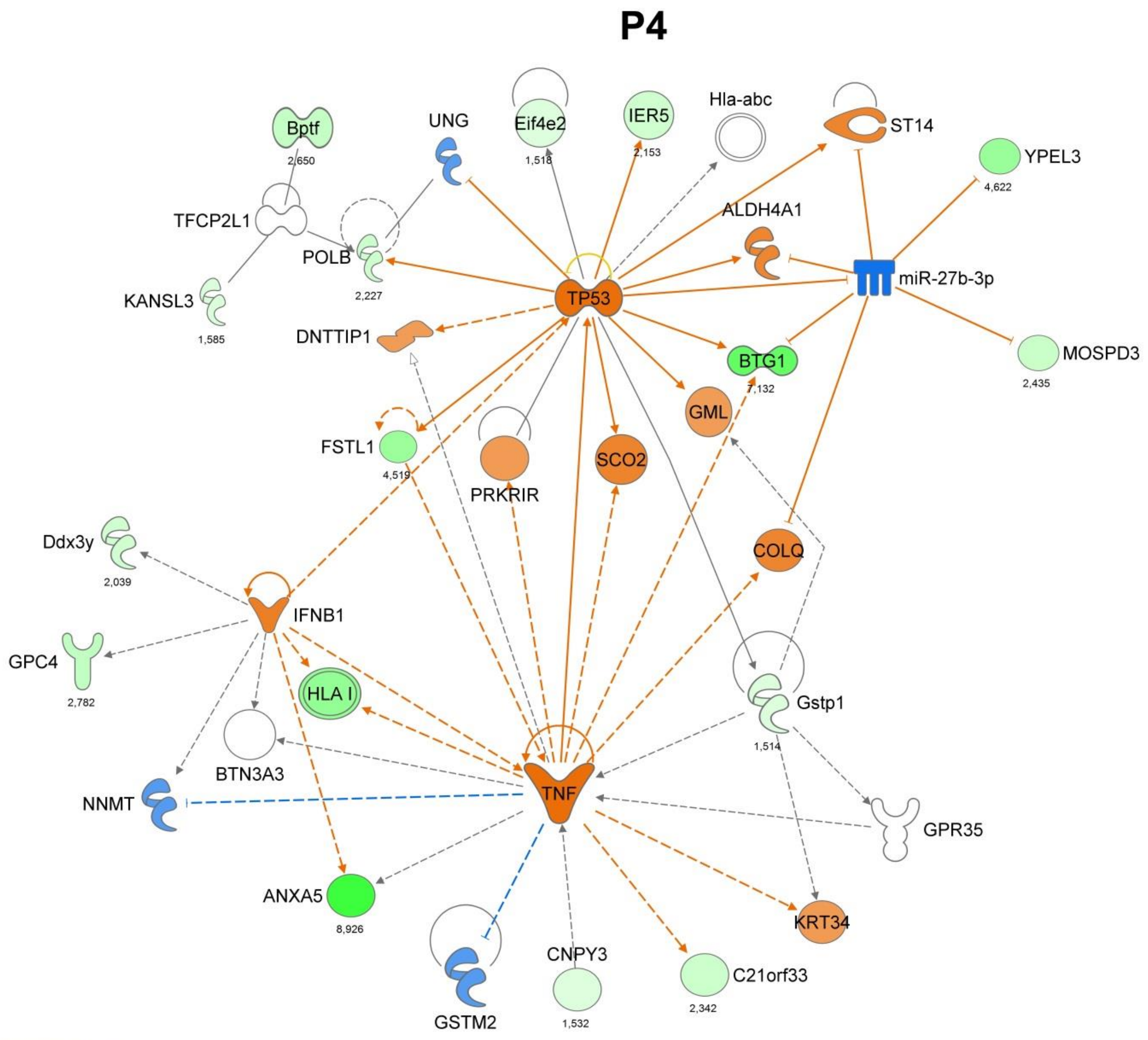

© 2000-2014 QIAGEN. All rights reserved.

Network 17

Lipid metabolism, small molecule biochemistry, cell death

P2-P4 net (22)*

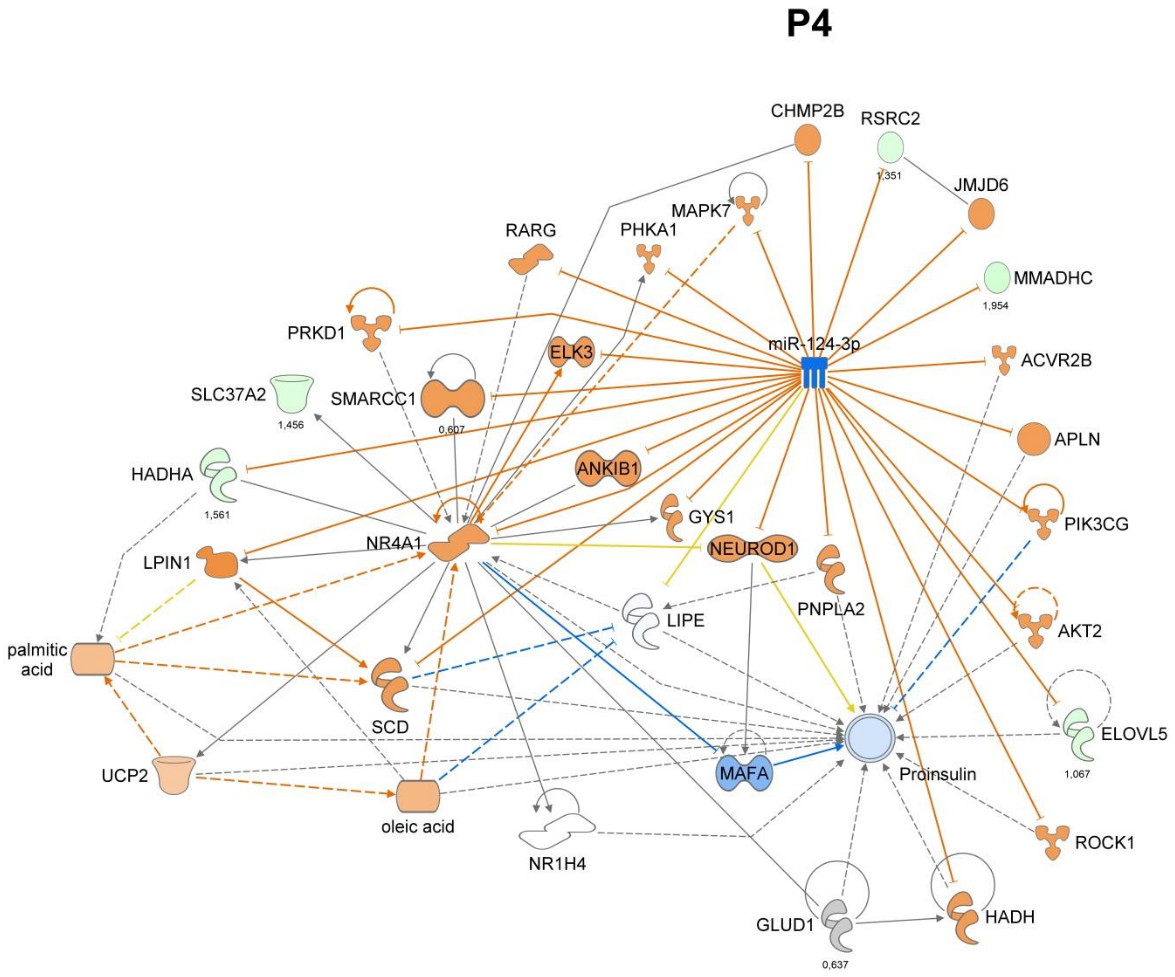

© 2000-2014 QIAGEN. All rights reserved.

Network 18

Energy production, lipid metabolism, small molecule biochemistry

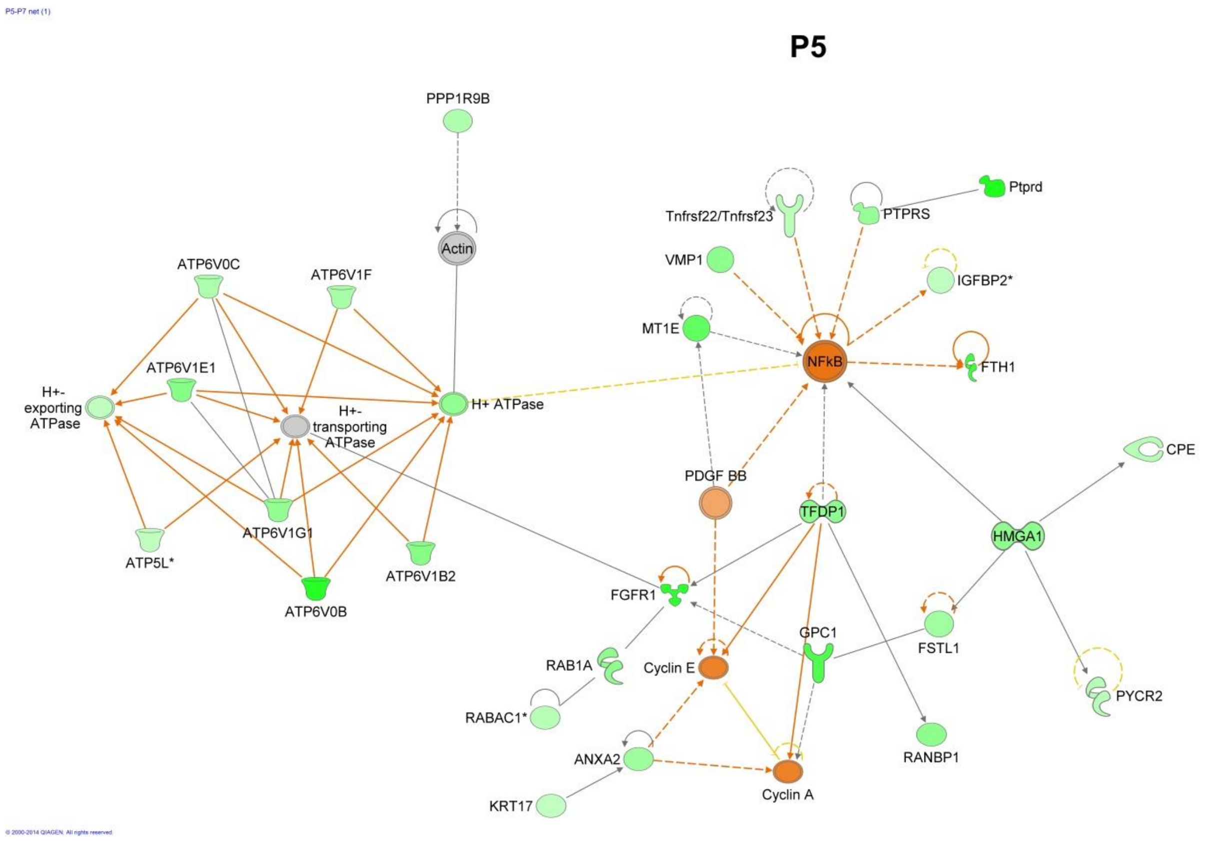

Network 1

Molecular transport, cellular compromise

P5-P7 net (7)

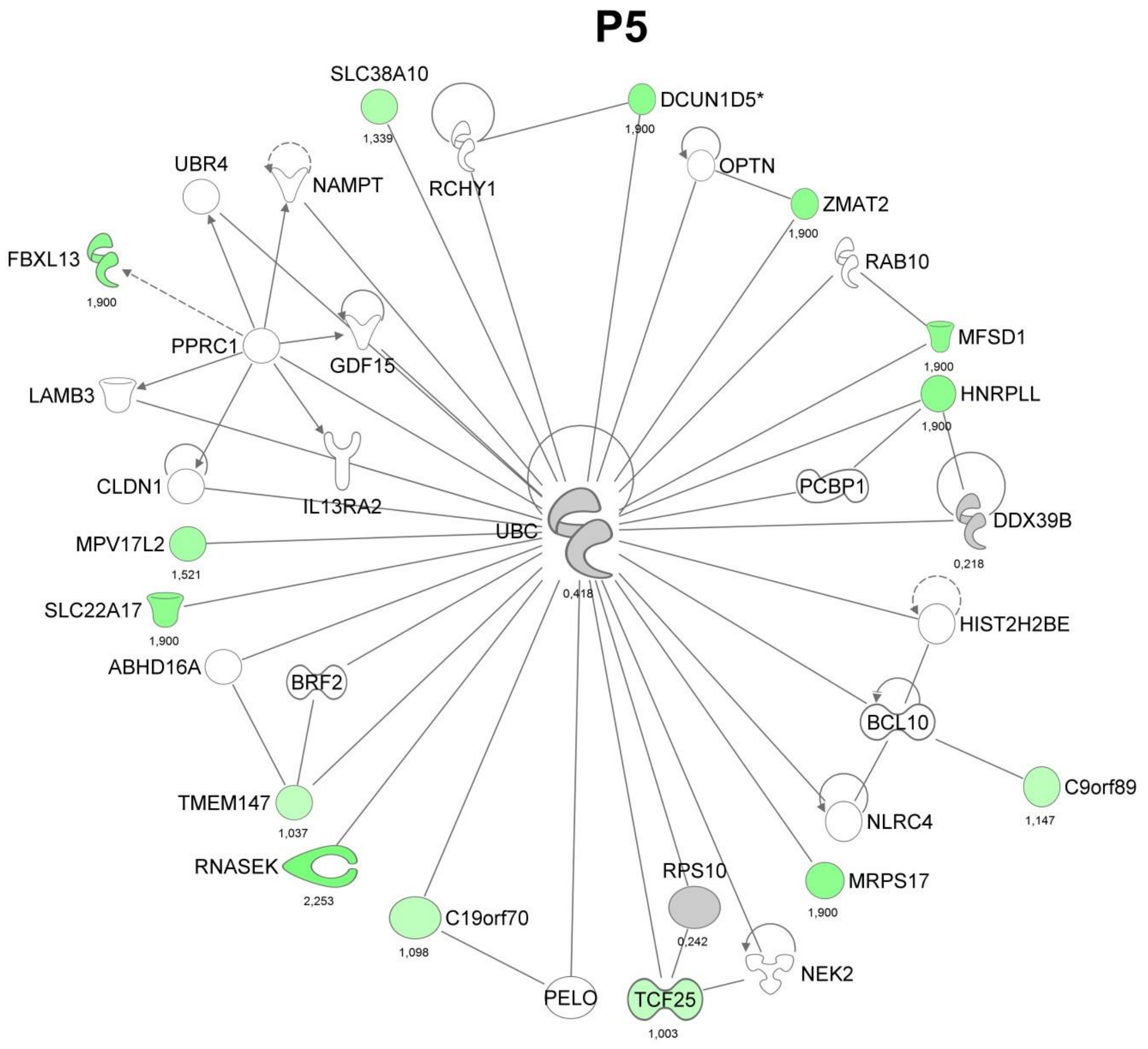

© 2000-2014 QIAGEN. All rights reserved.

Network 2

Cellular movment, cell morphology

P5-P7 net (8)

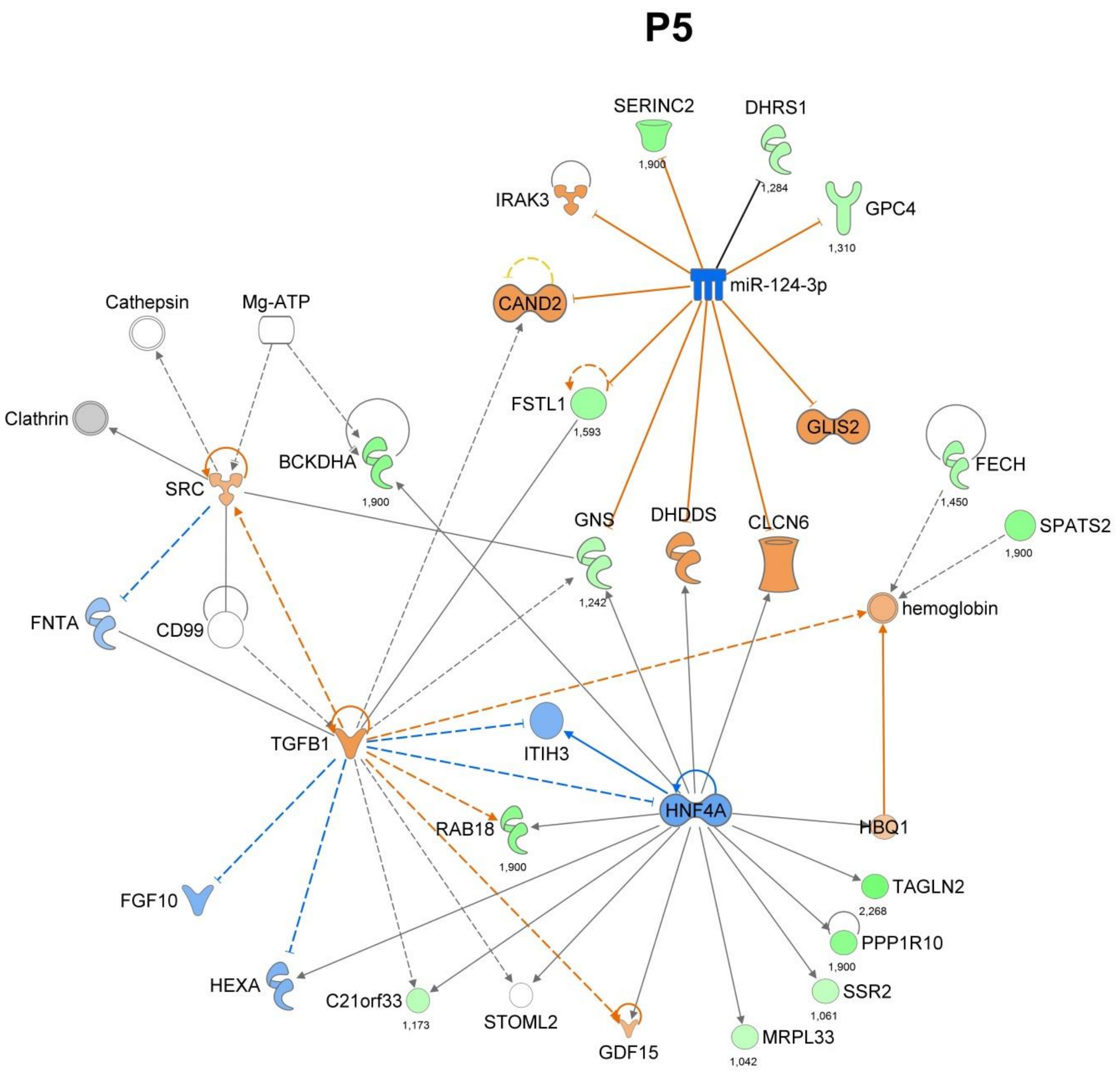

© 2000-2014 QIAGEN. All rights reserved.

Network 3

Carbohydrate metabolism, small molecule biochemistry, cell-to-cell signaling and interaction

P5-P7 net (9)

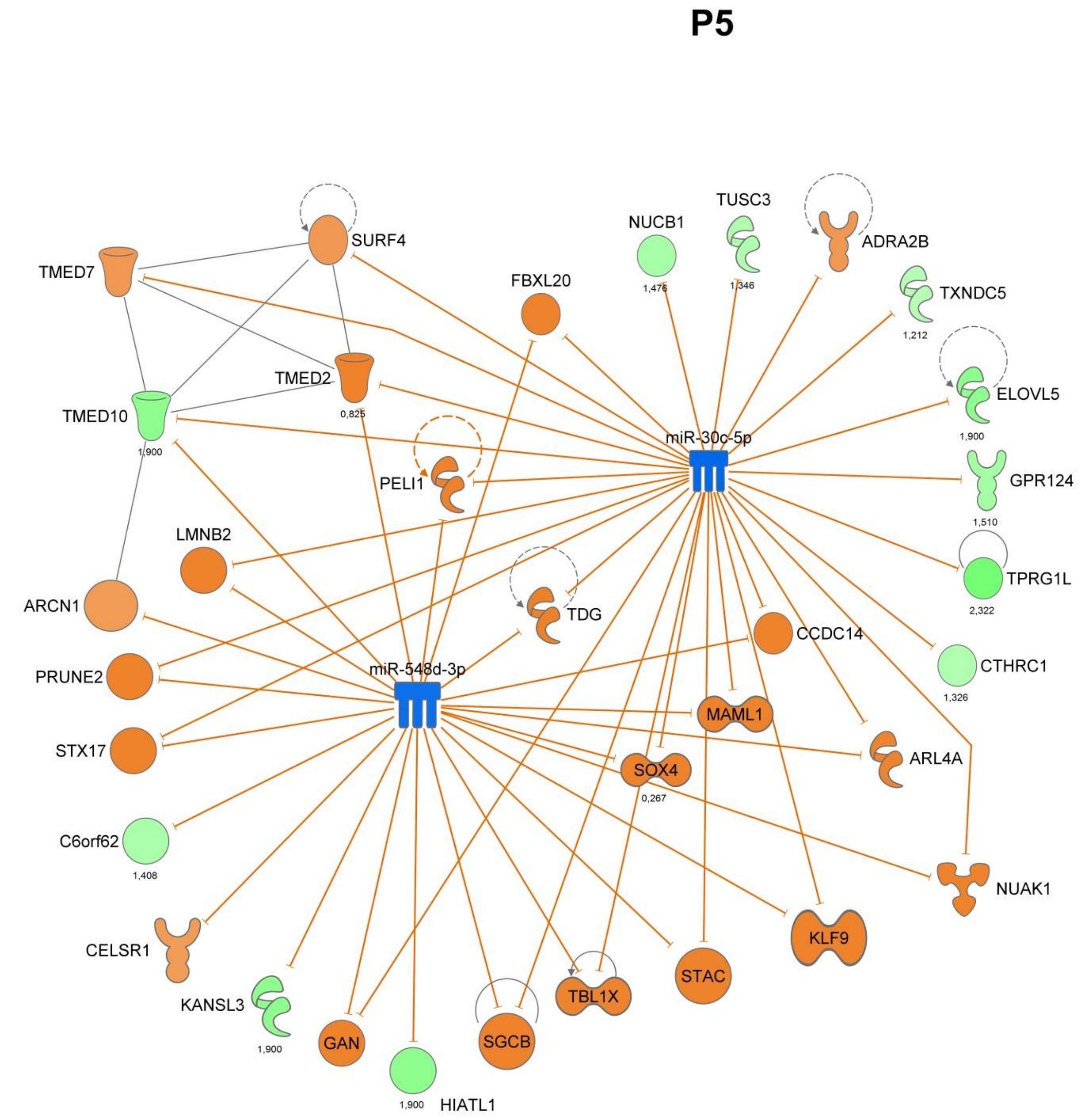

© 2000-2014 QIAGEN. All rights reserved.

Network 4

Cellular assembly and organization, cellular function and maintenance, cell death

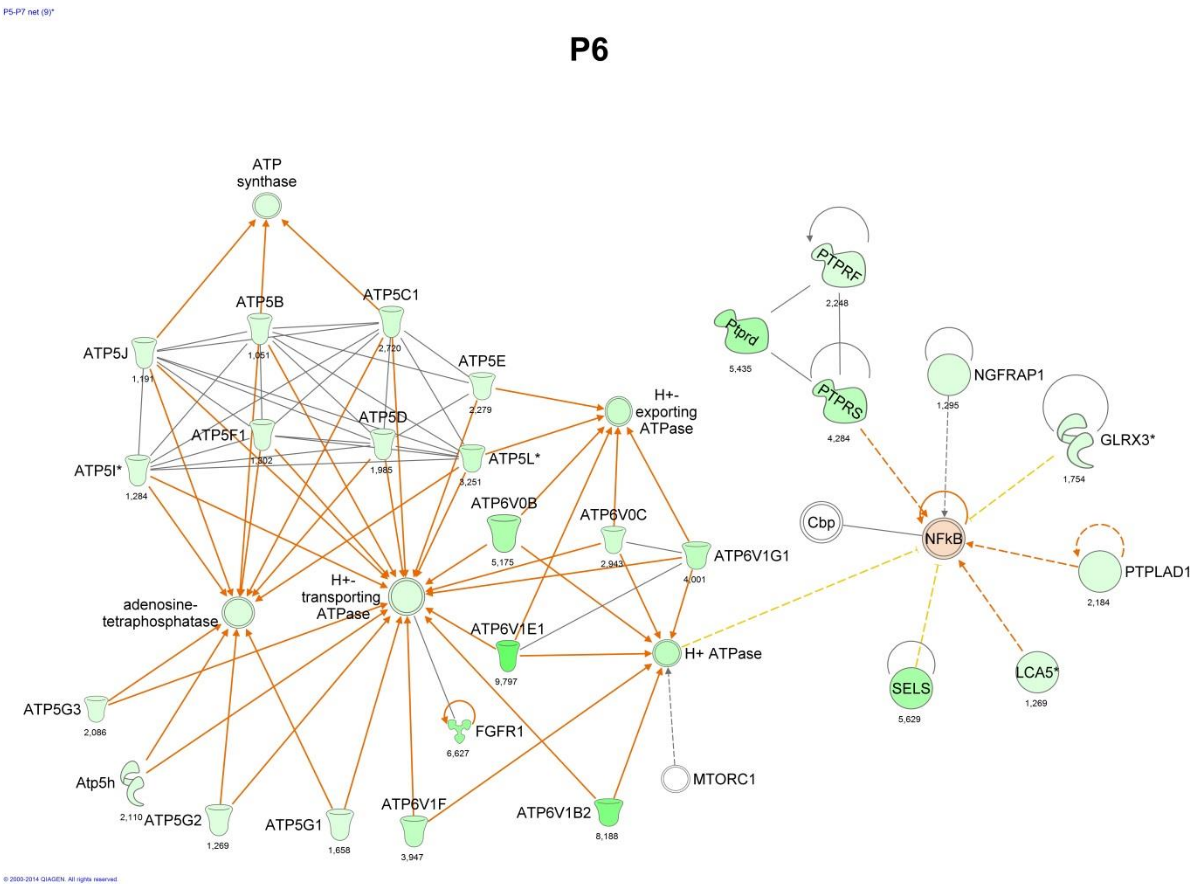

Network 1

Energy production, nucleic acid metabolism, small molecule biochemistry

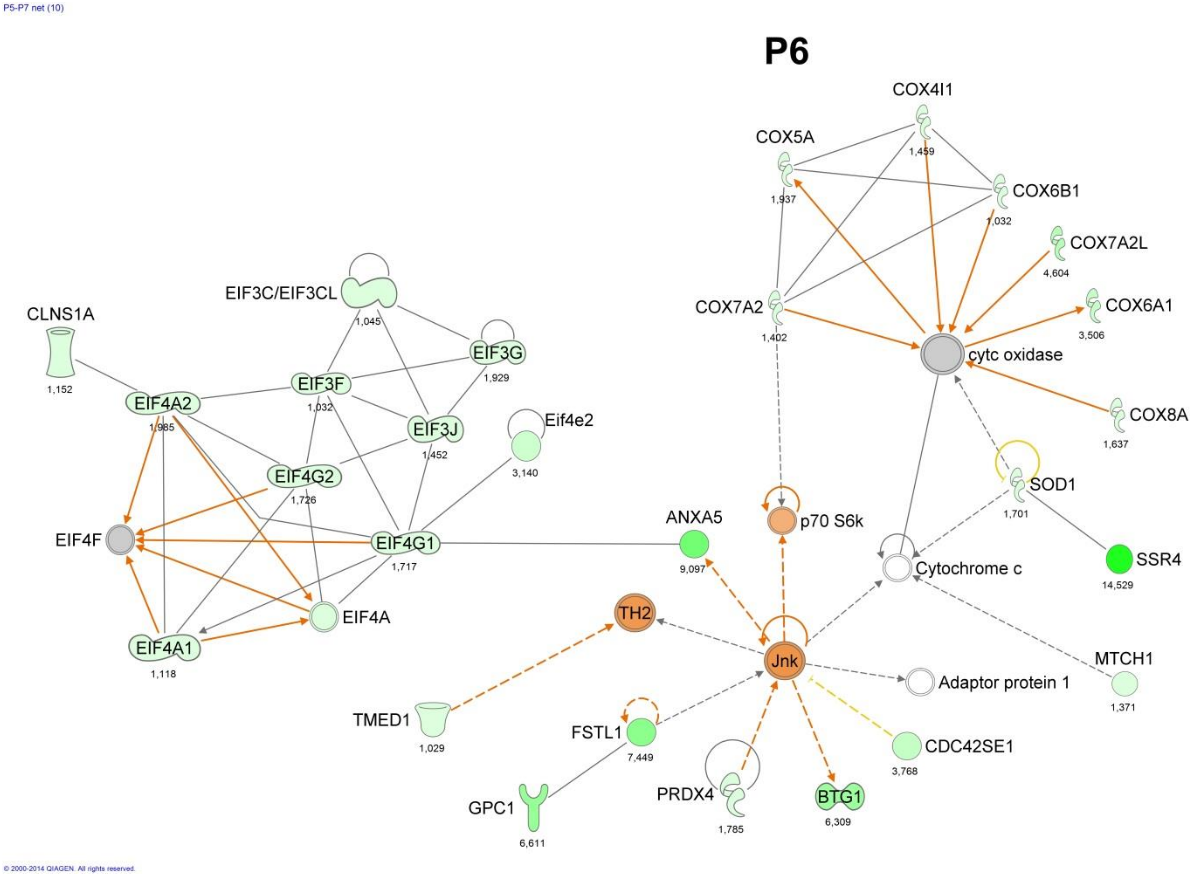

Network 2

Gene expression, protein synthesis, RNA post-transcriptional modification

P5-P7 net (11)

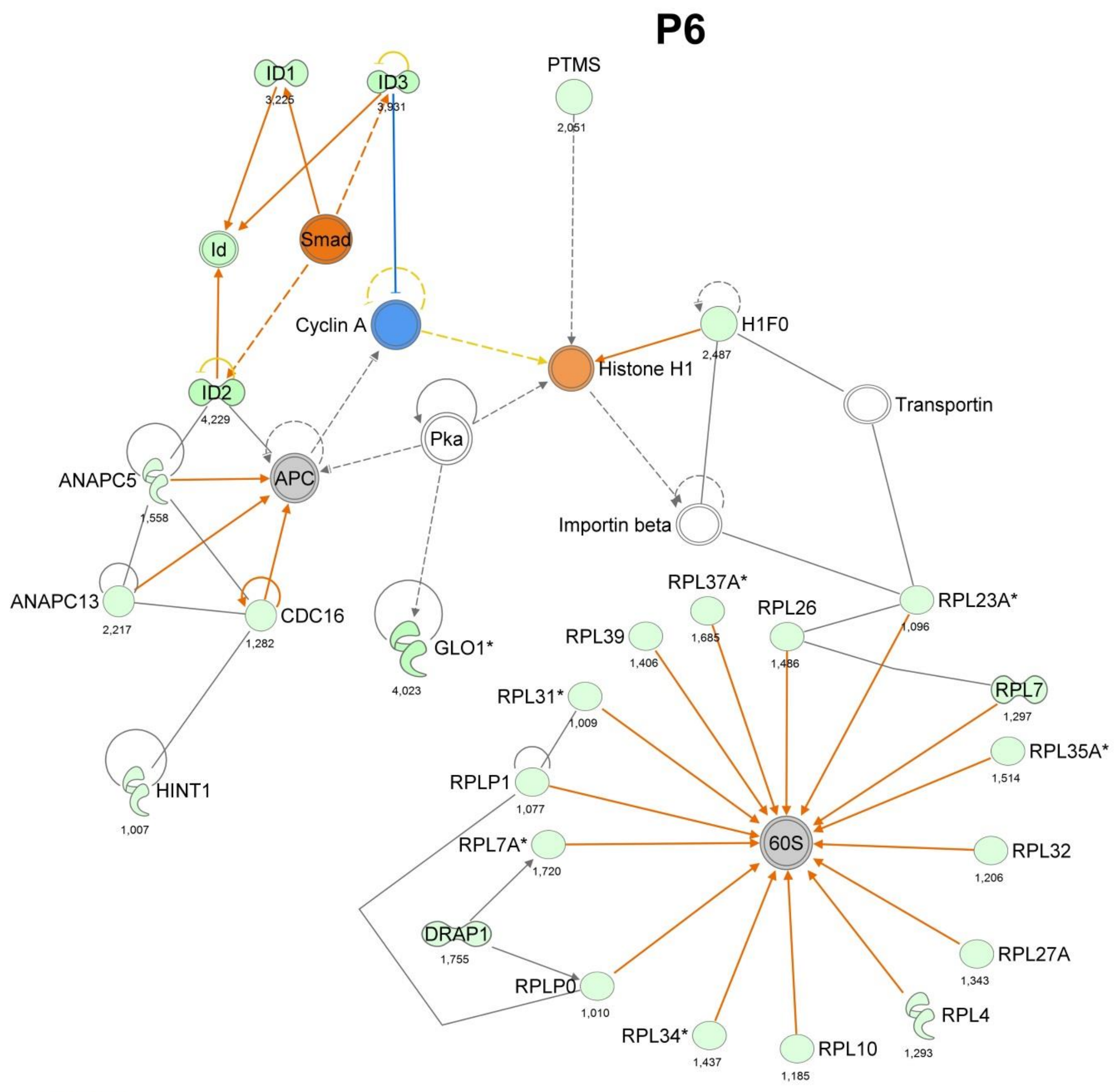

© 2000-2014 QIAGEN. All rights reserved.

Network 3

Gene expression, cellular development, cellular growth and proliferation

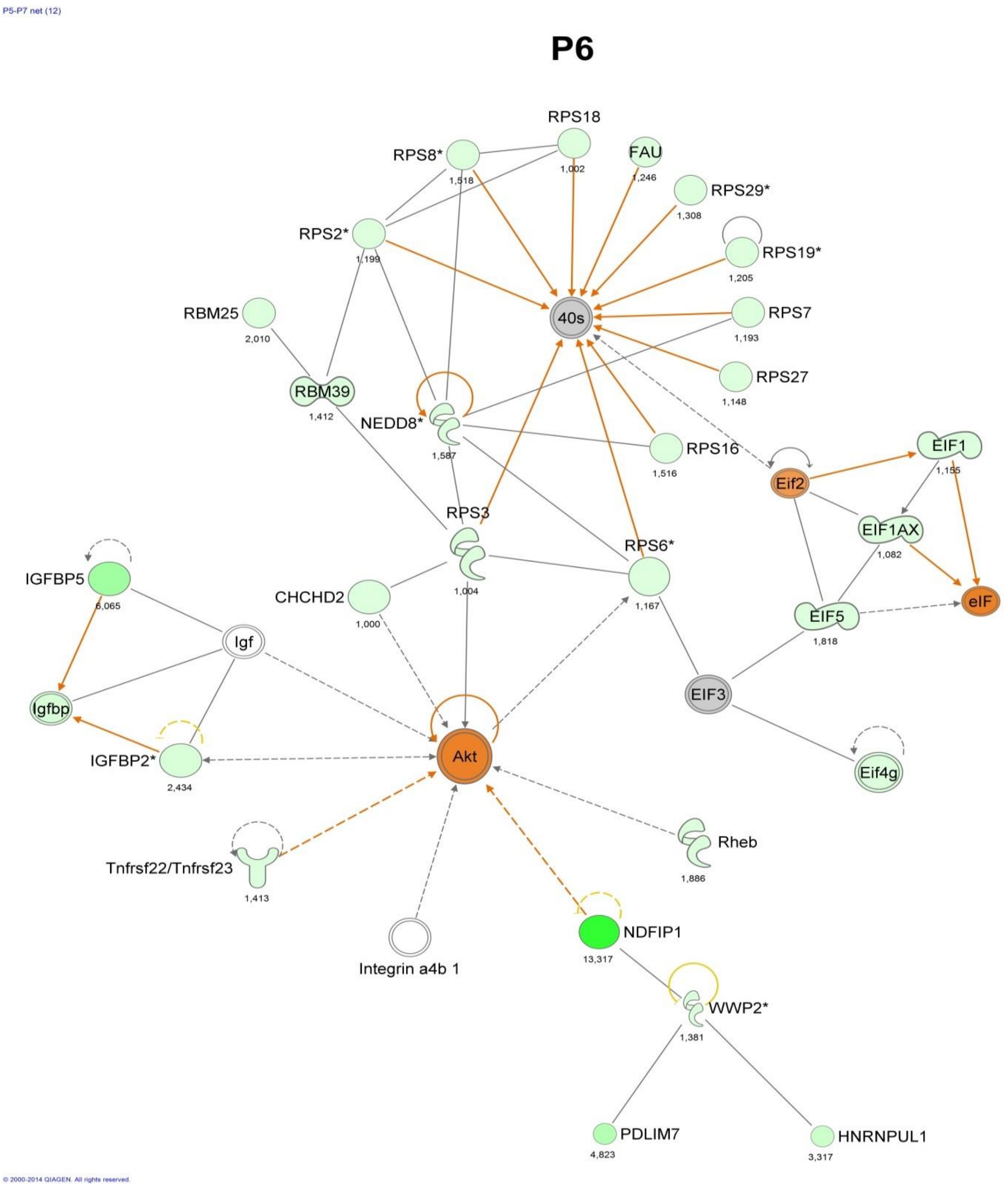

Network 4

Protein synthesis, RNA post-transcriptional modification, gene expression

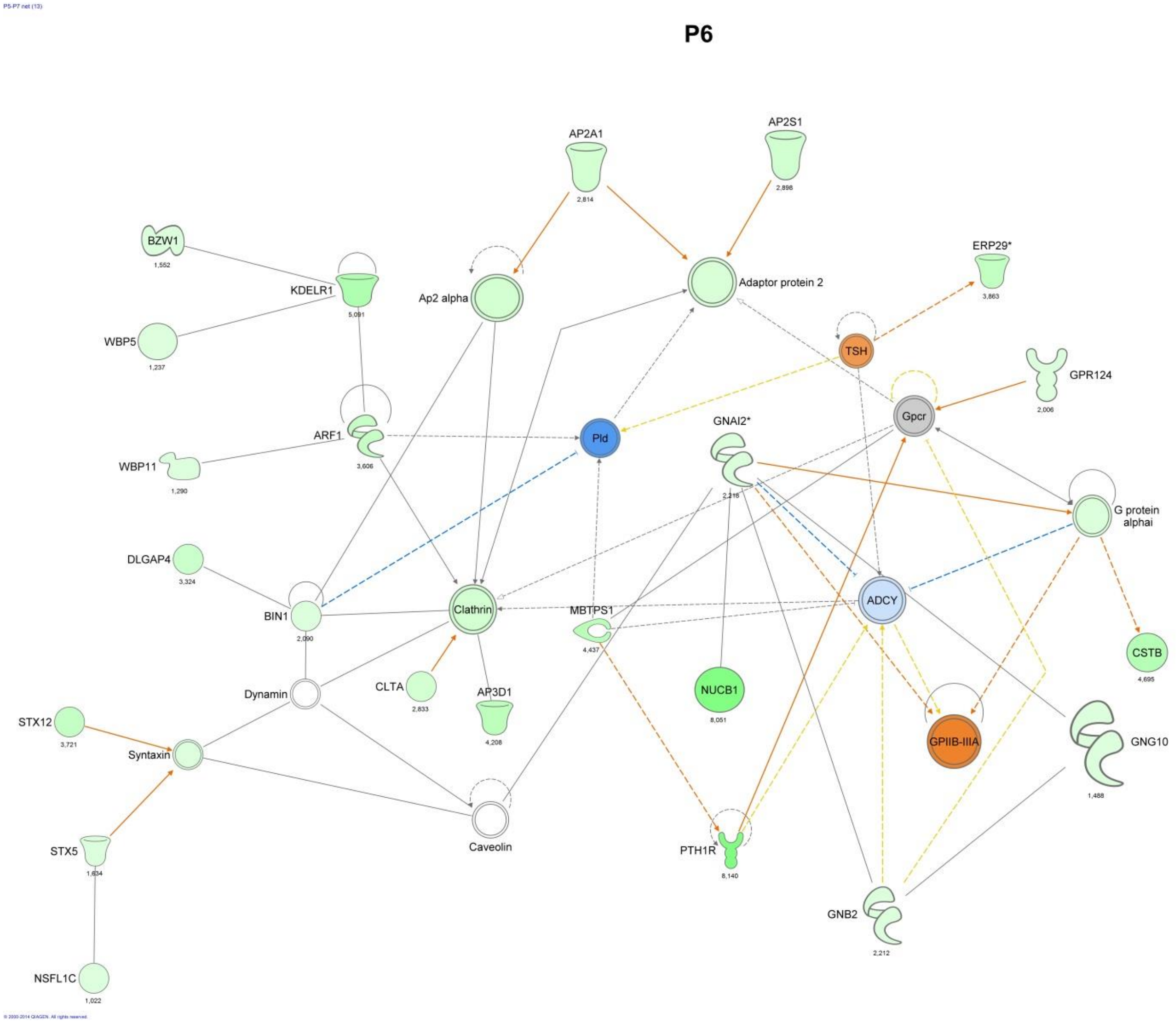

Network 5

Molecular transport, protein trafficking, connective tissue development and function

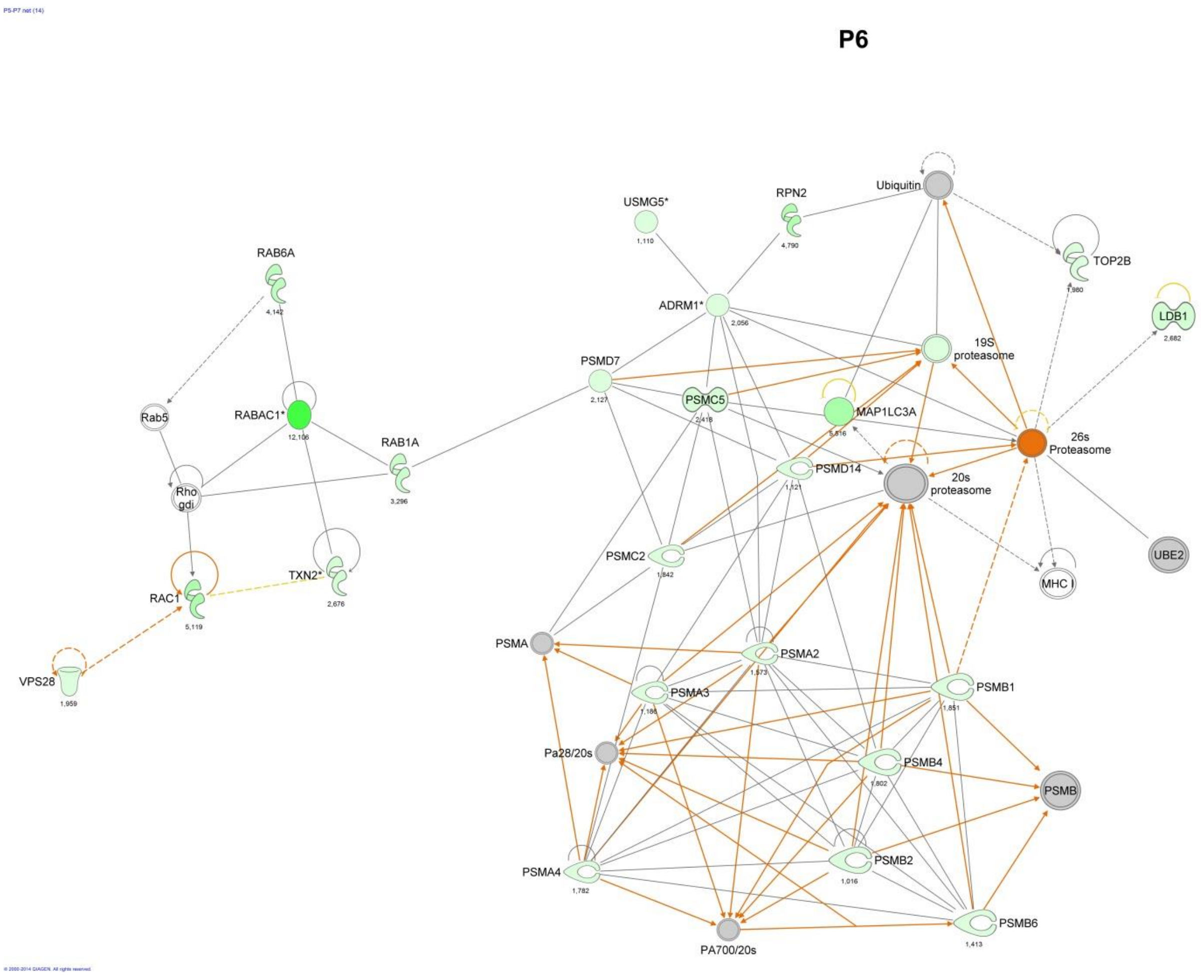

Network 6

Cell death, tissue morphology, embryonic development

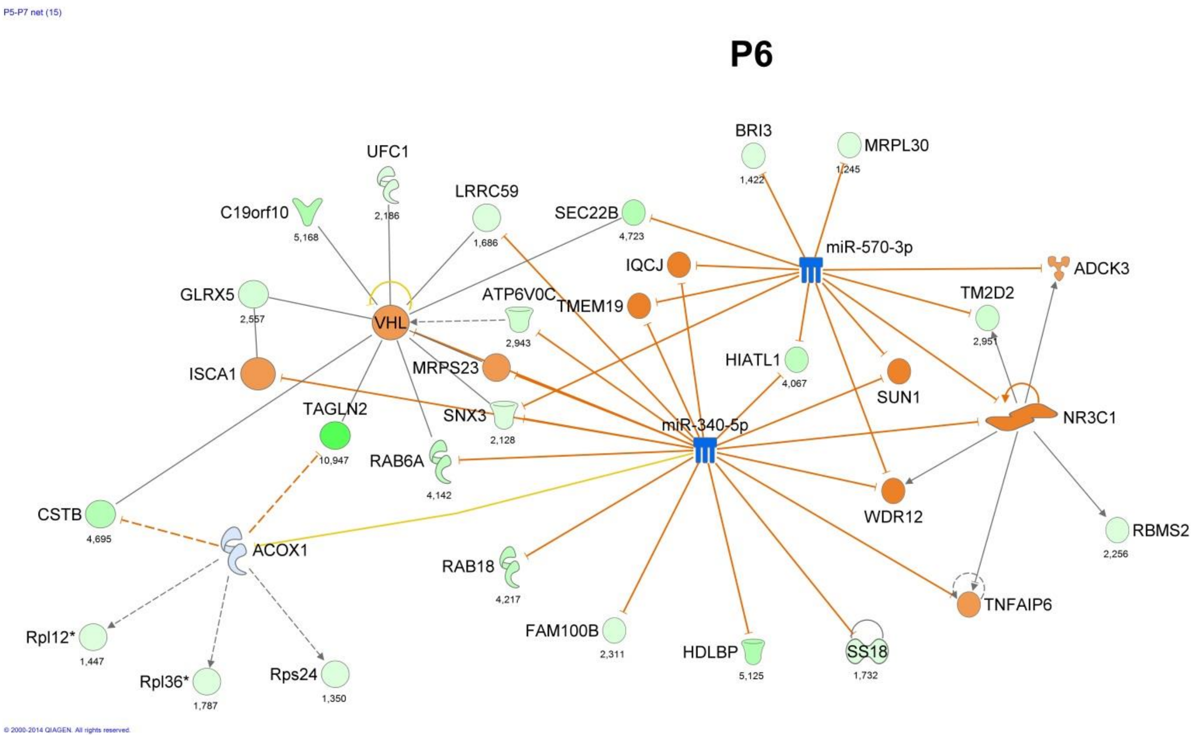

© 2000-2014 QIAGEN. All rights reserved.

Network 7

Carbohydrate metabolism, small molecule biochemistry

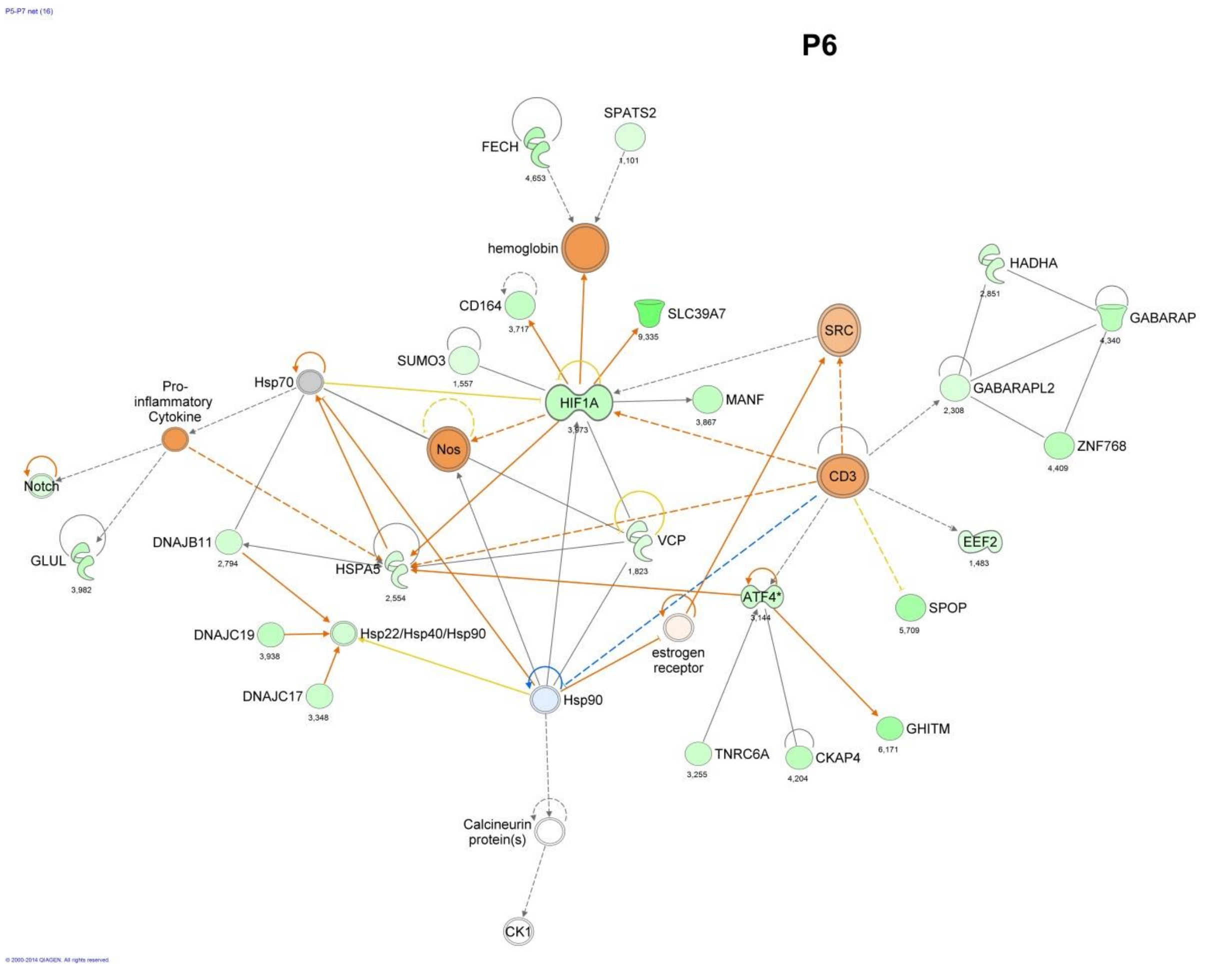

Network 8

Cellular compromise, cellular function and maintenance

P5-P7 net (3)

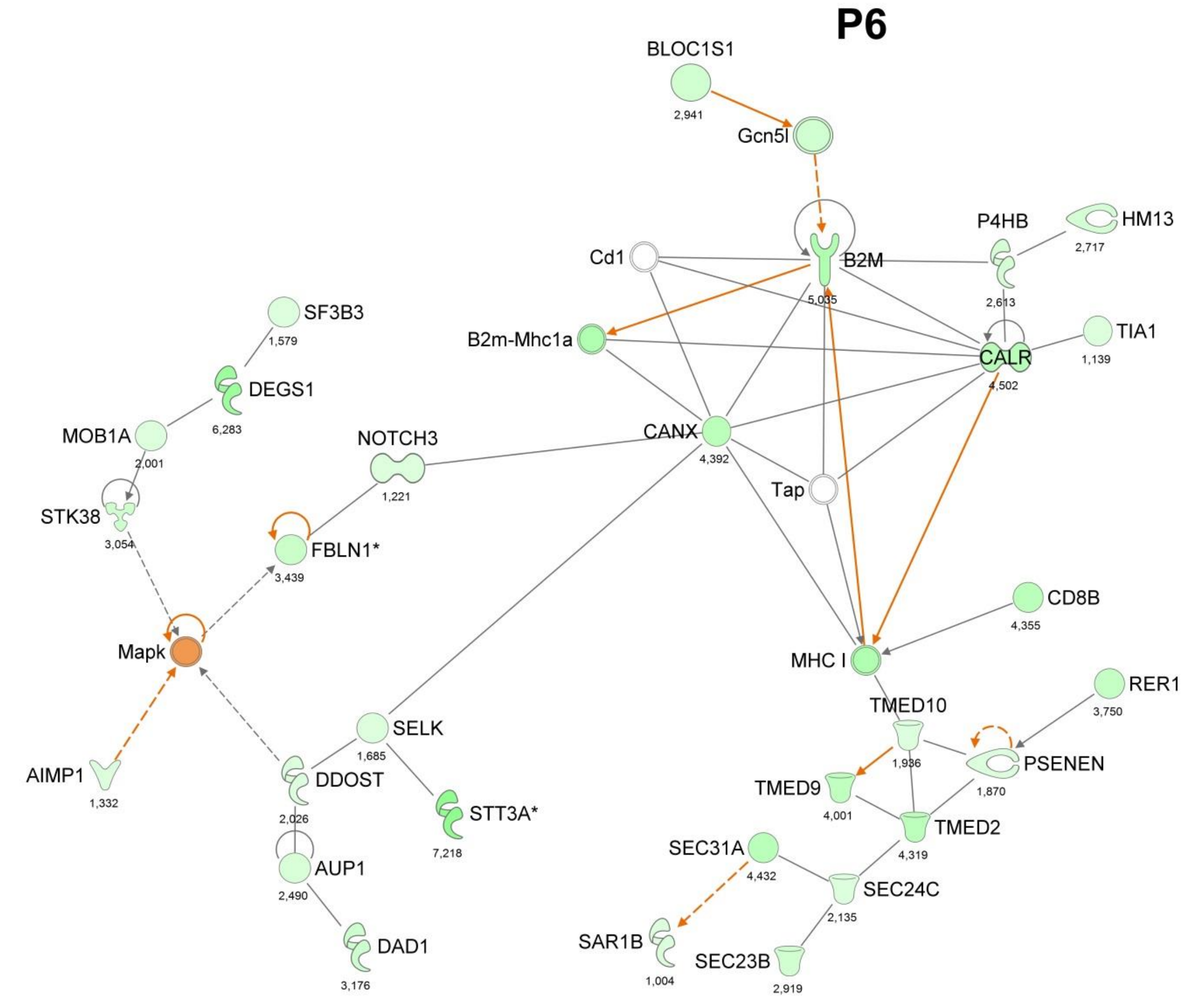

© 2000-2014 QIAGEN. All rights reserved.

Network 9

Cellular assembly and organization, cellular function and maintenance, post-transcriptional modification

P5-P7 net (2)

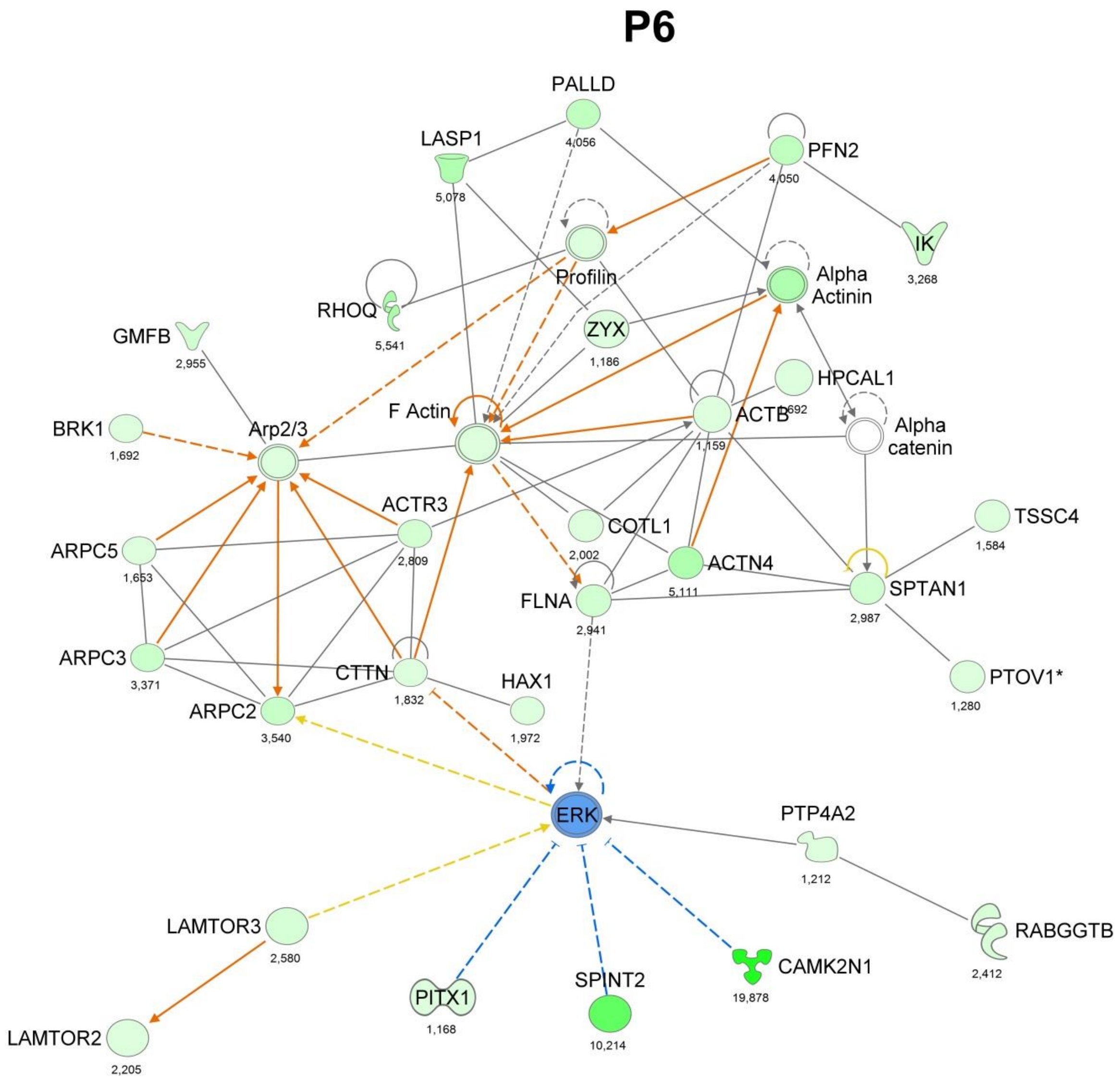

© 2000-2014 QIAGEN. All rights reserved.

Network 10

Cellular assembly and organization, cellular function and maintenance, carbohydrate metabolism

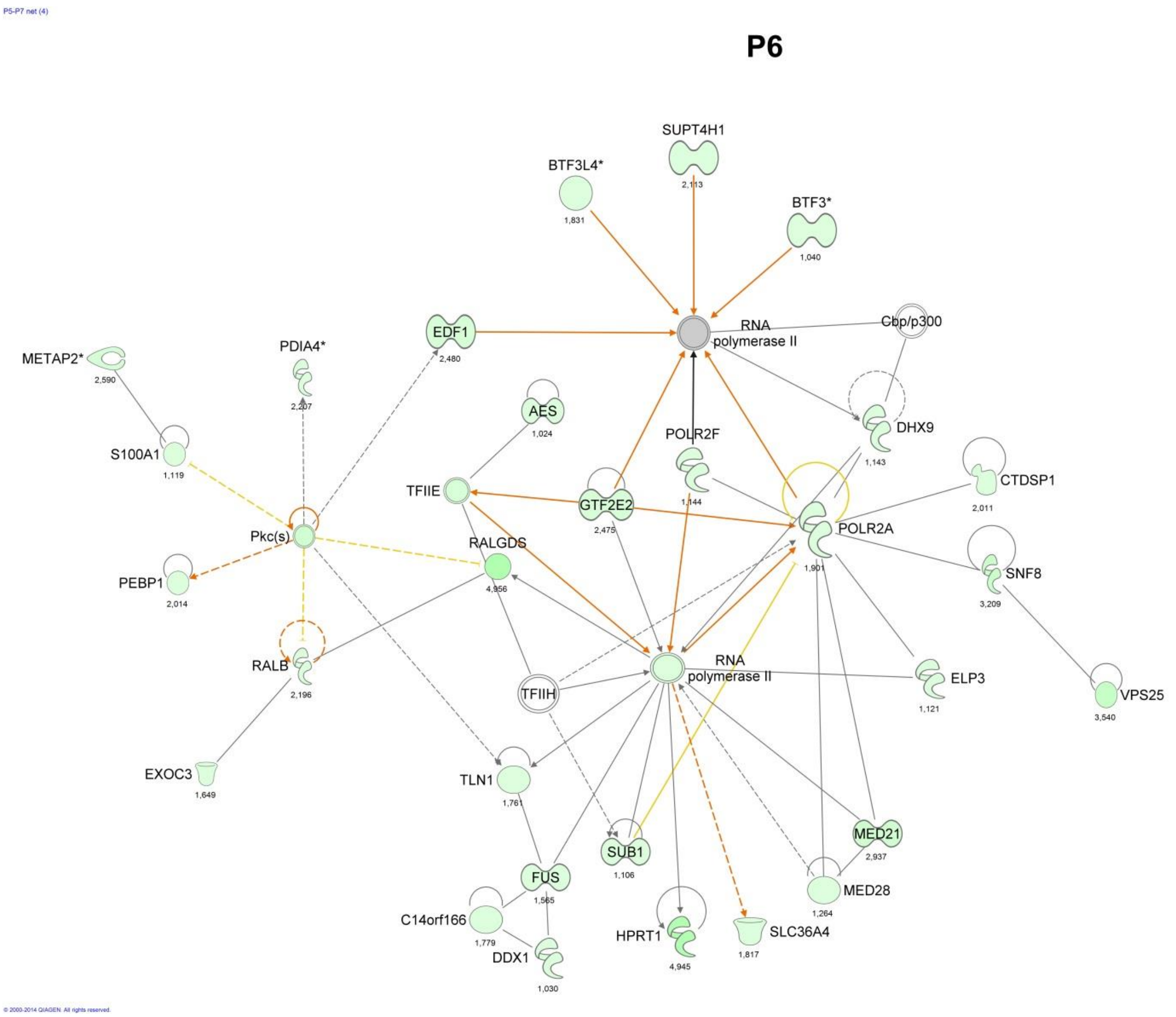

Network 11

Gene epression, DNA replication

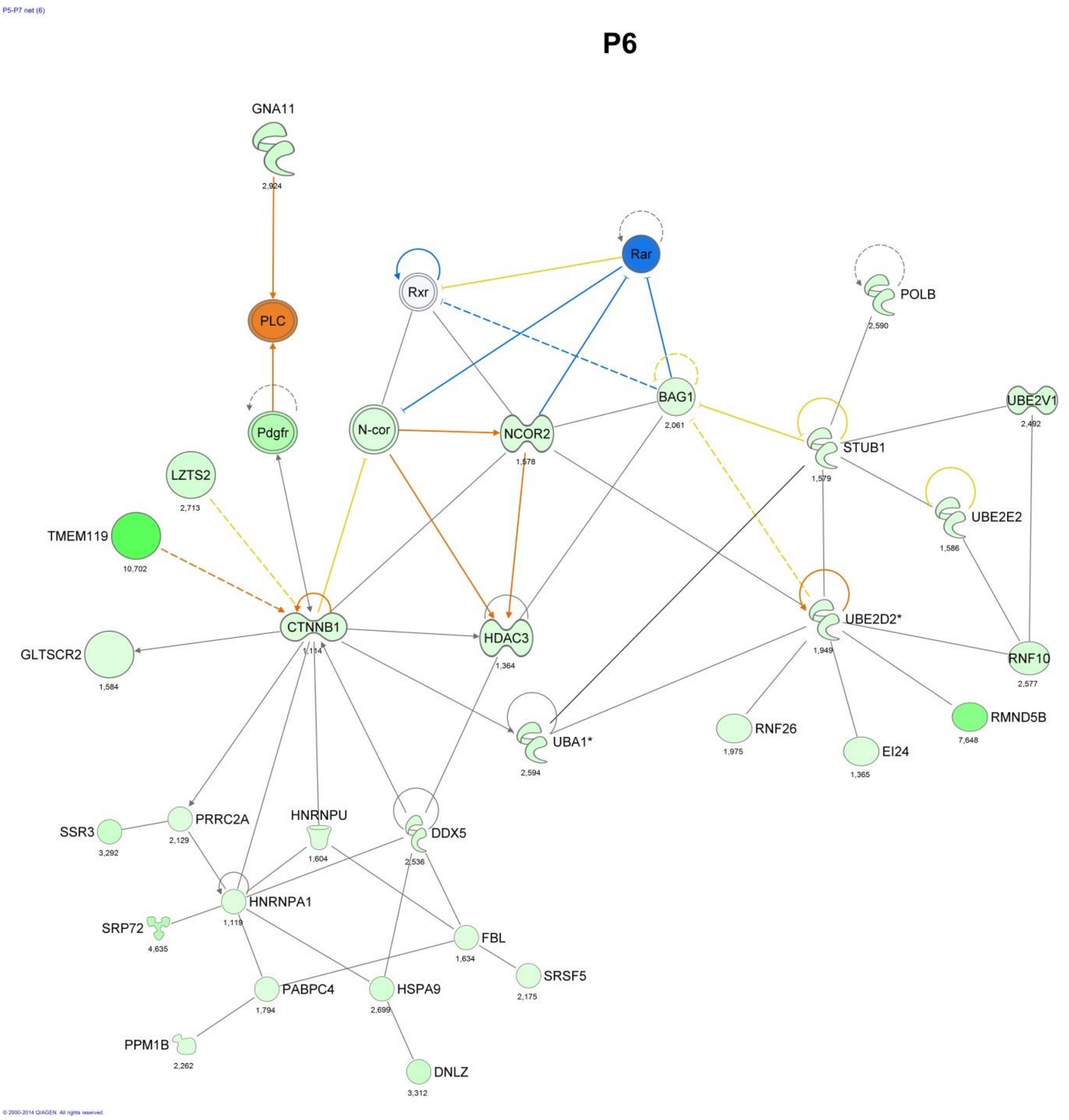

Network 12

RNA post-transcriptional modification, DNA replication

P5-P7 net (8)

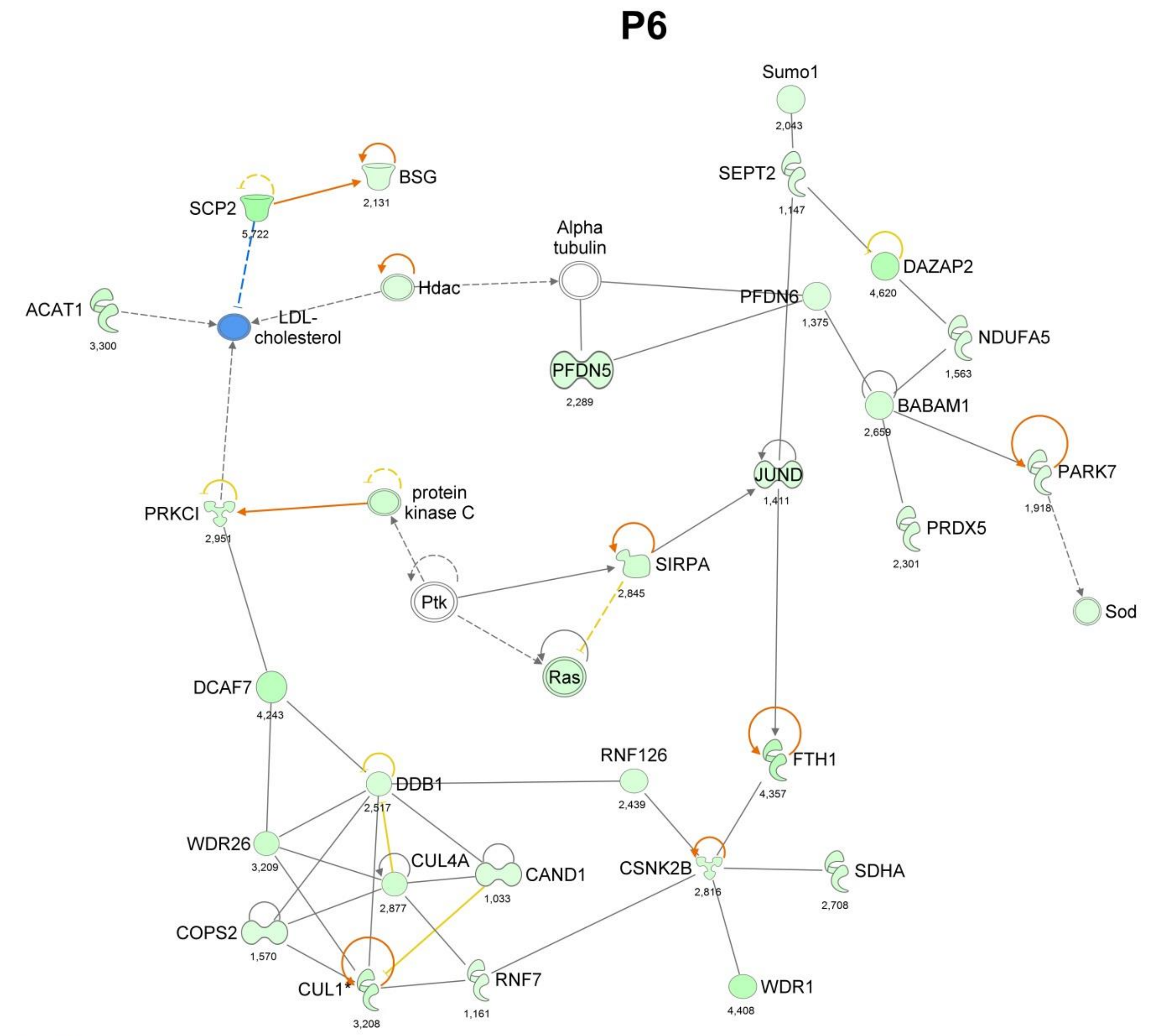

© 2000-2014 QIAGEN. All rights reserved.

Network 13

RNA replication, cellular development, cellular growth and proliferation

P5-P7 net (17)

P6

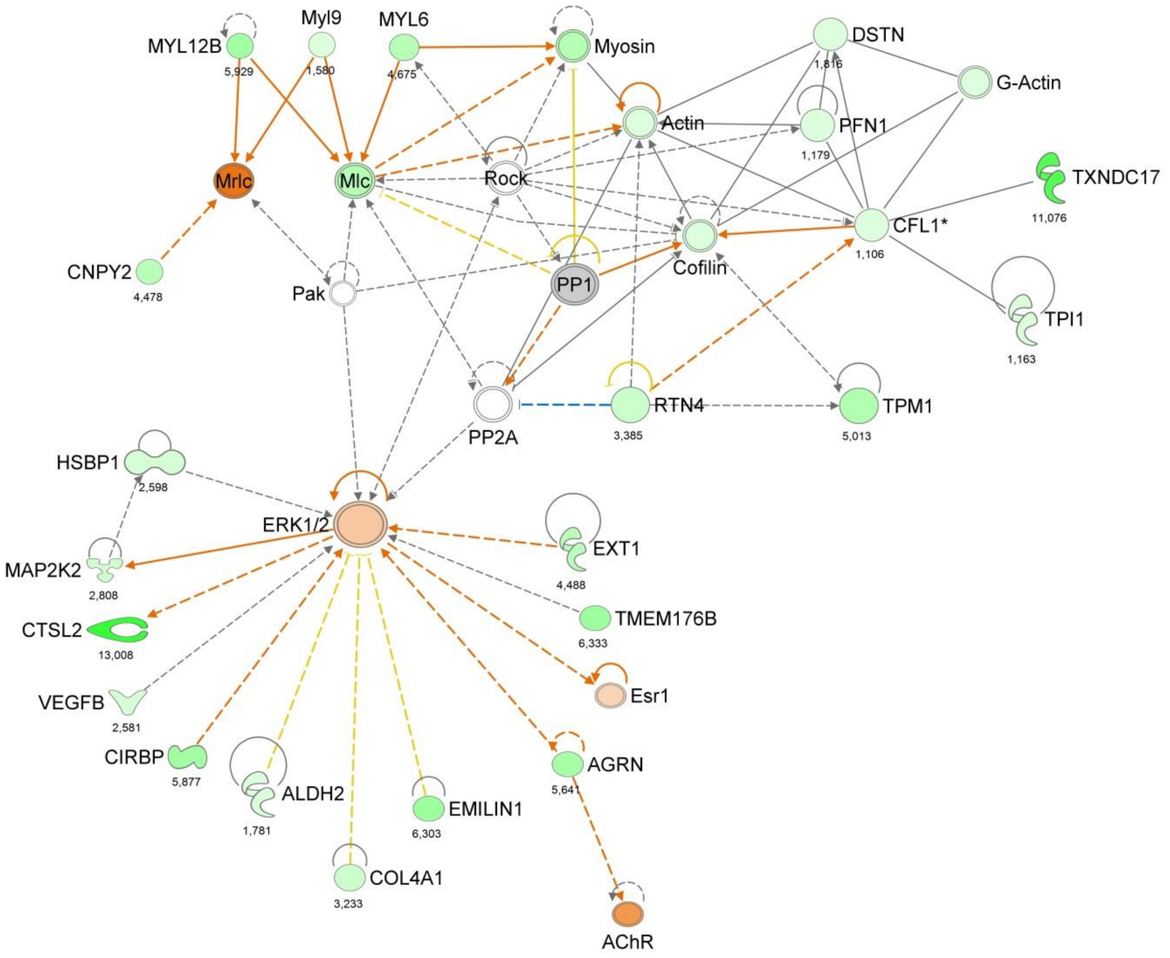

© 2000-2014 QIAGEN. All rights reserved.

Network 14

Cellular assembly and organization, organ morphology

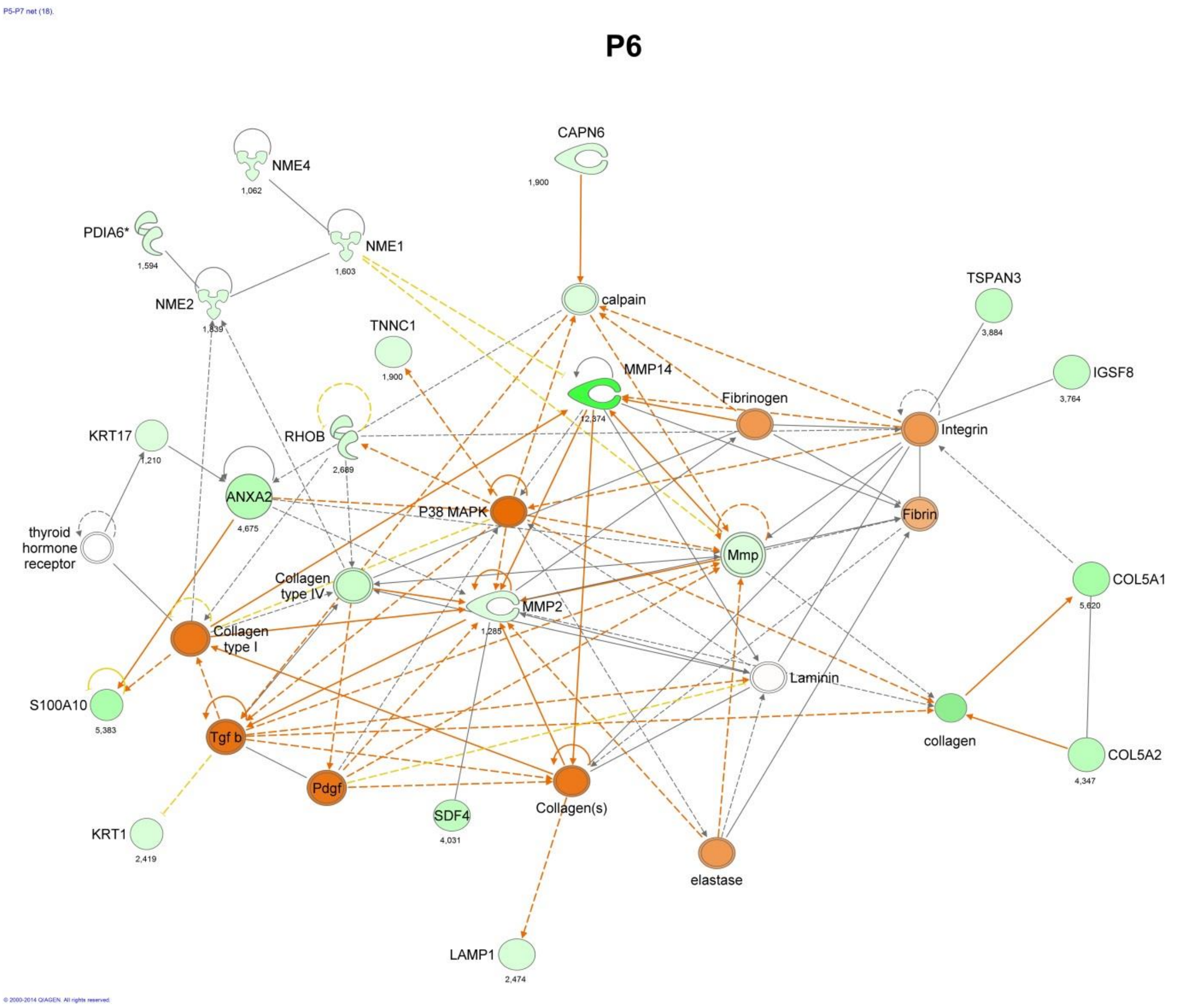

Network 15

Nucleic acid metabolism, small molecule biochemistry

P5-P7 net (8)

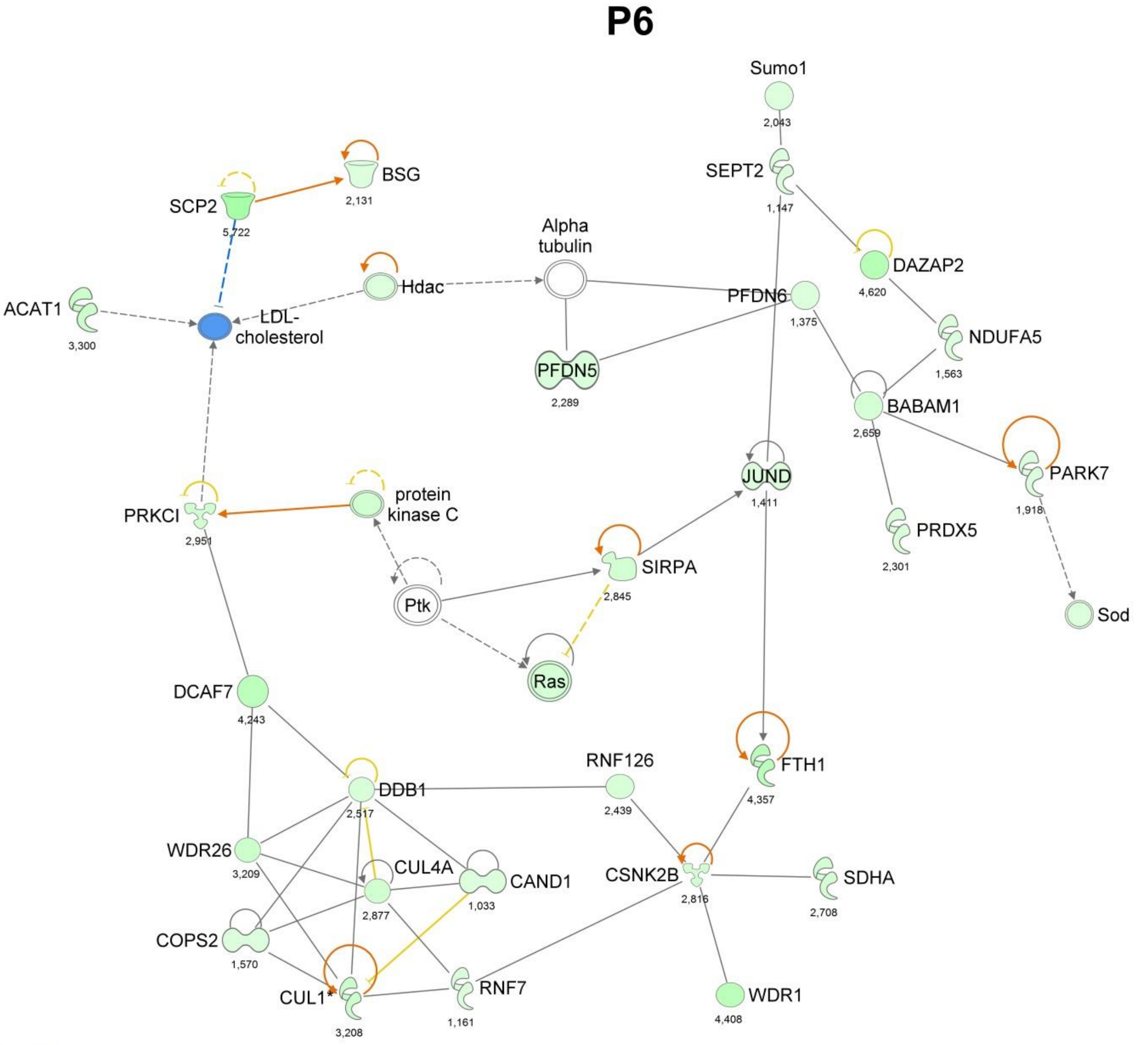

© 2000-2014 QIAGEN. All rights reserved.

Network 16

RNA replication, cellular development, cellular growth and proliferation

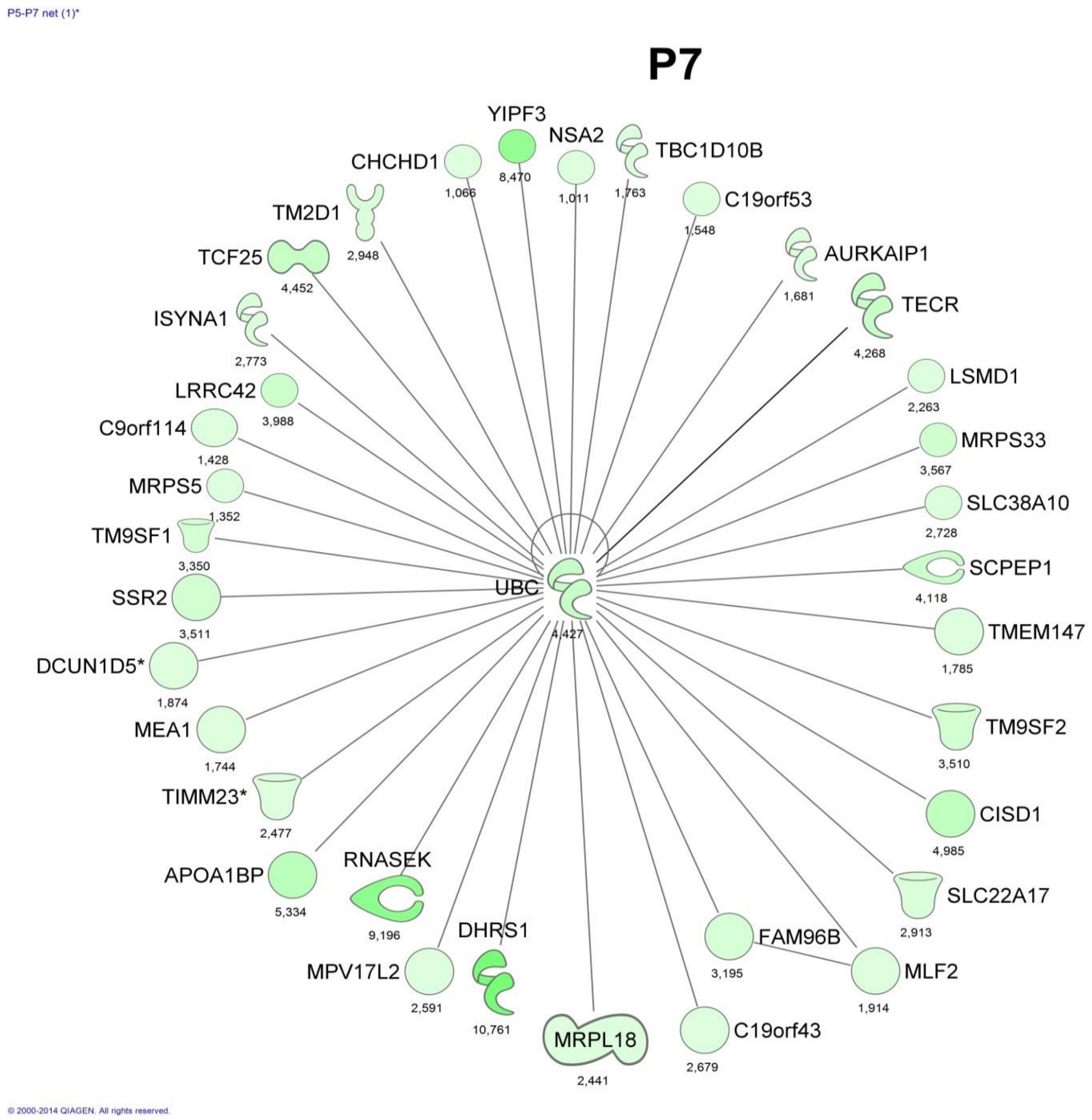

Network 1

Lipid metabolism, nucleic acid metabolism, small molecule biochemistry

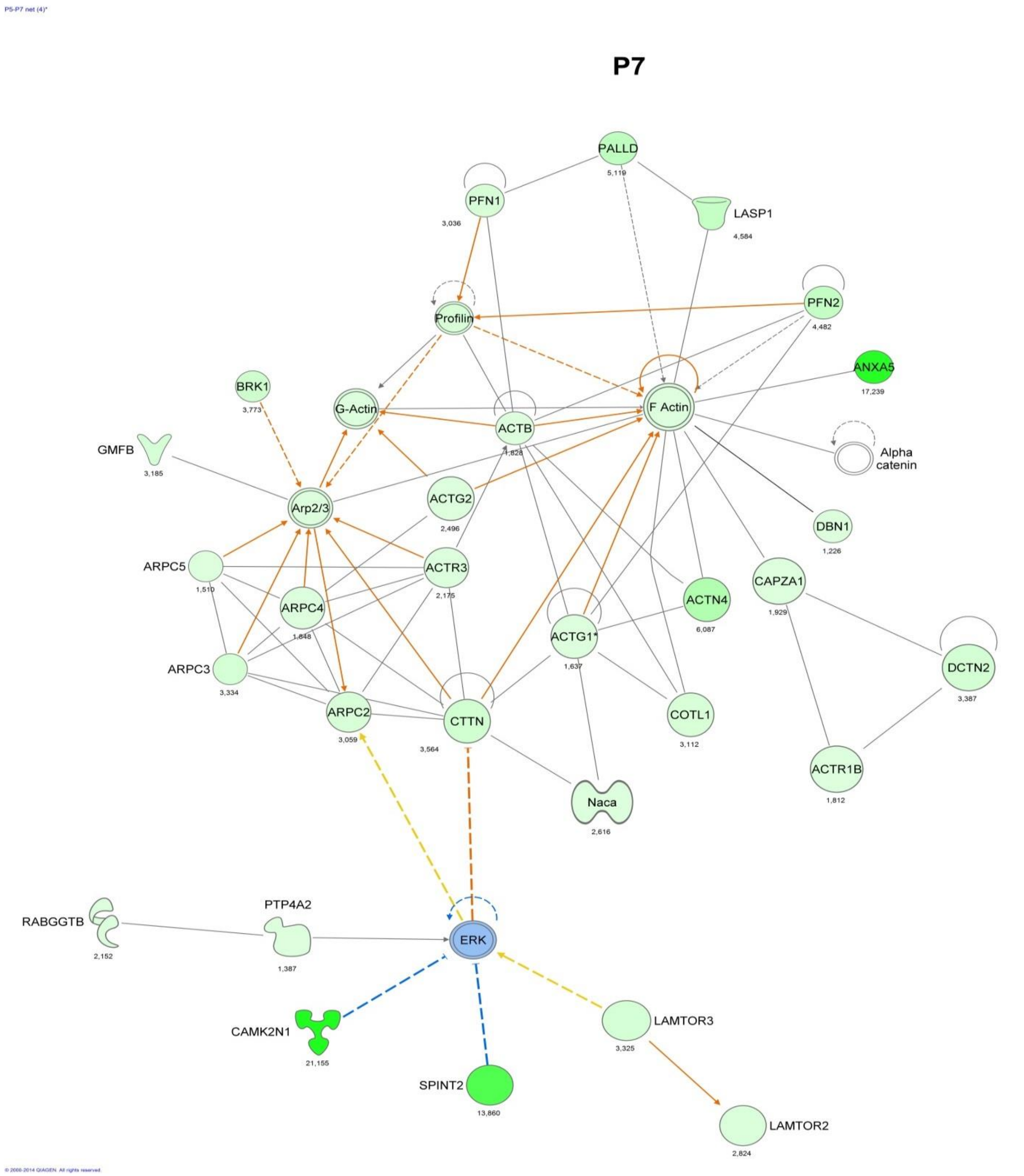

Network 2

Cellular assembly and organization, cellular function and maintenance, protein synthesis

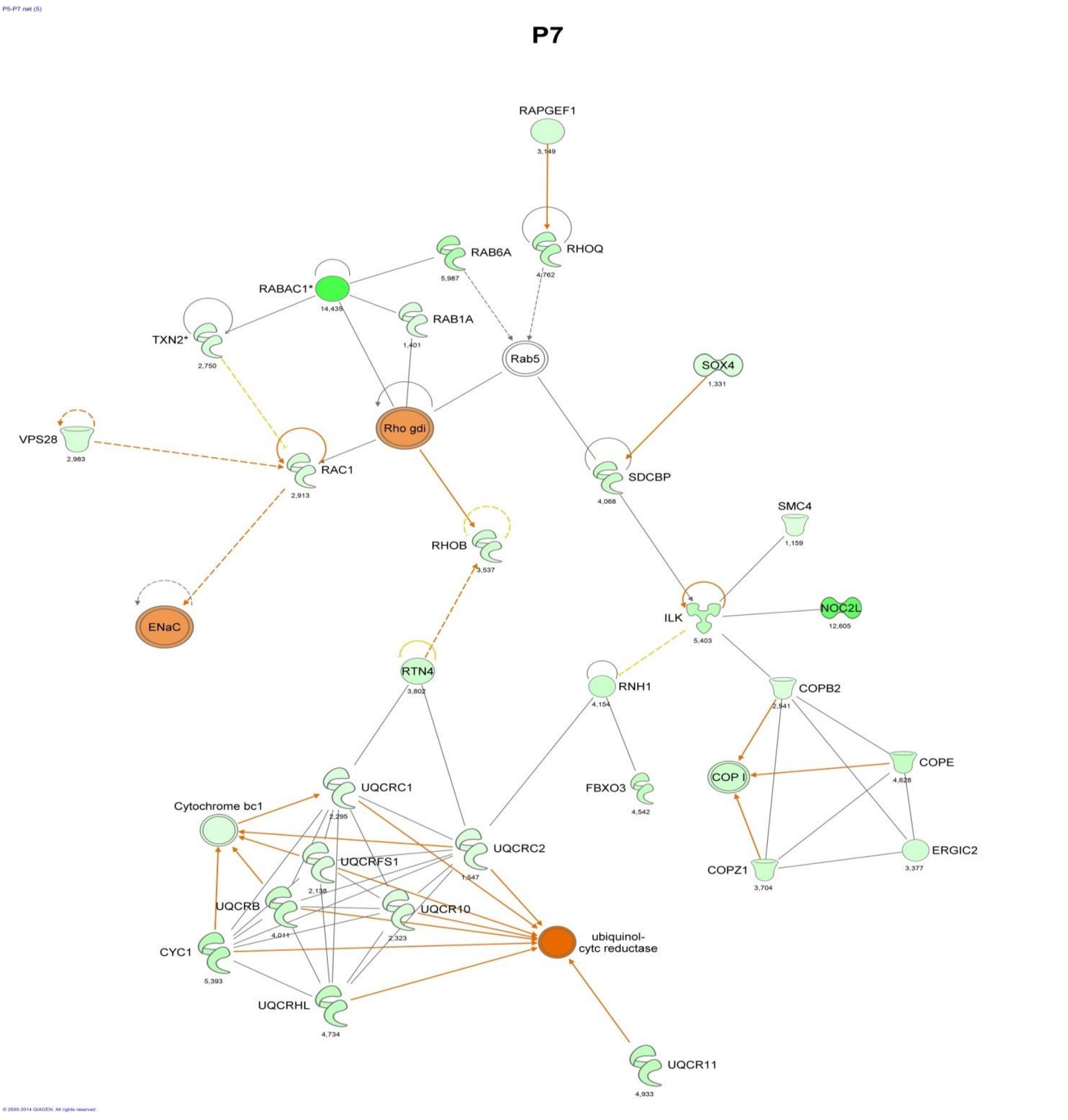

Network 3

Cellular function and maintenance, cell morphology, connective tissue development and function

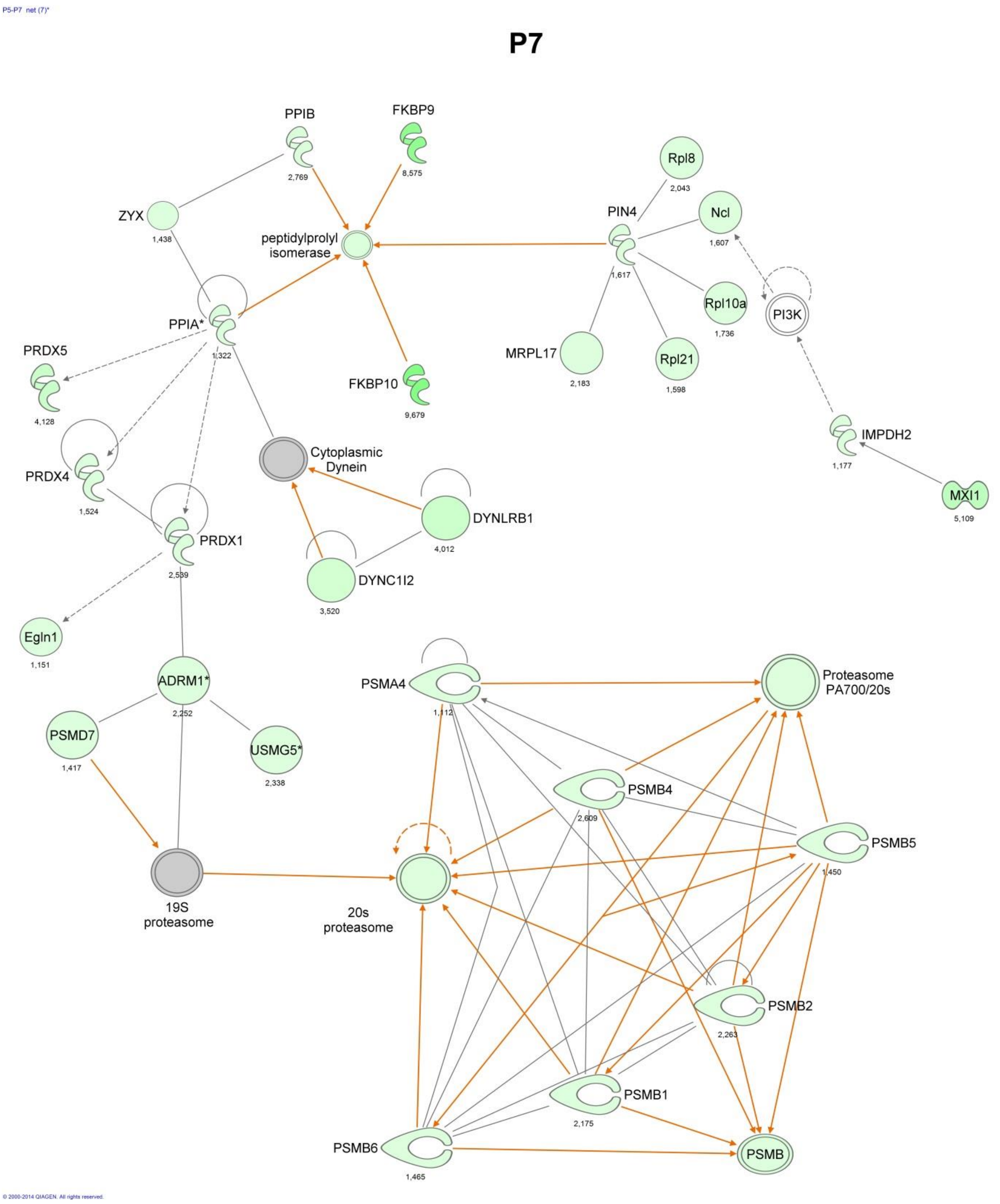

© 2000-2014 QIAGEN. All rights reserved.

Network 4

Cell death, small molecule biochemistry

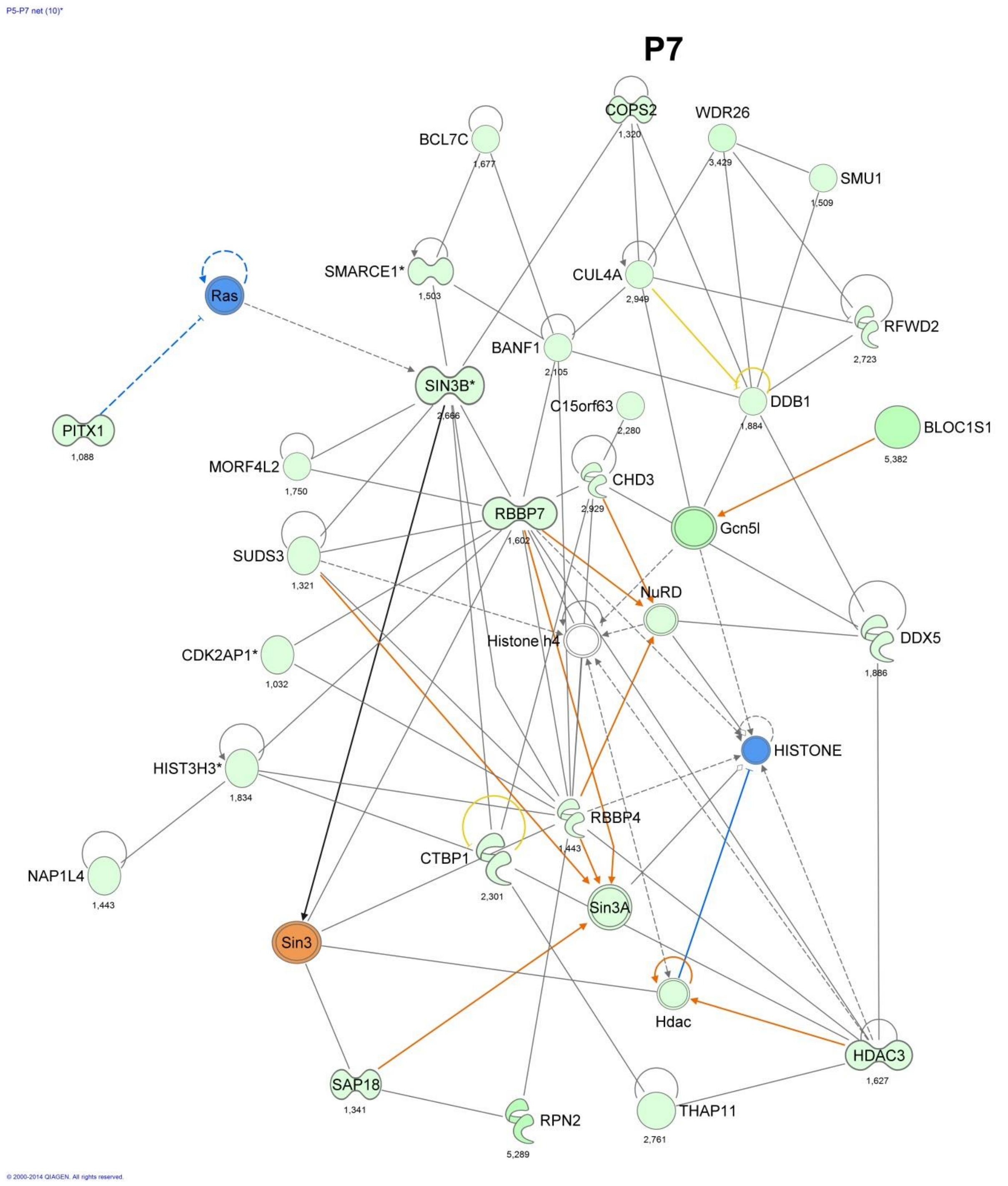

Network 5

Cell cycle, DNA replication, gene expression

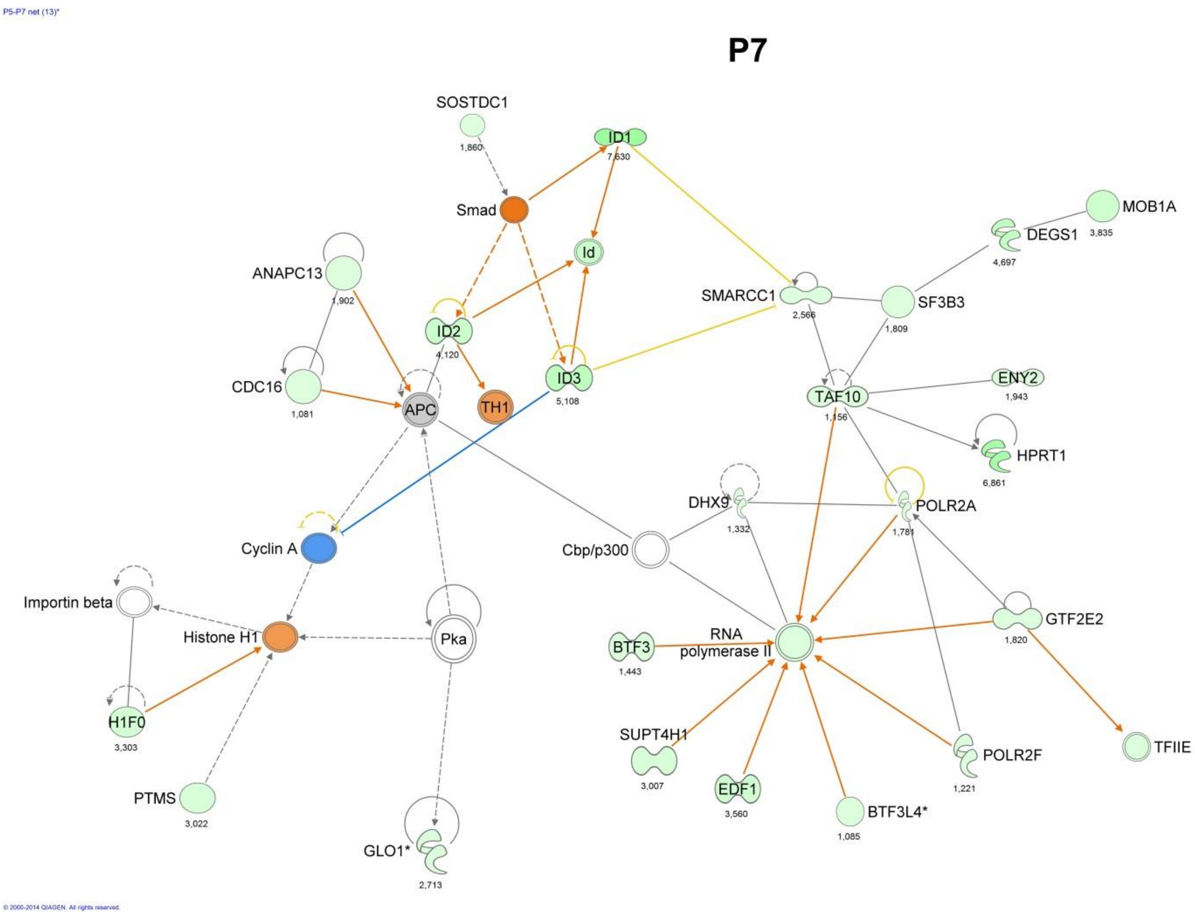

Network 6

Gene expression, cellular development, cellular growth and proliferation

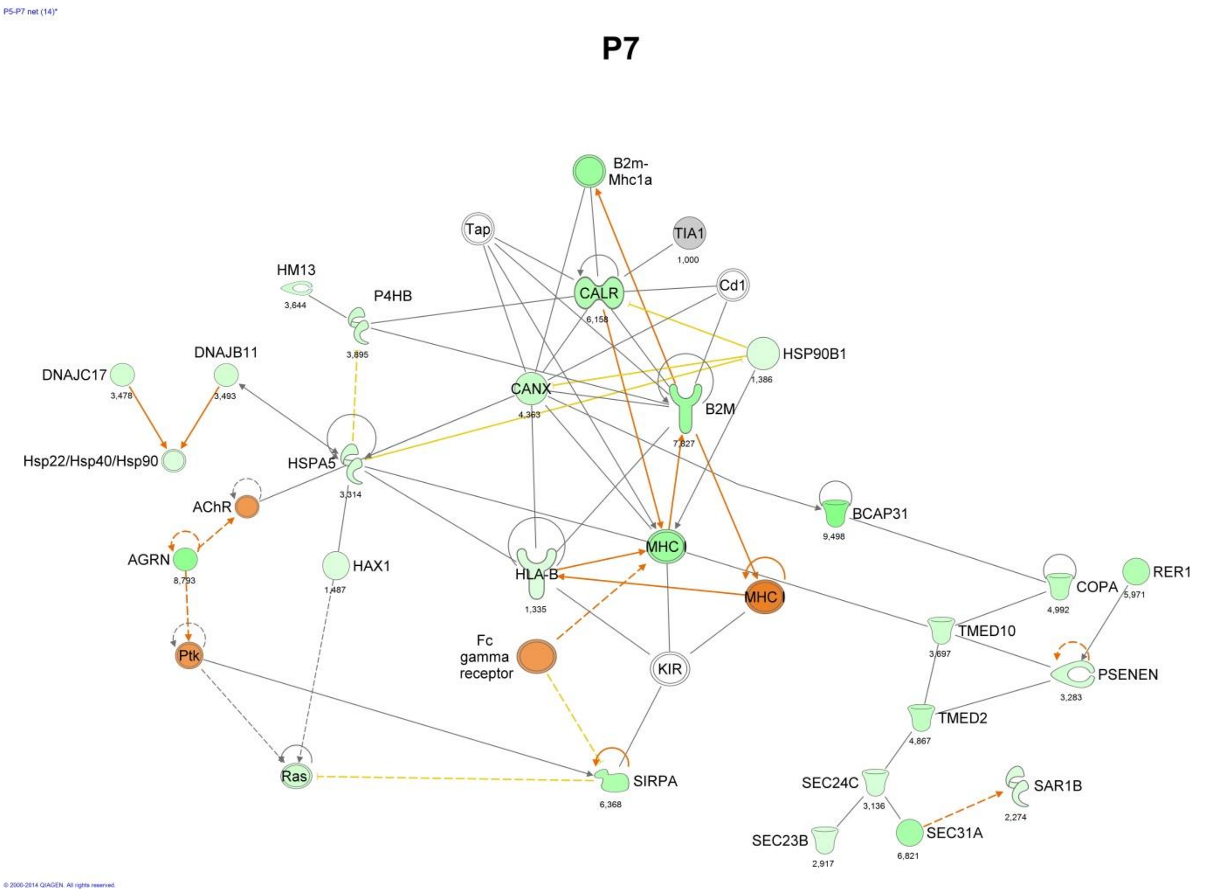

Network 7

Cellular compromise, cellular function and maintenance, post-transcriptional modification

P5-P7 net (15)*

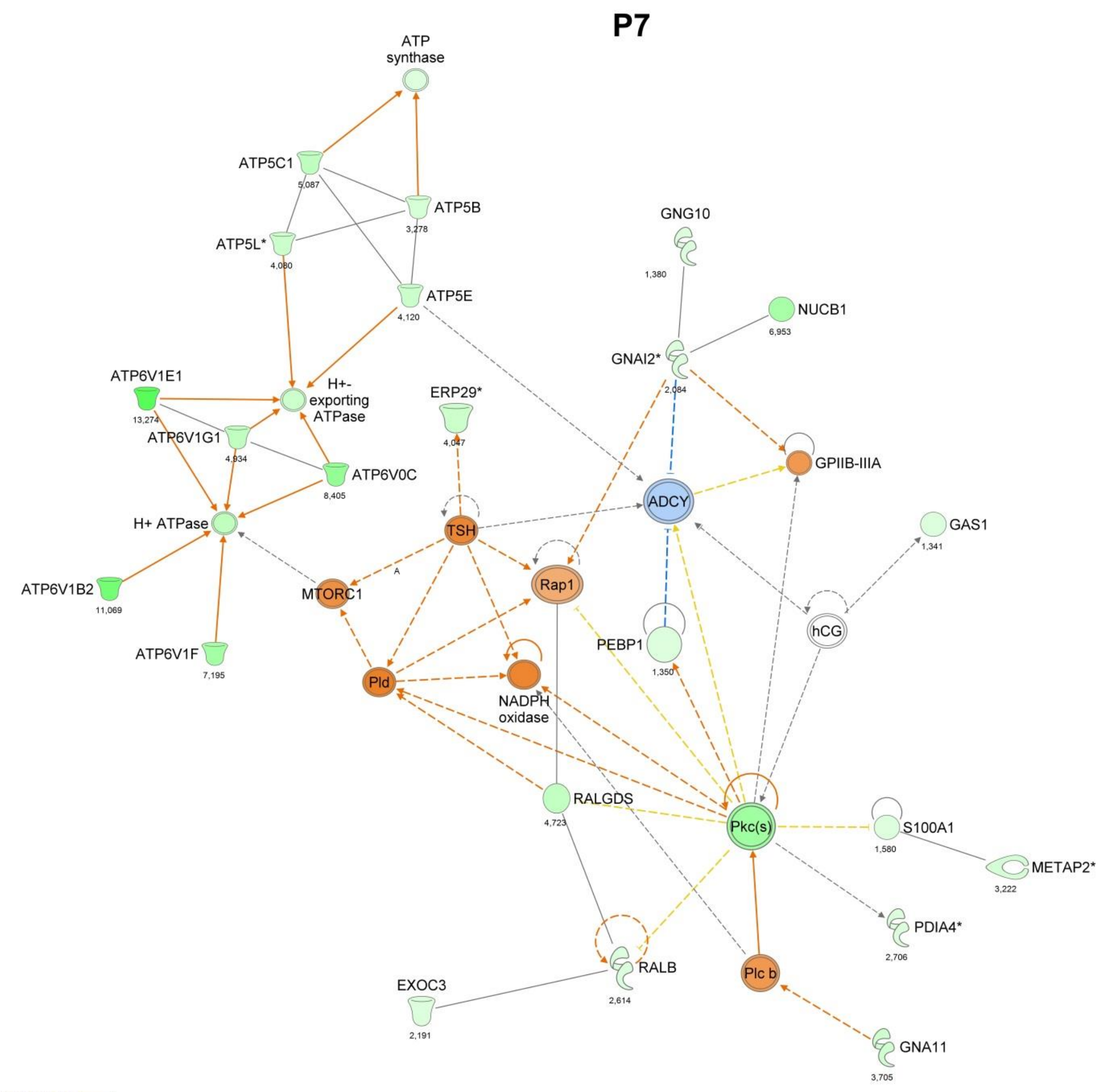

© 2000-2014 QIAGEN. All rights reserved.

Network 8

Molecular transport, DNA replication, energy production

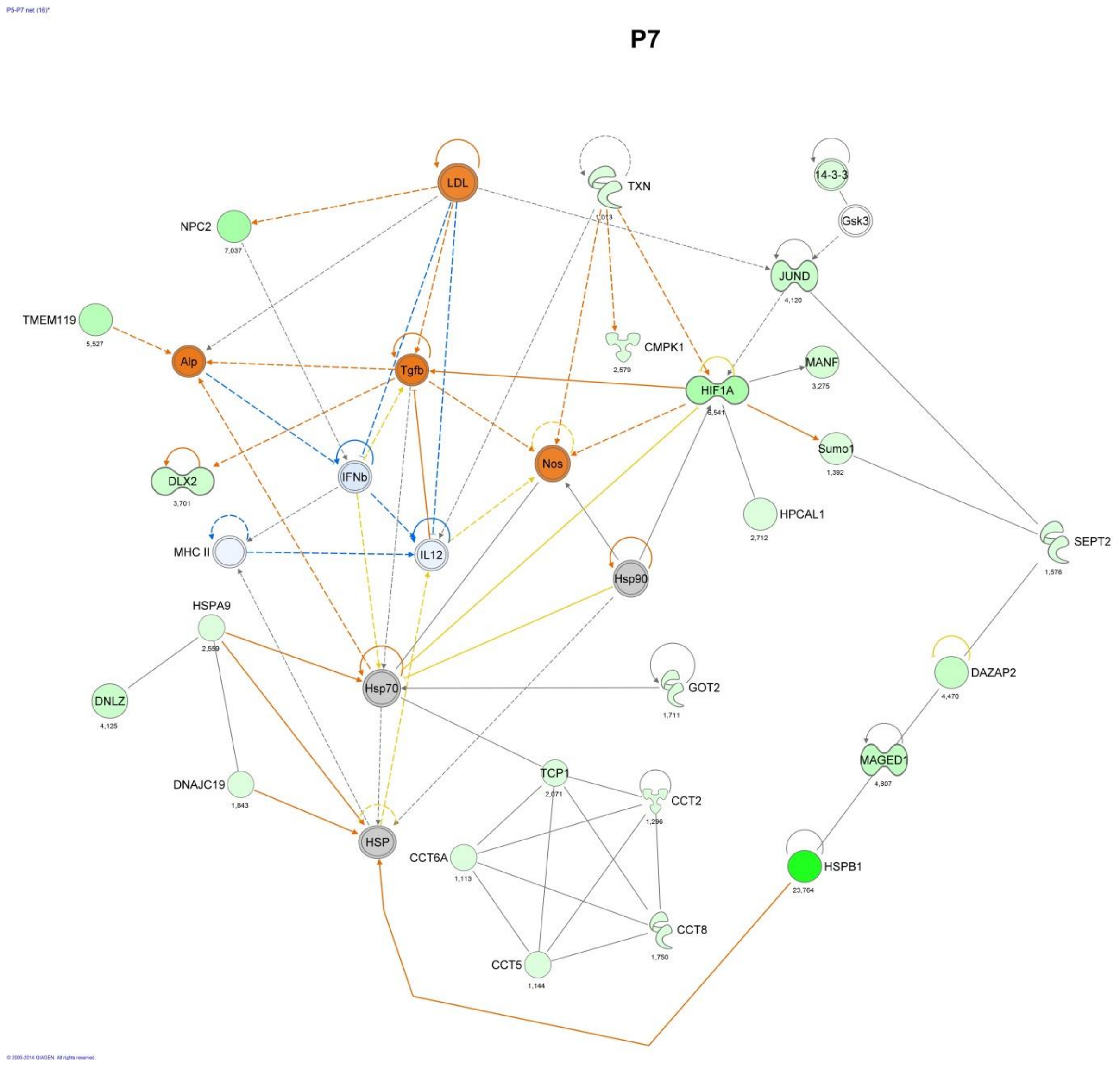

Network 9

Cell death, post-transcriptional modification protein folding

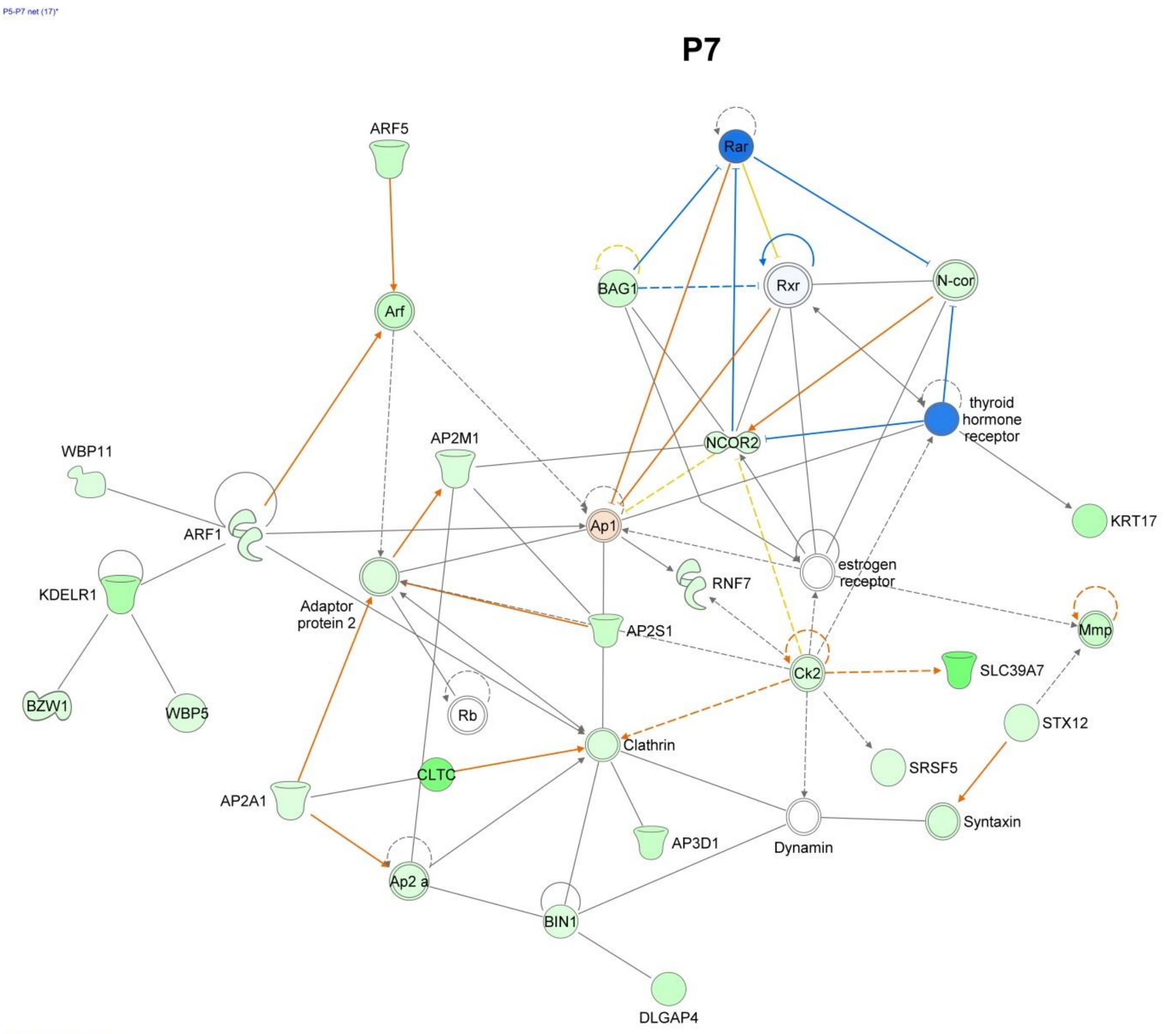

© 2000-2014 QIAGEN. All rights reserved.

Network 10

Molecular transport, protein trafficking, cellular assembly and organization

P5-P7 net (18)*

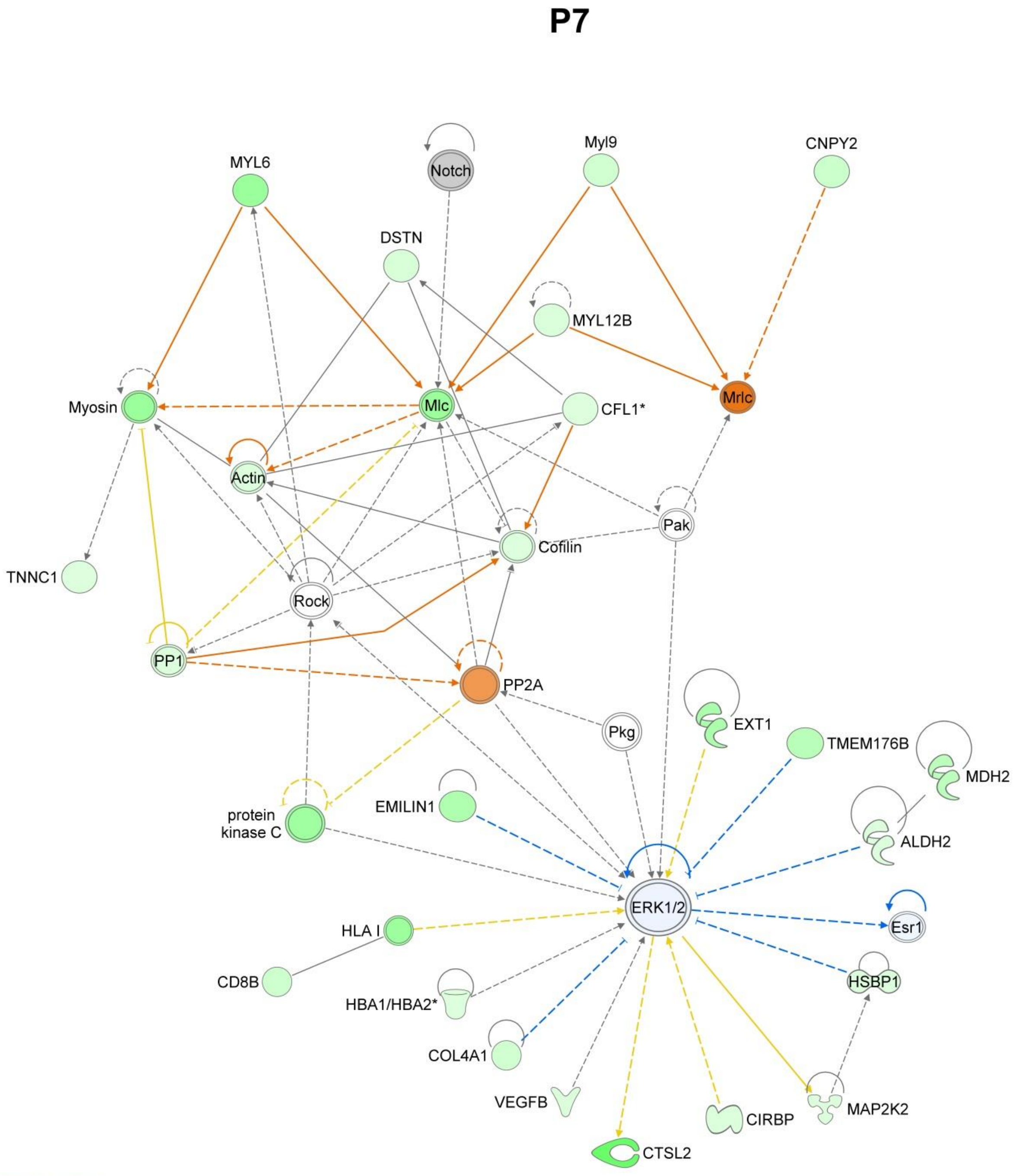

© 2000-2014 QIAGEN. All rights reserved.

Network 11

Cellular assembly and organization, organ morphology, skeletal and muscular system development and function

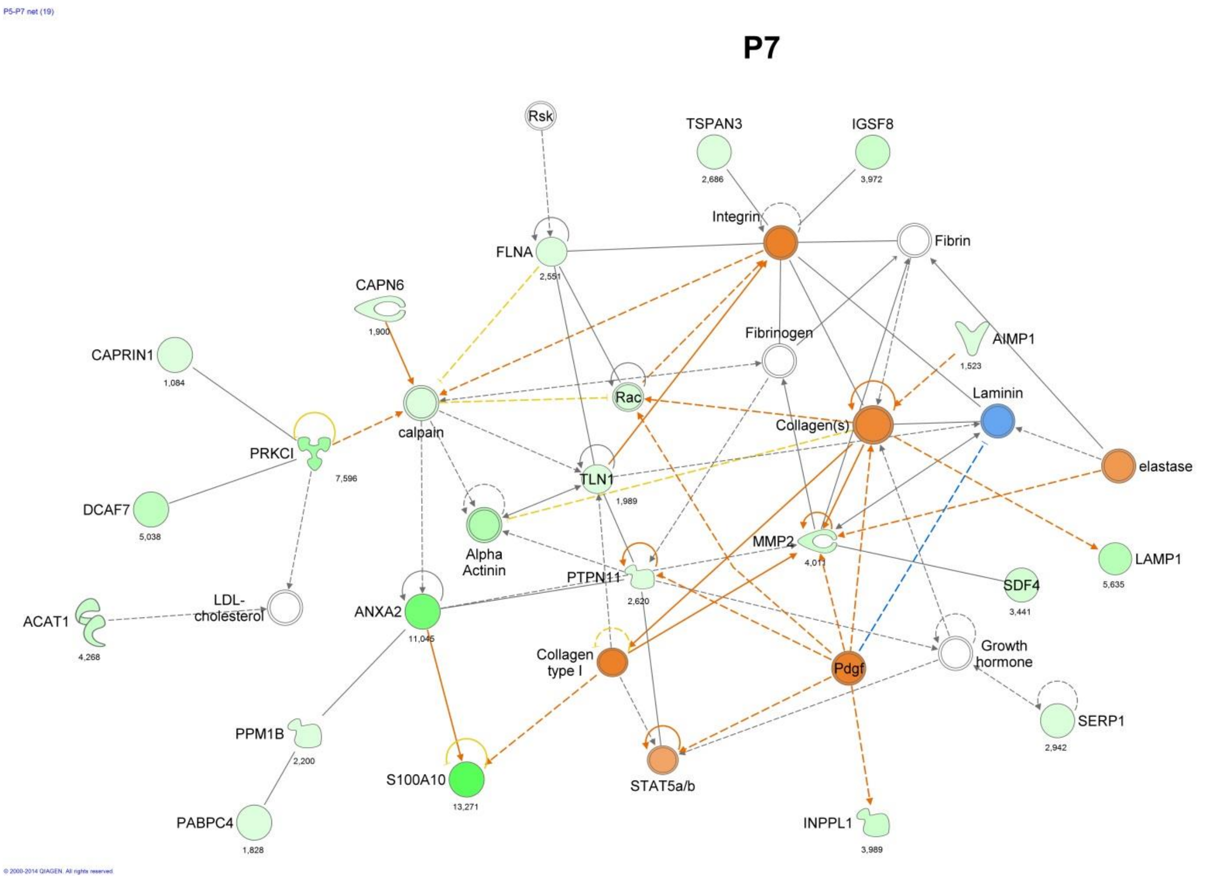

Network 12

Cell morphology, cellular assembly and organization, cellular movment

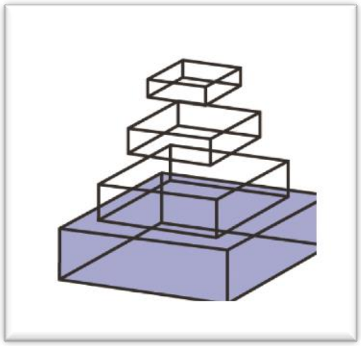

Supplement: Supplementary file 2 [file networkanalysisofpost-natalstages.PDF]
